# Genomically matched therapy in advanced solid tumors: the randomized phase 2 ROME trial

**DOI:** 10.1038/s41591-025-03918-x

**Published:** 2025-09-29

**Authors:** Paolo Marchetti, Giuseppe Curigliano, Mauro Biffoni, Sara Lonardi, Simone Scagnoli, Lorenzo Fornaro, Valentina Guarneri, Ugo De Giorgi, Paolo Antonio Ascierto, Giovanni Blandino, Giulia D’Amati, Massimo Aglietta, Chiara Cremolini, Pierfranco Conte, Edoardo Crimini, Maurizio Ceracchi, Simona Pisegna, Sofia Verkhovskaia, Roberto Bordonaro, Sergio Bracarda, Giovanni Butturini, Lucia Del Mastro, Andrea DeCensi, Agnese Fabbri, Elisabetta Fenocchio, Stefania Gori, Giulio Metro, Annamaria Pessino, Daniele Pozzessere, Fabio Puglisi, Stefano Tamberi, Alberto Zambelli, Donatella Marino, Ettore Capoluongo, Federico Cappuzzo, Bruna Cerbelli, Giuseppe Giannini, Umberto Malapelle, Federica Mazzuca, Marianna Nuti, Giancarlo Pruneri, Maurizio Simmaco, Lidia Strigari, Giuseppe Tonini, Nello Martini, Andrea Botticelli

**Affiliations:** 1 Department of Oncology, IDI-IRCCS, Rome, Italy; 2 European Institute of Oncology, IRCCS, Milan, Italy; 3 Department of Oncology and Hemato-Oncology, University of Milan, Milan, Italy; 4 Department of Oncology and Molecular Medicine, Italian National Institute of Health (ISS), Rome, Italy; 5 Department of Oncology, Veneto Institute of Oncology IOV-IRCCS, Padua, Italy; 6 Department of Radiological, Oncological and Pathological Sciences, Sapienza University of Rome, Rome, Italy; 7 Oncology Unit, AOU Policlinico Umberto I, Rome, Italy; 8 Department of Translational Research and New Technologies in Medicine and Surgery, University of Pisa, and Division of Medical Oncology, Pisa University Hospital, Pisa, Italy; 9 Department of Surgery, Oncology, and Gastroenterology, University of Padua, Padua, Italy; 10 IRCCS Istituto Romagnolo per lo Studio dei Tumori (IRST) ‘Dino Amadori’, Meldola, Italy; 11 Istituto Nazionale Tumori IRCCS Fondazione ‘G. Pascale’, Naples, Italy; 12 Translational Oncology Research Unit, IRCCS Istituto Nazionale Tumori Regina Elena (IRE), Rome, Italy; 13 Candiolo Cancer Institute FPO Istituto di Ricovero e Cura a Carattere Scientifico Candiolo, Candiolo, Italy; 14 Department of Translational Research and New Technologies in Medicine and Surgery, University of Pisa, Pisa, Italy; 15 S. Camillo Hospital IRCCS, Lido di Venezia, Italy; 16 Fondazione Periplo, Cremona, Italy; 17 CMV STAT S.r.l., Rome, Italy; 18 STS, Rome, Italy; 19 Department of Experimental Medicine, Sapienza University of Rome, Rome, Italy; 20 Medical Oncology Unit, ARNAS Garibaldi, Catania, Italy; 21 Oncologia Medica Traslazionale, Azienda Ospedaliera Santa Maria di Terni, Terni, Italy; 22 Department of Hepatopancreatobiliary (HPB) Surgery, Pederzoli Hospital, Peschiera del Garda, Verona, Italy; 23 IRCSS Ospedale Policlinico San Martino, UO Clinica Oncologia Medica, Genoa, Italy; 24 Department of Internal Medicine and Medical Specialties (DIMI), Università di Genova, Genoa, Italy; 25 Department of Medicine and Medical Oncology Unit, E.O. Ospedali Galliera, Genoa, Italy; 26 Medical Oncology and Breast Unit, Department of Oncology and Hematology, Central Hospital of Belcolle, Viterbo, Italy; 27 Department of Oncology, IRCCS Sacro Cuore Don Calabria Hospital, Negrar di Valpolicella, Verona, Italy; 28 Medical Oncology, Santa Maria della Misericordia Hospital, University of Perugia, Perugia, Italy; 29 Oncologia Medica 1, IRCSS Ospedale Policlinico San Martino, Genoa, Italy; 30 Medical Oncology Unit, Nuovo Ospedale di Prato-Santo Stefano, Azienda USL Toscana Centro, Prato, Italy; 31 Department of Medical Oncology, CRO Aviano, National Cancer Institute, IRCCS, Aviano, Italy; 32 Department of Medicine, University of Udine, Udine, Italy; 33 Oncology Unit, Santa Maria delle Croci Hospital, AUSL Romagna, Ravenna, Italy; 34 Oncology Unit, ASST Papa Giovanni XXIII, Bergamo, Italy; 35 Department of Medicine and Surgery, Università degli Studi di Milano Bicocca, Milan, Italy; 36 Department of Oncology, Ordine Mauriziano Hospital, Turin, Italy; 37 Dipartimento Di Eccellenza In Medicina Molecolare E Biotecnologie Mediche, Università Federico II, Naples, Italy; 38 Division of Medical Oncology 2, IRCCS Regina Elena National Cancer Institute, Rome, Italy; 39 Department of Molecular Medicine, Sapienza University of Rome, laboratory affiliated to Istituto Pasteur Italia, Fondazione Cenci-Bolognetti, Rome, Italy; 40 Department of Public Health, University Federico II of Naples, Naples, Italy; 41 Department of Clinical and Molecular Medicine, Sapienza University, Oncology Unit, Azienda Ospedaliera Universitaria Sant’Andrea, Rome, Italy; 42 Department of Experimental Medicine, ‘Sapienza’ University of Rome, Rome, Italy; 43 Department of Advanced Diagnostics, Fondazione IRCCS Istituto Tumori di Milano, University of Milan, Milan, Italy; 44 Laboratory of Clinical Biochemistry, Sant’Andrea University Hospital, Rome, Italy; 45 Department of Neurosciences, Mental Health and Sensory Organs (NESMOS), Sapienza University of Rome, Rome, Italy; 46 Department of Medical Physics, IRCCS Azienda Ospedaliero-Universitaria di Bologna, Bologna, Italy; 47 Department of Medicine and Surgery, Università Campus Bio-Medico, Rome, Italy; 48 Medical Oncology, Fondazione Policlinico Universitario Campus Bio-Medico, Rome, Italy; 49 Fondazione ReS, Rome, Italy; 50 Medical Oncology 1, IRCCS Regina Elena National Cancer Institute, Rome, Italy; 51 Clinical Trial Center, Oncology Department A.O., Papardo-Messina, Italy; 52 Medical Oncology Unit, RCCS Istituto Tumori ‘Giovanni Paolo II’, Bari, Italy; 53 Department of Experimental and Clinical Medicine, Magna Græcia University, Catanzaro, Italy; 54 Centro Oncologico San Leopoldo Mandic, Isola Tiberina Gemelli isola, Roma, Italy; 55 Department of Medical Oncology, University Hospital, Ferrara, Italy; 56 Division of Medical Oncology 1, St. Chiara University Hospital, Pisa, Italy; 57 Department of Oncology and Hematology, AUSL Piacenza Guglielmo da Saliceto Hospital, Piacenza, Italy; 58 Medical Oncology Unit, Comprehensive Cancer Centre, AUSL-IRCCS di Reggio Emilia, Reggio Emilia, Italy; 59 Nervous System Medical Oncology Department, IRCCS Istituto delle Scienze Neurologiche di Bologna, Bologna, Italy; 60 Medical Oncology, Humanitas Gradenigo, Torino, Italy; 61 Oncology Unit, Foundation IRCCS Casa Sollievo della Sofferenza, San Giovanni Rotondo, Italy; 62 Oncology Unit, Ramazzini Hospital, Azienda Unità Sanitaria Locale Modena (AUSL), Carpi, Italy; 63 Dipartimento di Medicina di Precisione in Area Medica, Chirurgica e Critica (Me.Pre.C.C), Università degli studi di Palermo AOUP ‘Paolo Giaccone’, Palermo, Italy; 64 Clinica Oncologica, Università Politecnica delle Marche – AOU delle Marche, Ancona, Italy; 65 Medical Oncology 2, Istituto Oncologico Veneto IOV IRCCS, Padova, Italy

**Keywords:** Cancer genomics, Cancer models, Cancer immunotherapy, Targeted therapies, Drug development

## Abstract

Despite recent advancements demonstrating the potential of tumor-agnostic biomarkers to guide effective therapies, randomized evidence supporting the clinical superiority of precision oncology approaches compared to standard therapies remains limited. The ROME trial was a multicenter, randomized, open-label phase 2 study comparing tailored treatment (TT) to standard of care (SoC) in patients with advanced solid tumors progressing after one or two lines of therapy. Comprehensive genomic profiling on tissue and blood was performed to identify actionable alterations. Overall response rate (ORR) was the primary endpoint, and progression-free survival (PFS), overall survival (OS), time to treatment failure (TTF), time to next treatment (TTNT) and safety were the secondary endpoints. Between November 2020 and August 2023, 1,794 patients were screened, 897 were evaluated by the molecular tumor board (MTB) and 400 were randomized to TT or SoC. TT achieved a significantly higher ORR (17.5% versus 10%; *P* = 0.0294) and improved median PFS (3.5 months versus 2.8 months; hazard ratio = 0.66 (0.53–0.82), *P* = 0.0002). TT also showed superior 12-month PFS rates (22.0% versus 8.3%). Median OS was similar, with a 52% crossover rate. Grade 3/4 adverse events were also similar (40% TT versus 52% SoC). These results highlight the potential of TT to improve outcomes for patients with diverse actionable genomic alterations. These results also provide relevant evidence supporting a tumor-agnostic precision oncology strategy and highlight the potential of TTs, guided by genomic profiling and MTB recommendations, to significantly improve outcomes for patients with diverse actionable genomic alterations. ClinicalTrials.gov identifier: NCT04591431.

## Abstract (web summary)

In the proof-of-concept phase 2 ROME trial, comprehensive genomic profiling followed by molecular tumor board evaluation and randomization of patients with metastatic solid cancer to receive personalized therapy or standard of care led to a significantly higher objective response rate and longer progression-free survival in patients who received personalized therapy.

## Main

Precision oncology aims to enhance clinical outcomes by tailoring cancer treatments to individual patients, focusing on specific vulnerabilities identified through the analysis of cancer characteristics and the patient’s clinical history^1^. However, implementing these strategies presents major challenges, such as detecting actionable biomarkers, interpreting their clinical significance within the context of an individual patient’s history and systematically utilizing available tailored treatments (TTs). This complexity is further compounded by the variability in the frequency of actionable molecular alterations across different tumor types, making histology a less reliable factor for determining treatment options^2^. Additionally, standard therapeutic options vary considerably across different tumor types. As a result, the clinical significance of an actionable genetic alteration can differ substantially across various cancer types^3^.

Among the different approaches for detailed molecular characterization of tumor samples, genomic tests are the most widely used in clinical trials to identify patients with actionable alterations.

Recent advancements have uncovered that treatments targeting some tumor-agnostic biomarkers induce considerable clinical benefits regardless of histology. Notable examples include pembrolizumab^4^ and dostarlimab^5^ for tumors exhibiting high microsatellite instability (MSI-H); pembrolizumab for high tumor mutational burden (hTMB)^6^; larotrectinib^7^, entrectinib^8^ and repotrectinib^9^ for tumors harboring *NTRK* gene fusions; dabrafenib and trametinib for *BRAF* V600 mutation-positive tumors^10^; selpercatinib for *RET* fusions^11^; and trastuzumab deruxtecan for HER2-overexpressing (3+) solid tumors^12^.

However, definitive evidence demonstrating the superiority of these approaches over standard therapies is lacking, with inconsistent outcomes reported across randomized agnostic precision oncology clinical trials. For instance, although the SHIVA trial did not show progression-free survival (PFS) benefits for off-label TT compared to standard treatments^13^, the MOSCATO-01 trial achieved its endpoint, underscoring the value of broad genomic testing and targeted drug treatments^14^.

Large, prospective, non-randomized trials are being conducted to identify molecularly defined subpopulations of patients deriving benefit from TTs. The results of multiple cohorts of the Targeted Agent and Profiling Utilization Registry (TAPUR) study were published^15–18^. Similarly, the MyPathway and the NCI-MATCH trials provided valuable insights into the actionability of molecular findings in multiple tumor types^19–21^.

The European Society for Medical Oncology (ESMO) sought to address these challenges by developing tools such as ETAC-S, which contains minimum requirements for identifying TTs with tumor-agnostic potential based on mechanistic evidence paired with prospective clinical insights^22^. Another widely implemented framework is the ESMO Scale for Clinical Actionability of molecular Targets (ESCAT)^23^, which ranks genomic aberrations to guide therapeutic decisions. The SAFIR02-BREAST trial demonstrated the utility of ESCAT, indicating that only patients harboring genomic alterations ranked as tier I and tier II benefit from maintenance TT, whereas those harboring alterations with lower actionability levels do not^24^.

Moreover, molecular tumor boards (MTBs) provide essential multidisciplinary expertise for implementing precision medicine^25^. However, the impact of MTBs on patient outcomes requires further validation, as current evidence is limited and varies based on factors such as drug availability and tumor types treated^26–29^. Despite these advancements, important hurdles remain in effectively implementing precision oncology in Italy^30^. Although regulatory approvals for targeted therapies have increased recently, many MTB-recommended treatments continue to face narrow or delayed approvals, limiting patient access to TTs even when actionable alterations are detected. Consequently, referral to clinical trials often becomes a viable option for patients to access TTs. Challenges such as limited enrollment availability and strict inclusion criteria further hinder the efficiency of this approach. Therefore, collaborative policies are essential to overcome these issues and facilitate the implementation of precision oncology in clinical practice^25^.

The nationwide ROME trial was designed to provide comprehensive genomic profiling and TT for patients with solid tumors across multiple medical oncology units in Italy. This trial aims to evaluate the outcomes of a TT established after discussions in the MTB compared to the standard of care (SoC). Additionally, this approach seeks to generate high-quality data to identify potentially effective treatments that deserve further clinical validation in specific biomarker-defined subsets of patients.

## Results

### Patient enrollment and baseline characteristics

From 13 October 2020 to 19 July 2023, 1,794 patients with Eastern Cooperative Oncology Group Performance Status (ECOG PS) of 0 or 1 and metastatic solid tumors, who had received up to two lines of therapy, were accrued across 40 oncological centers in Italy. These patients were screened for genomic alteration on tissue and peripheral blood with FoundationOne CDx and FoundationOne Liquid CDx centralized next-generation sequencing (NGS). A total of 897 patients with potentially actionable molecular alterations were identified and discussed during 127 weekly MTB meetings. Patients with mutations deemed actionable by at least one of the drugs available in the trial were subsequently randomized in a 1:1 ratio to received TT versus SoC. In total, 497 patients were excluded from randomization based on MTB directives outlined in Methods.

**Extended Data Fig. 1:**
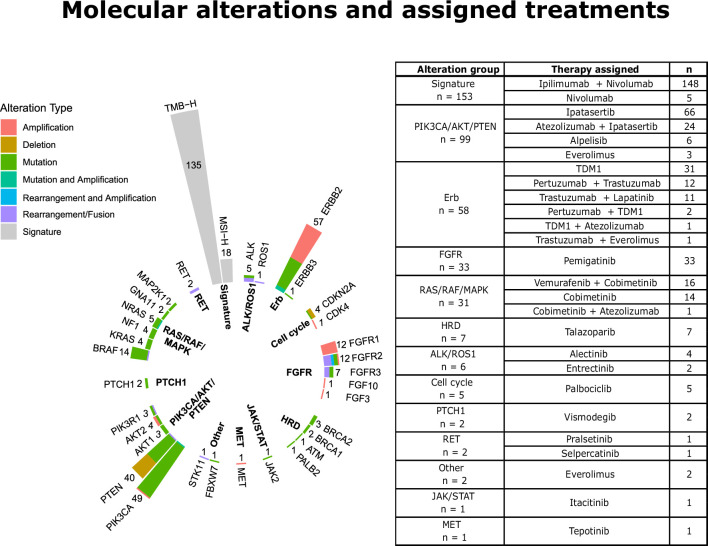
Molecular alterations and assigned treatments. Genomic alterations and tailored treatment proposed by MTB in ITT population. The figure summarizes genomic alterations identified and personalized treatment strategies recommended by the Molecular Tumor Board (MTB) for the intention-to-treat (ITT) population.

Actionable genomic alterations and MTB-selected treatment options for the intention-to-treat (ITT) population are reported in Extended Data Fig. 1.

**Fig. 1:**
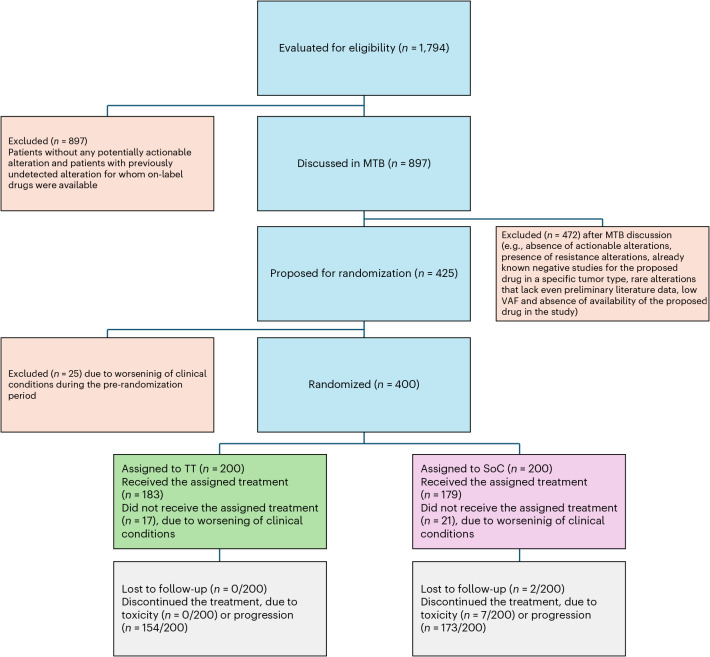
CONSORT diagram. CONSORT diagram for the ROME trial.

**Extended Data Fig. 2:**
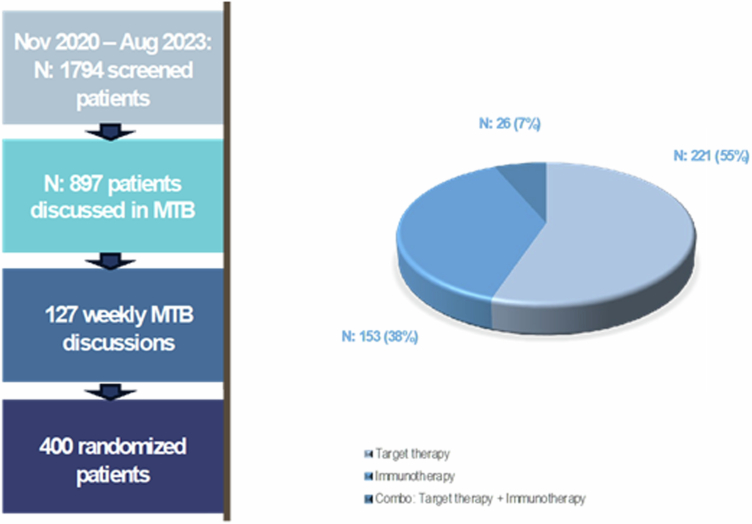
Molecular tumor board activity and treatment outcomes in the intention-to-treat (ITT) population. MTB outcomes. Molecular tumor board activity and treatment allocation in the intention-to-treat (ITT) population.

Following the MTB recommendations, 221 (55%) patients were referred to a targeted therapy, 153 (38%) to immunotherapy and 26 (7%) to a combination of both (Extended Data Fig. 2). Thus, 400 patients were ultimately randomized into the TT and SoC arms. Patients were allowed to crossover at the locally confirmed progression of the disease. The CONSORT diagram is presented in Fig. 1.

Patient characteristics and lines of prior therapy are detailed in Table 1 and Supplementary Table 1, respectively. The median age, baseline ECOG PS and number of previous lines of treatment were similar between arms. The TT arm had a higher proportion of women. The primary tumor origins were well balanced, with only glioblastomas and biliary tract tumors showing a slight overrepresentation in the SoC and TT arms, respectively, with no statistical differences observed. The TT arm also had a higher percentage of patients who received prior targeted therapy or hormonal therapy or a combination of chemotherapy and targeted therapy. In contrast, the SoC arm had more patients with prior immunotherapy.

**Table 1:** Characteristics of the ITT study population

Characteristics	Overall *n* = 400 (%)	SoC *n* = 200 (%)	TT *n* = 200 (%)
Age (years)			
Median (range)	61 (22–85)	60 (34–84)	62 (22–85)
Gender			
Male	192 (48.0)	100 (50.0)	92 (46.0)
Female	208 (52.0)	100 (50.0)	108 (54.0)
Ethnicity			
Caucasian	392 (98.1)	196 (98.0)	196 (98.0)
Asian	2 (0.5)	0 (0.0)	2 (1.0)
Hispanic	3 (0.8)	2 (1.0)	1 (0.5)
African American	1 (0.2)	1 (0.5)	0 (0.0)
Other	1 (0.2)	0 (0.0)	1 (0.5)
NA	1 (0.2)	1 (0.5)	0 (0.0)
Primary tumor			
Colorectal	65 (16.2)	31 (15.5)	34 (17.0)
Breast	40 (10.0)	20 (10.0)	20 (10.0)
Gastric	36 (9.0)	19 (9.5)	17 (8.5)
Glioblastoma	36 (9.0)	21 (10.5)	15 (7.5)
Biliary tract/gallbladder	35 (8.8)	10 (5.0)	25 (12.5)
NSCLC	35 (8.8)	18 (9.0)	17 (8.5)
Ovarian	18 (4.5)	8 (4.0)	10 (5.0)
Pancreatic	18 (4.5)	10 (5.0)	8 (4.0)
Melanoma	13 (3.2)	8 (4.0)	5 (2.5)
Anus	11 (2.8)	5 (2.5)	6 (3.0)
Others^a^	93 (23.2)	50 (25.0)	43 (21.5)
ECOG PS			
0	237 (59.3)	116 (58.0)	121 (60.5)
1	162 (40.4)	83 (41.5)	79 (39.5)
2	1 (0.3)	1 (0.5)	0 (0.0)
Previous treatment lines			
One	204 (51.0)	99 (50.0)	105 (52.0)
Two	196 (49.0)	101 (50.0)	95 (48.0)

Populations were well balanced after randomization. All variables were tested, and no significant difference was detected between treatment arms.

^a^The detailed description of this group is provided in Supplementary Table 4.

### Efficacy and survival outcomes

**Fig. 2:**
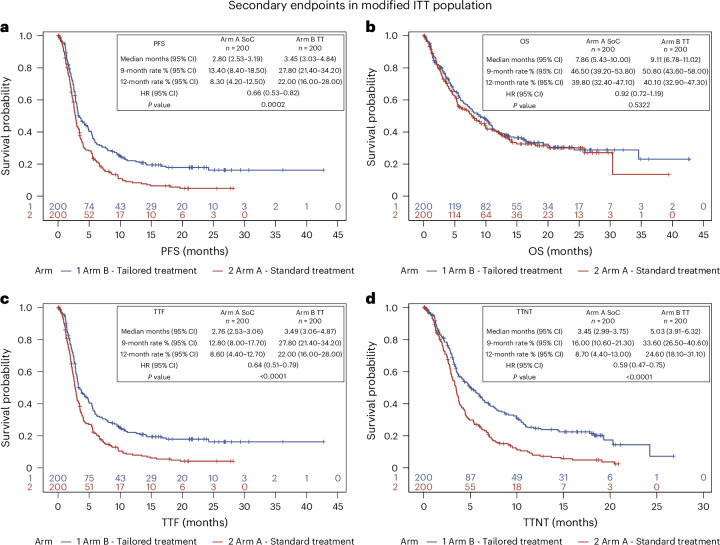
Secondary endpoints in the ITT population. Secondary survival endpoints in the ITT population. **a**, PFS (*P* = 0.002). **b**, OS (*P* = 0.5322). **c**, TTF (*P* < 0.0001). **d**, TTNT (*P* < 0.0001). Statistical test used to calculate *P* values: log-rank test.

The overall response rate (ORR) in the ITT population of 400 patients demonstrated a statistically significant improvement in the TT arm compared to the SoC arm. Among the 200 patients receiving TT, the ORR was 17.5% (95% confidence interval (CI): 12.5–23.5), which included 3% (six patients) achieving a complete response and 14.5% (29 patients) achieving a partial response. In contrast, the SoC arm achieved an ORR of 10% (95% CI: 6.2–15.0), with no complete responses and a 10% partial response rate (20 patients). Stable disease was observed in 19.5% (39 patients) in the TT arm and in 17.5% (35 patients) in the SoC arm, and progressive disease was reported in 63.0% and 72.5% of patients in the TT and SoC arms, respectively. The primary endpoint analysis conducted using the stratified analysis by Cochran–Mantel–Haenszel (CMH) test demonstrated a significant difference in ORR between groups (*P* = 0.0285; Extended Data Table 1). ORR varied across prespecified strata: in the breast cancer subgroup (Stratum A), ORR was 20.0% for TT versus 35.0% for SoC; in the non-colorectal gastrointestinal cohort (Stratum B), rates were 14.8% versus 9.1%; in non-small cell lung cancer (NSCLC) (Stratum C), 12.5% versus 6.7%; and in ‘Other malignancies’ (Stratum D), 19.1% versus 6.4%. The unique discrepancy with general results was observed in the breast cancer subgroup, probably as a consequence of the small breast cancer cohort and its inherent heterogeneity. The *P* value from the chi-square test (*P* = 0.0294; Extended Data Table 2) is nearly identical to the stratified CMH result, supporting the robustness of the main conclusion of the study. Furthermore, the Breslow–Day test for homogeneity of odds ratios across strata was not significant (*P* = 0.1231), failing to provide evidence of heterogeneity in the treatment effect across cancer types. PFS was significantly improved in the TT arm (median 3.5 months, 95% CI: 3.0–4.8) compared to the SoC arm (2.8 months, 95% CI: 2.5–3.2), with a hazard ratio (HR) of 0.66 (95% CI: 0.53–0.82, *P* = 0.0002; Fig. 2a). This benefit extended to key timepoints, showing substantial higher 9-month and 12-month PFS rates in the TT arm: 27.8% (95% CI: 21.4–34.2) at 9 months and 22.0% (95% CI: 16.0–28.0) at 12 months compared to 13.4% (95% CI: 8.4–18.5) and 8.3% (95% CI: 4.2–12.5), respectively, in the SoC arm. No significant difference in median overall survival (mOS) was observed between the TT and SoC arms (mOS: 9.1 months (TT) and 7.9 months (SoC); HR = 0.92, 95% CI: 0.72–1.19, *P* = 0.5322; Fig. 2b). A high crossover rate (59%) from SoC to TT occurred after disease progression. Among non-crossover patients in the SoC arm (*n* = 74), key reasons included rapid clinical deterioration (*n* = 33) and death before crossover eligibility (*n* = 25). Full details are reported in Supplementary Tables 2 and 3. Approximately half of the enrolled patients in both arms had already received two prior lines of systemic therapy, which may have influenced both treatment responsiveness and overall clinical outcome. Analyses of the key secondary endpoints—time to treatment failure (TTF) and time to next treatment (TTNT)—showed superior outcomes in the TT arm compared to the SoC arm. Median TTF was 3.5 months (95% CI: 3.1–4.9) in the TT arm versus 2.8 months (95% CI: 2.5–3.1) in the SoC arm (HR = 0.64, 95% CI: 0.51–0.79, *P* < 0.0001). At 12 months, TTF was 22.0% (95% CI: 16.0–28.0) in the TT arm and 8.6% (95% CI: 4.4–12.7) in the SoC arm (Fig. 2c). Patients receiving TT had a significantly longer TTNT than those in the SoC arm (median TTNT: 5.0 months (95% CI: 3.9–6.3) versus 3.5 months (95% CI: 3.0–3.8); HR = 0.59, 95% CI: 0.47–0.75). The TT arm also demonstrated a significantly higher proportion of patients remaining on treatment at 12 months (24.6%, 95% CI: 18.1–31.1) than the SoC arm (8.7%, 95% CI: 4.4–13.0) (Fig. 2d).

### Safety

**Fig. 3:**
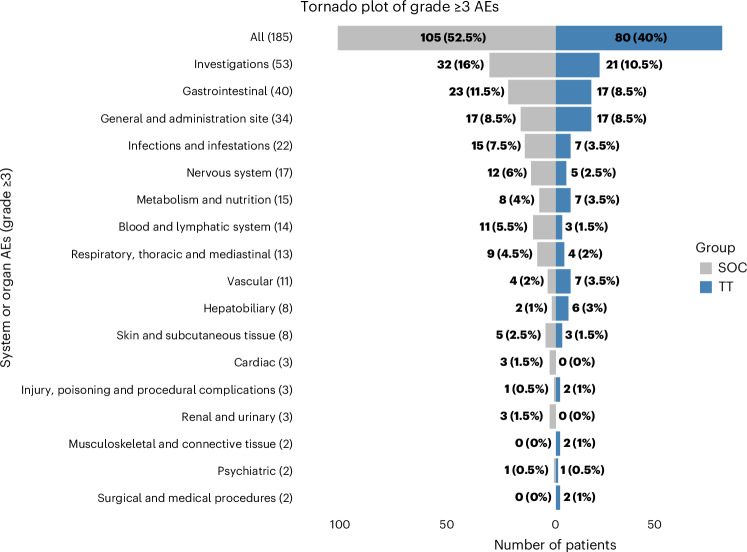
Tornado plot with the System Organ Class groups of grade ≥3 AEs according to CTCAE version 5. This figure shows the system or organ grade ≥3 AEs that occurred during the study for patients receiving SoC or TT.

**Extended Data Fig. 3:**
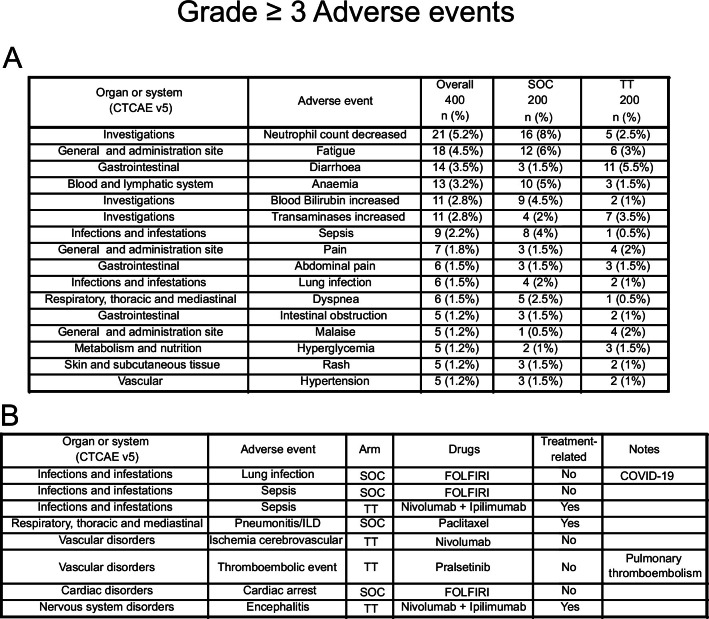
Grade ≥ 3 Adverse events. **a**) Grade ≥ 3 adverse events that occurred in at least 5 patients. **b**) Grade 5 adverse events. Each line reports a different event. SoC: standard of care, TT: tailored treatment; CTCAE: Common Terminology Criteria for Adverse Events.

The overall incidence of grade ≥3 adverse events (AEs) was similar in the TT and SoC arms (40.0% (80/200) and 52.5% (105/200), respectively). However, the specific AEs differed between the arms. In the TT arm, diarrhea was the most common AE (11 patients, 5.5%), whereas neutropenia was the most common AE in the SoC arm (16 patients, 8.0%) (Fig. 3 and Extended Data Fig. 3). Figure 3 presents a tornado plot illustrating these AEs, categorized by System Organ Class categories of the Common Terminology Criteria for Adverse Events (CTCAE) version 5. Extended data provide a comprehensive list of grade ≥3 AEs (Extended Data Fig. 3a) and all grade 5 AEs (Extended Data Fig. 3b).

### Exploratory subgroup analyses

**Fig. 4:**
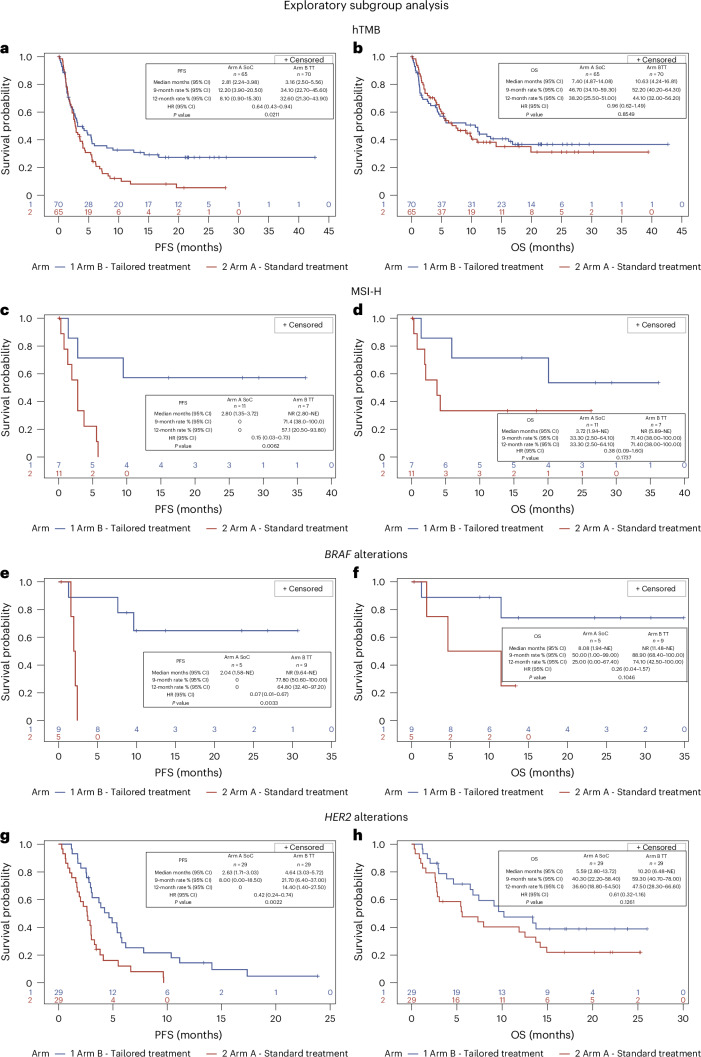
PFS and OS in exploratory subgroups. **a**, PFS in hTMB (*P* = 0.0211). **b**, OS in hTMB (*P* = 0.8549). **c**, MSI-H PFS (*P* = 0.0062). **d**, MSI-H OS (*P* = 0.1737). **e**, *BRAF* alterations PFS (*P* = 0.0033). **f**, *BRAF* alterations OS (*P* = 0.1046). **g**, *HER2* alterations PFS (*P* = 0.0022). **h**, *HER2* alterations OS (*P* = 0.1261). Statistical test used to calculate *P* values: log-rank test. *P* values derived from exploratory analyses should be interpreted with caution, as these analyses are hypothesis generating rather than confirmatory. Due to the non-predefined nature of exploratory endpoints and the potential for an inflated *α* level, the statistical significance observed in these analyses serves primarily to generate hypotheses for future confirmatory studies rather than providing definitive evidence NE, not evaluable; NR, not reached.

**Extended Data Fig. 4:**
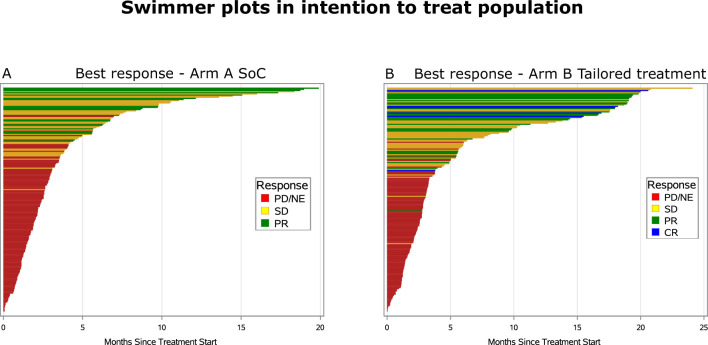
Swimmer plots for SoC and TT (ITT population). **a**) SOC; **b**) TT. TT: tailored treatment; SOC: standard of care; PD: Progressive disease; NE: Not evaluable; SD: Stable disease; PR: Partial response; CR: Complete response; ITT: intention-to-treat.

**Extended Data Fig. 5:**
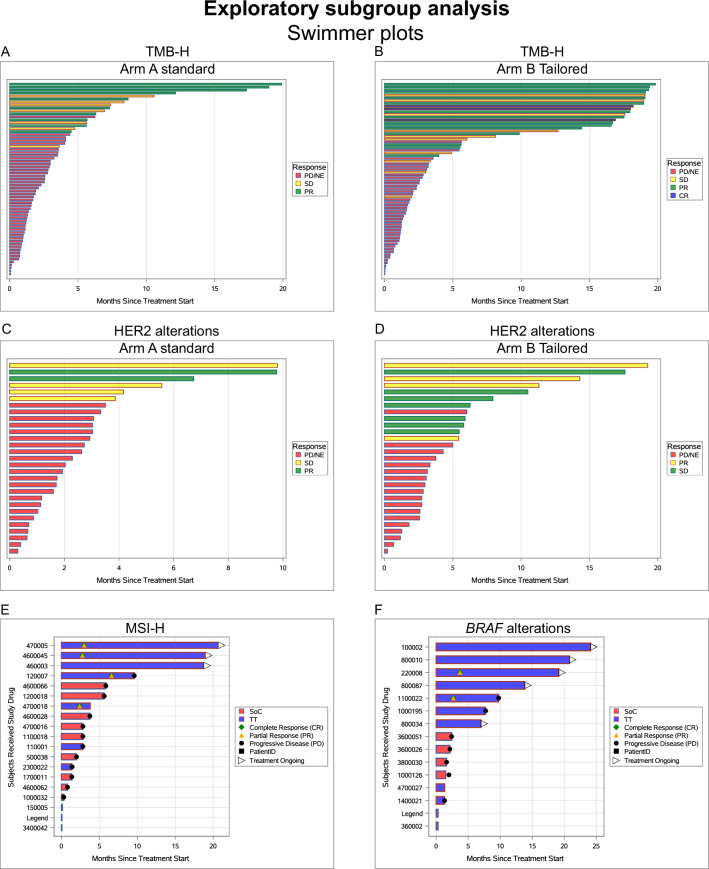
Swimmer plots for SoC and TT in exploratory hTMB, MSI, BRAF and HER2 subgroups. **a**) hTMB SOC; **b**) hTMB TT; **c**) HER2 alterations SOC; **d**) HER2 alterations TT; **e**) MSI-H; **F**) *BRAF* alterations. TT: tailored treatment; SOC: standard of care; PD: Progressive disease; NE: Not evaluable; SD: Stable disease; PR: Partial response; CR: Complete response; hTMB: High tumor mutational burden; MSI-H: microsatellite instability high.

Further exploratory analyses in specific subgroups provided additional insights. To assess the efficacy of TT against SoC, we analyzed subgroups defined by clinically relevant molecular alterations: MSI-H, hTMB, *BRAF* mutations and *ERBB2* (*HER2)* alterations (including amplifications and mutations). Within the ITT population, these alterations were detected with the following frequencies: hTMB/microsatellite stable (MSS) (≥10 mutations per megabase, 33.7%), MSI-H (4.5%), *BRAF* mutations (4.3%) and *ERBB2* alterations (14.3%). Treatment allocation was as follows: patients with hTMB, irrespective of microsatellite status, in the TT arm received immunotherapy (ipilimumab plus nivolumab or nivolumab monotherapy); patients with *BRAF* mutations received vemurafenib plus cobimetinib; and patients with *ERBB2* alterations received one of several anti-HER2 regimens (pertuzumab plus trastuzumab, TDM1, trastuzumab plus lapatinib, pertuzumab plus TDM1, TDM1 plus atezolizumab or trastuzumab plus everolimus). The results of these exploratory subgroup analyses of the ORR are presented in Extended Data Table 3; the swimmer plots for duration of response are reported in Extended Data Figs. 4 and 5; and PFS and OS are reported in Fig. 4.

Of the 135 patients with hTMB and MSS status (65 in the SoC arm and 70 in the TT arm), all patients in the TT arm received immunotherapy (ipilimumab plus nivolumab or, in a few cases, nivolumab monotherapy). The ORR was higher in the TT arm (25.7%, 95% CI: 16–37.6) than in the SoC arm (15.4%, 95% CI: 7.6–26.5) (Extended Data Table 3). PFS was significantly longer in the TT arm (3.2 months, 95% CI: 2.5–5.6) compared to the SoC arm (2.8 months, 95% CI: 2.2–4.0) (HR = 0.64, 95% CI: 0.43–0.94; Fig. 4a). At 12 months, the PFS rate was 32.6% (95% CI: 21.3–43.9) in the TT arm versus 8.1% (95% CI: 0.9–15.3) in the SoC arm, representing a 25% absolute improvement in patients alive and progression free at 1 year. OS was 10.6 months (95% CI: 4.2–16.8) in the TT arm and 7.4 months (95% CI: 4.9–14.1) in the SoC arm (HR = 0.96, 95% CI: 0.62–1.49; Fig. 4b).

Among 18 patients with MSI-H, the ORR was 57.1% (95% CI: 18.4–90.1) in the TT arm and 0% in the SoC arm (Extended Data Table 3). PFS had not been reached in the TT arm at the time of analysis, whereas it was 2.8 months (95% CI: 1.4–3.7) in the SoC arm. Furthermore, at 1 year, 57.1% (95% CI: 20.5–93.8) of patients in the TT arm remained on treatment compared to none in the SoC arm (HR = 0.15, 95% CI: 0.03–0.73; Fig. 4c). The mOS was not reached in the TT arm. However, at 12 months, the OS rate was 71.4% in the TT arm compared to 33.3% and an mOS of 3.7 months in the SoC arm (HR = 0.38; Fig. 4d).

Of the 14 randomized patients with *BRAF* alterations, five received SoC and nine received TT, consisting of a BRAF/MEK inhibitor combination. A notable difference in PFS was observed: PFS was not reached in the TT arm, whereas it was 2.0 months (95% CI: 1.6–not evaluable) in the SoC arm (HR = 0.07, 95% CI: 0.01–0.67; Fig. 4e). The ORR was 22.2% (95% CI: 2.8–60.0) in the TT arm compared to 0% in the SoC arm (Extended Data Table 3). Despite the small sample size, the 12-month PFS rate was substantially higher in the TT arm (64.8%, 95% CI: 32.4–97.2) compared to the SoC arm (0%). The TT arm also demonstrated superior OS compared to the SoC arm; mOS was not reached in the TT arm, whereas it was 8.1 months (95% CI: 1.9–not evaluable) in the SoC arm (HR = 0.26, 95% CI: 0.04–1.57; Fig. 4f).

In the subgroup of 58 patients with *HER2* alterations, various anti-HER2 agents were administered. One patient in this group had an *ERBB3* mutation, was also HER2 positive and received an indication for anti-HER2 treatment. The ORR was 13.8% (95% CI: 3.9–31.7) in the TT arm versus 6.9% (95% CI: 0.9–22.8) in the SoC arm (Extended Data Table 3). PFS was significantly longer in the TT arm (4.6 months, 95% CI: 3.0–5.7) compared to the SoC arm (2.6 months, 95% CI: 1.7–3.0) (HR = 0.4, 95% CI: 0.24–0.74; Fig. 4g). Furthermore, at 12 months, 14.4% (95% CI: 1.40–27.50) of patients in the TT arm remained progression free compared to no patients in the SoC arm. Patients with HER2-altered tumors receiving TT showed a trend toward higher OS compared to the SoC arm, with an mOS of 10.2 months (95% CI: 6.5–not evaluable) versus 5.6 months (95% CI: 2.8–13.7) (HR = 0.61, 95% CI: 0.32–1.16; Fig. 4h).

## Discussion

**Fig. 5:**
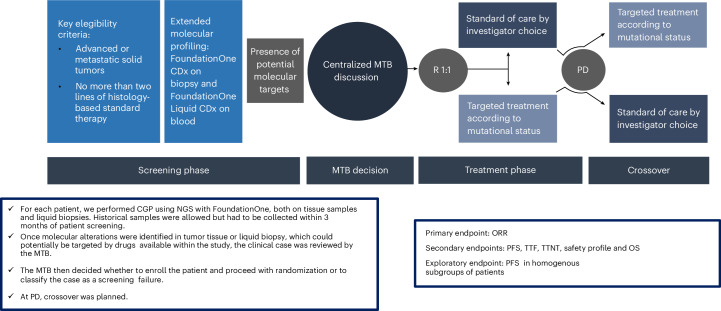
Schematic representation of the ROME trial design. The patient flow is shown from the left to right of the figure. The upper boxes represent the two main eligibility criteria, molecular profiling, MTB evaluation, randomization, treatment with TT or SoC and possible crossover at disease progression. The central blue boxes indicate the study phases. The lower boxes summarize the main study characteristics (left) and the study endpoints (right). CGP, comprehensive genomic profiling; PD, progressive disease; R, randomization.

The ROME trial provides evidence that TT, guided by comprehensive genomic profiling and expert MTB decision-making, significantly improves outcomes in pretreated patients with metastatic disease. The study demonstrated statistically significant improvements in the ORR and prolonged PFS compared to SoC, underscoring the clinical value of molecularly driven precision oncology. The improvement in ORR, the primary endpoint of the study, observed in the TT arm compared to the SoC arm within the ITT population (17.5% versus 10%), is clinically relevant and methodologically robust, although modest in magnitude. Notably, the TT arm achieved complete responses in a patient population with advanced metastatic disease treated in later lines, whereas no complete responses were observed in the SoC arm, underlining the potential efficacy of TTs in achieving deeper and potentially more durable clinical responses. The improvement in ORR was consistent despite variations across predefined cancer type strata. Subgroup analyses revealed pronounced benefit in the ‘Other malignancies’ stratum (19.1% versus 6.4%), positive trends in non-colorectal gastrointestinal (14.8% versus 9.1%) and NSCLC (12.5% versus 6.7%) subgroups and a lower ORR in the TT arm for breast cancer (20.0% versus 35.0%). However, the analyses of the differences in the odds ratios of response across subgroups have merely exploratory importance due to the limited size of most subgroups and, more importantly, because the primary aim of the trial was to assess the feasibility and potential global impact of a tailored therapeutic strategy based on comprehensive genomic profiling. The observed numerical imbalance across the four predefined cohorts resulted from a combination of protocol-driven and real-world factors. Although the initial design anticipated competitive enrollment with balanced strata, the trial’s biomarker-driven framework inherently favored variability in recruitment. A key factor contributing to this disparity was the differential prevalence of actionable genomic alterations across tumor types. Stratum D, which included diverse malignancies, exhibited higher rates of potentially actionable targets compared to other strata. Molecular profiling success rates further influenced recruitment dynamics. Tumors with lower shed DNA or suboptimal sample quality, common in certain histologies, reduced liquid biopsy utility, whereas tissue availability varied by cancer type. Additionally, site-specific expertise and patient populations probably played an important role, with centers specializing in rare cancers contributing more to Stratum D, whereas those focused on common tumors faced stricter exclusion thresholds. Temporal factors, including profiling turnaround times and drug availability after protocol amendments, also contributed to uneven accrual. Despite these imbalances, the trial’s statistical integrity remained robust, as previously discussed. The observed disparities reflect real-world heterogeneity in the distribution of actionable targets rather than methodological shortcomings. The ROME trial’s agnostic design, prioritizing molecular over histologic stratification, inherently accommodates such variability, validating its approach in capturing the complexity of precision oncology (Fig. 5). The recruitment pattern underscores a critical challenge in biomarker-driven trials: molecular prevalence and clinical exclusion criteria inevitably influence enrollment dynamics. The trial’s outcomes, however, demonstrate that robust statistical methods can mitigate imbalances, ensuring meaningful conclusions even in heterogeneous populations. These insights support the feasibility of tumor-agnostic frameworks while emphasizing the need for adaptive designs in future precision oncology studies. A major methodological departure from prior precision oncology trials is the timing of molecular profiling. In contrast to most studies that also use archival tissue samples from primary tumors, the ROME trial specifically employed metastatic tissue collected upon disease progression after the last therapy (with few exceptions; see supplementary materials). This strategy provided a more precise evaluation of the tumor’s mutational landscape at study entry. The addition of liquid biopsy analysis, comprehensive MTB assessment and early randomization (within the first two lines of therapy) further enhanced the accuracy of TT recommendations while mitigating the confounding effects of clonal heterogeneity frequently observed in later-stage disease.

A key finding from this study is the significant long-term benefit of PFS associated with TT. At the 12-month mark, patients receiving TT demonstrated improved disease control, indicating that it can extend PFS beyond 1 year for certain patients. This benefit, observed even in pretreated metastatic cases, highlights the clinical importance of personalized treatment strategies based on individual molecular alterations.

The observed significant improvements in ORR and PFS within the TT arm were not accompanied by a similar improvement in OS. The substantial crossover rate (58.8%) from the SoC arm to the TT arm after disease progression, although ethically appropriate, likely contributed to this lack of OS benefit. The high rate of non-crossover (41.2%) underscores the challenges of implementing precision therapies in late-line settings, where declining performance status and emergent complications often preclude treatment changes. This reality suggests that TTs may require earlier deployment to maximize clinical impact. This highlights the need for future studies to incorporate stratified crossover analyses to better understand the long-term effects of TTs on survival outcomes.

Subgroup analyses focused on specific molecular alterations (hTMB, *BRAF* mutations and *ERBB2* alterations) revealed considerable benefits. The hTMB subgroup demonstrated a particularly strong PFS benefit with immunotherapy (nivolumab with or without ipilimumab), showing efficacy across both MSI-H and MSS tumor types. This finding supports hTMB as a predictive biomarker for immunotherapy response, even within a biomarker-agnostic approach, particularly when using the TMB calculation algorithm employed by the Foundation One platform. Despite this promising conclusion, literature reports on TMB have been inconsistent, likely due to several factors. Key contributing elements include methodological variations, site-specific influences and a lack of standardization across the field. The complexity is further compounded by the use of different platforms and proprietary algorithms as well as variability in panel size, gene coverage, bioinformatics pipelines, sequencing depths and tumor purity. Additionally, cancer-type-specific variations play an important role in the assessment of TMB. Despite these challenges, the potential of hTMB as a predictive tool for immunotherapy response remains promising, highlighting the need for continued exploration and standardization efforts. Similarly, TTs showed efficacy against tumors harboring *BRAF* and *HER2* alterations. Although the smaller sample sizes in some subgroups may have limited the statistical power of certain observations, the overall findings emphasize the potential of precision oncology.

The similar incidence of grade 3/4 AEs in the TT and SoC arms, albeit with different AE profiles (diarrhea and fatigue in TT versus neutropenia in SoC), underscores the importance of proactive, personalized management of toxicities to optimize the therapeutic benefit–risk ratio.

The pivotal role of the MTB in shaping clinical decision-making was evident throughout the trial. Composed of a multidisciplinary team of specialists, the MTB enabled a mutation-agnostic and tumor-agnostic approach, tailoring treatment plans based on comprehensive molecular profiles rather than histology alone, avoiding the passive acceptance of the mutation/drug axiom. This approach, combined with the clinical data provided by referring clinicians, facilitated a patient-specific therapeutic strategy. In scenarios where multiple targeted treatment options were available for a single molecular pathway (such as PI3K/AKT or ERBB2 alterations), the MTB prioritized agents based on each patient’s unique molecular profile and clinical characteristics. Factors driving these decisions included the specific type of alteration (for example, *ERBB2* mutation versus amplification), published evidence on drug efficacy in specific contexts, prior therapeutic exposures and drug safety profiles. Notably, several agents (alpelisib, tepotinib, talazoparib, selpercatinib and pralsetinib) were introduced via protocol amendment in 2024. These updates, documented in Extended Data Table 4, preserved trial integrity through predefined statistical analysis plan (SAP) stratification while enhancing real-world relevance.

The MTB’s ability to address each patient’s biological complexity and clinical heterogeneity optimized therapy selection, contributing substantially to the positive outcomes of this trial. In case of detection of multiple actionable alterations, the MTB evaluated the potential clinical benefit of each therapeutic option, considering evidence from published literature, drug mechanism of action, patient-specific clinical factors, prior therapeutic responses and toxicity risks. The presence of multiple actionable biomarkers considerably complicates therapeutic decision-making due to pathway interactions, conflicting clinical evidence and diverse drug toxicities. Managing this complexity often exceeds individual clinician capacity and simple biomarker–drug algorithms. In the era of precision oncology, the MTB model is proving essential for interpreting complex genomic data and guiding personalized treatment plans that extend beyond traditional histopathological and direct target–drug association models, as seen in the prescription of TTs based on demonstrated efficacy in controlled clinical studies. The ROME trial provides, to our knowledge, the strongest evidence supporting MTB implementation in clinical practice, thanks to its agnostic and randomized design. Previous evidence suggested a potential improvement in survival outcomes when MTBs were implemented, as published by Miller et al.^31^ in a prospective, non-randomized phase 2 study. MTB utility is also being assessed in other prospective clinical trials, such as the Danish phase 2, non-randomized ProTarget study, implementing a Drug Rediscovery Protocol (DRUP)-like design^32^. Another example is the TARGET study (NCT04723316), a large nationwide prospective trial based in the United Kingdom designed for this purpose, aiming to enroll 6,000 patients with advanced cancers^33^. Our findings underscore the critical need to further develop MTBs and implement advanced decision support tools to effectively integrate and interpret complex multiomic data, ultimately enhancing the precision of clinical recommendations. The management of dual biomarkers (that is, hTMB and *HER2* alteration) posed unique challenges reflecting real-world precision oncology dilemmas, where pathway crosstalk (for example, MAPK activation dampening immunotherapy efficacy) and heterogeneous evidence bases complicate decisions. Our MTB addressed these scenarios by carefully evaluating multiple dimensions, including the biological relevance and actionability of each target (assessed using validated tools), variant allele frequency (VAF) of the detected alterations, quantitative thresholds for biomarkers such as TMB, strength of existing clinical evidence, potential interactions between targeted pathways, patient-specific clinical factors (including prior treatments, clinical status and comorbidities) and safety and toxicity profiles of candidate therapies, thus enabling structured and patient-centered decision-making. AI platforms can enhance MTB decisions in multitarget scenarios by structuring complex genomic/clinical data (for example, prioritizing therapies for HER2^+^/hTMB cases) and reducing cognitive overload through evidence-based recommendations. In Italy, the Alliance Against Cancer (ACC) consortium is advancing AI tools under initiatives such as CraNE/EUNetCCC to support MTBs in analyzing mutational profiles, aligning with European Union goals for precision oncology. These systems cross-reference real-world outcomes, biomarker–drug evidence and toxicity profiles, offering clinicians actionable hierarchies while maintaining human oversight. The work of the ACC exemplifies how AI augments, rather than replaces, expert deliberation, improving efficiency and consistency in biomarker-driven care.

The complexities of precision oncology necessitate adaptive platform trials to expedite patient access to effective TTs and keep pace with rapidly advancing genomic knowledge^34,35^. Platform trials, often referred to as multiarm, multistage studies, are randomized controlled trials (RCTs) that evaluate multiple treatments within different arms of a single trial, all compared to a shared control arm. This design incorporates adaptive features, allowing for the removal of ineffective treatment arms and the inclusion of new arms tailored to specific drug–target interactions. Additionally, patients may be reassigned to different treatment arms based on their response to prior therapies or changes in biomarker profiles. By replacing the traditional sequential trial model, this approach accelerates the process of drug discovery and development^36^. Furthermore, it will be beneficial to complement RCTs with observational studies to assess the generalizability and real-world applicability of their results. Future precision oncology will strongly benefit from dynamic national and international clinical trial databases, comprehensively annotated with gene-specific and variant-specific targets, directly linked to advanced decision support platforms to facilitate patient stratification into molecularly driven trials. Although the ROME trial provides robust evidence regarding the efficacy and safety of molecularly guided therapies in a controlled environment, the complexities of real-world practice—including patient heterogeneity, varied access to genomic testing and differences in healthcare infrastructure—may influence the outcomes observed. Observational studies using robust real-world data sources, such as population-based cancer registries, electronic health records and multicenter collaborative networks, combined with advanced AI analysis, would be instrumental in addressing these aspects. Future research priorities include optimizing biomarker-driven patient selection and MTB processes, developing innovative trial designs, investigating specific strategies to overcome resistance mechanisms and exploring combination approaches with immunotherapy and other tailored agents to propel the field of precision oncology further and realize its potential.

## Methods

### Inclusion and ethics

The study was approved by the institutional ethics committee of the coordinating center (Sapienza no. rif. C.E. 5575; February 2020) and by the ethics committee of each participating center. The investigational sites that approved the study protocol were as follows: AOU Policlinico Umberto I, Rome; European Institute of Oncology (IEO), IRCCS, Milan; Veneto Institute of Oncology IOV-IRCCS, Padua; Division of Medical Oncology, Pisa University Hospital; IRCCS Istituto Romagnolo per lo Studio dei Tumori (IRST) ‘Dino Amadori’, Meldola; Istituto Nazionale Tumori IRCCS Fondazione ‘G. Pascale’, Naples; IRCCS Istituto Nazionale Tumori Regina Elena (IRE), Rome; IRCCS Istituto di Candiolo, Candiolo; Medical Oncology Unit, ARNAS Garibaldi Catania; Azienda Ospedaliera Santa Maria di Terni, Terni; Pederzoli Hospital, Peschiera del Garda; IRCSS Ospedale Policlinico San Martino, Genoa; Ospedali Galliera, Genoa; Central Hospital of Belcolle, Viterbo; IRCCS Sacro Cuore Don Calabria Hospital, Negrar di Valpolicella; Santa Maria della Misericordia Hospital, Perugia; Nuovo Ospedale di Prato-Santo Stefano, Azienda USL Toscana Centro, Prato; CRO Aviano, National Cancer Institute, IRCCS, Aviano; Santa Maria delle Croci Hospital, AUSL Romagna, Ravenna; Oncology Unit ASST Papa Giovanni XXIII, Bergamo; Ordine Mauriziano Hospital, Turin; AOU Policlinico S. Andrea, Rome; Azienda Ospedaliera Universitaria Federico II, Napoli; Fondazione IRCCS Istituto Tumori di Milano; Fondazione Policlinico Universitario Campus Bio-Medico, Rome; A.O. Papardo-Messina; IRCCS Istituto Tumori ‘Giovanni Paolo II’, Bari; Policlinico universitario ‘Mater Domini’, Catanzaro; Centro Oncologico San Leopoldo Mandic, Isola Tiberina Gemelli isola; Rome; University Hospital, Ferrara; Misericordia Hospital, Grosseto; AUSL Piacenza Guglielmo da Saliceto Hospital, Piacenza; Comprehensive Cancer Centre, AUSL-IRCCS di Reggio Emilia, Reggio Emilia; Humanitas Gradenigo, Torino; Foundation IRCCS Casa Sollievo della Sofferenza, San Giovanni Rotondo; Ramazzini Hospital, Azienda Unità Sanitaria Locale Modena (AUSL), Carpi; AOUP ‘Paolo Giaccone’, Palermo; and AOU delle Marche, Ancona.

The competent authority, Agenzia Italiana del Farmaco (AIFA), authorized the trial on 8 July 2020 (AIFA/SC/P/76132). The trial is registered on ClinicalTrials.gov with identifier NCT04591431 and in the European Union Drug Regulating Authorities Clinical Trials Database (EudraCT) with number 2018-002190-21. The trial adhered to the principles of the Declaration of Helsinki regarding research involving human subjects. Forty-one centers received ethics approval and participated in the study enrollment. All patients signed the specifically conceived informed consent form (ICF).

### Study design, population and inclusion/exclusion criteria

This randomized, prospective, multicenter, proof-of-concept phase 2 clinical trial enrolled patients with inoperable advanced or metastatic solid tumors, regardless of histology. Eligible patients had failed at least one, but no more than two, prior lines of systemic therapy. An ECOG PS of 0 or 1 was required as well as being at least 18 years of age at the time of signing the ICF. All patients had to have measurable or evaluable disease defined per Response Evaluation Criteria in Solid Tumors version 1.1 (RECIST 1.1)^37^ or immune-related response criteria (irRC)^38^. If clinically indicated, magnetic resonance imaging (MRI) and positron emission tomography (PET) scans could have been performed. PET Response Criteria in Solid Tumors (PERCIST)^39^ were applied in cases where a baseline PET scan was available. Adequate renal, liver and bone marrow functions were required at baseline. Patients who were candidates for potentially curative surgery or other locoregional therapies were excluded. Those with well-established actionable molecular targets, not detected in previous molecular profiling, for which approved and marketed targeted therapies were available—such as *EGFR* mutations or *ALK* translocations in lung cancer, *BRAF* mutations in melanoma, *KIT* mutations in gastrointestinal stromal tumors, *ERBB2* amplification in breast cancer or tumor-agnostic biomarker-driven treatments already available in clinical practice—were also excluded. Furthermore, patients with only bone and/or brain metastases, uncontrolled brain disease (untreated and/or symptomatic) or those whose brain metastases had not been monitored for over 2 months were excluded. Patients with concurrent severe and/or uncontrolled medical conditions that could compromise their participation in the study (for example, uncontrolled diabetes, clinically relevant cardiac disease, uncontrolled hypertension, congestive heart failure, ventricular arrhythmias, active ischemic heart disease, myocardial infarction within the last year, chronic liver or renal disease, active gastrointestinal ulceration or severely impaired lung function) were also excluded (see supplementary materials for all inclusion/exclusion criteria).

For genomic testing, a tissue sample obtained during the screening phase or within 6 months before enrollment was required. The biopsy should be performed during the screening period, after the completion of conventional therapy for recurrent/metastatic cancer. Samples collected within 3 months before the patient’s ICF signature were acceptable, as were samples collected within 6 months, with prior acceptance by the MTB. Archived tissue samples were accepted for patients with glioblastomas and high-grade malignant gliomas. Patients with only one available biopsy, either liquid or solid, due to a failure of one method during the screening phase remained eligible for inclusion and discussion in the MTB. Conversely, if both tissue and liquid biopsy characterizations failed, patients were classified as screening failures.

Molecular profiling was performed using FoundationOne CDX and FoundationOne Liquid CDX tests on tissue and liquid biopsies. DNA from formalin-fixed, paraffin-embedded tumor tissue samples was isolated using the DNA extraction method and analyzed with the FoundationOne CDx panel to assess alterations across 324 genes, including substitutions, insertions and deletions (indels), copy number alterations (CNAs) and gene rearrangements. Genomic signatures of MSI and TMB were also evaluated. For FoundationOne Liquid CDx, circulating cell-free DNA (cfDNA) was extracted from plasma collected from anticoagulated peripheral whole blood samples using FoundationOne Liquid CDx cfDNA blood collection tubes. The cfDNA was analyzed for substitutions, indels in 311 genes, rearrangements in four genes and CNAs in three genes. Additionally, the test evaluated tumor fraction and genomic signatures such as blood-based tumor mutational burden (bTMB) and MSI-H status.

Upon identification of actionable alterations in either tumor tissue or liquid biopsy, and after MTB discussion, selected patients were assigned to receive immunotherapy, targeted therapy or both based on MTB recommendations. A crossover between the two treatment arms was planned upon progressive disease. The study design is reported in Fig. 5.

### MTB activities

An MTB convened weekly to review each eligible patient. The MTB consisted of medical oncologists, pathologists, geneticists, immunologists, bioinformaticians and other relevant specialists. Before the MTB discussion, the steering committee identified patients with potentially actionable genomic alterations. The MTB then reviewed each case, considering comprehensive molecular profiling data alongside the patient’s clinical features, such as performance status, comorbidities and concurrent treatments. The treating clinician presented each case selected by the steering committee, highlighting potential actionable therapeutic targets.

Molecular alterations were evaluated using databases such as ClinVAR, OncoKB and COSMIC and the ESMO ESCAT scale. The board employed VAF thresholds of 1% for tissue biopsies and 2% for liquid biopsies. It systematically reviewed potential intrinsic resistance mechanisms, such as *KRAS* mutations in patients considered for PIK3CA or AKT inhibitors. For patients with multiple actionable alterations (for example, hTMB alongside *HER2* amplification or *BRAF* mutation), the MTB prioritized therapies using a hierarchical framework prioritizing clinical urgency (for example, immunotherapy for MSI-H tumors regardless of co-alterations), followed by biomarker–drug evidence strength (for example, HER2-targeted agents over exploratory TMB-directed therapies when both were present) and, finally, considering toxicity overlap avoidance (for example, excluding PI3K inhibitors if preexisting hyperglycemia).

Patients discussed at the MTB who were not randomized were excluded for various reasons, including the absence of target alterations, the presence of resistance mutations, known negative studies related to a specific alteration, rare mutations without literature data, low VAF, unavailability of drugs for specific alterations, clinical considerations, high levels of uncertainty regarding potentially targetable alterations and the presence of established targetable alterations with available standard treatments. In cases suggesting a germline origin, germline testing was recommended.

**Extended Data Fig. 6:**
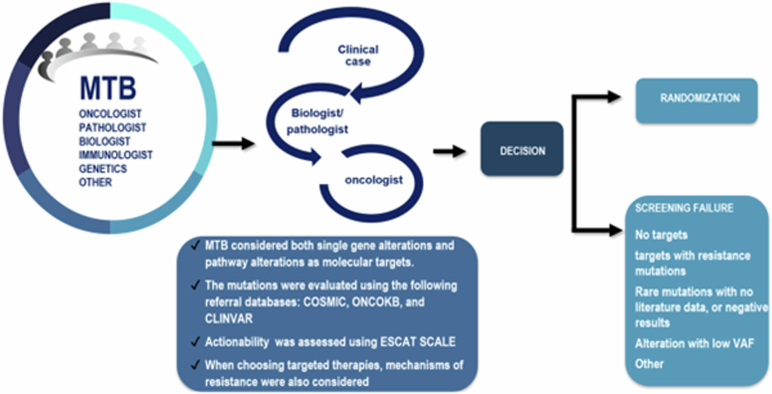
MTB structure and activities. The Molecular Tumor Board (MTB) ‘s organizational structure and core activities illustrate its workflow and multidisciplinary collaboration for precision oncology decision-making.

MTB recommendations included randomizing patients to receive TT, referring them to a geneticist in the presence of somatic alterations with potential germline significance, modifying SoC only when necessary and suggesting enrollment in clinical trials or early drug access programs, when available in Italy. MTB activities are summarized in Extended Data Fig. 6.

### Treatment regimen

Patients who met the inclusion and exclusion criteria and received a recommendation for a specific targeted agent or combination therapy from the MTB were randomized in a 1:1 ratio to receive either SoC or TT, according to their molecular profiles. In the SoC arm, patients were treated with standard chemotherapy, targeted therapy or immunotherapy based on primary tumor histology according to national guidelines and at the investigators’ discretion. The MTB also proposed possible modifications based on molecular profiling and expert opinion in the control group.

In the TT arm, patients received targeted therapy, immunotherapy or a combination that was selected according to their genomic profiles, as determined by the MTB. Combination therapies were proposed in cases where efficacy data for the combination were well established in different malignancies (for example, pertuzumab plus trastuzumab for HER2^+^ breast cancer and vemurafenib plus cobimetinib for *BRAF*-mutant melanoma) or when there was a biological rationale due to the presence of multiple actionable mutations. Such proposals were contingent upon the availability of published phase 1b/2 data supporting the combination in other tumor types. In cases involving immunotherapy, the combination of ipilimumab and nivolumab was usually preferred unless a patient’s clinical condition or comorbidities suggested otherwise. The decision was made collaboratively between the MTB and the patient’s treating clinician.

Patients were allowed to crossover to the TT arm upon disease progression. A substantial amendment to the protocol was implemented in October 2022, introducing additional treatments to the existing options, including entrectinib, alpelisib, pralsetinib and selpercatinib. Investigational therapies included erlotinib, pertuzumab, vemurafenib, trastuzumab emtansine, alectinib, vismodegib, cobimetinib, atezolizumab, trastuzumab, ipatasertib, entrectinib, everolimus, palbociclib, lapatinib, ipilimumab, nivolumab, brigatinib, ponatinib, itacitinib, pemigatinib, alpelisib, tepotinib, pralsetinib, talazoparib and selpercatinib. In cases where hTMB coexisted with a targetable mutation, a combination of immunotherapy and targeted therapy was generally preferred, provided that safety data were available. All treatments were administered according to the investigator’s brochure or the relevant regulatory clinical protocol. Each targeted agent was associated with a specific pathway, signature or gene alteration (Extended Data Table 4).

### Study endpoints

The primary endpoint of the study was the ORR, defined as the proportion of patients achieving a complete response or partial response, with respect to the total number of randomized patients. Secondary endpoints included PFS, defined as the time from randomization to disease progression or death, whichever occurred first; TTF, defined as the time from randomization until patient withdrawal for any reason, including disease progression or death; TTNT, defined as the time from randomization to the start of the next line of therapy; and OS, defined as the time from randomization to death from any cause. Additional planned secondary endpoints not reported in this paper include concordance between molecular profile on tumor tissue and ctDNA, quality of life measurements, immune fitness in the two treatment arms and the association between the molecular evaluation and gene expression profiling.

### Assessment of treatment outcomes and safety

Tumor response was locally assessed according to RECIST version 1.1 and irRC. In patients who discontinued treatment for reasons other than disease progression or consent withdrawal, tumor assessments continued until documented progression. Survival was monitored until death, loss to follow-up, consent withdrawal or study termination. AEs were graded using CTCAE version 5.0, with the frequency and severity of AEs documented for each patient.

### Data collection and trial oversight

Data were collected at the clinical sites and inserted into a web-based electronic case report form (eCRF) by investigators or designated personnel. The trial was overseen by an independent external data monitoring committee, which conducted periodic safety evaluations and reviewed efficacy during prespecified interim analyses. Per the study protocol, the data monitoring committee (DMC) conducted a prespecified interim analysis after 20% of the randomized patients (*n* = 76) had reached the final efficacy evaluation (day 308 or early termination), irrespective of treatment group assignment. The DMC reviewed these data and recommended proceeding with patient accrual as planned. As predefined, the stopping threshold for conditional power (CPt) was set at 5%. Comparisons were made between the observed TT group response rate at interim analysis and the projected end-of-study SoC group response rate (5%). At interim analysis, the TT group exhibited a 7.1% response rate. The calculated CPt for the projected SoC outcome, derived from the sample size assumptions, was 88.7%, demonstrating strong evidence to continue the trial and prompting a unanimous DMC recommendation to proceed. The authors confirm that the trial was conducted in compliance with the protocol, its amendments and the principles of Good Clinical Practice. All authors had access to the data used for manuscript preparation and contributed to its drafting, critical review or editing. The healthcare business of Merck KGaA, Darmstadt, Germany (CrossRef Funder ID: 10.13039/100009945), reviewed this manuscript for medical accuracy only before journal submission. The authors are fully responsible for the content of this paper, and the views and opinions described in the publication reflect solely those of the authors.

### Sample size

We hypothesized that TT would yield higher ORR compared to SoC, specifically a 20% ORR for TT versus 5% for SoC. According to the site of primary tumor, four cohorts were defined as breast cancer (Stratum A), non-colorectal gastrointestinal cancers (Stratum B), NSCLC (Stratum C) and ‘other malignancies’ (Stratum D), with competitive enrollment across these strata. To detect a 15% difference between the two arms, assuming an *α* of 0.10 and a *β* of 0.20 (80% power) and employing a one-sided chi-square test, a total of 86 patients (43 in each arm of the four cohorts) were required. The four cohorts were evaluated as separate strata, resulting in a combined cohort size of 344 patients (86 patients per stratum). The overall ORR was assessed using a two-sided CMH test at a 5% significance level; an unstratified sensitivity analysis was also performed. Considering a dropout rate of 10%, 384 patients were required. Assuming actionable mutations in at least 30% of eligible patients, the planned screening sample size was 1,280. During the course of the study, the screening failure rate was higher than initially anticipated, leading to a total of 1,794 screened patients. Due to the extended screening periods required for genomic testing and the inherent unpredictability of patient eligibility based on mutational test results, 12 additional patients undergoing screening at the time the target of 384 randomized participants was reached were permitted by the steering committee to enroll, resulting in a final total of 400 patients.

### Statistical analysis

All analyses were conducted using SAS software (v.9.4). The SAP, detailing the analyses to be executed, was finalized before database lock (SAP v.1.0). The randomization list was generated with a dedicated SAS program using the PROC PLAN procedure by an independent statistician. The randomization lists were incorporated into the eCRF system, which automatically managed the assignment of treatment arms. A soft lock of the database was performed on 31 July 2024. SAP v.2.0 was finalized on 30 January 2025, after the database freezing and data review meeting, and governs all analyses presented here. The full SAP is available upon reasonable request. Descriptive statistics were used to summarize the data, with mean, s.d., quartiles for continuous variables and frequency distributions for categorical variables. Differences in clinical characteristics were evaluated with *t*-test, binomial test, chi-quadro and CMH. The Breslow–Day test was used to evaluate the homogeneity of odds ratios. Box plots and tornado plots assessed the overall incidence of grade 3/4 AEs. Kaplan–Meier survival analysis, accompanied by the log-rank test, was employed to compare OS and PFS across arms and subgroups. To visualize clinical benefit duration (response and stable disease) across molecular subgroups—including high TMB and *PI3K/AKT*-altered and *ERBB2*-positive cohorts—swimmer plots were generated. HRs and 95% CIs were also calculated. Homogeneous subgroups of patients were evaluated in a prespecified exploratory analysis. Exploratory biomarker analyses, including assessments of hTMB (≥10 mutations per megabase), *BRAF* V600E mutations and *HER2* amplifications, were predefined in the SAP to evaluate treatment effect heterogeneity. These subgroups were selected based on emerging preclinical evidence during the trial’s enrollment phase, with steering committee approval for post hoc stratification. All statistical analyses were conducted on the ITT population. R v.4.3.3 and RStudio were used to build toxicity and genomic alterations plots.

### Reporting summary

Further information on research design is available in the Nature Portfolio Reporting Summary linked to this article.

## Online content

Any methods, additional references, Nature Portfolio reporting summaries, source data, extended data, supplementary information, acknowledgements, peer review information; details of author contributions and competing interests; and statements of data and code availability are available at 10.1038/s41591-025-03918-x.

## Supplementary information

Supplementary Information Supplementary Table 1: Type of previous treatments received by patients in the ITT population; Supplementary Table 2: Crossover rate; Supplementary Table 3: Reasons for patients not undergoing crossover (SoC arm); Supplementary Table 4: Extension of tumor primary site listed as ‘Other’ in Table 1; Protocol V4; Protocol Appendix 2; and CONSORT checklist.

Reporting Summary

## Figures and Tables

**Extended Data Table 1:**
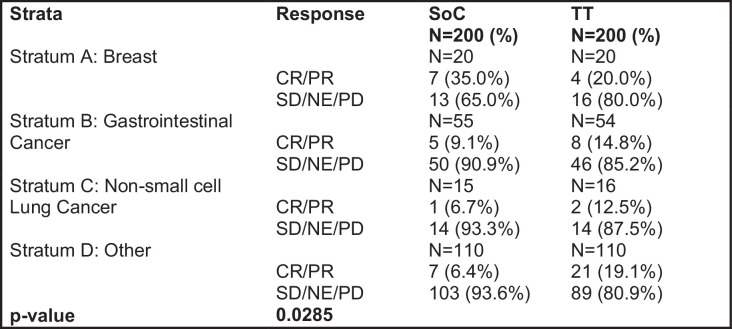
ORR in ITT population

ORR in the ITT population, summarizing the proportion of patients achieving a predefined clinical response to the treatment. CR, complete response; PD, progressive disease; PR, partial response; SD, stable disease.

**Extended Data Table 2:**
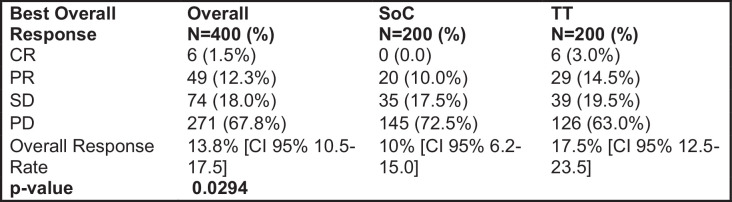
ORR as per strata

ORR analyzed according to stratified subgroups. CR, complete response; PD, progressive disease; PR, partial response; SD, stable disease.

**Extended Data Table 3:**
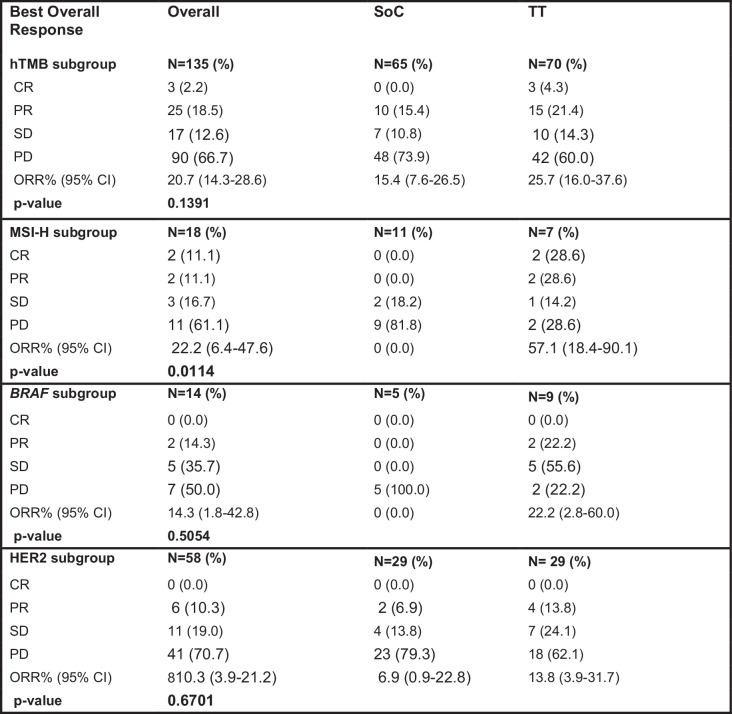
Treatment response in exploratory hTMB, MSI, BRAF and HER2 subgroups

Treatment response observed in exploratory subgroups, including hTMB, MSI, *BRAF* mutations and *HER2* alterations. CR, complete response; PD, progressive disease; PR, partial response; SD, stable disease.

**Extended Data Table 4:**
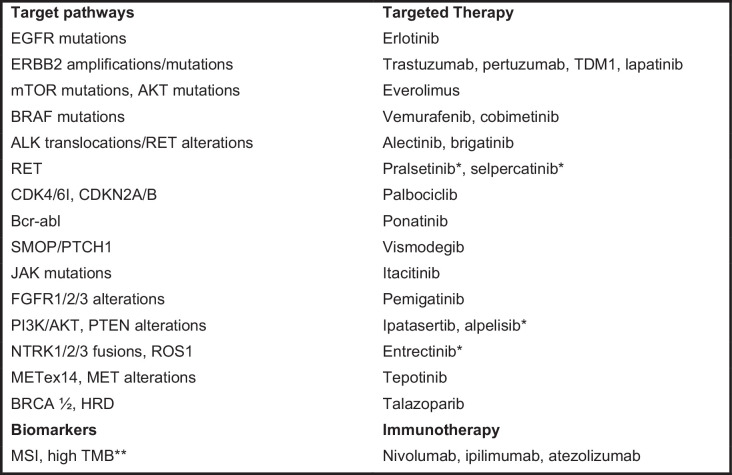
Available targeted treatments and pathways

Overview of available targeted treatments and their associated pathways.

* Drugs included in the V4 protocol amendment in October 2022.

** hTMB defined as ≥10 mutations per megabase in solid and/or liquid NGS.

## Data Availability

Individual deidentified participant data generated during the current study are available upon reasonable request from academic or qualified clinical researchers affiliated with recognized institutions, strictly for the purpose of conducting non-commercial, ethically approvable research aligned with the original scope of the trial. Applicants are required to submit a detailed research proposal, curriculum vitae and a declaration of non-conflict of interest. Requests must clearly describe the research objectives and methodology and must be reviewed and approved by the steering committee of the ROME trial during dedicated review sessions. Approval is granted based on scientific merit, data availability, intended data use and absence of overlapping research initiatives by the trial investigators. All approved requestors will be required to sign a data access agreement that restricts data use solely to the approved research project and prohibits any further distribution or use. Data will be shared via a secure data-sharing platform within 4–8 weeks of approval, contingent upon data volume and complexity. Data requests will be considered within 12 months of manuscript publication. The trial registration, study protocol and methodological details are publicly accessible through ClinicalTrials.gov (accession identifier NCT04591431; https://clinicaltrials.gov/ct2/show/NCT04591431). Additional publicly available datasets used in the analysis include the ClinVar database: freely accessible at https://www.ncbi.nlm.nih.gov/clinvar/; the OncoKB database: accessible at https://www.oncokb.org/; the COSMIC database: accessible at https://cancer.sanger.ac.uk/cosmic; and the ESMO ESCAT scale: accessible at https://www.esmo.org/guidelines/esmo-scale-for-clinical-actionability-of-molecular-targets-escat. No other public repositories or datasets requiring accession codes were used in this study.
