# Supplementary material for: Genomically matched therapy in advanced solid tumors: the randomized phase 2 ROME trial
Source: Nat Med. 2025 Sep 29;31(10):3514–23. doi: 10.1038/s41591-025-03918-x (PMC12532583; doi:10.1038/s41591-025-03918-x)
Supplement: Supplementary file 1 — Supplementary Table 1: Type of previous treatments received by patients in the ITT population; Supplementary Table 2: Crossover rate; Supplementary Table 3: Reasons for patients not undergoing crossover (SoC arm); Supplementary Table 4: Extension of tumor primary site listed as ‘Other’ in Table 1; Protocol V4; Protocol Appendix 2; and CONSORT checklist. [file 41591_2025_3918_MOESM1_ESM.pdf]

nature medicine

Supplementary information

https://doi.org/10.1038/s41591-025-03918-x

Genomically matched therapy in advanced solid tumors: the randomized phase 2 ROME trial

In the format provided by the authors and unedited

SUPPLEMENTARY MATERIALS

Suppl. Table 1: Type of previous treatments received by patients in the ITT population

Type of treatment	Overall<br>N=pts	SoC<br>N=pts	TT<br>N=pts
Chemotherapy	267	134	133
Immunotherapy	40	27	13
Chemo+immunotherapy	24	9	15
Chemo+target therapy	101	47	54
Hormone/target therapy	41	17	24
Radiotherapy*	13	7	6
ADC	1	1	0

Overview of prior treatments administered to the intention-to-treat (ITT) population, detailing therapeutic approaches and their distribution among patients. The column represents the number of patients who have received a specific treatment. A patient may have received one or two previous treatments, according to the study protocol. Pts= patients; SoC= standard of care; TT= tailored treatment.
*Only for unresectable primitive brain tumor. SoC: standard of care, TT: tailored treatment, ADC: antibody-drug conjugate.

Suppl. Table 2: Crossover rate

	SoC (%)	TT (%)	Total (%)
Patients progressed	180	154	334
Crossover	106 (58.8)	49 (31.8)	155 (46.4)

SoC: standard of care, TT: tailored treatment

Suppl. Table 3: reasons for patients not undergoing crossover (SoC arm)

No crossover reason	SoC (N=74)
AE	7*
Death	25
Worsening conditions or PD	33
Consent Withdraw	3
Lost to Follow up	3
Other	3**

*AE: 4 not related and 3 related to the treatment. ** other: 1 clinical decision; 2 data not avalilable.
PD: progression disease, AE: adverse events. SoC: standard of care, TT: tailored treatment.

Suppl. Table 4: Extension of Tumor Primary Site listed as "other" in Table 1. SCLC: small cell lung cancer; CNS: central nervous system; CUP: carcinoma of unknown primary; HCC: hepatocellular carcinoma; H&N: head and neck; RCC: renal cell carcinoma.

Tumor type	Count
Cervix	9
Neuroendocrine	9
Astrocytoma and other CNS	7
Sarcoma	7
Salivary gland	6
Small bowel	6
SCLC	5
Vater Ampulla	5
Thyroid	4
Urothelial	4
Endometrium	3
Oesophageal carcinoma	3
Penis	3
Skin squamous cell carcinoma	3
Adrenal	2
CUP	2
HCC	2
Meningioma	2
Prostate	2
Skin appendage tumor	2
H&N (unspecified)	1
Langherans cell histiocytosis	1
Mesothelioma	1
Oligodendroglioma	1
RCC	1

Urethra	1
Uterine carcinosarcoma	1
Total	93

Study Code (Sponsor): MAR-BAS-18-005

Eudract number: 2018-002190-21

PROTOCOL TITLE

The ROME trial from histology to target: the road to personalize target therapy and immunotherapy

(Final Version 4.0 – 24 May 2022)

No Profit Sponsor : Fondazione per la Medicina Personalizzata

Scientific Coordinator: Prof. Paolo MARCHETTI
"Sapienza - University of Rome"
Rome

Study Code: MAR-BAS-18-005
(FINAL Version 4.0 - 24.05.2022)

PROTOCOL APPROVAL PAGE

Name & Title: Prof. PAOLO MARCHETTI

Signature and Date: ______________________ 13/06/2022

Responsible/Protocol Author (dd/mm/yyyy)

Name & Title: Dott. MAURO BIFFONI

Signature and Date: ______________________ 13/6/2022

Protocol Co-Scientific Coordinator (dd/mm/yyyy)

Name & Title: Prof. PAOLO MARCHETTI

Signature and Date: ______________________ 13/06/2022

Scientific Coordinator (dd/mm/yyyy)

PROTOCOL APPROVAL PAGE BY PRINCIPAL INVESTIGATOR

I, the undersigned ____

(Name and Surname)

Principal Investigator at

CENTER ____

declare:

I have read the clinical trial Protocol:

STUDY CODE: MAR-BAS-18-005

EUDRACT NUMBER (if applicable): 2018-002190-21

TITLE

"The ROME trial from histology to target: the road to personalize target therapy and immunotherapy"

FINAL VERSION n.4.0 DATE 24/05/2022

I approve the above mentioned PROTOCOL

I commit to conduct the clinical trial as established with the Responsible/Protocol Author, Protocol Co-Scientific Coordinator and Coordinating Investigator

Signature and Date: ____________________________ ____/____/____

Investigator (dd/mm/yyyy)

LIST OF ABBREVIATIONS

AE	Adverse Event
AESI	Adverse Events of Special Interest
AIOM	Italian Association of Medical Oncology
ALK	Anaplastic lymphoma kinase
aPTT	activated Partial Thromboplastin Time
cfDNA	cell-free DNA
CI	Confidence Interval
CNAs	Copy Number Alterations
CNS	Central Nervous System
CSR	Clinical Study Report
CTCAE	Common Terminology Criteria for Adverse Events
CVAD	Central Venous Access Device
CT	Computed Tomography
CTCs	Circulating tumor cells
ctDNA	circulating tumor DNA
DMC	Data Monitoring Committee
DSUR	Development Safety Update Report
ERBB2	Receptor Tyrosine-Protein Kinase
EC	Ethics Committee
ECG	Electrocardiogram
ECHO	Echocardiogram
ECOG	Eastern Cooperative Oncology Group
EGFR	Epidermal Growth Factor Receptor
EORTC	European Organization for Research and Treatment of Cancer
ESF	Eligibility Screening Form
eCRF	electronic Case Report Form
FGFR	Fibroblast Growth Factor Receptor
FO	FoundationOne CDx
FOL	FondationOne Liquid CDx

FFPE	Formalin- fixed paraffin-embedded
GCP	Good Clinical Practice
GIST	Gastrointestinal Stromal Tumor
HER2	Human Epidermal growth factor Receptor 2
IB	Investigator’s Brochure
ICF	Informed Consent Form
ICH	International Conference on Harmonization
IMP	Investigational Medicinal Product
INR	International Normalized Ratio
IRB	Institutional Review Board
irRC	immune related Response Criteria
ISS	Istituto Superiore di Sanità
ITT	Intention-To-Treat
IWRS	Interactive Web-based Response System
JAK	Janus Kinase
LVEF	Left Ventricular Ejection Fraction
LMWH	Low Molecular Weight Heparin
MedDRA	Medical Dictionary for Regulatory Activities
MMR	Mis-Match Repair
MRI	Magnetic Resonance Imaging
MSI	Microsatellite-instability
MTB	Molecular Tumor Board
NCI-CTCAE	National Cancer Institute-Common Terminology Criteria for Adverse Events
NSCLC	Non-small-cell lung carcinoma
ORR	Overall Response Rate
OS	Overall Survival
PD	Progressive Disease
PD-L1	Programmed death-ligand 1
PERCIST	Positron Emission Tomography (PET) Response Criteria in Solid Tumors
PET	Positron Emission Tomography

PFS	Progression Free Survival
PP	Per Protocol
PPV	Positive Predictive Value
PROs	Patient-reported outcomes
QoL	Quality of Life
QP	Qualified Person
RET	REarranged during Transfection
RECIST	Response evaluation criteria in solid tumors
SD	Stable Disease
rSDV	remote Source Document Verification
SADRs	Serious Adverse Drug Reactions
SAE	Serious Adverse Event
SAP	Statistical Analysis Plan
SC	Steering Committee
SGOT	Serum Glutamic Oxaloacetic Transaminase
SGPT	Serum Glutamic Pyruvic Transaminase
SmPCs	Summary of Product Characteristics
SoC	Standard of Care
TMB	Tumor Mutational Burden
TT	Tailored Treatment
TTF	Time to Treatment Failure
TTNT	Time to Next Treatment
VEGF	Vascular Endothelial Growth Factor
VEGFR	Vascular Endothelial Growth Factor Receptor
WHO-ATC	World Health Organization-Anatomical Therapeutic Chemical Classification

SYNOPSIS

**Study Title:**	The ROME trial from histology to target: the road to personalize target therapy and immunotherapy
**Scientific Coordinator**	Paolo Marchetti
**Principal Investigator of the Coordinating Site**	Andrea Botticelli
**Internal Study code**	MAR-BAS-18-005
**Eudract no:**	2018-002190-21
**Version**	FINAL VERSION 3.0 (4 February 2022)
**Non Profit Promoter**	Fondazione per la Medicina Personalizzata
**Type of Study**	Randomized, prospective, multicenter, Proof of Concept, Phase II clinical trial
**Indication**	Patients with progressive disease (recurrent and/or metastatic) of breast cancer, metastatic gastro-intestinal tumors, non small cell lung cancer (NSCLC) or others. Patients should have completed or failed at least 1 line of treatment and no more than 2 as defined by the current version of the AIOM guidelines. Patients who are candidates to potentially curative surgery or other locoregional treatment are excluded
**Therapeutic Area**	Medical Oncology
**Background and Purpose**	Personalizing cancer medicine depends on the implementation of personalized diagnostics and therapeutics. Detailed genomic and gene expression signatures screening are likely to play a central role in this.[1-6]<br>Personalized Medicine has been widely depicted as a striking innovation, that is able to reform the standard approach to disease management, replacing the *one-size-fits-all* scheme of medicine with a *single-patient-sized* medical intervention.<br><br>Personalized medicine promoters usually highlight its potential to combine a more effective health-care with costs containment, according to the following rules:<br><br>● monitoring of disease risks and more effective prevention;<br>● early intervention;<br>● selection of optimal therapy;<br>● reduction of trial-and-error prescribing and reduction of adverse drug reactions;<br>● exclusion of unnecessary drugs;<br>● therapeutic drug monitoring and disease progression/remission monitoring;<br>● increased patient compliance with therapy.<br><br>In spite of expectations, many unsolved practical issues, from technical and scientific to ethical, legal and economic topics, are slowing down the translation of personalized medicine principles into medical practice. Furthermore, wide adoption of personalized strategies also has to deal with the peculiar rules, policy and reimbursement system of each country.<br><br>Application of Personalized Medicine in the real world seems entangled by the unmet need to develop evidence-based guidelines.<br><br>The benefits of personalized medicine in routine clinical practice have firstly emerged in oncology. The power of precision medicine in the field of anticancer therapy resides in the possibility to characterize the genomic profile of both the disease (eg somatic mutations in the *tumor tissue or blood sample*) and the

	patient (eg the *germinal genomic profile*). The first piece of information allows stratification of patients in responder and non-responder to specific drugs, improving efficacy and avoiding wasting of expensive medications as biological drugs [7-13]. Personalized medicine for cancer can be classified in:<br><br>- targeted therapy (which bloks the growth of cancer cells by interfering with specific molecular targets of cancer cells) or<br>- immunotherapy (which use the body'immune system to fight cancer cells by stimulating the immune system)<br><br>Targeted therapy belongs to Personalized Medicine approach and the study of genetic mutations on tumor tissue or blood sample (CTC or cfDNA) are changing the scenario of the treatment approach of cancer patients.<br><br>In clinical practice, the use of target therapies driven by mutation's assessment has radically changed the survival of patients affected by breast cancer, NSCLC, melanoma, colo-rectal cancer, while the clinical application of specific gene expression signatures is driving the choice of the best adjuvant strategy in early breast cancer.[14-20]<br><br>Despite the efficacy of such approach its use is restricted to a relatively small fraction of patients and the evaluation of mutation is conditioned by the primary site of the cancer, i.e. by the tumor histology. The current biological understanding leads to hypothesize that the cancer behavior is highly dependent from the underlying driver genetic alterations independently from the histology. It's widely demonstrated that such molecular alterations are detected regardless of the histology, and this has already modified the treatment approach of some cancers.<br><br>Furthermore, several studies have demonstrated the efficacy of the choice of treatment according to genomic evaluation regardless of its histology with acceptable cost-effectiveness profile. [21-32].<br><br>In the context of precision medicine the Immuno-oncology is becoming Precision Immuno-oncology and the efforts of science are directed towards the identification of predictive biomarkers of response to immune checkpoint inhibitors.<br>Promising biomarkers are Microsatellite Instability (MSI) and the tumour mutational load (TMB). In particular TMB is a quantitative biomarker that reflects the total number of mutations carried by tumor cells. TMB is well-known to reflect neoantigens burden potentially recognized by the immune system. This has been shown to correlate with better anti-PD-1 response in particular for both pembrolizumab and nivolumab combined with ipilimumab .<br>The same findings were demonstrated in the OAK study considering peripheral blood mutational load and response to atezolizumab. [33-35]<br>High tumor mutation burden (defined as tumors that have high ≥10 mutations/megabase, mut/mb) allows to identify 45% of patients who can benefit from immunotherapy regardless of PD-L1 expression. So, ever keeping in mind that although many evidences are available, the relationship between histology and genomic alterations is still under definition, as well as the relationship between the latter and gene expression.

	The aim of the present investigation is to combine all of the information available to drive the therapy selection according to the genomic alteration profile.<br><br>Therefore, the main objective of our study is to evaluate the efficacy of therapy according to genomic profile (TT – Tailored Treatment) versus Standard of Care (SoC).<br><br>A molecular profile of the cancer will be evaluated on tumor tissue biopsy (using the FoundationOne CDX (with updated gene panel 324 gene reflecting CDx) at the time of patient inclusion in the trial and on circulating DNA fragments (i.e. using FoundationOne Liquid CDX test) at the time of patient inclusion in the trial and at progression of disease.<br>Once identified molecular abnormalities on tumor tissue (not only those that are disease-specific), that can be modulated with target therapeutic intervention available within the present study, patients will be randomized to receive:<br>- Therapy at choice of physician, according to Standard of Care (SoC);<br>- Therapy according to genomic profile, Tailored Treatment (TT).
**Primary Objective**	The main objective of our study is to evaluate the efficacy (meant as overall response rate ORR) of TT vs SoC.
**Secondary Objectives**	To evaluate the long term survival (meant as progression free survival PFS) of TT vs SoC.
**Primary Endpoint**	Evaluation of the OVERALL RESPONSE RATE (ORR) of the Treatment at choice of physicians, according to Standard of Care (SoC) or of the Tailored Treatment (TT). The ORR will be constructed according to the specific design of the study, therefore including also the Rescue Therapy Phase data.<br>This means that the ORR will take into account 3 evaluations:<br>• The ORR estimation on the original final population (i.e 384 patients divided into the 4 groups of type of cancer)<br>• The ORR estimation done on the TT patients, which will include the original randomized TT patients and the patients switched from the standard of care therapy (SoC therapy) to the TT Therapy, this latter within the Rescue Therapy Phase (patients switching upon the first documented progression)<br>• The ORR estimation done on the population composed by the original TT patients, the original SoC patients and the switched TT patients. This means that the total population analyzed will include the original 384 population data (as per randomization) and the additional switched TT patients.
**Secondary Endpoints**	Evaluation of the:<br>• Progression Free Survival (PFS) of SoC vs TT<br>• Time to Treatment Failure (TTF) of SoC vs TT<br>• Time to Next Treatment (TTNT) of SoC vs TT<br>• Concordance between molecular profile on tumor tissue and ctDNA<br>• QoLs included in the two arms of the study of SoC vs TT<br>• The safety profile between the two treatment arms of SoC vs TT<br>• The immune fitness in the two treatment arms of SoC vs TT<br>• The association between the molecular evaluation and gene expression profiling<br>• Overall survival (OS) is defined as the time from randomization to death from any cause. Data for patients with no record of death will be censored at the last date they were known to be alive. The analysis of OS will follow the same methodology as the primary endpoint

**Inclusion Criteria**	1. Age ≥ 18 at time of signing Informed Consent Form<br>2. Patients able and willing to provide a written informed consent to participate to the study<br>3. Patients with recurrent/metastatic breast, gastrointestinal cancer, non small cell lung cancer or others<br>4. Patients not treatable with potentially curative surgery or other loco-regional treatments.<br>5. Patients should have been completed at least or failed the first line of treatment for breast cancer, gastro-intestinal, non small cell lung cancer or other cancer.<br>6. ECOG performance status from 0 to 1<br>7. Molecular target not actionable with approved drugs identified during screening by profiling with FoundationOne CDX on biopsy and FoundationOne Liquid CDx on blood<br>8. Biopsiable disease (tumor biopsy mandatory for tumor profiling). The biopsy must be performed during the screening period, when patients complete the conventional therapy for their recurrent/metastatic cancer. Historical samples will be considered for the study if collected within 3 months before the ICF signature of the patient. Samples older than 3 months, with a maximum timeframe of 6 months, and collected before progression of disease after the last treatment administered will be considered upon clinical judgement of the Investigator, after confirmation by the coordinating site or MTB. Samples obtained from a biospy of a metastatic lesion in progression after the last treatment administered represent the optimal tissue sample for genomic testing. Patients with glioblastomas and high grade malignant gliomas can be enrolled with the historical tissue samples<br>9. Measurable disease, eligible to standard treatment. Patients must have measurable or evaluable disease defined, per RECIST 1.1 or irCS (immune related Response Criteria), as at least one lesion that can be accurately measured in at least one dimension (longest diameter to be recorded for non-nodal lesions and short axis for nodal lesions) as ≥20 mm with conventional techniques or as ≥10 mm with spiral computed tomography (CT) scan, Magnetic Resonance Imaging (MRI), or a subcutaneous or superficial lesion that can be measured with calipers by clinical exam. For lymph nodes, the short axis must be ≥15 mm. Patients who have assessable disease by physical or radiographic examination but do not fully meet the above definitions of measurable disease (but still remains measurable) are eligible and will be considered to have evaluable disease. Patient's whose disease cannot be objectively measured by physical or radiographic examination (e.g., elevated serum tumor marker only) are NOT eligible. PET scan could be performed, if clinically indicated. For PET response evaluation PERCIST criteria will be applied.<br>10. Adequate renal function defined by a serum creatinine <1.5xUNL (upper normal limit).<br>11. Adequate liver function test defined by SGOT & SGPT <3xUNL (5xUNL in case of liver metastases), and bilirubin level <1.5xUNL<br>12. Adequate bone marrow function defined by platelets >100,000/mm3, hemoglobin >10 g/dL, and neutrophils >1,000/mm3<br>13. For female of child-bearing potential and for all women < 1 year after the onset of menopause: a negative pregnancy test <72 hours before starting study treatment is required. If sexually active, female of childbearing potential must use "highly effective" methods of

	contraception for the study duration. Contraception should continue after the last treatment for 3 months or for longer periods according to what reported in Appendix 1 of the protocol<br>14. For male of reproductive potential: any sexually active male patient must use a condom while on study treatment. Contraception should continue after the last treatment for 3 months or for longer periods according to what reported in the Appendix 1 of the protocol
**Exclusion Criteria**	1. Patients who have only bone and/or brain metastases<br>2. Patients treated with more than 2 lines for breast cancer, gastro-intestinal, non small cell lung cancer or other cancer<br>3. Patients with uncontrolled disease (untreated and/or sintomatic) and patients whose brain metastases have not been monitored for >2 months<br>4. Patients with well-established actionable targets for which approved and marketed targeted drugs are available (i.e. lung cancer with EGFR mutation, or ALK translocation, B-RAF mutant melanoma, GIST with KIT mutations or breast cancer with HER2 amplification and more)<br>5. Patient participating in another clinical trial with an experimental drug<br>6. Anticoagulation with anti-vitamin K (Low Molecular Weight Heparin [LMWH] is allowed)<br>7. Patients with other concurrent severe and/or uncontrolled medical disease which could compromise participation in the study, including uncontrolled diabetes, cardiac disease, uncontrolled hypertension, congestive cardiac failure, ventricular arrhythmias, active ischemic heart disease, myocardial infarction within one year, chronic liver or renal disease, active gastrointestinal tract ulceration, severely impaired lung function<br>8. Pregnant and/or breastfeeding women<br>9. Patients with any psychological, familial, sociological or geographical condition potentially hampering compliance with the study protocol and follow-up schedule<br>10. HIV, HBV, or HCV infection as per specific test performed at the screening visit or known as per Medical History<br>11. Patients with documented contraindication to any of the IMPs that will be used for the study, as reported in the respective SmPcs/IBs<br>12. Patients treated with the following drugs, because of the risk of immunosuppression: Chronic or high-dose oral corticosteroid therapy, TNF-inhibitors and Anti-T cell antibodies
**Main Parameters of Efficacy**	The primary endpoint is **Overall Response Rate (ORR)**.<br>ORR is defined as proportion of patients with reduction in tumor burden of a Conplete Response (CR) or Partial Response (PR)
**Main Parameters of Safety**	• Incidence and severity of AEs and SAEs<br>• Laboratory test abnormalities<br>• Vital Signs<br>• Physical Examination<br>• ECG and ECHO<br>• Concomitant Medications

<table>
<tr><td>Study Treatment</td><td>
Patients who are eligible according to the inclusion/exclusion criteria will be randomized in 2 treatment groups:
1) STANDARD OF CARE
Patients will be treated according the current version of the AIOM guidelines for their type of cancer. As an example, patients could be treated with standard chemotherapy and/or targeted therapy according to the histological results.
2) TAILORED THERAPY according to the genomic profile
Patients will be treated with target therapy and/or immunotherapy according to their genomic profile evidenced by the FO profiling.
Patients will be treated with one or more drugs of the following list, if a safe combination is avalaible in a phase II trial already conducted
<table>
<tr><th>TARGETED THERAPY</th><th>MOLECULAR TARGET</th></tr>
<tr><td>ERLOTINIB</td><td>EGFR mutation</td></tr>
<tr><td>TRASTUZUMAB, PERTUZUMAB, TDM1, LAPATINIB</td><td>ERBB2 amplifications/mut</td></tr>
<tr><td>EVEROLIMUS</td><td>mTOR mutations, AKT mut</td></tr>
<tr><td>VEMURAFENIB, COBIMETINIB</td><td>BRAFV600E mutations</td></tr>
<tr><td>ALECTINIB, BRIGATINIB</td><td>ALK, RET</td></tr>
<tr><td>PALBOCICLIB</td><td>CDK4/6, CDKN2A/p16</td></tr>
<tr><td>PONATINIB</td><td>Bcr-abl</td></tr>
<tr><td>VISMODEGIB</td><td>SMO/PTCH1</td></tr>
<tr><td>ITACITINIB</td><td>JAK mutation</td></tr>
<tr><td>INCB054828</td><td>FGFR1/2/3</td></tr>
<tr><td>IPATASERTIB</td><td>PI3K, AKT, PTEN</td></tr>
<tr><td>ENTRECTINIB</td><td>ROS1</td></tr>
<tr><td>ALPELISIB</td><td>PI3K, AKT</td></tr>
<tr><td>TEPOTINIB</td><td>MET amplification/exon14 skipping mutations (*)</td></tr>
<tr><td>PRALSETINIB</td><td>RET</td></tr>
<tr><td>TALAZOPARIB</td><td>BRCA1/2, ATM, other HRD status</td></tr>
<tr><td>SELPERCATINIB</td><td>RET</td></tr>
<tr><td>IMMUNOTHERAPY</td><td>BIOMARKERS</td></tr>
<tr><td>ATEZOLIZUMAB, NIVOLUMAB, IPILIMUMAB</td><td>MSI, HIGH TUMOR MUTATIONAL BURDEN, OTHER</td></tr>
</table>
Note: comma (,) means single drugs which can be used for that target.
(*) as primary oncogenic driver
Drugs will be administered according to their respective SmPCs (or IBs in case of drugs under development). More information about each IMPs as well as recommendation for toxicity management are given in the protocol, Appendix 2
</td></tr>
<tr><td>Study Procedures</td><td>Study Design:</td></tr>
</table>

	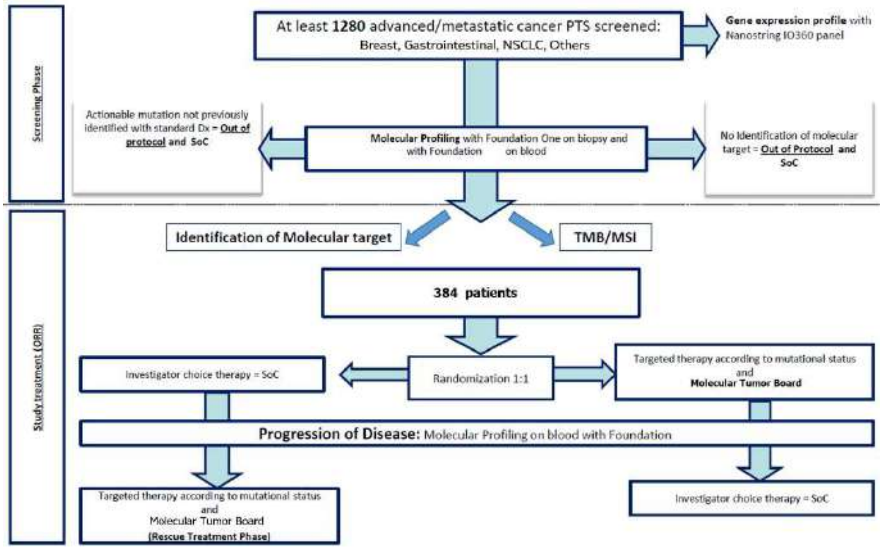<br><br>Prospective, 2 arms:<br>1. Therapy at choice of physician, according to Standard of Care (SoC)<br>2. Tailored treatment according to genomic profile (Tailored Treatment, TT)<br>After FO evaluations patients with actionable molecular alterations, not previously identified with other methods, for which approved drugs according to histotype are available, will be excluded.<br>All the study drugs listed in the above table (which are the ones that will be used within the present study) will be administered according to the respective SmPCs and /or Investigator's Brochure data (IB) and Appendix 2 of the present protocol. Any different combination will be reported as a protocol violation and managed according to GCPs requirements. Details on how to document and evaluate such cases will be reported in the study specific Safety Management Plan.<br><br>Patients will be qualified for the study according to the results of the analysis performed on tissue samples collected and analyzed with FoundationOne CDX at screening. Historical samples will be considered for the study if collected within 3 months from the ICF signature of the patient. Samples older than 3 months, with a maximum timeframe of 6 months, will be considered upon clinical judgement of the Investigator.<br>In any case, the analysis of the historical samples must be conducted with Foundation One service for the purposes of the study. In case of referral patients, if a tumor tissue sample analyses performed according to the Foundation One service technic will be provided as part of the medical history, it will not be needed to repeat neither the biopsy nor the relevant determination.<br>To be noticed that the diagnosis may be made on the tissue biopsy of a metastatic site if primary tumor is not available.<br>Patient-reported outcomes (PROs) will be used to evaluate physical, mental and social health status of patients (PRO questionnaires are used to assess symptoms and functions, and to enhance communication between clinicians and patients): EORTC QLQ-C30 will be used to evaluate the quality of life of cancer patients.
**Molecular Profile**	Genomic evaluation will be performed using the FoundationOne CDX (with updated gene panel 324 gene reflecting CDx) service and FoundationOne Liquid CDx service. FoundationOne is a comprehensive genomic profile that applies next

	generation sequencing in a unique manner to identify all 4 types of genomic alterations across all genes known to be unambiguous drivers of solid tumors with high accuracy. The test simultaneously sequences the coding region of 324 cancer-related genes plus introns from 28 genes often rearranged or altered in cancer to a typical median depth of coverage of greater than 500X. Each covered read represents a unique DNA fragment to enable the highly sensitive and specific detection of genomic alterations that occur at low frequencies due to tumor heterogeneity, low tumor purity and small tissue samples. FoundationOne detects all classes of genomic alterations, including base substitutions, insertions and deletions (indels), copy number alterations (CNAs) and rearrangements using a small, routine FFPE sample (including core or fine needle biopsies). FoundationOne ®Liquid CDx is Foundation Medicine's best-in-class, blood-based circulating tumor DNA assay, designed to interrogate the most druggable cancer-related genes. FoundationOne Liquid CDx was rigorously validated to identify all four classes of alterations, achieving the superior performance you can expect from Foundation Medicine. FoundationOne ®Liquid CDx has been updated to interrogate the same number of cancer-related genes as the FoundationOne® CDx, increasing diagnostic capabilities and increasing its clinical usefulness.<br>If a relevant alteration is found in any one of the genes on the current gene list, the report will identify the gene and alteration and will provide an interpretation that is specific to the patient's tumor.

Current Gene List[2]

Genes with full coding exonic regions included in FoundationOne®CDx for the detection of substitutions, insertion-deletions (indels), and copy-number alterations (CNAs).

ABL1	ACVR1B	AKT1	AKT2	AKT3	ALK	ALOX12B	AMER1 (FAM123B)	APC
AR	ARAF	ARFRP1	ARID1A	ASXL1	ATM	ATR	ATRX	AURKA
AURKB	AXIN1	AXL	BAP1	BARD1	BCL2	BCL2L1	BCL2L2	BCL6
BCOR	BCORL1	BRAF	BRCA1	BRCA2	BRD4	BRIP1	BTG1	BTG2
BTK	C11orf30 (EMSY)	CALR	CARD11	CASP8	CBFB	CBL	CCND1	CCND2
CCND3	CCNE1	CD22	CD274 (PD-L1)	CD70	CD79A	CD79B	CDC73	CDH1
CDK12	CDK4	CDK6	CDK8	CDKN1A	CDKN1B	CDKN2A	CDKN2B	CDKN2C
CEBPA	CHEK1	CHEK2	CIC	CREBBP	CRKL	CSF1R	CSF3R	CTCF
CTNNA1	CTNNB1	CUL3	CUL4A	CXCR4	CYP17A1	DAXX	DDR1	DDR2
DIS3	DNMT3A	DOT1L	EED	EGFR	EP300	EPHA3	EPHB1	EPHB4
ERBB2	ERBB3	ERBB4	ERCC4	ERG	ERRFI1	ESR1	EZH2	FAM46C
FANCA	FANCC	FANCG	FANCL	FAS	FBXW7	FGF10	FGF12	FGF14
FGF19	FGF23	FGF3	FGF4	FGF6	FGFR1	FGFR2	FGFR3	FGFR4
FH	FLCN	FLT1	FLT3	FOXL2	FUBP1	GABRA6	GATA3	GATA4
GATA6	GID4 (C17orf39)	GNA11	GNA13	GNAQ	GNAS	GRM3	GSK3B	H3F3A
HDAC1	HGF	HNF1A	HRAS	HSD3B1	ID3	IDH1	IDH2	IGF1R
IKBKE	IKZF1	INPP4B	IRF2	IRF4	IRS2	JAK1	JAK2	JAK3
JUN	KDM5A	KDM5C	KDM6A	KDR	KEAP1	KEL	KIT	KLHL6
KMT2A (MLL)	KMT2D (MLL2)	KRAS	LTK	LYN	MAF	MAP2K1 (MEK1)	MAP2K2 (MEK2)	MAP2K4
MAP3K1	MAP3K13	MAPK1	MCL1	MDM2	MDM4	MED12	MEF2B	MEN1
MERTK	MET	MITF	MKNK1	MLH1	MPL	MRE11A	MSH2	MSH3
MSH6	MST1R	MTAP	MTOR	MUTYH	MYC	MYCL (MYCL1)	MYCN	MYD88
NBN	NF1	NF2	NFE2L2	NFKBIA	NKX2-1	NOTCH1	NOTCH2	NOTCH3
NPM1	NRAS	NT5C2	NTRK1	NTRK2	NTRK3	P2RY8	PALB2	PARK2
PARP1	PARP2	PARP3	PAX5	PBRM1	PDCD1 (PD-1)	PDCD1LG2 (PD-L2)		PDGFRA
PDGFRB	PDK1	PIK3C2B	PIK3C2G	PIK3CA	PIK3CB	PIK3R1	PIM1	PMS2
POLD1	POLE	PPARG	PPP2R1A	PPP2R2A	PRDM1	PRKAR1A	PRKCI	PTCH1
PTEN	PTPN11	PTPRO	QKI	RAC1	RAD21	RAD51	RAD51B	RAD51C
RAD51D	RAD52	RAD54L	RAF1	RARA	RB1	RBM10	REL	RET
RICTOR	RNF43	ROS1	RPTOR	SDHA	SDHB	SDHC	SDHD	SETD2
SF3B1	SGK1	SMAD2	SMAD4	SMARCA4	SMARCB1	SMO	SNCAIP	SOCS1
SOX2	SOX9	SPEN	SPOP	SRC	STAG2	STAT3	STK11	SUFU
SYK	TBX3	TEK	TET2	TGFBR2	TIPARP	TNFAIP3	TNFRSF14	TP53
TSC1	TSC2	TYRO3	U2AF1	VEGFA	VHL	WHSC1 (MMSET)	WHSC1L1	WT1
XPO1	XRCC2	ZNF217	ZNF703					

Select Rearrangements[2,3]

Genes with select intronic regions for the detection of gene rearrangements, one gene with a promoter region and one non-coding RNA gene.

ALK	BCL2	BCR	BRAF	BRCA1	BRCA2	CD74	EGFR	ETV4
ETV5	ETV6	EWSR1	EZR	FGFR1	FGFR2	FGFR3	KIT	KMT2A (MLL)
MSH2	MYB	MYC	NOTCH2	NTRK1	NTRK2	NUTM1	PDGFRA	RAF1
RARA	RET	ROS1	RSPO2	SDC4	SLC34A2	TERC*	TERT (promoter only)**	
TMPRSS2								

*TERC is non-coding RNA gene.
**TERT is gene with promoter region.

Reports will include also tumor mutational burden (TMB) and microsatellite instability (MSI) status—biomarkers that may help predict response to checkpoint inhibitors.

**GENE EXPRESSION**	All screened patients (expected n = 1280) will have tissue samples profiled using the Nanostring IO360 panel.
**Statistical Considerations**	**RANDOMIZATION**<br>The randomization list, in a 1:1 ratio to one of two treatment arms, will be prepared by an independent Statistician who will maintain it during the whole course of the study. The list will be centralized and managed by an Interactive Web-based Response System (IWRS) implemented as a function in the eCRF.<br>A complete block randomization scheme will be applied to achieve balance in treatment assignment within each of the 4 strata, as defined by cancer type group.<br><br>**DATA MANAGEMENT**<br>The data collection will be carried out at site. The data will be entered by the Investigator or a designee in a web-based Database (eCRF).<br><br>**SAMPLE SIZE**

	Four independent experiments will be carried out within each considered cancer-site. We hypothesize that therapy based on molecular profile (TT) would result in a high overall response rate (ORR) with respect to SoC, i.e. 5% ORR of SoC in favor of 20% of TT. On the basis of that hypothesis, assuming α = 0.10 and β = 0.20 (i.e. 80% of power), and conducting a one-sided Chi-square test, 86 patients (43 patients in each arm of the four cohorts) are sufficient for detecting a 15% difference between the two arms (i.e. SoC = 5% versus TT = 20%), in terms of number of responders. According to the nature of the study (i.e. basket study), in order to evaluate the ORR on the whole sample, the four cohorts will be also considered as strata of a single cohort of 344 patients (86 patients in each strata).The ORR will be tested conducting a two sides Cochran–Mantel–Haenszel test at 5% significant level. A sensitivity analysis will also be carried out not considering the strata (i.e. Chi-square test).<br>In order to account for the possible influence of not evaluable patients and to safe sufficient power for the analyses of the single stratum, an additional 10% of patients will be considered. Therefore, a total of 384 patients will be enrolled.<br>The cited four cohorts will be competitive with each other.<br>Assuming that in at least 30% of eligible patients the actionable mutations will be observed, the total number of patients to be screened is expected to be around 1280.<br><br>**INTERIM ANAYSIS**<br>An Interim Analysis will be carried-out when 20% of the randomized total patients (i.e. 76 patients), regardless of which of the four groups they belong to, have reached the final efficacy evaluation (Day 308 or early termination). As soon as the 76th patient has reached the final efficacy evaluation (including the patients who early terminated the study, if any, so that the total number of patients to be analyzed in the Interim Analysis could be greater than 76) the pre-specified analysis will be carried-out in the ITT population by an Independent Statistician. The results will be given to the Data Monitoring Committee (DMC) in terms of "go/no go" data, who will review them and provide the relevant recommendations according to pre-specified rules (please refer to section 10.5.1). There will be no interruption in the patients enrolment during the interim analysis activities.<br>The method for performing this analysis will be based on the following papers: Lan et al (1982)[47], Wang et al (2002)[48] and Siu and Lan (2001)[49].<br>The interim analysis will be based on the computation of the so called Conditional Power (CP). The CPt is the probability, given the data observed at the time t of the interim analysis that are statistically significant at the level α (0.05 for the present studies) for the two-sided test verifying H0 versus H1, at the end of the study. Unknown parameters will be estimated with the observed data and under the H0 model.<br><br>The main assumptions are the following:<br>Nt=76 patients (where t = time of the interim analysis);<br>CPt= conditional power at time t;<br>At = threshold for CPt under which the trial will be stopped. That threshold is fixed on the expected result of the SoC group, i.e. 0.05 (5% of Responder patients).<br><br>The decision rules to be adopted at time t in function of the CPt value will be the following:<br>• If CPt<At, then stop the studies (there is a very strong evidence of futility);

	• otherwise, if CPt≥At, the studies will continue under the initial conditions.<br><br>The interim analysis is intended to perform intermediate evaluation of efficacy data in order to assess the need to early terminate the study because of futility (i.e. very poor chance of success), avoiding possible unethical exposure of patients to undue treatment.<br><br>The use of the above described procedure does not inflate the nominal significance level of the study. If the study is early terminated for futility the alpha level is deflated. As a consequence, no adjustment of the significance level of the test is requested.<br><br>A more detailed document to describe the above procedure will be prepared and provided to the Data Monitoring Committee before performing the assessment (DMC charter).<br><br>**STATISTICAL ANALYSIS**<br>The analyses will be performed with SAS version 9.4. On the basis of what described above, all the analyses to be carried out will be detailed in the Statistical Analysis Plan (SAP) which will be finalized in Version 1.0 before the Data Base freezing. Version 2.0 of the SAP will be prepared after the Data Review Meeting (in which the protocol violations will be evaluated) and will include the list of the patients belonging to the defined populations and the randomization list.<br>All the variables will be descriptively analyzed by treatment and visit (mean, median, standard deviation, interquartile range for continuous variables, frequency distribution for categorical variables). Efficacy analysis will be applied in both the ITT and PP populations. Results from the ITT population will be considered the primary ones.
**Planned Sample Size**	1280 patients
**Total Number of Centers**	About 50 oncologic centers in Italy
**Recruitment Duration**	24 months
**Patient Involvement In The Study**	18 months according to the mean time expected for progression of disease and taking into account the rescue treatment phase.
**Total Study Duration**	46 months up to the Clinical Study Report completion
**Data Management**	The data cleaning will be performed by CMV-Stat Data Management team who will provide the Investigator with the list of the detected inconsistencies on each group of data analyzed. The validation of the inconsistencies (change or acceptance) will be made by the Investigator. Before the data freezing, CMV-Stat will code the medical terms according to the following dictionaries (a Validation Coding Report will be prepared by CMV-Stat Biologist in charge and approved by the Sponsor or his delegates):<br>● MedDRA for the pathologies and the Adverse Events<br>● WHO-ATC for the drugs<br>At the end of the study, CMV-Stat will be in charge of the Data Base freezing. The Data Manager in charge will provide the Biostatistics Unit with the cleaned Data Base for the Statistical Analyses.<br>All the details relevant to the data management will be described in the Data Management Plan, which will be prepared by the Data Manager in charge of the study.

**CONTACTS FOR SCIENTIFIC ISSUES**	**Prof. Paolo Marchetti**<br>email: paolo.marchetti@uniroma1.it<br><br>**Dr. Andrea Botticelli**<br><br>Oncologia<br>Azienda Ospedaliera Universitaria - Policlinico Umberto 1<br>Viale del Policlinico, 155<br>00161 Rome<br>email: andrea.botticelli@uniroma1.it

1 INDEX

2 GENERAL INFORMATION 22
2.1 PERSONALIZED MEDICINE 22
3 INTRODUCTION 25
3.1 BACKGROUND INFORMATION 25
3.1.1 NANOSTRING....26
3.2 INVESTIGATIONAL PRODUCT DESCRIPTION 26
4 STUDY RATIONALE AND RISK BENEFIT ASSESSMENT 28
5 PURPOSES AND OBJECTIVES OF THE CLINICAL TRIAL 29
6 EXPERIMENTAL DESIGN....29
6.1 STUDY ENDPOINTS....30
6.1.1 Primary Endpoint 30
6.1.2 Secondary Endpoint 30
6.2 INVESTIGATION PLAN 30
6.3 FLOW CHART (TABLE 1)....31
7 STUDY POPULATION....34
7.1 SAMPLE SIZE 34
7.2 IDENTIFICATION PARAMETERS....34
7.3 PATIENT SELECTION....34
7.3.1 Inclusion Criteria....34
7.3.2 Exclusion Criteria 35
7.4 WITHDRAWAL PROCEDURES AND REPLACEMENT....35
7.4.1 Replacement Policy....36
8 SUBJECT TREATMENT 36
8.1 STUDY TREATMENTS....36
8.1.1 Treatment Packaging, Labeling and Drug Accoutability....36
8.1.2 Destruction 37
8.1.3 Transport and storage and supply Instructions 37
8.2 RANDOMIZATION 37
8.3 CONCOMITANT THERAPIES/TREATMENTS....37
9 STUDY PROCEDURES....38
9.1 VISIT SCHEDULING....39
10 EFFICACY EVALUATION....45
10.1 EFFICACY PRIMARY ENDPOINTS....45
10.2 EFFICACY SECONDARY ENDPOINTS 45

10.3 FOUNDATION ONE 46
10.4 NANOSTRING 48
10.5 LOCAL STUDY SITE LABORATORY ASSESSMENTS 49
10.6 TUMOR ASSESSMENTS AND IMMUNO-MONITORING 50
11 SAFETY REPORTING OF ADVERSE EVENTS 51
11.1 SAFETY AND TOLERABILITY EVALUATION 51
11.2 ASSESSMENT OF SAFETY 52
11.2.1 Specification of Safety Variable 52
11.2.2 Adverse Events 52
11.3 MONITORING AND RECORDING OF AEs 53
11.4 MANAGEMENT OF SPECIAL SITUATION REPORTS 55
11.4.1 Special situation reports 55
11.4.2 Monitoring of Safety information 55
11.4.3 Exchange of special situation reports (AESI) 55
11.4.4 Case transmission verification 55
11.4.5 Aggregate Reports 56
11.4.6 Other Reports 56
11.4.7 Queries 56
11.5 STUDY COMMITTEES 56
11.5.1 DATA MONITORING COMMITTEE 56
11.5.2 MOLECULAR TUMOR BOARD AND STEERING COMMITTEE 57
12 DIRECT ACCESS TO SOURCE DATA/DOCUMENTS 57
13 QUALITY CONTROL AND QUALITY ASSURANCE PROCEDURES 57
13.1 CASE REPORT FORM (CRF): GENERAL INFORMATION 57
13.2 AUDITS 57
13.3 STUDY MONITORING 57
13.4 INSPECTIONS 58
14 DATA HANDLING 58
14.1 DATA MANAGEMENT 58
15 INTERIM ANAYSIS 58
16 STATISTICAL ANALYSIS 59
17 ETHICAL ISSUE 62
17.1 ETHICAL AUTHORIZATIONS 63
17.2 PROTOCOL AMENDMENTS 63
17.3 INFORMED CONSENT 63

18 ADMINISTRATIVE PROCEDURES 63
18.1 CHANGES IN STUDY CONDUCTION OR PLANNED ANALYSIS 63
18.2 STUDY DISCONTINUATION/TERMINATION 64
18.3 ARCHIVING 64
18.4 USE OF INFORMATION AND PUBLICATION OF THE STUDY RESULTS 64
18.5 CIVIL LIABILITY INSURANCE 65
18.6 FINANCING OF THE STUDY 65
19 INVESTIGATOR'S RESPONSIBILITY 65
20 FINAL STUDY REPORT 65
21 BIBLIOGRAPHY 66
22 APPENDICES 71

2 GENERAL INFORMATION

2.1 PERSONALIZED MEDICINE

Personalizing cancer medicine depends on the implementation of personalized diagnostics and therapeutics. Detailed genomic screening is likely to play a central role in this. [1-6]
Personalized Medicine has been widely depicted as a striking innovation, that is able to reform the standard approach to disease management, replacing the *one-size-fits-all* scheme of medicine with a *single-patient-sized* medical intervention.

Personalized medicine promoters usually highlight its potential to combine a more effective health-care with costs containment, according to the following rules:

- monitoring of disease risks and more effective prevention;
- early intervention;
- selection of optimal therapy;
- reduction of trial-and-error prescribing and reduction of adverse drug reactions;
- exclusion of unnecessary drugs;
- therapeutic drug monitoring and disease progression/remission monitoring;
- increased patient compliance with therapy.

But, in spite of expectations, many unsolved practical issues, from technical and scientific to ethical, legal and economic topics, are slowing down the translation of personalized medicine principles into medical practice. Furthermore, wide adoption of personalized strategies also has to deal with the peculiar rules, policy and reimbursement system of each country.

Application of Personalized Medicine in the real world seems entangled by the unmet need to develop evidence-based guidelines.

The benefits of personalized medicine in routine clinical practice have firstly emerged in oncology. The power of precision medicine in the field of anticancer therapy resides in the possibility to characterize the genomic profile of both the disease (eg somatic mutations in the *tumor tissue or blood sample*) and the patient (eg the *germinal genomic profile*). The first piece of information allows stratification of patients in responder and non-responder to specific drugs, improving efficacy and avoiding wasting of expensive medications as biological drugs.
Personalized medicine for cancer can be classified in:

- targeted therapy (which bloks the growth of cancer cells by interfering with specific molecular targets of cancer cells) or
- immunotherapy (which use the body'immune system to fight cancer cells by stimulating the immune system)

Targeted therapy belongs to Personalized Medicine approach and the study of genetic alterations on tumor tissue or blood sample (CTC or cfDNA) are changing the treatment of cancer patients.

In clinical practice, the use of target therapies driven by genomic alteration assessment assessment has radically changed the survival of patients affected by breast cancer, NSCLC, melanoma, colo-rectal cancer.

Despite the efficacy of such approach its use is restricted to a relative small fraction of patients and the evaluation of genomic alterations is conditioned by the primary site of the cancer, i.e. by the histology. The current biological understanding leads to hypothesize that the cancer characteristics are highly dependent on the driver genetic

alterations and not only from the anatomic site of origins. It's widely demonstrated that such genetic alterations are detected regardless of the histology, and this has already modified the treatment of some cancers.

Furthermore, several studies have demonstrated the efficacy of the choice of treatment according to genomic evaluation regardless of its histology with acceptable cost-effectiveness profile.

In the context of precision medicine the Immuno-oncology is becoming Precision Immuno-oncology and the efforts of science are directed towards the identification of predictive biomarkers of response to immune checkpoint inhibitors.

Promising biomarkers are MSI and the tumour mutational load (TMB) In particular TMB a quantitative biomarker that reflects the total number of mutations carried by tumor cells. TMB is well-known to reflect neoantigens burden potentially recognized by the immune system. This has been shown to correlate with better anti-PD-1 response in particular for both pembrolizumab and nivolumab combined with ipilimumab .

The same findings were demonstrated in the OAK study considering peripheral blood mutational load and response to atezolizumab. [33-35]

High tumor mutation burden (defined as tumors that have high ≥10 mutations/megabase, mut/mb) allows to identify 45% of patients who can benefit from immunotherapy regardless of PD-L1 expression.

FOUNDATION ONE CDx

FoundationOne (with updated 324 gene reflecting CDx) is a next generation sequencing based in vitro diagnostic device for detection of substitutions, insertion and deletion alterations (indels), and copy number alterations (CNAs) in 324 genes and select gene rearrangements, as well as genomic signatures including microsatellite instability (MSI) and tumor mutational burden (TMB) using DNA isolated from formalin-fixed paraffin embedded (FFPE) tumor tissue specimens.

Current Gene List[2]

Genes with full coding exonic regions included in FoundationOne®CDx for the detection of substitutions, insertion-deletions (indels), and copy-number alterations (CNAs).

ABL1	ACVR1B	AKT1	AKT2	AKT3	ALK	ALOX12B	AMER1 (FAM123B)	APC
AR	ARAF	ARFRP1	ARID1A	ASXL1	ATM	ATR	ATRX	AURKA
AURKB	AXIN1	AXL	BAP1	BARD1	BCL2	BCL2L1	BCL2L2	BCL6
BCOR	BCORL1	BRAF	BRCA1	BRCA2	BRD4	BRIP1	BTG1	BTG2
BTK	C11orf30 (EMSY)	CALR	CARD11	CASP8	CBFB	CBL	CCND1	CCND2
CCND3	CCNE1	CD22	CD274 (PD-L1)	CD70	CD79A	CD79B	CDC73	CDH1
CDK12	CDK4	CDK6	CDK8	CDKN1A	CDKN1B	CDKN2A	CDKN2B	CDKN2C
CEBPA	CHEK1	CHEK2	CIC	CREBBP	CRKL	CSF1R	CSF3R	CTCF
CTNNA1	CTNNB1	CUL3	CUL4A	CXCR4	CYP17A1	DAXX	DDR1	DDR2
DIS3	DNMT3A	DOT1L	EED	EGFR	EP300	EPHA3	EPHB1	EPHB4
ERBB2	ERBB3	ERBB4	ERCC4	ERG	ERRFI1	ESR1	EZH2	FAM46C
FANCA	FANCC	FANCG	FANCL	FAS	FBXW7	FGF10	FGF12	FGF14
FGF19	FGF23	FGF3	FGF4	FGF6	FGFR1	FGFR2	FGFR3	FGFR4
FH	FLCN	FLT1	FLT3	FOXL2	FUBP1	GABRA6	GATA3	GATA4
GATA6	GID4 (C17orf39)	GNA11	GNA13	GNAQ	GNAS	GRM3	GSK3B	H3F3A
HDAC1	HGF	HNF1A	HRAS	HSD3B1	ID3	IDH1	IDH2	IGF1R
IKBKE	IKZF1	INPP4B	IRF2	IRF4	IRS2	JAK1	JAK2	JAK3
JUN	KDM5A	KDM5C	KDM6A	KDR	KEAP1	KEL	KIT	KLHL6
KMT2A (MLL)	KMT2D (MLL2)	KRAS	LTK	LYN	MAF	MAP2K1 (MEK1)	MAP2K2 (MEK2)	MAP2K4
MAP3K1	MAP3K13	MAPK1	MCL1	MDM2	MDM4	MED12	MEF2B	MEN1
MERTK	MET	MITF	MKNK1	MLH1	MPL	MRE11A	MSH2	MSH3
MSH6	MST1R	MTAP	MTOR	MUTYH	MYC	MYCL (MYCL1)	MYCN	MYD88
NBN	NF1	NF2	NFE2L2	NFKBIA	NKX2-1	NOTCH1	NOTCH2	NOTCH3
NPM1	NRAS	NT5C2	NTRK1	NTRK2	NTRK3	P2RY8	PALB2	PARK2
PARP1	PARP2	PARP3	PAX5	PBRM1	PDCD1 (PD-1)	PDCD1LG2 (PD-L2)		PDGFRA
PDGFRB	PDK1	PIK3C2B	PIK3C2G	PIK3CA	PIK3CB	PIK3R1	PIM1	PMS2
POLD1	POLE	PPARG	PPP2R1A	PPP2R2A	PRDM1	PRKAR1A	PRKCI	PTCH1
PTEN	PTPN11	PTPRO	QKI	RAC1	RAD21	RAD51	RAD51B	RAD51C
RAD51D	RAD52	RAD54L	RAF1	RARA	RB1	RBM10	REL	RET
RICTOR	RNF43	ROS1	RPTOR	SDHA	SDHB	SDHC	SDHD	SETD2
SF3B1	SGK1	SMAD2	SMAD4	SMARCA4	SMARCB1	SMO	SNCAIP	SOCS1
SOX2	SOX9	SPEN	SPOP	SRC	STAG2	STAT3	STK11	SUFU
SYK	TBX3	TEK	TET2	TGFBR2	TIPARP	TNFAIP3	TNFRSF14	TP53
TSC1	TSC2	TYRO3	U2AF1	VEGFA	VHL	WHSC1 (MMSET)	WHSC1L1	WT1
XPO1	XRCC2	ZNF217	ZNF703					

Select Rearrangements[2,3]

Genes with select intronic regions for the detection of gene rearrangements, one gene with a promoter region and one non-coding RNA gene.

ALK	BCL2	BCR	BRAF	BRCA1	BRCA2	CD74	EGFR	ETV4
ETV5	ETV6	EWSR1	EZR	FGFR1	FGFR2	FGFR3	KIT	KMT2A (MLL)
MSH2	MYB	MYC	NOTCH2	NTRK1	NTRK2	NUTM1	PDGFRA	RAF1
RARA	RET	ROS1	RSPO2	SDC4	SLC34A2	TERC*	TERT (promoter only)**	
TMPRSS2								

*TERC is non-coding RNA gene.
**TERT is gene with promoter region.

FOUNDATIONONE LIQUID CDX

FoundationOne Liquid CDx is Foundation Medicine's best-in-class, blood-based circulating tumor DNA assay, designed to interrogate the most druggable cancer-related genes. FoundationOne Liquid CDx was rigorously validated to identify all four classes of alterations, achieving the superior performance you can expect from Foundation Medicine. FoundationOne ®Liquid CDx has been updated to interrogate the same number of cancer-related genes as the FoundationOne® CDx, increasing diagnostic capabilities and increasing its clinical usefulness.

Key Benefits of FoundationOne Liquid CDx:

- Unparalleled accuracy with 99% sensitivity and 99% specificity (PPV) across all four types of genomic alterations - Base substitutions, Insertions and deletions, Copy number variations and Rearrangements / gene fusions
- Integration with FoundationOne results when previous FoundationOne testing has been ordered
- Requires only two tubes of blood and returns results within 14 days.

IMMUNOTHERAPY

Tumor Mutational Burden (TMB) and Microsatellite Instability (MSI) are available on FoundationOne Reports.
FoundationOne comprehensive genomic profiling reports include tumor mutational burden, which is a validated, quantitative genomic biomarker associated with response to cancer immunotherapies in a number of tumor types. Patients with a high TMB are more likely to benefit from certain cancer immunotherapies.
TMB status is provided on every FoundationOne report and does not require additional tissue, or result in increased turnaround time, or cost.
MSI is the condition of genetic hypermutability (predisposition to mutation) that results from impared DNA Mis-Match Repair (MMR). The presence of MSI represents phenotypic evidence that MMR is not functioning normally.

3 INTRODUCTION

3.1 BACKGROUND INFORMATION

Only few studies investigated the efficacy of the choice of treatment according to genomic evaluation regardless of its histology.

The Shiva trial [38] represents the first randomized trial that investigate the role of molecular profile to guide the choice of therapy regardless of the anatomic site of cancer. in the Shiva trial 195 patients were randomized to receive molecularly targeted agent versus therapy at choice of physicians. The study didn’t show an improvement in term of PFS and OS between molecularly targeted agents compared with standard treatment. Despite the expectations the results of Shiva are quite disappointing, but the reason for this failure may depend on the weakness of the design of the study. First, the study considered only few molecular modifications that could be treated with monotherapy while patients with advanced diseases often present several alterations and are unlikely to respond to monotherapy. Furthermore the therapy proposed didn’t consider some molecular and clinical implication, for example they used imatinib for RET alteration or hormone therapy as single agents.

The BATTLE2 study [39] demonstrated that molecular profile-treatment is a promising approach even though the study considered only patients affected by NSCLC and only few molecular alterations (EGFR mutation and/or EGFR amplification/high polysomy; 2) KRAS or BRAF mutation; 3) VEGF and/or VEGFR-2 overexpression; 4) RXR α, β, or γ overexpression and/or cyclin D1 overexpression and/or CCND1)

In the ProfiLER study [41] 2676 patients were enrolled and 609 of them were carrier of at least 1 actionable molecular alteration, whereas 394 had from 2 to 6. The preliminary results showed a better 3-years and 5 years OS rate in patients treated with molecular profile driven therapies.

Other ongoing trials (MATCHT study [42], TAPUR study [43], IMPACT 2 study [44], SAFIRO 02 [45-46]) are investigating the role of molecular-directed therapy. In particular the IMPACT 2 trial is studying the possibility to drive the therapy according to molecular profiling.

3.1.1 NANOSTRING

Description of the equipment

The nCounter Analysis System represents the first and unique platform able to carry out the study up to 800 targets simultaneously for the analysis of gene expression (mRNA and miRNA), Copy Number Variation (DNA), lncRNA, fusion genes, single cells gene expression, proteins, in a fully automatic manner and without the need to use PCR or other enzymatic steps to amplify molecules of interest. This avoids the drawbacks normally resulting from the use of enzyme systems, such as the introduction of bias, creation of errors in the sequences of interest using FFPE samples, enzymatic reaction efficiency and molecules counting comparability between different samples.

Description of the methodology

The patented technology used in NanoString analysis system nCounter is based on color-coded molecular barcodes that can hybridize directly to many different types of target molecules and subsequent spotting on a solid phase, ready to be count. The automated workflow is highly simplified with a total hands of time of 15 minutes, maximizing the performance of the system and minimizing the possibility of errors.

nCounter system consists of two parts:

- nCounter Prep Station: an automatic instrument for the purification of Overall target-probe molecule and subsequent immobilization in a dedicated support for the reading.
- nCounter Digital Analyzer: an automatic scanner used to count the number of target differentiated on the base of the probe unique to each studied gene.

NanoString Technologies provides life science tools for translational research and molecular diagnostic products. The company's nCounter Analysis System has been employed in life sciences research since it was first introduced in 2008 and has been cited in more than 1,900 peer-reviewed publications. Nanostring Techology offers a cost-effective way to easily profile the expression of hundreds of genes, proteins, miRNAs, copy number variationsand SNV facilitating a wide variety of basic research and translational medicine applications, including biomarkers discovery and validation. The company's technology is also being used in diagnostics. The Prosigna® Breast Cancer Prognostic Gene Signature Assay together with the nCounter Dx Analysis System is used as a prognostic indicator for distant recurrence of early breast cancer. Prosigna test is a good example of the traslational potentiality of nCounter system. This assay is using a signature of 50 genes (PAM 50) and a validated algorithm that include also clinical features of breast cancer (size of tumor and nodal status), to provide molecular subtypes and risk of recurrence. In addition, the company is collaborating with multiple biopharmaceutical companies in the development of companion diagnostic tests for various cancer therapies including Immunoterapy, helping to realize the promise of precision oncology.

The tumor profiling based on tissue analisys will represent the reference towards which the analysis of NanoString results will be compared.

The analysis will be performed at the "U.O.C. Laboratorio Analisi Biochimica Clinica-Diagnostica Molecolare Avanzata, directed by Prof. Maurizio Simmaco", Azienda Ospedaliero-Universitaria S. Andrea, via di Grottarossa 1035 -00189 Rome (Italy).

3.2 INVESTIGATIONAL PRODUCT DESCRIPTION

Patients who are eligible according to the inclusion/exclusion criteria will be randomized in 2 treatment groups:

1) STANDARD OF CARE
Patients will be treated according the current version of the AIOM guidelines for their type of cancer. As an example, patients could be treated with standard chemotherapy and/or targeted therapy according to the histological results.

2) TAILORED THERAPY according to the genomic profile
Patients will be treated with target therapy and/or immunotherapy according to their genomic profile evidenced by the FO profiling.
Patients will be treated with one or more drugs of the following list, if a safe combination is avalaible in a phase II trial already conducted

The following is the list of available drugs, that will be used for the purposes of the present study:

**TARGETED THERAPY**	**MOLECULAR TARGET**
ERLOTINIB	EGFR mutation
TRASTUZUMAB, PERTUZUMAB, TDM1, LAPATINIB	ERBB2 amplifications/mut
EVEROLIMUS	mTOR mutations, AKT mut
VEMURAFENIB, COBIMETINIB	BRAFV600E mutations
ALECTINIB, BRIGATINIB	ALK, RET
PALBOCICLIB	CDK4/6, CDKN2A/p16
PONATINIB	Bcr-abl
VISMODEGIB	SMO/PTCH1
ITACITINIB	JAK mutation
INCB054828	FGFR1/2/3
IPATASERTIB	PI3K, AKT, PTEN
ENTRECTINIB	ROS1
ALPELISIB	PI3K, AKT
TEPOTINIB	MET amplification/exon14 skipping mutations (*)
PRALSETINIB	RET
TALAZOPARIB	BRCA1/2, ATM, other HRD status
SELPERCATINIB	RET
**IMMUNOTHERAPY**	**BIOMARKERS**
ATEZOLIZUMAB, NIVOLUMAB, IPILIMUMAB	MSI, HIGH TUMOR MUTATIONAL BURDEN, OTHER

Note: comma (,) means single drugs which can be used for that target.
(*) as primary oncogenic driver

Drugs will be administered according to their respective SmPCs (or IBs in case of drugs under development). More information about each IMP as well as recommendation for toxicity management are present in the Appendix 2 of the protocol .

Any different combination will be reported as a protocol violation and managed according to GCPs requirements. Details on how to document and evaluate such cases will be reported in the study specific Safety Management Plan.

4 STUDY RATIONALE AND RISK BENEFIT ASSESSMENT

The potential of precision medicine has emerged forcefully in recent years. The use of molecular target therapy has spread in almost all cancer, with benefit for patients in term of survival outcomes and reduction of toxicity. Targeted agents have demonstrated to be superior to the standard untargeted treatments particularly in HER2+ breast cancer [50], NSCLC, with EGRF mutations [51] and ALK traslocations [52], colorectal cancer [53,54], BRAF mutated melanoma[55] and more. Moreover, recent drugs agnostic approval, such as pembrolizumab for MSI cancers and entrectinib for cancers carrying NTRK fusions, demonstrated that patients could benefit from target therapy regardless the histology.[56,57] We briefly discuss the state of art of several cancer and how the personalized approach of the ROME trial can be placed in this context.

The status of estrogen receptor (ER), progesterone receptor (PR) and human epidermal growth factor receptor 2 (HER2) and the proliferative rate (usually expressed by KI67%) guide the therapeutic choices in breast cancer patients. In ER+/HER2- patients, cyclin-dependant kinase 4 and 6 inhibitors (iCDK 4/6) represent the most frequent upfront treatment.[58] It is still unclear which the correct treatment has to be offered after a progression to upfront iCDK4/6.[59] Fulvestrant and the combination everolimus plus exemestane are frequently used as hormonal treatments in second line but with an expected objective response rate (ORR) below 10% and a significant clinical benefit reserved to patients without a strong hormone-resistance and non-visceral/non-aggressive disease. An alternative, in patients with extensive visceral progressive disease or primary resistance to hormonal therapy, is chemotherapy. However, despite the several toxicities and the impact on patient's quality of life, the objective response rate (ORR) and survival outcomes of a chemotherapy-based second line are far from those of molecular target therapies[60]. Moreover, recent preclinical studies have demonstrated that several mutation in intracellular pathways (such as Pi3K, RB1 and more) could lead the resistance to iCDK4/6-based first line and become target for tailored therapies.[61,62] In this context, the ROME trial will offer to patients the possibility to receive a tailored therapy based on the results from Foundation-One genomic test on both tissue and blood, not available otherwise in clinical practise. Identifying a targetable mutation could allow patients to receive targeted treatment and delay the use of chemotherapy.

Immunotherapy, in particular the combination atezolizumab, an anti PDL-1 agent, and nab-paclitaxel, has dramatically changed the landscape of treatments for PDL-1 positive triple negative breast cancer (TNBC).[63] However, only a slight number of patients achieve a long-term benefit, while the rest will experience a progression of disease. Moreover, only about 40% of TNBCs are PDL-1 positive, limiting the impact of immunotherapy on a large part of patients. Germline BRCA mutation can be found in about 30% of TNBC, allowing the use of poly-adenosine diphosphate-ribose polymerase inhibitors (PARPi).[64] However, despite the initial benefit, almost every TNBC will become resistant to PARP1. Finally, there is not a clear indication from the main phase III trials and international guidelines on which second and third line mono-chemotherapy treatment is the more effective, with an ORR <20% and a very short survival benefit.[65,58]. In this scenario, the ROME trial will offer to those patients a chance for a tailored therapy not available otherwise.

The landscape of non-small cell lung cancer (NCLC) treatment has completely changed in the last years, thanks to the identification of several molecular targets and the introduction of immunotherapy. Oncogene-addicted NSCLC patients (EGFR mutated, ALK translocated, ROS-1 mutated, and BRAF V600E mutated) will be excluded from our trial due to approved target treatment available in clinical practice [66, 67, 68,69]. Non oncogene-addicted NSCLC patients will benefit from an upfront treatment with immunotherapy (if PDL-1≥50%) or a combination of immunotherapy and chemotherapy (if PDL-1<50%) [70, 71, 72]. However, about 50-60% of patients will have no to short benefit from those treatments, requiring a subsequent chemotherapy-based second line. Palliative mono-chemotherapy with poor clinical and survival outcomes can be administered as third line in those patients with a good performance status (PS). Moreover, several rare targetable genomic alteration have been identified in recent trials, highlighting the efficacy of target therapies in this small population. such as larotrectinib in patients with NTRK fusion.[73] In this context, the ROME trial will offer the extraordinary chance to identify over 300 genomic alteration using FoundationOne test and

use one from about 16 different target therapies to treat NSCLC patients with a progressive disease form first or second line treatments.

Despite advances in knowledge of molecular mechanisms, chemotherapy plus targeted antiangiogenic or anti EGFR agents still represents the backbone of metastatic colorectal cancer (mCRC) treatment [74]. Although a well-established first and second line treatments, the larger part of mCRC patients will experience a progression of disease and the subsequent approved third line treatment with regorafenib or TAS 102 has an ORR <10% and poor impact on survival.[75,76] The ROME trial will offer to patients with progressive mCRC positive to targetable mutation the chance of a tailored treatment, with an supposed better impact on clinical outcomes (attended ORR=20%). In fact, several trials with target agents such as trastuzumab plus lapatinib/pertuzumab in HER2 positive and encorafenib plus cetuximab in BRAF V600E mutated mCRC have shown to be superior to untargeted treatments.[54,77] Finally, one of the first an agnostic FDA drug approval has been immunotherapy in cancer with microsatellite instability (MSI). Impressive results has been seen in metastatic MSI colorectal patients treated with immunotherapy as both rescue and upfront treatment [78].The ROME trial could contribute to evaluate the impact of tailored therapies in mCRC.

This approach could be extended to all other cancer histologies in which the second or third line of treatment has not well defined, has unsatisfactory clinical outcomes or is lack of well-defined therapeutic targets.

5 PURPOSES AND OBJECTIVES OF THE CLINICAL TRIAL

The primary objective of our study is to evaluate the efficacy (meant as overall response rate ORR) of TT vs SoC.

The secondary objective is to evaluate the long term survival (meant as progression free survival PFS) of TT vs SoC.

6 EXPERIMENTAL DESIGN

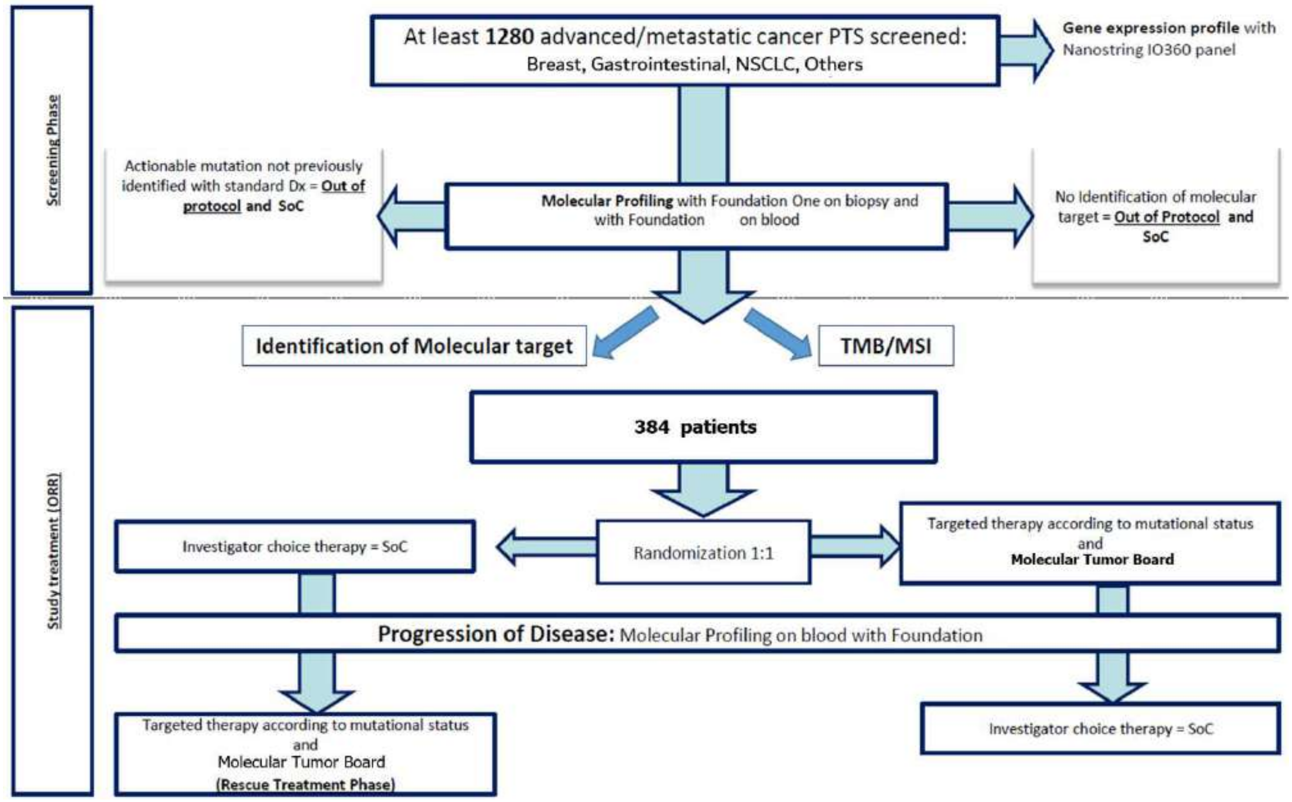

6.1 STUDY ENDPOINTS

6.1.1 Primary Endpoint

Evaluation of the OVERAL RESPONSE RATE (ORR) of the treatment at choice of physicians, according to Standard of Care (SoC) and of the Tailored Treatment (TT).

The ORR will be constructed according to the specific design of the study, therefore including also the Rescue/Swithed Therapy Phase data.

This means that the ORR will take into account 3 evaluations:

- The ORR estimation on the original final population (i.e 384 patients divided into the 4 groups of type of cancer)
- The ORR estimation done on the TT patients, which will include the original randomized TT patients and the patients swithed from the standard of carevtherapy (SoC therapy) to the TT Therapy, this latter within the Rescue Therapy Phase (patients switching upon the first documented progression)
- The ORR estimation done on the population composed by the original TT patients, the original SoC patients and the switched TT patients. This means that the total population analyzed will include the original 384 population data (as per randomization) and the additional switched TT patients.

6.1.2 Secondary Endpoint

Evaluation of the:

- Progression Free Survival (PFS) of SoC vs TT
- Time to Treatment Failure (TTF) of SoC vs TT
- Time to Next Treatment (TTNT) of SoC vs TT
- Concordance between molecular profile on tumor tissue and ctDNA
- QoLs included in the two arms of the study of SoC vs TT
- The safety profile between the two treatment arms of SoC vs TT
- The immune fitness in the two treatment arms of SoC vs TT
- The association between the molecular evaluation and gene expression
- Overall survival (OS) is defined as the time from randomization to death from any cause. Data for patients with no record of death will be censored at the last date they were known to be alive. The analysis of OS will follow the same methodology as the primary endpoint. At the time of the primary analysis, an interim analysis of OS will be performed.

6.2 INVESTIGATION PLAN

This study is a Phase II, randomized, multicenter, Proof of Concept, clinical trial. Patients with progressive disease (recurrent and/or metastatic) of breast cancer, metastatic gastro-intestinal tumors, non small cell lung cancer (NSCLC) or others will be included. Patients should have completed at least 1 line of treatment and no more than 2 as defined by the current version of the AIOM guidelines. Patients are included if surgery is contraindicated.

Patients could have received targeted therapy for metastatic disease.

A molecular profile of the cancer will be evaluated on tumor tissue biopsy and on ctDNA of around 1280 patients at patient inclusion.

After FO evaluations patients with actionable mutations, not previous identified with other methods, for which approved drugs according to histotype are available, will be excluded.

Once identified molecular abnormalities (not only those that are disease-specific), that can be modulated with target or immunotherapeutic intervention available within the present study, patients will be randomized to receive:

ARM A: Therapy at choice of physician, according to Standard of Care (SoC)
ARM B: Tailored treatment according to genomic profile (Tailored Treatment, TT)

A specific manual for FO will detail genetic tests management.
The Molecular Tumor Board (MTB – refer to section 10.5.2) will define the target therapy and immunotherapy while standard treatment will be decided by study physicians.
Patients should remain in the treatment phase of the study until investigator assesses radiographic or clinical progressive disease, unmanageable toxicity, or study termination.
Tumor assessments will be conducted every 12 weeks from the date of randomization until any of the above events occurs. Specific TT (i.e. ipilimumab plus nivolumab) may require a different time to tumor assessment.
Delays in treatment administration will not impact the timing of the tumor assessments. If a tumor assessment must be performed early/late, subsequent assessments will be conducted according to the original schedule of every 12 weeks from the date of randomization.
Tumor assessments must be conducted until progressive disease (PD for RECIST 1.1 or iCPD for iRECIST if clinically indicated), even if treatment has been discontinued due to investigator-determined PD or unacceptable toxicity.
After discontinuation of study treatment for reason different from progressive disease and withdrawal of consent, tumor assessments will continue until progression.
In addition, patients will be followed for survival until death, loss to follow-up, withdrawal of consent, or study termination.

6.3 FLOW CHART (TABLE 1)

STUDY PROCEDURES	SCREENING PHASE	RANDOMIZATION		TREATMENT PHASE					RESCUE/SWITCHED THERAPY PHASE				SURVIVAL FOLLOW-UP (OS)
	V0 (D -45;-D -1)		C1D1 Baseline V1 Day 0	C2D1 V2 Day 28 (±7)	C3D1 V3 Day 56 (±7)	C4D1 V4 Day 84 (±7)	First Progression (PD1) SWITCH OF THERAPY	C1D1 V_{n+1} Day 1 (±7)	C2D1 V_{n+2} Day 28 (±7)	C3D1 V_{n+3} Day 56 (±7)	C4D1 V_{n+4} Day 84 (±7)	Second Progression (PD2) / End of study (notes 14, 15)	(Every 4 Months after second progression /end of study)
				Repeat the same schedule until Progression of Disease					Repeat the same schedule until Progression of Disease				
Informed Consent Form (PART 1)	X												
Informed Consent Form (PART 2)***			X										
Inclusion/Exclusion Criteria	X												
Medical History	X												
Oncological Medical History *(note1)*	X												
Pregnancy test *(note 2)*	X												
ECOG PS *(note 3)*	X		X	X	X	X		X	X	X	X	X	
Physical examination and vital signs *(note 4)*	X		X	X	X	X		X	X	X	X	X	
Local Laboratory Assessments *(note 2)*	X		X	X	X	X		X	X	X	X		
ECG *(note 5)*	X					X					X		
Total body CT scan *(note 6)*	X					X					X		
Bone scan *at the screening and only if clinically indicated*	X					X					X		
MRI *only if clinically indicated (note 6 and 7)*	X					X					X		
PET/TC *only if clinically indicated (Note 8)*						X					X		
RECIST ASSESSMENT						X					X		
irRC ASSESSMENT *(note 9)*						X					X		
Tumor Tissue and biomarker analysis (Foundation One)	X												
ctDNA analysis (Foudantion One Liquid)	X						X						
Gene cancer Immunoprofiling (Nanostring)	X												
Molecular Tumor Board (MTB)	X						X						
Randomization		X											
Drug administration *(note 10)*			X	X	X	X		X	X	X	X		
Adverse event *(note 11)*	X		X	X	X	X		X	X	X	X	X	
Concomitant treatment *(note 12)*	X		X	X	X	X		X	X	X	X	X	
QoL (EORTC-QL-C30), PROS *(note 13)*			X			X					X	X	
Blood sample for immuno-monitoring *(note 2)*			X	X		X	X						
Treatment Switch							X						
End of study *(note 14)*												X	
Visit or phone contact													X

*** Part 2 of the informed Consent Form will be signed by all the patients randomized in the TT arm and by patients who switch from SoC to TT, in case of PD.

NOTES

Note 1: only oncological diseases

Note 2 (for the full list of parameters please refer to paragraphs 10.5, 10.6 and 10.7):

- Hematology, blood chemistry, INR, and activated partial thromboplastin time (aPTT) should be performed at a local laboratory of the study site in the 7 days prior to each administration of study medication (i.e. from Day 21 to Day 28 of each cycle of treatment) or when clinically indicated. INR and aPTT will be collected for all patients receiving therapeutic doses of anti-coagulants before the start of every therapy cycle, and when clinically indicated
- Pregnancy Test. A serum β-HCG test should be performed for all women of childbearing potential and for all women < 1 years after the onset of menopause. Testing should be performed at the local laboratory of the study site within 72 hours prior to each administration of study medication (i.e. at Day 21 of each cycle of treatment). For all other women, documentation must be present in medical history confirming that the patient is not of childbearing potential
- Serum sample for immuno-monitoring should be collected in the 7 days prior to each administration of study medication (i.e. from Day 21 to Day 28 of each cycle of treatment). The samples will be submitted to the dedicated central laboratory. Samples for immuno-monitoring evaluation will be collected at baseline (v1), at the first cycle of treatment (V2), at the first lesion evaluation and in case of PD.
- HIV, HBV, or HCV have to be performed in local laboratory if historical assessments are not available (Only at V0, Screening Phase).
- Specific IMPs blood test or diagnostic procedures: Other blood test (or diagnostic procedures) specifically required for the IMPs administered to the patient in TT arm have to be performed according to details the specific section of the protocol (Appendix 2)

Note 3: ECOG performance status must be performed before administration of each study treatment

Note 4: Symptom-directed physical examination including vital signs (blood pressure, pulse rate, and body temperature) and weight every cycle

Note 5: 12-Lead ECG and ECHO every 12 weeks

Note 6: Tumor assessments (CT scans, MRI scans, etc.) will be performed every 12 weeks after the date of randomization and will continue until confirmed progressive disease. If a tumor assessment must be performed early or late, subsequent assessments should be conducted according to the original schedule of every 12 weeks from the date of randomization. To ensure comparability, the techniques used for tumor assessment at Screening/Baseline should be consistent with those used subsequently in the study. Specific TT (ie ipilimumab plus nivolumab) may require a different time to tumor assessment. The first tumor assessments with ipilimumab plus nivolumab should be performed after the first 4 administration of ipilimumab.

Note 7: Brain CT/MRI scans should be performed on patients with symptoms/signs suggestive of CNS involvement or other unexplained neurological symptoms and the same institution/facility used at baseline should be used throughout the study, to the extent possible

Note 8: The diagnosis may be made on the biopsy of a metastatic site if primary tumor is not available (referring to target leasion mapping)

Note 9: In patients treated with immunotherapy, radiological evaluation should be done according to iRECIST. In case of iuPD a radiological confirmation is needed at 4-8 weeks according to iRECIST.

Note 10: The Drug administration has to be stopped in case of PD1 to start the Rescue/Switched Therapy Phase

Note 11: Adverse Events (AEs) including SAEs should be documented according to NCI-CTCAE, last updated version at each visit

Note 12: Concomitant medications and cancer-related surgery or procedures should be documented at each visit; including prescription, over-the-counter, and herbal/homeopathic remedies and/or therapies, as well as any cancer-related diagnostic, therapeutic, or surgical procedures performed

Note 13: QoL EORTC QLQ-C30 must be completed by patients at the time of tumor assessment and in any case within an interval of no more than 3 days

Note 14: The end of study visit has to be performed after second PD (PD2). In case of withdrawal, death or lost to follow the last visit performed has to be considered as end of study visit.

Note 15: Diagnostic procedures, QoL will be performed unless they have been performed within 7 days before the end of study visit

7 STUDY POPULATION

7.1 SAMPLE SIZE

A total of around 1280 patients will be screened from about 50 sites in Italy.

7.2 IDENTIFICATION PARAMETERS

Patients with progressive disease (recurrent and/or metastatic) of breast cancer, metastatic gastro-intestinal tumors, non small cell lung cancer (NSCLC) or others. Patients should have completed or failed at least 1 line of treatment and no more than 2 as defined by the current version of the AIOM guidelines.. Patients who are candidates to potentially curative surgery or other locoregional treatment are excluded.

7.3 PATIENT SELECTION

7.3.1 Inclusion Criteria

1. Age ≥ 18 at time of signing Informed Consent Form
2. Patients able and willing to provide a written informed consent to participate to the study
3. Patients with recurrent/metastatic breast, gastrointestinal cancer,non small cell lung cancer or others
4. Patients not treatable with potentially curative surgery ot other loco-regional treatments.
5. Patients should have been completed or failed at least 1 line of treatment for breast cancer, gastro-intestinal, non small cell lung cancer or other cancer.
6. ECOG performance status from 0 to 1
7. Molecular target not actionable with approved drugs identified during screening by profiling with FoundationOne CDX on biopsy and FoundationOne Liquid CDx on blood
8. Biopsiable disease (tumor biopsy mandatory for tumor profiling). The biopsy must be performed during the screening period, when patients complete the conventional therapy for their recurrent/metastatic cancer. Historical samples will be considered for the study if collected within 3 months before the ICF signature of the patient. Samples older than 3 months, with a maximum timeframe of 6 months, and collected before progression of disease after the last treatment administred will be considered upon clinical judgement of the Investigator, after confirmation by the coordinating site or MTB. Samples obtained from a biospy of a metastatic lesion in progression after the last treatment administred represent the optimal tissue sample for genomic testing. Patients with glioblastomas and high grade malignant gliomas can be enrolled with the historical tissue samples
9. Measurable disease, eligible to standard treatment. Patients must have measurable or evaluable disease defined, per RECIST 1.1 or irCS (immune related Response Criteria), as at least one lesion that can be accurately measured in at least one dimension (longest diameter to be recorded for non-nodal lesions and short axis for nodal lesions) as ≥20 mm with conventional techniques or as ≥10 mm with spiral computed tomography (CT) scan, Magnetic Resonance Imaging (MRI), or a subcutaneous or superficial lesion that can be measured with calipers by clinical exam. For lymph nodes, the short axis must be ≥15 mm. Patients who have assessable disease by physical or radiographic examination but do not fully meet the above definitions of measurable disease (but still remains measurable) are eligible and will be considered to have evaluable disease. Patient's whose disease cannot be objectively measured by physical or radiographic examination (e.g., elevated serum tumor marker only) are NOT eligible. PET scan could be performed, if clinically indicated. For PET response evaluation PERCIST criteria will be applied.
10. Adequate renal function defined by a serum creatinine <1.5xUNL (upper normal limit).
11. Adequate liver function test defined by SGOT & SGPT <3xUNL (5xUNL in case of liver metastases), and bilirubin level <1.5xUNL

12. Adequate bone marrow function defined by platelets >100,000/mm3, hemoglobin >10 g/dL, and neutrophils >1,000/mm3
13. For female of child-bearing potential and for all women < 1 years after the onset of menopause: a negative pregnancy test <72 hours before starting study treatment is required. If sexually active, female of childbearing potential must use "highly effective" methods of contraception for the study duration. Contraception should continue after the last treatment for 3 months or for longer periods according to what reported in the APPENDIX 1
14. For male of reproductive potential: any sexually active male patient must use a condom while on study treatment. Contraception should continue after the last treatment for 3 months or for longer periods according to what reported in the Appendix 1.

7.3.2 Exclusion Criteria

1. Patients who have only bone and/or brain metastases
2. Patients treated with more than 2 lines for breast cancer, gastro-intestinal, non small cell lung cancer and other cancer
3. Patients with uncontrolled disease (untreated and/or sintomatic) and patients whose brain metastases have not been monitored for >2 months
4. Patients with well-established actionable targets for which approved and marketed targeted drugs are available (i.e. lung cancer with EGFR mutation, or ALK translocation, B-RAF mutant melanoma, GIST with KIT mutations or breast cancer with HER2 amplification)
5. Patient participating in another clinical trial with an experimental drug
6. Anticoagulation with anti-vitamin K (Low Molecular Weight Heparin [LMWH] is allowed)
7. Patients with other concurrent severe and/or uncontrolled medical disease which could compromise participation in the study, including uncontrolled diabetes, cardiac disease, uncontrolled hypertension, congestive cardiac failure, ventricular arrhythmias, active ischemic heart disease, myocardial infarction within one year, chronic liver or renal disease, active gastrointestinal tract ulceration, severely impaired lung function
8. Pregnant and/or breastfeeding women
9. Patients with any psychological, familial, sociological or geographical condition potentially hampering compliance with the study protocol and follow-up schedule
10. HIV, HBV, or HCV infection as per specific test performed at the screening visit or known as per Medical History
11. Patients with documented contraindication to any of the IMPs that will be used for the study, as reported in the respective SmPcs/IBs and in Appendix 2
12. Patients treated with the following drugs, because of the risk of immunosuppression: Chronic or high-dose oral corticosteroid therapy, TNF-inhibitors and Anti-T cell antibodies

7.4 WITHDRAWAL PROCEDURES AND REPLACEMENT

Patients may withdraw from the study at any time for any reason. Investigators may withdraw patients from the study and/or from study treatment in the event of intercurrent illness, adverse events, protocol violation, administrative reasons, or for other reasons. Patients who prematurely drop from study treatment will continue to be followed for post-treatment assessments, unless patients withdraw their consent. Excessive patient withdrawals can render the study uninterpretable; therefore, unnecessary withdrawal of patients should be avoided. If a patient decides to withdraw, all efforts should be made to complete and report study assessments as thoroughly as possible. The investigator should contact the patient or a responsible relative by telephone or through a personal visit to establish as completely as possible the reason for the withdrawal. All actions taken to complete such data should be documented in the patient's chart. A complete final evaluation at the time of the patient's withdrawal should be made with an explanation of why the patient is withdrawing from the study. If the reason for removal of a patient from the study is

an adverse event, the principal specific event will be recorded first in the applicable source documentation (such as the patient's chart) and then will be recorded on the eCRF. In case a patient decides to prematurely discontinue study treatment ("refuses treatment"), the patient should be asked if she or he may still be contacted for further scheduled study assessments. The outcome of that discussion should be documented in both the medical records and in the eCRF.

7.4.1 Replacement Policy

FOR PATIENTS

Patients randomized into the study will not be replaced.

FOR CENTERS

A center may be replaced for the following administrative reasons:

- Excessively slow recruitment
- Poor protocol adherence

8 SUBJECT TREATMENT

8.1 STUDY TREATMENTS

The schedule of drugs and the dose modifications will be done according to their respective SmPCs (or IBs in case of drugs under development). More information about each IMPs as well as recommendation for toxicity management are present in Appendix 2 of the protocol

8.1.1 Treatment Packaging, Labeling and Drug Accountability

All the drugs will be labelled according to applicable laws and regulations and according to the Annex 13 of the Good Manufacturing Practice requirements and recorded in local language. An example of the label used will be provided as a separated document for the ethical approval. Each patient will receive the adequate quantity of drugs, according to the study prescription schedule.

The packaging and the labeling of the drugs is responsibility of the Company . The Company will also provide all related QP Certifications.

The Principal Investigator and/or his delegates are responsible of the drugs dispensation and of their accountability and reconciliation, according to the scheduling time specified in the flow chart of the study. The Principal Investigator and/or his delegates should record the data relevant to the accountability in the patient's medical record first then in a dedicated form, and in a specific mask of the eCRF. The drug accountability will be managed directly with the eCRF.

The Sponsor is responsible for ordering the drugs for the investigator sites.

Drug shipment/distribution from Company/ packaging to the Investigator Sites will include the use of qualified shipping boxes. A temperature log must be maintained to make certain that the drugs are supplied at the correct temperature.

The Sponsor, must ensure the appropriate storage and management of the drugs on the investigator's site. The Sponsor has also the responsibility to perform training of sites including acknowledgement of receipt procedure for either cold chain and/or ambient drugs.

The investigator and/or his delegates will receive the drugs ordered by the Sponsor when have been obtained the approval of the study protocol by the IRB/IEC and following requirements:

- availability of a signed and dated clinical trial contract between parties
- approval/notification of the regulatory authority
- availability of the curriculum vitae of the principal investigator

- availability of a signed and dated clinical trial protocol or immediately imminent signing of the clinical trial protocol

8.1.2 Destruction

The destruction of unused and/or expired drugs should be performed at participating sites in accordance to local regulation and to the agreement stipulated with the Sponsor.The Sponsor must ensure that the record of destruction is available and correctly archived. If destruction is not possible on site, the Sponsor will provide for collection and disposal after the study team has carried out the reconciliation of the medicinal product under study..

8.1.3 Transport and storage and supply Instructions

Specific instructions on transport, storage and supply will be defined in the study specific guideline. The Company will provide specific documents regarding drugs supply on request of Sponsor (i.e CoC/Batch Certificate, CoA, TSE/BSE Statement, Stability Statement regarding Transport and Storage Conditions, Master Safety Data File).
In case of complaint and temperature Deviations/Recall, immediately contact the sponsor as provided in the instructions for dispensing and storage in the ISF, than the Sponsor is responsible to notify to the Company about any product complaint or temperature deviation related to drugs supplied by the Company (Report complaint/temperature deviations with a dedicated form sent to the Company). The Company is responsible to evaluate the complaint/temperature deviation and provide the final decision about the use of the affected drugs.

8.2 RANDOMIZATION

Four randomization lists, one for each of the four cancer-site cohort, in a ratio of 1 : 1, will be prepared by the Independent Statistician in charge of the study who will maintain it during the whole course of the study. The lists will be centralized. They will be generated and managed by an IWR System implemented as a function in the eCRF by qualified vendor. The Investigator will be in charge of the access to the system. Once the patient performs all the screening procedures, the Investigator will access the Randomization page and, by choosing the cohort to which the patient belongs, asks for the randomization number, which will be unique across all the centers (centralized list) in each cohort. Once the randomization number is assigned, the system will send an email to the Investigator and all the team members included in the mailing list, in order to confirm the randomization number assignment. Details on the procedures to be followed will be included in a specific document.

8.3 CONCOMITANT THERAPIES/TREATMENTS

Patients should receive full supportive care including transfusion of blood and blood products, antibiotics, etc., according to standard of care, when necessary.
All protocol-allowed medications taken by the patient for concomitant disease should continue as necessary during the study and be recorded on the electronic case report form (eCRF).

All information and details about drug-to-drug interaction of every single IMPs present the experimental list of Tailored Therapy (TT) can be found in the dedicated section (Appendix 2)

The following list of allowed medications is provided as guidance. Treatments prescribed to patients should be adapted according to the local standard of care practice.
The following treatments/procedures are permitted:

- Bisphosphonates may be given according to their product license and routine clinical practice, at the investigator’s discretion

- Palliative surgical procedures. Any diagnostic, therapeutic or surgical procedure performed during the study period should be recorded including the dates, description of the procedure(s), and any clinical findings. Patients that will perform a surgery after which the disease will be no more measurable or evaluable have to withdraw from the study.
- As a precautionary measure, it is recommended, but not strictly required, that if patients require placement of a central venous access device (CVAD), the procedure should be done 7 days prior to first study treatment start. The date of CVAD placement should be noted in the medical record and recorded in the eCRF. Episodes of CVAD replacement should be recorded, as should CVAD-related thrombosis, infection, or dysfunction.
- Anti-coagulation therapy for maintenance of patency of permanent indwelling IV catheters is permitted, while anti-coagulation with anti-vitamin K is prohibited.
- Palliative radiotherapy. Radiotherapy is only allowed during the study treatment period for the indication of bone or brain lesions present at baseline. If a patient requires radiation therapy to a new lesion, that new lesion would, per Response Evaluation Criteria in Solid Tumors (RECIST or irRC), qualify as progressive disease.
- Acceptable methods of contraception must be used when the female patient or female/male partner is not surgically sterilized or does not meet the study definition of post-menopausal (12 months of amenorrhea).

The following treatments are not permitted:

- Treatment with other systemic anti-cancer agents (e.g., chemotherapy, hormonal therapy, immunotherapy) or other treatments not part of protocol-specified anti-cancer therapy.
- Concurrent investigational agents of any type.
- Initiation of herbal remedies for cancer treatment. Herbal remedies initiated prior to study entry and continuing during the study are permitted and must be reported on the appropriate eCRF.
- All concomitant medications which are not allowed or strongly discouraged for every single IMPs in the TT list is reported in the dedicated section of the protocol (Appendix 2).

The following treatments should be avoided because of the risk of immunosuppression:

- Chronic or high-dose oral corticosteroid therapy.
- TNF-inhibitors.
- Anti-T cell antibodies.

9 STUDY PROCEDURES

All patients must provide written Informed Consent Form (ICF) before any study-specific assessments or procedures are performed.

An Eligibility Screening Form (ESF) documenting the patient's fulfillment of the entry criteria is to be completed by the investigator/designee for all patients considered for the study. The completed form will be filed in the patient's chart and considered as source document. Patients who are considered for study entry but fail to meet the eligibility requirements (therefore the screening failure patients) should also have an ESF completed with the reason for lack of eligibility well reported, since this provides information on the selected trial population. This screening information will be entered in the eCRF.

This study will have a two-part consent process. All patients must complete Part 1 of the ICF during the Screening Period. Part 1 of the ICF will cover all aspects of the trial except for information related to any specific tailored therapies (TT). Part 2 of the ICF will provide information related to the specific tailored therapy assigned to an individual patient by IWRS and it must be signed only by the patients randomized in TT arm.

Patients who are randomized in the SoC arm will have to complete Part 2 of the ICF just in case of swhitch to the TT treatment due to Progression Disease.

Patient-reported outcomes (PROs) will be used to evaluate physical, mental and social health status of patients (PRO questionnaires are used to assess symptoms and functions, and to enhance communication between clinicians and patients). The QoL that will be used is the EORTC QLQ-C30, a validated questionnaire designed to assess different aspects of health and quality of life for cancer patients. It is a copy right instrument which has been translated and validated in over 110 languages, and therefore a certified Italian version will be used on the purposes of the present study.

9.1 VISIT SCHEDULING

SCREENING EXAMINATION AND ELIGIBILITY SCREENING FORM (VISIT 0)

The following screening tests and procedures must be completed between Day -45 days and Day -1 (i.e. during the screening period) (except where indicated). The Baseline is considered as Day 0 during which the randomization procedure will be implemented and the drug will be administered. The timing of all procedures is summarized in Table 1 (flow chart). The patient will undergo to the administration of the Informed Consent (Part 1) at the Screening Visit, before any of the procedures listed below will be implemented:

- Review of inclusion and exclusion criteria
- Eligibility Screening Form completion
- Complete medical history and demographics including clinically significant diseases within the last 5 years, smoking history, complete cardiovascular history and all medication taken over the last 90 days prior to randomization including prescription, over the counter, and herbal/homeopathic remedies and/or therapies
- Oncological Medical History including cancer history, prior cancer therapies and procedures
- Pregnancy Test. A serum β-HCG test should be performed for all women of childbearing potential and for all women < 1 years after the onset of menopause. For all other women, documentation must be present in medical history confirming that the patient is not of childbearing potential
- ECOG performance status
- Complete physical exam, including vital signs (blood pressure, pulse rate, and body temperature) and physical measurements (body weight and height)
- Hematology, blood chemistry, INR, and activated partial thromboplastin time (aPTT) should be performed at a local laboratory of the study site
- HIV, HBV, or HCV have to be performed if historical assessments are not available
- 12-Lead ECG and LVEF assessment (ECHO)
- Tumor assessment should be performed as specified in Section 10.6 to ensure comparability, the techniques used for tumor assessment at screening should be consistent with those used subsequently in the study, e.g., MRI, CT, PET, bone scans, etc., as well as cytological (e.g., relevant cytology reports documenting malignant pleural effusions, bone marrow aspirations, cerebral spinal fluid, etc.) and photographic data, if available. A CT or MRI brain scan is to be performed at screening only in patients with signs or symptoms suggesting CNS involvement or other unexplained neurological symptoms, and during the study, if clinically indicated.
- Bone scan if clinically indicated
- Tumor tissue and biomarker analysis. The relevant analysis will be performed by FoundationOne CDX on tumor blocks in the central laboratory. Historical samples will be considered for the study if collected within 3 months from the ICF signature of the patient. Tissue samples should be collected after progression of disease from the last treatment. Samples older than 3 months, with a maximum timeframe of 6 months, and collected before progression of disease after the last treatment administered will be considered upon clinical judgement of the Investigator, after confirmation by the coordinating site or MTB. Samples obtained from a biospy of a metastatic lesion in progression after the last treatment administered represent the optimal tissue sample for genomic testing.

Patients with glioblastomas and high grade malignant gliomas can be enrolled with the historical tissue samples considering the difficulties related to repeat the biopsy on CNS

- ctDNA analysis. The analysis will be performed by FoundationOne Liquid CDx on blood sample in the central laboratory
- Gene cancer immunoprofiling. The analysis will be performed by Nanostring Technology on samples in the central laboratory
- Adverse events (AEs) will be collected starting from the Informed Consent signature. Adverse Events (AEs) including SAEs should be documented according to NCI-CTCAE, last updated version at each visit or when AEs occur
- Concomitant medications and cancer-related surgery or procedures should be documented at each visit; including prescription, over-the-counter, and herbal/homeopathic remedies and/or therapies, as well as any cancer-related diagnostic, therapeutic, or surgical procedures performed. Drug to drug interaction has to be evaluated referring to dedicated section of the protocol (Appedix 2)

RANDOMIZATION

The randomization will take place only after completion of all clinical activities planned in the screening phase and after obtaining the FoundationeOne reports. The genomic profiling reported in the reports will be evaluated in ad hoc meetings of the Molecular Tumor Board to identify the possible TT treatment for the patient and therefore the possibility to randomize the patient in the study.

If the MTB gives indication to randomize the patient in the study, it will be the PI to carry out this action, through the appropriate page of the eCRF.

BASELINE PROCEDURES (C1D1 – VISIT 1)

Procedures listed below need to be performed at Baseline:

- Pregnancy Test. A serum β-HCG test should be performed for all women of childbearing potential and for all women < 1 years after the onset of menopause. Testing should be performed at the local laboratory of the study site within 72 hours prior to each administration of study medication. For all other women, documentation must be present in medical history confirming that the patient is not of childbearing potential
- ECOG performance status, to be collected before the administration of the study medication
- Complete physical exam, including vital signs (blood pressure, pulse rate, and body temperature) and physical measurements (body weight and height), to be collected before the administration of the study medication. In case of infusion therapy vital signs will be recorded also after infusion
- Hematology, blood chemistry, INR, and activated partial thromboplastin time (aPTT) should be performed at a local laboratory of the study site in the 7 days prior to each administration of study medication (i.e. from Day 21 to Day 28 of each cycle of treatment) or when clinically indicated. For the full list of analites please refer to paragraphs 10.5. INR and aPTT will be collected for all patients receiving therapeutic doses of anti-coagulants before the start of every therapy cycle, and when clinically indicated
- If the patient is assigned to the TT arm, she/he must sign Part 2 of the Informed Consent Form.
- Administration of study medication according to the assigned treatment arm by IWRS
- Adverse Events (AEs) including SAEs should be documented according to NCI-CTCAE, last updated version at each visit or when AEs occur
- Concomitant medications and cancer-related surgery or procedures should be documented at each visit; including prescription, over-the-counter, and herbal/homeopathic remedies and/or therapies, as well as any cancer-related diagnostic, therapeutic, or surgical procedures performed. Drug to drug interaction has to be evaluated referring to dedicated section of the protocol (Appendix 2)
- Administration of the QoLs EORTC QLQ-C30
- Serum sample for immuno-monitoring will be collected and stored at the site until the shipment to the central laboratory.

TREATMENT PERIOD ASSESSMENTS AND PROCEDURES
During the treatment period, a window of ± 7 days will apply to all visits and assessments, unless otherwise specified. Depending on the visit, specific procedures need to be performed as below indicated:

EVERY VISIT WITHOUT TUMOR ASSESMENT UNTIL PROGRESSION OF DISEASE OR STUDY TERMINATION

- Pregnancy Test. A serum β-HCG test should be performed for all women of childbearing potential and for all women < 1 years after the onset of menopause. Testing should be performed at the local laboratory of the study site within 72 hours prior to each administration of study medication.For all other women, documentation must be present in medical history confirming that the patient is not of childbearing potential
- ECOG performance status, to be collected before the administration of the study medication
- Complete physical exam, including vital signs (blood pressure, pulse rate, and body temperature) and physical measurements (body weight and height), to be collected before the administration of the study medication. In case of infusion therapy vital signs will be recorded also after infusion
- Hematology, blood chemistry, INR, and activated partial thromboplastin time (aPTT) should be performed at a local laboratory of the study site in the 7 days prior to each administration of study medication (i.e. from Day 21 to Day 28 of each cycle of treatment) or when clinically indicated. For the full list of analites please refer to paragraphs 10.5. INR and aPTT will be collected for all patients receiving therapeutic doses of anti-coagulants before the start of every therapy cycle, and when clinically indicated
- Administration of study medication according to schedule of study medications
- Adverse Events (AEs) including SAEs should be documented according to NCI-CTCAE, last updated version at each visit or when AEs occur
- Serum sample for immuno-monitoring will be collected only at **C2D1 – VISIT 2** and stored at the site until the shipment to the central laboratory**.**
- Concomitant medications and cancer-related surgery or procedures should be documented at each visit; including prescription, over-the-counter, and herbal/homeopathic remedies and/or therapies, as well as any cancer-related diagnostic, therapeutic, or surgical procedures performed. Drug to drug interaction has to be evaluated referring to dedicated section of the protocol (Appendix 2)
- Some treatments could require a treatment cycle different from 4 weeks. In this case a new visit should be added on CRF and considered as a visit without tumor assessment. In any case,tumor assessment should be performed every 12 weeks as required. Specific TT (ie ipilimumab plus nivolumab) may require a different time to tumor assessment.

EVERY VISIT WITH TUMOR ASSESMENT (EVERY 12 WEEKS after first drug administration) UNTIL PROGRESSION OR STUDY TERMINATION

- Pregnancy Test. A serum β-HCG test should be performed for all women of childbearing potential and for all women < 1 years after the onset of menopause. Testing should be performed at the local laboratory of the study site within 72 hours prior to each administration of study medication (i.e. at Day 21 of each cycle of treatment). For all other women, documentation must be present in medical history confirming that the patient is not of childbearing potential
- ECOG performance status, to be collected before the administration of the study medication
- Complete physical exam, including vital signs (blood pressure, pulse rate, and body temperature) and physical measurements (body weight and height), to be collected before the administration of the study medication. In case of infusion therapy vital signs will be recorded also after infusion
- Hematology, blood chemistry, INR, and activated partial thromboplastin time (aPTT) should be performed at a local laboratory of the study site in the 7 days prior to each administration of study medication (i.e. from Day 21 to Day 28 of each cycle of treatment) or when clinically indicated. For the full list of analites please refer to

paragraphs 10.5 . INR and aPTT will be collected for all patients receiving therapeutic doses of anti-coagulants before the start of every therapy cycle, and when clinically indicated

- 12-Lead ECG and LVEF assessment (ECHO) every three month and as clinically indicated
- Tumor assessments (CT scans, MRI scans, etc.) will be performed every 12 weeks after the date of randomization and will continue until confirmed progressive disease. If a tumor assessment must be performed early or late, subsequent assessments should be conducted according to the original schedule of every 12 weeks from the date of randomization. To ensure comparability, the techniques used for tumor assessment at Screening/Baseline should be consistent with those used subsequently in the study. Specific TT (ie ipilimumab plus nivolumab) may require a different time to tumor assessment. The first tumor assessments with ipilimumab plus nivolumab should be performed after the first 4 administration of ipilimumab.
  Brain CT/MRI scans should be performed on patients with symptoms/signs suggestive of CNS involvement or other unexplained neurological symptoms. and the same institution/facility used at baseline should be used throughout the study, to the extent possible. PET scan could be performed, if clinically indicated. For PET response evaluation PRECIST criteria will be applied
  The current version of the RECIST Criteria (unidimensional tumor measurement) will be used to evaluate response and assess progressive disease.
- Bone scan as clinically indicated
- Administration of study medication according to schedule of study medications. (Do not administrate if PD to tumor assestment according to RECIST)
- Adverse Events (AEs) including SAEs should be documented according to NCI-CTCAE, last updated version at each visit or when AEs occur.
- Concomitant medications and cancer-related surgery or procedures should be documented at each visit; including prescription, over-the-counter, and herbal/homeopathic remedies and/or therapies, as well as any cancer-related diagnostic, therapeutic, or surgical procedures performed
- QoL EORTC QLQ-C30 must be completed by patients at the time of tumor assessment and in any case within an interval of no more than 3 days
- Serum sample for immuno-monitoring should be collected only at C4D1 – VISIT 4 and stored at the site until the shipment to the central laboratory.

N.B: The visits will be carried out as indicated above. The subsequent cycles of visit each will be repeated as described until the first disease progression (PD) or end of study.
During the treatment phase, extra visits should be scheduled at the clinician's discretion and registred on eCRF (UNSCHEDULED VISIT).
Some treatments could require a treatment cycle different from 4 weeks. In this case a new visit should be added on CRF and considered as a visit without tumor assessment. In any case,tumor assessment should be performed every 12 weeks as required. Specific TT (ie ipilimumab plus nivolumab) may require a different time to tumor assessment. The first tumor assessments with ipilimumab plus nivolumab should be performed after the first 4 administration of the combination ipililumab and nivolumab and according to iRECIST.

.

1st PROGRESSION DISEASE OR UNACCEPTABLE TOXICITY

At the time of the first progression of disease or unacceptable toxicity :

- blood sample will be collected to evaluate the molecular profile of the cancer on circulating DNA fragments (i.e. using FoundationOne Liquid CDx test).
- Serum sample for immuno-monitoring should be collected and stored at the site until the shipment to the central laboratory.

- Study treatment (SoC or TT) will be interrupted waiting for the evaluations for the Rescue/Switched Phase. If the patient's clinical conditions are rapidly worsening, the researcher can switch directly to rescue therapy without waiting for the results of the FoundationOne Liquid CDx test, notifying the coordinanting center and Sponsor in writing.
  1. Tailored Therapy (TT Arm): unacceptable toxicity is defined as:
     A. a toxicity that requires permanent discontinuation of the Tailored Therapy (TT) according to Appendix 2 of the protocol.
     B. a toxicity that requires an interruption of more than 4 (four) weeks for Target Therapy and for the Immuno Therapy please refer to the SmPC and Appendix 2
  2. Standard of Care (SoC): unacceptable toxicity is defined as a toxicity that requires permanent discontinuation of the therapy according to SmPC

RESCUE/SWITCHED THERAPY PHASE

Each cycle of visits in rescue/switched therapy follows the same scheme described for C2D1, C3D1, C4D1. The cycles will continue until the second Progression of Disease or unacceptable toxicity that signs the end of study for the patient. Therefore the assessments that will be conducted on the patients continuing in the Rescue/Switched Therapy Phase are the same indicate into treatment period paragraph.

All randomised patients in the SoC arm who switch to the TT arm must sign part 2 of the informed consent before starting the new treatment.

If the patient's clinical conditions are rapidly worsening, the researcher can switch directly to rescue therapy without waiting for the results of the FoundationOne Liquid CDx test, notifying the coordinanting center and Sponsor in writing.

NB: To be noticed that the Rescue Therapy Phase is part of the main study and the patients who shift from the standard therapy (SoC) to the tailored therapy (at the time of the first documented progression) will be part of the population on which the assessments of the final ORR will be made.

UNSCHEDULED VISITS

During the treatment (i.e. Treatment Phase and Rescue/Switched Therapy Phase), extra visits could be scheduled at the clinician's discretion and registered in eCRF.

PD2 AND/OR END OF STUDY VISIT

The End of Study Visit will be performed by patients who withdraw from the study for one of the following reasons: by the second progression disease (PD2), by clinical judgment of the physician or by request of the patient.

In both cases following procedures need to be performed as follows:

- ECOG performance status
- Adverse Events (AEs) including SAEs should be documented according to NCI-CTCAE, last updated version at each visit or when AEs occur
- Concomitant medications and cancer-related surgery or procedures should be documented at each visit; including prescription, over-the-counter, and herbal/homeopathic remedies and/or therapies, as well as any cancer-related diagnostic, therapeutic, or surgical procedures performed
- QoL EORTC QLQ-C30 must be completed by patients at the time of tumor assessment and in any case within an interval of no more than 3 days

If the diagnostic procedures, QoL are performed in the 7 days prior to the end of study visit, they will not need to be repeated.

N.B: In case of withdrawal, death or lost to follow the last visit performed has to be considered as end of study visit.

SURVIVAL FOLLOW UP: EVERY 4 MONTHS AFTER 2nd PD AND END OF STUDY VISIT

After progressive disease (PD2) and END OF STUDY, a clinical visit or a phone contact has to be scheduled every 4 months to evaluate the Overall Survival (OS).
Survival information will be collected until death, loss to follow-up, withdrawal of consent.

FOLLOW UP PROCEDURES

Post–study treatment cancer-related medical or surgical procedures and therapies. Any cancer-related diagnostic, therapeutic, or surgical procedure, or cancer therapy administered during the follow-up period, should be recorded including the dates and description of the procedure(s) or therapies, and any clinical findings.
Requests to withdraw consent must be documented in the source documents and signed by the investigator.
Immediately prior to the data cutoffs for the final PFS analysis and final OS analysis, the investigative sites will contact every patient that is alive to confirm current survival status. (The study Sponsors will notify all investigators of the timing of this survival data sweep).

TREATMENT DISCONTINUATION VISIT

Details relevant to the procedures requested at the Treatment Discontinuation Visit are summarized in Table 2, reported here below:

TABLE 2. TREATMENT DISCONTINUATION VISIT PROCEDURES

	**Treatment Discontinuation**
Pregnancy test	**X**
ECOG performance status	X
Physical examination and vital signs	X
Routine blood exams	X
ECG / ECHO	X
Tumor assessment (through radiographic determination)	X
Bone scan if clinically indicated	X
Adverse event	X
Concomitant treatment	X
QoL (EORTC QLQ-C30)	X
Post–study treatment cancer-related medical or surgical procedures and therapies	X
Survival information (collected via telephone)	X

10 EFFICACY EVALUATION

10.1 EFFICACY PRIMARY ENDPOINTS

The primary endpoint is **Overall Response Rate (ORR**) of the Treatment at choice of physicians, according to Standard of Care (SoC) or of the Tailored Treatment (TT).

ORR is defined as proportion of patients with reduction in tumor burden of a CR or PR.

10.2 EFFICACY SECONDARY ENDPOINTS

The secondary efficacy variables are as follows:

Progression free survival (PFS): PFS the time from randomization to the first documented radiographical progressive disease, using current RECIST (Therasse et al. 2000 [40]), or irRC (Hodi et al. 2016 [37]), or death from any cause, whichever occurs first. Carcinomatous meningitis diagnosed by cytological evaluation of cerebral spinal fluid will also define progressive disease. Medical photography will also be allowed to monitor chest wall recurrences of subcutaneous lesions.

Overall survival (OS): is defined as the time from randomization to death from any cause. Data for patients with no record of death will be censored at the last date they were known to be alive. The analysis of OS will follow the same methodology as the primary endpoint.

Time to treatment failure (TTF): Time from randomization to discontinuation of treatment for any reason, including disease progression, treatment toxicity, and death.

Time to next treatment (TTNT): Time from end of primary treatment to institution of next therapy.

QoLs outcomes of patients included in the two arms of the study.

Biomarker analysis: The relationship between molecular- immunological markers and efficacy outcomes will be evaluated. Details will be reported in the Statistical Analysis Plan.

Concordance between **molecular profile on tumor tissue and ctDNA.**

The immune fitness in the two treatment arm**.**

The association between the **molecular evaluation and gene expression profiling.**

10.3 FOUNDATION ONE CDX

Patients will be qualified for the study according to the results of the analysis performed on tissue samples collected at screening and analyzed by a central laboratory . Historical samples will be considered for the study if collected within 3 months from the ICF signature of the patient. Samples older than 3 months, with a maximum timeframe of 6 months, and collected before progression of disease after the last treatment administred will be considered upon clinical judgement of the Investigator, after confirmation by the coordinating site or MTB. Samples obtained from a biospy of a metastatic lesion in progression after the last treatment administred represent the optimal tissue sample for genomic testing.

In patients with glioblastomas and high grade malignant gliomas can be enrolled with the historical tissue samples considering the difficulties related to repeat the biopsy on CNS

In case of referral patients, if a tumor tissue sample analyses performed according to the Foundation Medicine technic will be provided as part of the medical history, it will not be needed to repeat neither the biopsy nor the relevant determination.

Genomic evaluation will be performed using the Foundation One CDx service and FoundationOne Liquid CDx service. FoundationOne (with update of 324 gene, reflecting CDx panel) is a next generation sequencing based in vitro diagnostic device for detection of substitutions, insertion and deletion alterations (indels), and copy number alterations (CNAs) in 324 genes and select gene rearrangements, as well as genomic signatures including microsatellite instability (MSI) and tumor mutational burden (TMB) using DNA isolated from formalin-fixed paraffin embedded (FFPE) tumor tissue specimens.

FoundationOne Liquid CDx is Foundation Medicine's best-in-class, blood-based circulating tumor DNA assay, designed to interrogate the most druggable cancer-related genes. FoundationOne ®Liquid CDx has been updated to interrogate the same number of cancer-related genes as the FoundationOne® CDx.

If a relevant alteration is found in any one of the genes on the current gene list (see table reported below), the report will identify the gene and alteration and will provide an interpretation that is specific to the patient's tumor.

Reports will include also tumor mutational burden (TMB) and microsatellite instability (MSI) status—biomarkers that may help predict response to checkpoint inhibitors.

Current Gene List[2]

Genes with full coding exonic regions included in FoundationOne®CDx for the detection of substitutions, insertion-deletions (indels), and copy-number alterations (CNAs).

ABL1	*ACVR1B*	*AKT1*	*AKT2*	*AKT3*	*ALK*	*ALOX12B*	*AMER1 (FAM123B)*	*APC*
AR	*ARAF*	*ARFRP1*	*ARID1A*	*ASXL1*	*ATM*	*ATR*	*ATRX*	*AURKA*
AURKB	*AXIN1*	*AXL*	*BAP1*	*BARD1*	*BCL2*	*BCL2L1*	*BCL2L2*	*BCL6*
BCOR	*BCORL1*	*BRAF*	*BRCA1*	*BRCA2*	*BRD4*	*BRIP1*	*BTG1*	*BTG2*
BTK	*C11orf30 (EMSY)*	*CALR*	*CARD11*	*CASP8*	*CBFB*	*CBL*	*CCND1*	*CCND2*
CCND3	*CCNE1*	*CD22*	*CD274 (PD-L1)*	*CD70*	*CD79A*	*CD79B*	*CDC73*	*CDH1*
CDK12	*CDK4*	*CDK6*	*CDK8*	*CDKN1A*	*CDKN1B*	*CDKN2A*	*CDKN2B*	*CDKN2C*
CEBPA	*CHEK1*	*CHEK2*	*CIC*	*CREBBP*	*CRKL*	*CSF1R*	*CSF3R*	*CTCF*
CTNNA1	*CTNNB1*	*CUL3*	*CUL4A*	*CXCR4*	*CYP17A1*	*DAXX*	*DDR1*	*DDR2*
DIS3	*DNMT3A*	*DOT1L*	*EED*	*EGFR*	*EP300*	*EPHA3*	*EPHB1*	*EPHB4*
ERBB2	*ERBB3*	*ERBB4*	*ERCC4*	*ERG*	*ERRFI1*	*ESR1*	*EZH2*	*FAM46C*
FANCA	*FANCC*	*FANCG*	*FANCL*	*FAS*	*FBXW7*	*FGF10*	*FGF12*	*FGF14*
FGF19	*FGF23*	*FGF3*	*FGF4*	*FGF6*	*FGFR1*	*FGFR2*	*FGFR3*	*FGFR4*
FH	*FLCN*	*FLT1*	*FLT3*	*FOXL2*	*FUBP1*	*GABRA6*	*GATA3*	*GATA4*
GATA6	*GID4 (C17orf39)*	*GNA11*	*GNA13*	*GNAQ*	*GNAS*	*GRM3*	*GSK3B*	*H3F3A*
HDAC1	*HGF*	*HNF1A*	*HRAS*	*HSD3B1*	*ID3*	*IDH1*	*IDH2*	*IGF1R*
IKBKE	*IKZF1*	*INPP4B*	*IRF2*	*IRF4*	*IRS2*	*JAK1*	*JAK2*	*JAK3*
JUN	*KDM5A*	*KDM5C*	*KDM6A*	*KDR*	*KEAP1*	*KEL*	*KIT*	*KLHL6*
KMT2A (MLL)	*KMT2D (MLL2)*	*KRAS*	*LTK*	*LYN*	*MAF*	*MAP2K1 (MEK1)*	*MAP2K2 (MEK2)*	*MAP2K4*
MAP3K1	*MAP3K13*	*MAPK1*	*MCL1*	*MDM2*	*MDM4*	*MED12*	*MEF2B*	*MEN1*
MERTK	*MET*	*MITF*	*MKNK1*	*MLH1*	*MPL*	*MRE11A*	*MSH2*	*MSH3*
MSH6	*MST1R*	*MTAP*	*MTOR*	*MUTYH*	*MYC*	*MYCL (MYCL1)*	*MYCN*	*MYD88*
NBN	*NF1*	*NF2*	*NFE2L2*	*NFKBIA*	*NKX2-1*	*NOTCH1*	*NOTCH2*	*NOTCH3*
NPM1	*NRAS*	*NT5C2*	*NTRK1*	*NTRK2*	*NTRK3*	*P2RY8*	*PALB2*	*PARK2*
PARP1	*PARP2*	*PARP3*	*PAX5*	*PBRM1*	*PDCD1 (PD-1)*	*PDCD1LG2 (PD-L2)*		*PDGFRA*
PDGFRB	*PDK1*	*PIK3C2B*	*PIK3C2G*	*PIK3CA*	*PIK3CB*	*PIK3R1*	*PIM1*	*PMS2*
POLD1	*POLE*	*PPARG*	*PPP2R1A*	*PPP2R2A*	*PRDM1*	*PRKAR1A*	*PRKCI*	*PTCH1*
PTEN	*PTPN11*	*PTPRO*	*QKI*	*RAC1*	*RAD21*	*RAD51*	*RAD51B*	*RAD51C*
RAD51D	*RAD52*	*RAD54L*	*RAF1*	*RARA*	*RB1*	*RBM10*	*REL*	*RET*
RICTOR	*RNF43*	*ROS1*	*RPTOR*	*SDHA*	*SDHB*	*SDHC*	*SDHD*	*SETD2*
SF3B1	*SGK1*	*SMAD2*	*SMAD4*	*SMARCA4*	*SMARCB1*	*SMO*	*SNCAIP*	*SOCS1*
SOX2	*SOX9*	*SPEN*	*SPOP*	*SRC*	*STAG2*	*STAT3*	*STK11*	*SUFU*
SYK	*TBX3*	*TEK*	*TET2*	*TGFBR2*	*TIPARP*	*TNFAIP3*	*TNFRSF14*	*TP53*
TSC1	*TSC2*	*TYRO3*	*U2AF1*	*VEGFA*	*VHL*	*WHSC1 (MMSET)*	*WHSC1L1*	*WT1*
XPO1	*XRCC2*	*ZNF217*	*ZNF703*					

Select Rearrangements[2,3]

Genes with select intronic regions for the detection of gene rearrangements, one gene with a promoter region and one non-coding RNA gene.

ALK	*BCL2*	*BCR*	*BRAF*	*BRCA1*	*BRCA2*	*CD74*	*EGFR*	*ETV4*
ETV5	*ETV6*	*EWSR1*	*EZR*	*FGFR1*	*FGFR2*	*FGFR3*	*KIT*	*KMT2A (MLL)*
MSH2	*MYB*	*MYC*	*NOTCH2*	*NTRK1*	*NTRK2*	*NUTM1*	*PDGFRA*	*RAF1*
RARA	*RET*	*ROS1*	*RSPO2*	*SDC4*	*SLC34A2*	*TERC**	*TERT (promoter only)***	
TMPRSS2								

*TERC is non-coding RNA gene.
**TERT is gene with promoter region.

Tumor tissue and biomarker analyses. After signing an informed consent and before randomization into the study, patients must undergo to the assessment of the molecular abnormalities (not only those that are disease-specific), which can be modulated with specific therapeutic intervention. The relevant analysis will be performed by Foundation One.

The diagnosis may be made on the biopsy of a metastatic site if primary tumor is not available. Historical samples will be considered for the study if collected within 3 months from the ICF signature of the patient. Tissue samples should be collected after progression of disease from the last treatment. Samples older than 3 months, with a maximum

timeframe of 6 months, and collected before progression of disease after the last treatment administered will be considered upon clinical judgement of the Investigator, after confirmation by the coordinating site or MTB. Samples obtained from a biospy of a metastatic lesion in progression after the last treatment administered represent the optimal tissue sample for genomic testing. In patients with glioblastomas and high grade malignant gliomas can be enrolled with the historical tissue samples considering the difficulties related to repeat the biopsy on CNS

It is highly recommended that tumor blocks are sent to the central laboratory; however, if this is not possible, 11 unstained and freshly cut slides per tumor specimen will be submitted. Blocks will be returned to the originating institution; slides sent to the central laboratory can be returned only if not used. In any case, specific instructions on the handling of the samples for centralized analysis will be provided to each site with a specific Investigator's Manual.

ctDNA analysis. After signing an informed consent (before randomization into the study) and at progression of disease patients must undergo to the assessment of the molecular abnormalities (not only those that are disease-specific), which can be modulated with specific therapeutic intervention. The analysis will be performed by FoundationOne Liquid CDx on blood sample in the central laboratory.
MTB will evaluate the results of both tissue and liquid genomic test in order to assign the TT to a patient.

10.4 NANOSTRING

Description of the equipment
The nCounter Analysis System represents the first and unique platform able to carry out the study up to 800 targets simultaneously for the analysis of gene expression (mRNA and miRNA), Copy Number Variation (DNA), lncRNA, fusion genes, single cells gene expression, proteins, in a fully automatic manner and without the need to use PCR or other enzymatic steps to amplify molecules of interest. This avoids the drawbacks normally resulting from the use of enzyme systems, such as the introduction of bias, creation of errors in the sequences of interest, enzymatic reaction efficiency and molecules counting comparability between different samples.
Description of the methodology.
The patented technology used in NanoString analysis system nCounter is based on color-coded molecular barcodes that can hybridize directly to many different types of target molecules and subsequent spotting on a solid phase, ready to be count. The automated workflow is highly simplified with a total hands of time of 15 minutes, maximizing the performance of the system and minimizing the possibility of errors.

nCounter system consists of two parts:

- nCounter Prep Station: an automatic instrument for the purification of Overall target-probe molecule and subsequent immobilization in a dedicated support for the reading.
- nCounter Digital Analyzer: an automatic scanner used to count the number of target differentiated on the base of the probe unique to each studied gene.

NanoString Technologies provides life science tools for translational research and molecular diagnostic products. The company's nCounter Analysis System has been employed in life sciences research since it was first introduced in 2008 and has been cited in more than 1,500 peer-reviewed publications. Nanostring Techology offers a cost-effective way to easily profile the expression of hundreds of genes, proteins, miRNAs, copy number variationsand SNV facilitating a wide variety of basic research and translational medicine applications, including biomarkers discovery and validation. The company's technology is also being used in diagnostics. The Prosigna® Breast Cancer Prognostic Gene Signature Assay together with the nCounter Dx Analysis System is used as a prognostic indicator for distant recurrence of breast cancer. Prosigna test is a good example of the ranslational potentiality of nCounter system. This using a signature of 50 genes

(PAM 50) and a validated algorithm that include also clinical features of breast cancer (size of tumor and nodal status), to provide molecular subtypes and risk of recurrence. In addition, the company is collaborating with multiple biopharmaceutical companies in the development of companion diagnostic tests for various cancer therapies including Immunoterapy, helping to realize the promise of precision oncology.

All screened patients (expected n = 1280) will have tissue samples profiled using the Nanostring IO360.

10.5 LOCAL STUDY SITE LABORATORY ASSESSMENTS

All local laboratory of the study site sample collection and testing will be scheduled as indicated in Table 1. Additional assessments may be performed as clinically indicated. Normal ranges for the local laboratory of the study site parameters must be supplied to the study Sponsors before the study starts.
The following tests will be performed at a local laboratory of the study site:

- Hematology. Hemoglobin, hematocrit, platelet count, RBC, WBC with differential (neutrophils, lymphocytes, monocytes, eosinophils, basophils and other cells). Additional tests may be performed as per the institution's standard practice. Testing will be performed at screening at least before the start of every drugs administration, at the treatment discontinuation visit, and when clinically indicated
- Blood chemistry. Na+, K+, bicarbonate, Cl–, Urea, Ca++, uric acid, total protein, albumin, alkaline phosphatase, ALT (SGPT), AST (SGOT), GGT, LDH, total bilirubin, creatinine, non-fasting blood glucose. Additional tests may be performed as per institution's standard practice. Testing will be performed at screening, at least before the start of every drugs administration, at the treatment discontinuation visit, and when clinically indicated
- Coagulation. All patients will have INR and aPTT testing at baseline. Patients on therapeutic doses of anti-coagulants should have INR and aPTT measurements repeated during the study, at least before the start of every cycle, and when clinically indicated
- Pregnancy Test. A serum β-HCG test should be performed for all women of childbearing potential and for all women < 1 years after the onset of menopause. Testing should be performed at the local laboratory of the study site within 72 hours prior to each administration of study medication (i.e. at Day 21 of each cycle of treatment). For all other women, documentation must be present in medical history confirming that the patient is not of childbearing potential
- Specific IMPs blood test or diagnostic procedures: Other blood test (or diagnostic procedures) specifically required for the IMPs administered to the patient in TT arm have to be performed according to details the specific section of the protocol (Appendix 2)
- HIV/HCV/HBV test if not historical data are available.

The relevant laboratory assessments should be available prior to each administration of study treatment for dose modification or delay requirements.

NOTE: Hematology, blood chemistry, coagulation and serum β-HCG tests are valid also as part of screening exams if they have been performed within 7 days of randomization and therefore during the screening period. In general, if screening laboratory assessments are performed within 7 days prior to study treatment start, they will not need to be repeated.
Any abnormalities that are discovered during patient assessment should be further investigated where clinically indicated, in order to ensure that patients suitable to be included in the study and to receive study medication.

10.6 TUMOR ASSESSMENTS

The current version of the RECIST Criteria (unidimensional tumor measurement) will be used to evaluate response and assess progressive disease. The immune related Response Criteria (irRC) will be used in order to assess the tumor progression for patients treated with immune checkpoint inhibitors.

The minimum screening examinations should include:

- CT or MRI scan of the chest and abdomen (including liver, spleen, and adrenals)
- CT or MRI scan of the brain and/or spine where there is clinical suspicion of CNS metastases
- An isotope bone scan (with bone X-ray[s] as necessary) at baseline. It should be repeated in the event of clinical suspicion of progression of existing bone lesions and/or the appearance of new bone lesions
- Medical photography to monitor chest wall recurrences (i.e., subcutaneous skin lesions)
- MRI or CT scans that were performed before a patient signed consent to take part in the study may be used to provide baseline tumor status as long as they were performed within 45 days prior to the start of treatment, at the same hospital, with the same technique or machine, and preferably by the same individual as those for tumor assessments during the study. This should be documented in the study files at the site
- PET scan could be performed, if clinically indicated. For PET response evaluation PERCIST criteria will be applied.

The same assessment technique must be used throughout the study for evaluating a particular lesion (e.g., if a CT scan is used to assess metastatic lung lesions at baseline then a CT scan must be used at all subsequent tumor assessments to assess metastatic lung lesions). Preferibly, the same investigator should assess all tumor responses for each patient.

For patients with multiple measurable lesions, a maximum of five lesions per organ and 10 lesions in total that are representative of all involved organs should be designated as target lesions and recorded and measured at screening.

All other lesions should be identified as non-target lesions and should be recorded first at screening. Measurements of these lesions are not required, but the presence or absence of each should be noted throughout the treatment period and follow-up, if applicable, until confirmed evidence of progressive disease. Tumor lesions that are situated within a field of previous irradiation can be considered measurable if these lesions have shown clinical evidence of progression and can be reproducibly measured over time.

Patients who have metastatic disease that is confined to the bone are not eligible for response evaluation but will be included in the ORR analysis if the criteria for progressive disease is satisfied (i.e. new bone lesions after treatment initiation).

Tumor assessments will be performed every 12 weeks after the date of randomization. Specific TT (ie ipilimumab plus nivolumab) may require a different time to tumor assessment. The first tumor assessments with ipilimumab plus nivolumab should be performed after the first 4 administration of ipilimumab.

If a tumor assessment must be performed early or late, subsequent assessments should be conducted according to the original schedule of every 12 weeks from the date of randomization.

In case of Stable Disease (SD), follow-up assessments must have met the RECIST or irRC SD criteria at least once after study entry at a minimum interval of 6 weeks.

In cases where there is suspicion of progression before the next scheduled assessment, an unscheduled assessment is to be performed. The reason for the unscheduled assessment will be first documented in the patient's medical records and then reported on the eCRF.

For patients who discontinue study treatment for reasons *other than* death or progression events, efforts should be made to continue to perform scheduled tumor assessments every 12 weeks until patient death or progressive disease. After the cutoff date for the final PFS analysis, tumor assessments will no longer be collected.

10.7 IMMUNO-MONITORING

Samples for immuno-monitoring evaluation will be collected at baseline (v1), at the first cycle of treatment (V2), at the first lesion evaluation and in case of PD.

a. A*nalysis performed on serum samples*

Serum will be analysed for the presence of rheumatoid factor and for cytokines/chemokines relevant during anticancer immune response/tumor progression and for the presence of molecules released by cells during an immunogenic cell death (ATP and HMGB1). Cytokines and chemokines analysed are: IFNa, IFNg, IL1a, IL1b, IL4, IL6, IL8, IL10, IL12p70, TNFa. VEGF, TGFb .

Checkpoint inhibitors BTLA, CD27 CD28, CD80, CD137, CTLA4, GITR, HVEM, IDO, LAG3, PD1, PDL1, PDL2, TIM3 will be also assayed (using the Immuno-Oncology checkpoint 14-Plex Human ProcartaPlex Panel1).

b. *Immuno monitoring*
The immunological fitness of cancer patients will be studied) by flow cytometry, evaluating:

1. CD4 and CD8 T cell subpopulations following the expression of CCR7 and CD45 and inhibitory receptors, PD-1, 2B4, CTLA-4, LAG-3, Tim-3, correlated with exhausted T cells phenotype and for the expression of CD137, marker of T cell survival and activation.
2. Th17: Th17 cells of the CD4 T cell subset produce IL17 to drive antitumor immune responses by recruiting immune cells into tumours (CD4+, CCR6+, CD161+ and production of IL-17).
3. Regulatory T cells: Tregs represent a CD4+CD25+ T cell subset able to suppress the immune response producing IL10 and TGFβ. Different Treg subpopulations will be evaluated according to the expression of FOXP3 and CD45RA markers (resting Treg: CD45RA+FOXP3low; activated Treg: CD45RA-FOXP3high; not suppressive Treg: CD45RA-FOXP3low) (Miyara M, Immunity 2009).
4. Natural killers: NK cells will be evaluated through the expression of CD3 and CD56 markers and activation status and cytotoxic ability monitored by expression of CD69, CD107a and Granzyme B.
   In selected patients, due to the limited amount of blood, other investigational experiments will be performed to assess specific parameters.
5. *T cells rescue* from exhaustion in vitro: to investigate whether blocking of inhibitory receptors restores the proliferation of exhausted T cells in vitro, immunoselected T cells will be labelled with CFSE and cultured for 4 days with or without an anti-PD1 antibody in presence of anti-CD3 and anti-CD28 antibodies or PHA. The proliferation capacity of T cells will be analyzed by cytofluorimetry. Results of this analysis performed on PBMC at different times from initial dose can give indications on possible indicators of re-treatment/re-challenge.
6. *Detection of cancer specific CD8 T cells* (determination of pre-existing and induced immunity): Twenty HLA-A2+ patients will be monitored for the detection of antigen-specific CD8+ T cells before and at the end of treatments. The presence of circulating tumor antigen-specific CD8+ T cells will be analysed by flow cytometry using Dextramers (Immudex).

The analysis will be performed at the
Cell Therapy Unit and Laboratory of Tumor Immunology, directed by Prof.Marianna Nuti , Policlinico Umberto I - Viale Regina Elena 324, 00161 Rome, ITALY

11 SAFETY REPORTING OF ADVERSE EVENTS

11.1 SAFETY AND TOLERABILITY EVALUATION

Safety of the treatment will be evaluated as follows:

- Incidence and severity of AEs and SAEs.
- Laboratory test abnormalities.

- Vital Signs.
- Physical Examination.
- ECG and ECHO.
- Concomitant Medications.

11.2 ASSESSMENT OF SAFETY

11.2.1 Specification of Safety Variable

Safety assessments will consist of monitoring and reporting adverse events (AEs), serious adverse events (SAEs), adverse events of special interest (AESI), special situations and pregnancy throughout the protocol.

11.2.2 Adverse Events

DEFINITIONS
Adverse Event (AE)
Any untoward medical occurrence in a subject or clinical-trial subject administered a medicinal product and which does not necessarily have to have a causal relationship with this treatment. An adverse event can therefore be any unfavourable and unintended sign (e.g. an abnormal laboratory finding), symptom, or disease temporally associated with the use of a medicinal product, whether or not considered related to the medicinal product.

Adverse Reaction (AR)
All untoward and unintended responses to an investigational medicinal product related to any dose administered are considered adverse reactions.
AEs have to be classified by the Investigator as "Serious" or "Non Serious", according to the following definitions:

Serious Adverse Event (SAE)
Serious adverse event/reaction means an adverse event/reaction which:

- results in death,
- is life-threatening,
- requires in-patient hospitalization or prolongation of existing hospitalization,
- results in persistent or significant disability or incapacity,
- is a congenital anomaly/birth defect
- important medical event

Death
In case “death” is the only information available at the time of the SAE notification, it has to be reported as such. Nevertheless, the cause of death should be further investigated since death has to be considered as an outcome and not as an event.
Deaths are always SAEs. For fatal cases, the Principal Investigator (and/or the designees) will have to fill-in an “AE” CRF form, an “SAE” form and immediately notify by the Investigator them to the relevant EC.

Life-threatening
Life threatening in this context refers to an event/reaction in which the subject was at risk of death at the time of the event/reaction; it does not refer to an event/reaction that hypothetically might have caused death if more severe.

Hospitalization
The events that require a hospitalization for one of the reasons reported below are not to be considered to be SAEs:

hospitalization planned before entry to the clinical study which is part of the normal treatment or monitoring of the studied indication and not associated with any deterioration in condition;
hospitalization for routine treatment or monitoring of the studied indication, not associated with any deterioration in condition;
hospitalization for treatment, which was elective or pre-planned, for a pre-existing condition that is unrelated to the indication under study and did not worsen.

Important Medical Event (IME)
Any event that might not be immediately life threatening or result in death or hospitalization but might jeopardize the subject or might require intervention to prevent one of the other outcomes listed above.

Non Serious Adverse Event (NSAE)
Event that does not meet any of the criteria defining SAEs.
Causality assignment
The definition of "Suspected Adverse Reaction" implies the existence of a reasonable possibility of correlation between the event observed and the medicinal product tested. This means that is possible to identify evidences (data) which suggest the existence of such correlation

Unexpected Adverse Reaction (UAR)
An unexpected adverse reaction is an adverse reaction, the nature or severity of which is not consistent with the applicable Product Information (e. g. Investigator's Brochure for an unauthorized investigational product or the Summary of Product Characteristics for an authorized product).

Adverse Events of Special Interest (AESI)
An Adverse Event of Special Interest is defined as any AE (serious or non-serious) that is of scientific and medical concern specific to the study treatment, for which ongoing monitoring and rapid communication to the Sponsor is required.

11.3 MONITORING AND RECORDING OF AEs

The Investigator is responsible for the managing of the events meeting the definition and criteria of an adverse event (AE) or a serious adverse event (SAE), as provided in this protocol.
All adverse events occurring between the date of informed consent signature and 3 months after last drug administration will be recorded in the CRF (and in the SAE form, if applicable). Each subject will be monitored regularly by the Investigator and study personnel for adverse events occurring throughout the study.
Subjects will be monitored throughout the study for adverse events to the study treatment and/or procedures.
AEs will be documented and collected on an ongoing basis during the treatment period and the relevant follow-up period.
In case of AE due to a toxicity that requires interruption of the experimental treatment (TT Arm), the maximum period of interruption admitted is

- 4 (four) weeks for Targeted Therapy
- for Immunotherapy, please refer to the SmPC, IB and Appendix 2

The principal Investigator and/or the designees will instruct the subject on how to communicate any AEs occurred. Medical events fulfilling one or more criteria of seriousness and occurring after the informed consent has been signed, must be immediately (within maximum 24 hours) reported to the Sponsor (or delegate) using a SAE form. The Investigator has to follow all AEs until a final outcome is determined.

SAEs still present at the end of the study period and for the subsequent 3 months must be followed until the final outcome is determined.

Any subject who experiences an adverse event (AE) (whether serious or non-serious) or has a clinically significant abnormal laboratory test value(s) will be evaluated by the Investigator and will be treated and/or followed up, even after the study end, until the symptoms or value(s) return to normal, acceptable levels or chronicity, as judged by the Investigator.

Clinical Laboratory Abnormalities and Other Abnormal Assessments as AEs and SAEs

Abnormal laboratory findings (e.g. clinical chemistry, haematology, urinalysis) or other abnormal assessments (e.g. ECG, vital signs) that are judged by the Principal Investigator (and/or designees) as clinically significant will be recorded as AEs or SAEs if they meet the definition of an AE or SAE, as previously defined.

SERIOUS ADVERSE EVENT REPORTING

Once the Principal Investigator (and/or designees) becomes aware that a SAE has occurred in a study subject, he/she will immediately notify the Sponsor by contacting the reference person indicated into the specific section of Safety Management Plan.

The SAE form must be completed as thoroughly as possible with all available details of the event, signed by the Principal Investigator (or appropriately qualified designee), and mailed/faxed to the above mentioned contact within 24 hours from the first information received about the event. The Investigator will always provide an assessment of causality between the experimental drug/event.

Please refer to the definitions relevant to the causality assignment reported in the WHO-UMC guidelines.

Should a NSAE become serious, the Principal Investigator (or appropriately qualified designee) will then follow the same reporting procedures as for SAEs, by making prompt notification to the above mentioned contact .

The applicable follow-up information should be managed with the same modality and timeframe applied for the initial notification (a new SAE form should be completed and transmitted within 24 hours from the first information received about the event).

Progression of the investigational malignancy should NOT be reported as an adverse event if it is clearly related to suspected progression of the underlying tumour as defined by the RECIST criteria. Hospitalization due solely to progression of the underlying malignancy should also NOT be reported as SAE. Clinical symptoms of progression may be reported as adverse events if the symptom cannot be determined to be due solely to progression of the underlying malignancy or cannot be traced to the expected progression of the disease under study. In any case, if there is any doubt that an AE is due solely to the study disease, this should be reported as an AE or SAE.

It is mandatory for the Principal Investigator (and/or designees) to notify fatal events to his/her EC.

The management of the AESI is detailed in the relevant Safety Management Plan of this Protocol, in which are described all the AESI pertaining the products contained in the study therapy, according to what reported in the current Reference Safety Information documentation (i.e.: SmPCs, IBs).

AESIs should be reported on SAE Report Forms whether serious or not, with the same modality and timelines applied to the SAE transmission defined above.

Other reports of special situations (AESI) will be sent to the involved Company according to the schedule predefined in the respective Safety Agreement.

PREGNANCY CASES

Although pregnancy is an exclusion criterion and women of childbearing age are excluded from the study, cases of pregnancy may occur. The Investigator must report any possible pregnancy occurring in a patient or in the partner of a male patient enrolled in the study preferably within 24 hours of learning of its occurrence.

The pregnancy will be followed up till the final outcome.

Details on the management of pregnancy cases are provided in the relevant section of the Safety Management Plan, where specific company requirements will be detailed making reference to the relevant Safety Agreement.

11.4 MANAGEMENT OF SPECIAL SITUATION REPORTS

11.4.1 Special situation reports

The Sponsor will be responsible for collecting all adverse event reports (Adverse Events, AEs) and adverse events of special interest (AESI) listed in the relevant Safey Management Plan. In addition the following "special situations" must be collected, even in the absence of an AE:

- Data related to the Product usage during pregnancy or breastfeeding
- Data related to overdose, abuse, off-label use, misuse, inadvertent/erroneous administration, medication error or occupational exposure, with or without association with an AE/SAE unless otherwise specified in the protocol
- Data related to a suspected transmission of an infectious agent via a medicinal product

In addition, reasonable attempts should made to obtain and submit the age or age group of the patient, in order to be able to identify potential safety signals specific to a particular population.

11.4.2 Monitoring of Safety information

The Sponsor will keep track of all the AE reports defined by the protocol and also of the special situations emerging from the Study and relating to Companies' Products.
The Sponsor will also keep track of all serious adverse drug reactions (Serious Adverse Drug Reaction, SADR) associated with other non-Company investigational drugs (investigational medicinal product, IMP), as defined in the Protocol / Safety Management Plan (and according to what pre-defined in each Safety Agreement).

11.4.3 Exchange of special situation reports (AESI)

Serious adverse events (SAEs), pregnancy reports and AEs of special interest (AESIs) (detailed in the relevant Safety Management Plan), for patients who have been exposed to at least one of the study drugs, will be communicated to the relevant Company according to the specific Safety Agreement.

The Sponsor will comply with the tailored communication timeframes established in each Safety Agreement. Tracking of exchanged data will be kept during the course of the study and updated on an ongoing basis.

11.4.4 Case transmission verification

All individual case reports must be transmitted to the Company, by periodical list exchanging: this listing, to be sent to the Company Local Safety Unit, must contain the list of individual case reports already sent by Sponsor to the Company in the previous period (cumulative reports).
It is established that this reconciliation activity should be performed on a monthly basis or according to specific scheduled as defined in each Safety Agreement.
The periodical listing will be exchanged within seven (7) calendar days from the end of the agreed period, unless differently defined by the specific Safety Agreement.
Confirmation of receipt of the aforementioned listing by the Company will be in accordance with the deadlines agreed between the parties.

If during the reconciliation activity cases not received from the Company are found, they must be sent by the Sponsor to the Company within seven (7) calendar days of the latter's request, unless differently defined by the specific Safety Agreement.

11.4.5 Aggregate Reports

The Sponsor of the Study will be responsible for the preparation of their own Development Safety Update Report (DSUR) for the Study and for the submission of the report to the regulatory authorities and Ethics Committees of the concerned Member States, where applicable. The Sponsor agrees to share a copy of their own DSUR with each Company (if requested) as soon as reasonably possible after completion.The Company agrees to forward to the Sponsor a copy of the Company's Investigator Brochure and related updates for all the Products upon request. The Company also agrees that the Sponsor may cross-check, where relevant, the information contained therein.

11.4.6 Other Reports

The Sponsor will transmit to the Company the lists of non-serious AEs (according to predefined schedule and if requested) in the format agreed between the Parties, in order to allow adequate signal detection, which will be shared with the Sponsor in accordance with regulatory requirements.

11.4.7 Queries

The Sponsor will answer any questions relating to the safety profile emerging from the conduction of the Study.
In any case, answers to all safety questions addressed by the regulatory authorities or for publication purposes will be discussed and coordinated between the Parties. The Company should have the final decision and control over safety questions concerning the Products and therefore the Sponsor will share with the Company any information related to the safety profile and requested for safety enquires.

11.5 STUDY COMMITTEES

11.5.1 DATA MONITORING COMMITTEE

An independent Data Monitoring Committee (DMC) will be established in order to review the data data generated during the conduction of the study. The data that will be reviewed by the Committee will include (but will not be limited to) the evaluation of the progressive disease and overall tumor response, according to the primary and secondary endpoints of the protocol and will also focus on specific safety parameters, according to all the warnings reported in the SmPCs/IBs of the drugs that will be used during the conduction of the study (such as, but not limited to, cardiovascular parameters). All DMC-related details and activities will be specified in a separate DMC charter, in which the rules for the establishment, the composition, the management and contents of the meetings, the type of data that will be analyzed, the way the outcomes of each meeting will be shared, the way the board will take decisions, will be defined. The members of the Committee will be identified according to specific professional profiles, in order to ensure a full compliance with the objective of the Committee, which in any case will be totally independent from the Sponsor. In any case, the main objective of the DMC will be to follow-up on an ongoing basis on the safety profile emerging from the new therapeutic approach proposed by the present study, in order to be able to support the Sponsor in maintaining an adequate clinical management of the patients.

11.5.2 MOLECULAR TUMOR BOARD AND STEERING COMMITTEE

The Molecular Tumor Board (MTB) also acts as Steering Committee (SC). The MTB and the SC share the same members list that will be composed by opinion leaders in oncology, immunology, biology, biochemestry, statistics, bio-informatics and personalized medicine. The role of MTB is to elaborate the data of the FoundationOne report in order to identify the most effective and safe therapy or combination therapy for the individual patient.
Details on the composition of both the committees, the engagement rules for the meetings and the data evaluation, the rules for assessments dissemination will be defined in two different Charters, which will be finalized before the start of study enrollment.
The best TT treatment for the patient will be assigned by MTB based on genetic profiling of FO reports, the patients medical history and the latest data available from the scientific sources.

12 DIRECT ACCESS TO SOURCE DATA/DOCUMENTS

All records identifying the subject will be kept confidential and, to the extent permitted by the applicable laws and/or regulations, will not be made publicly available. Subject names will not be supplied to the sponsor. Only the subject number will be recorded in the eCRF, and if the subject name appears on any other document it must be obliterated before a copy of the document is supplied to the sponsor (if requested). Study findings stored on a computer will be stored in accordance with local data protection laws. The Principal Investigator or his/her delegates must allow the Regulatory Authority, the Independent Ethics Committee or the Sponsor (or its delegate) to have free access to and to conduct the relevant verification of all the original documentation of the study, including the informed consent forms signed by the subjects enrolled into the study, the relevant patient files and/or out-patient files. Those individuals who are given free access to the documentation must take every reasonable precaution to keep the identity of the subjects as reserved information, in accordance with relevant applicable legislation.

13 QUALITY CONTROL AND QUALITY ASSURANCE PROCEDURES

13.1 CASE REPORT FORM (CRF): GENERAL INFORMATION

An eCRF system Part 11 CFR compliant will be used to collect the data of the study. Pre-setting of the database will be done according to key parameters, which will guide the eCRF forms' design. Specific edit checks will rule the overall data consistency process. A validation protocol will be implemented before issuing the live system at sites' level (including a UAT procedure (User Acceptance Test).

13.2 AUDITS

The Principal Investigator should understand that it may be necessary for the Sponsor (or its delegates) to conduct one or more site audits during or after the study and agrees to allow access to all study related documentation and information and be available for discussion about the study.

13.3 STUDY MONITORING

Accredited Study Monitors delegated from the Sponsor will carry out the monitoring of the clinical study. Each eCRF will be reviewed on site or through remote Source Document Verification (rSDV) and checks will be made against source documents. The Principal Investigator or his/her delegates must ensure that the site notes will be available for direct

verification of source data. The Principal Investigator or his/her delegates agrees to allow regular visits (frequency depending on recruitment) by the study monitors of the designated Sponsor's delegate and adequate access to study personnel and documents.

13.4 INSPECTIONS

The Principal Investigator must allow Regulatory Authority (AIFA) to conduct Inspections.
The Inspection of the Regulatory Authority consists of an official review of the documents, facilities, records and any other resource considered by the Authority to be connected with the study.

14 DATA HANDLING

The data collection will be carried out at site. The data will be entered by the Investigator or a designee in a web-based Database provided by CMV-Stat. The electronic system will include the data entry pages prepared on the basis of the study flow-chart. The eCRF entries will be transcribed from source documents. Assessments for which source documents are usually available include laboratory data, medical history, physical examination, etc.. Details of the eCRF completion will be explained to the Principal Investigator and/or delegates during the Site Initiation Visit (SIV). All entries in the eCRF must be made in English language. The Principal Investigator and/or delegates must give a reasonable explanation for all missing data. Each delegate of the clinical staff needs to register for eCRF entries with an individual user name and password. The eCRFs must be updated after each visit scheduled by the clinical study. When corrections are made to entries in the eCRF by the Principal Investigator or delegates, the automated audit trail will keep track of all changes.

14.1 DATA MANAGEMENT

The data cleaning will be performed by CMV-Stat Data Management team who will provide the Investigator with the list of the detected inconsistencies on each group of data analyzed. The validation of the inconsistencies (change or acceptance) will be made by the Investigator. Before the data freezing, CMV-Stat will code the medical terms according to the following dictionaries (a Validation Coding Report will be prepared by CMV-Stat Biologist in charge and approved by the Sponsor or his delegates):

- MedDRA for the pathologies and the Adverse Events
- WHO-ATC for the drugs

At the end of the study, CMV-Stat will be in charge of the Data Base freezing. The Data Manager in charge will provide the Biostatistics Unit with the cleaned Data Base for the Statistical Analyses.
All the details relevant to the data management will be described in the Data Management Plan, which will be prepared by the Data Manager in charge of the study.

15 INTERIM ANALYSIS

An Interim Analysis will be carried-out when 20% of the randomized total patients (i.e. 76 patients), regardless of which of the four groups they belong to, have reached the final efficacy evaluation (Day 308 or early termination). As soon as the 76th patient has reached the final efficacy evaluation (including the patients who early terminated the study, if any, so that the total number of patients to be analyzed in the Interim Analysis could be greater than 76) the pre-specified analysis will be carried-out in the ITT population by an Independent Statistician. The results will be given to the Data Monitoring Committee (DMC) in terms of "go/no go" data, who will review them and provide the relevant

recommendations according to pre-specified rules (please refer to section 10.5.1). There will be no interruption in the patients enrolment during the interim analysis activities.
The method for performing this analysis will be based on the following papers: Lan et al (1982) [47], Wang et al (2002) [48] and Siu and Lan (2001) [49].
The interim analysis will be based on the computation of the so called Conditional Power (CP). The CPt is the probability, given the data observed at the time t of the interim analysis that are statistically significant at the level α (0.05 for the present studies) for the two-sided test verifying H0 versus H1, at the end of the study. Unknown parameters will be estimated with the observed data and under the H0 model.
The main assumptions are the following:
Nt=76 patients (where t = time of the interim analysis);
CPt= conditional power at time t;
At = threshold for CPt under which the trial will be stopped. That threshold is fixed on the expected result of the SoC group, i.e. 0.05 (5% of Responder patients).

The decision rules to be adopted at time t in function of the CPt value will be the following:

- If CPt<At, then stop the studies (there is a very strong evidence of futility);
- otherwise, if CPt≥At, the studies will continue under the initial conditions.

The interim analysis is intended to perform intermediate evaluation of efficacy data in order to assess the need to early terminate the study because of futility (i.e. very poor chance of success), avoiding possible unethical exposure of patients to undue treatment.

The use of the above described procedure does not inflate the nominal significance level of the study. If the study is early terminated for futility the alpha level is deflated. As a consequence, no adjustment of the significance level of the test is requested.

A more detailed document to describe the above procedure will be prepared and provided to the Data Monitoring Committee before performing the assessment (DMC charter).

16 STATISTICAL ANALYSIS

SAMPLE SIZE

Four independent experiments will be carried out within each considered cancer-site. We hypothesize that therapy based on molecular profile (TT) would result in a high overall response rate (ORR) with respect to SoC, i.e. 5% ORR of SoC in favor of 20% of TT. On the basis of that hypothesis, assuming α = 0.10 and β = 0.20 (i.e. 80% of power), and conducting a one-sided Chi-square test, 86 patients (43 patients in each arm of the four cohorts) are sufficient for detecting a 15% difference between the two arms (i.e. SoC = 5% versus TT = 20%), in terms of number of responders. According to the nature of the study (i.e. basket study), in order to evaluate the ORR on the whole sample, the four cohorts will be also considered as strata of a single cohort of 344 patients (86 patients in each strata).The ORR will be tested conducting a two sides Cochran–Mantel–Haenszel test at 5% significant level. A sensitivity analysis will also be carried out not considering the strata (i.e. Chi-square test).
In order to account for the possible influence of not evaluable patients and to safe sufficient power for the analyses of the single cohort, an additional 10% of patients will be considered. Therefore, a total of 384 patients will be enrolled. The cited four cohorts will be competitive with each other.
As above mentioned, assuming that in at least 30% of eligible patients the actionable mutations will be observed, the total number of patients to be screened is expected to be around 1280.

This sample size estimate is not appropriate for a conventional phase II trial, but it can be considered acceptable for an exploratory study, which have the purpose of obtaining preliminary information on the treatment effect without exposing a large number of patients to an experimental treatment

STATISTICAL AND ANALYTICAL METHODS

This study is a randomized, trial designed to evaluate the efficacy of Therapy according to genomic profile, identifying "driver mutations" compared to Therapy at choice of physician.

METHODS FOR THE VARIABLE ANALYSES

The analyses will be performed with SAS version 9.4. On the basis of what described above, all the analyses to be carried out will be detailed in the Statistical Analysis Plan (SAP) which will be finalized in Version 1.0 before the Data Base freezing. Version 2.0 of the SAP will be prepared after the Data Review Meeting (in which the protocol violations will be evaluated) and will include the list of the patients belonging to the defined populations and the randomization list.

All the variables will be descriptively analyzed by treatment and visit (mean, median, standard deviation, interquartile range for continuous variables, frequency distribution for categorical variables). Efficacy analysis will be applied in both the ITT and PP populations. Results from the ITT population will be considered the primary ones.

Baseline characteristics

A descriptive analysis for demographic data and baseline characteristics will be carried out in order to verify the balance among the arms and cohorts at study entry.

Efficacy Endpoints

Efficacy outcomes considered for this analysis will include PFS, overallresponse rate, TTF and TTNT. The PFS and overall response will be based on the IRF assessments.
The biomarker analyses at the time of protocol development do not take the form of testing fixed hypotheses involving specific cutoffs or other pre-specified prediction rules.
It is planned for the Statistical Analysis Plan (to be generated prior to the Data Base freezing) to use all available scientific evidence from independent studies or publications to specify testable prediction rules.

Primary Efficacy Endpoint

In accordance with the primary objective of the study, the proportion of responder patients measured at treatment end will be the primary endpoint, where a responder is defined as the patients who had a reduction in tumor burden of a CR or PR at treatment end as compared to baseline.
The Overall Response Rate will be tested in both the single cohorts and on the whole sample.
The treatment comparison, in each cohort, will be performed by the $\chi 2$ test. The test will be one-sided and conducted at the α 0.10 significance level. Only patients with measurable disease at baseline will be included in the analysis. Patients without a post-baseline tumor assessment will be considered to be non-responders
As for the whole sample, the treatment comparison will be performed by the Cochran-Mantel-Haenszel test stratified by type of cancer (four strata). The test will be two-sided and conducted at the α 0.05 significance level. A sensitivity analysis will be performed on the whole sample not considering the stratification (i.e. $\chi 2$ test).
The treatment effect will be estimated in terms of difference in response rate between the two treatments. The Mantel-Haenszel method will be applied for computing a stratified estimator and corresponding one-sided 80% Confidence Interval (CI). Support for "proof of concept" will be established if the upper limit of the CI does not cross the zero value.

The ORR will be constructed according to the specific design of the study, therefore including also the Rescue Therapy Phase data.
This means that the ORR will take into account 3 evaluations:

- The ORR estimation on the original final population (i.e 384 patients divided into the 4 groups of type of cancer)
- The ORR estimation done on the TT patients, which will include the original randomized TT patients and the patients swithed from the standard of care therapy (SoC therapy) to the TT Therapy, this latter within the Rescue Therapy Phase (patients switching upon the first documented progression)
- The ORR estimation done on the population composed by the original TT patients, the original SoC patients and the switched TT patients. This means that the total population analyzed will include the original 384 population data (as per randomization) and the additional switched TT patients.

Secondary Efficacy Endpoints

PFS based on investigator assessments. Data for patients who do not have documented progressive disease or who have not died within 18 weeks of the last tumor assessment will be censored at the time of the last investigator tumor assessment (or, if no tumor assessments are performed after the baseline visit, at the time of randomization plus 1 day). The Kaplan-Meier approach will be used to estimate median PFS for each treatment.

Overall Survival (OS): is defined as the time from randomization to death from any cause. Data for patients with no record of death will be censored at the last date they were known to be alive. The analysis of OS will follow the same methodology as the primary endpoint.

Time to treatment failure (TTF): These data will be analyzed descriptively in terms of medians, 25% and 75% percentiles, using the Kaplan-Meier method. This endpoint will be analyzed in the ITT and PP populations but the analysis on the ITT will be considered the most relevant. Patients with SD, PR, or CR at treatment discontinuation will be followed for tumor response until disease progression or start of a new anti-tumor therapy or death. Therefore, analysis of TTF will include all patients in the ITT population and will treat patients that are on progression, start a new anti-tumor therapy or die as patients reaching the endpoint (e.g. progression) and patients who are lost-to-follow-up as right censored information (censoring will be at the time of the last tumor assessment.) If patients die without documentation of disease progression, they will be considered to have had tumor progression at the time of their death unless there is sufficient documented evidence to conclude that death was not correlated with the disease progression and that no progression occurred prior to death.

Time to next treatment (TTNT): The same analysis proposed for the TTF will be performed on TTNT.

QoLs Data from the two Quality of Life (QoL) questionnaires will be descriptively analyzed. The purpose of this analysis is to obtain pilot estimates of the treatment effect on QoL for planning future studies.

Biomarker analyses. To evaluate the effect of molecular markers on efficacy outcome, efficacy outcomes will be summarized for all patients, and by treatment arm, within each subgroup determined by exploratory markers.

Concordance between molecular profile on tumor tissue and ctDNA.

The immune fitness in the two treatment arms.

The association between the molecular evaluation and gene expression profiling.

Safety Endpoints
The Safety population will be used to evaluate safety and tolerability data.

Physical examinations, ECGs, vital signs, laboratory tests, adverse events and concomitant medications will be considered.

Categorical variables (ECG normality, physical examination normality, etc.) will be analyzed with shift tables (baseline vs. final visit), while continuous variables (laboratory parameters, vital signs, etc.) by descriptive statistics.

Laboratory data will also be analyzed with shift tables (baseline vs. final visit), considering each value as being normal/abnormal with respect to the appropriate normal ranges.

Different units of measure will be converted into SI units. Afterwards, for each laboratory test, observed values will be normalized with respect to a unique reference range.

In order to facilitate identification of outliers and medical interpretation of results, normalized laboratory values will be graphically represented by means of plots of baseline vs. final assessments.

Adverse events will be coded using the MedDRA dictionary. Adverse events and all related information will be listed by patient. Descriptive statistics will be performed classifying the events by system organ class and preferred term; they will also be classified by seriousness and relationship with the study treatment and CTCAE grade.

Prior and concomitant medications will be summarized using the WHO coding system.
Prior and concurrent medical conditions will be coded using the MedDRA dictionary.
The safety analysis will take into account the toxicity profile of each IMP used in the Study. On the other hand, in order to be compliant to the current pharmacovigilance regulatory requirements, the data reported in the Reference Safety Information authorized documents (i.e.: Summary of Product Characteristics – SmPCs - of the products) and in the IBs in case of non marketd IMPs will represent the unique reference to assess expectedness of each reportable Serious Suspected Adverse Reaction.

ANALYSIS POPULATIONS

For this study, three populations will be considered:
Safety population defined as all patients who signed the Informed Consent and received the first treatment at least. This population will be used for the safety and tolerability analyses. The Safety population will also include all screening failure patients who experienced at least one documented AE.
Intention-To-Treat population (ITT) population defined as all the randomized patients who received the assigned treatment and had the first study evaluation at least. This population will be used for the efficacy analyses.
Per Protocol population (PP) population defined as all patients compliant with the study drug, having completed the study, fulfilled all the inclusion/exclusion criteria and having not used prohibited medications

17 ETHICAL ISSUE

This trial must be carried out in compliance with the protocol, designed to ensure adherence to Good Clinical Practice, as described in:

1. ICH Harmonized Tripartite Guidelines for Good Clinical Practice, 1996. Note for Guidance on Good Clinical Practice CPMP/ICH/135/95 (and any applicable update).
2. EU Directive 2001/20/EC, 2005/28/EC as well as the general principles of the REGULATION (EU) No 536/2014 OF THE EUROPEAN PARLIAMENT AND OF THE COUNCIL of 16 April 2014 on clinical trials on medicinal products for human use, and repealing Directive 2001/20/EC.
3. Declaration of Helsinki (1964, and its amendments and subsequent clarification), provided in Appendix 3.

The Principal Investigator or delegates agrees, when signing the protocol, to adhere to the instructions and procedures described in it and thereby to adhere to the principles of Good Clinical Practice that it conforms to.

17.1 ETHICAL AUTHORIZATIONS

The informed consent form and other information to the subjects issued for the purposes of the present study, should be submitted to the referent Ethics Committee and to the Regulatory Authority (AIFA for Italy).

17.2 PROTOCOL AMENDMENTS

All significant deviations from the protocol should be documented. Any changes made after data analysis has begun should be documented as such and the rationale provided. Amendments significantly affecting the scope of the investigation or the scientific quality of the study and require additional approval by the Independent Ethics Committee and by the Regulatory Authority (AIFA for Italy). If an approval is not required, the amendment must be reported to the Regulatory Authority for its information.

17.3 INFORMED CONSENT

It is the responsibility of the Principal Investigator or delegates to obtain a written informed consent from each subject. The Principal Investigator or delegates will explain the nature of the trial, its purpose, the procedures involved, the expected duration, the potential risks and benefits involved and any discomfort it may entail and provide the subject with a copy of the information sheet. The subject will be given sufficient time to consider the trial before deciding whether to participate. Each subject must be informed that participation in the trial is voluntary and that he/she may withdraw from the trial at any time and that withdrawal of consent will not affect his/her subsequent medical treatment or relationship with the treating physician. All patients are to provide written informed consent in accordance with applicable laws of the country. The patient will sign and date the informed consent form before he/she enters the study (i.e. before any study related activity). The Principal Investigator or delegates will explain the nature of the study, its purpose and the expected duration. The patient will be given sufficient time to consider the study before deciding whether to participate. Each patient must be informed that participation in the study is voluntary and that he/she may withdraw from the study at any time and that withdrawal of consent will not affect his/her medical treatment or relationship with the treating Investigator. In the case of an amendment that would directly affect the patient’s participation in the study, the patient must provide new written informed consent indicating that he/she re-consent to participate in the study

18 ADMINISTRATIVE PROCEDURES

18.1 CHANGES IN STUDY CONDUCTION OR PLANNED ANALYSIS

If it is necessary for the study protocol to be amended, the amendment should be submitted to the local Ethics Committee the Principal Investigator should submit it also to the regulatory authority before the protocol amendment is implemented. If a protocol amendment requires a change to the Informed Consent Form, then it has to be submitted for approval to the local Ethics Committee. Approval of the revised Informed Consent Form by the Ethics Committee is required before the revised form is used.

18.2 STUDY DISCONTINUATION/TERMINATION

The Sponsor has the right to close the study (or, if applicable, individual segments thereof (e.g. treatment arms; dose steps; centers) at any time, which may be due, but not limited to, the following reasons:

- If risk-benefit ratio becomes unacceptable owing to, for example
  - Safety findings from this study (i.e. SAEs, safety signal detection, etc…)
  - Results of the Data Monitoring Committee (DMC)
  - Results of parallel clinical studies
  - Results of parallel animal studies

  (on e.g. toxicity, teratogenicity, carcinogenicity or reproduction toxicity).
- If the study conduct (e.g. recruitment rate; drop-out rate; data quality; protocol compliance) does not suggest a proper completion of the trial within a reasonable time frame and a proper quality status.

The Investigator has the right to close his/her center at any time. For any of the above closures, the following applies:

- Closures should occur only after consultation between involved parties. Final decision on the closure must be in writing.
- All affected institutions (e.g. IEC(s)/IRB(s); competent authority(ies); study center; head of study center) must be informed as applicable according to local law and regulations.
- All study materials (except documentation that has to remain stored at site) must be returned to the Sponsor. The investigator will retain all other documents, unless differently requested by the Sponsor.

In case of study closure, ongoing subjects must be followed up in an ethical manner.

Additional reason for study termination might be the occurrence of futility, towards the statistical hypothesis which is behind the study design.

18.3 ARCHIVING

The Principal Investigator must ensure the archiving of the essential documents of the study as specified by the GCP (Appendix 4) and in compliance with the applicable legislation. The Principal Investigator or delegates must adopt all the necessary measures to avoid accidental or premature destruction.
Patient (hospital) files will be archived according to local regulations and in accordance with the maximum period of time permitted by the hospital, institution. Where the archiving procedures do not meet the minimum timelines required by the Sponsor, alternative arrangements must be made to ensure the availability of the source documents for the required period. The investigator/institution notifies the Sponsor if the archival arrangements change (e.g. relocation or transfer of ownership). The investigator site file is not to be destroyed without the Sponsor's approval. The contract with the investigator/institution will contain specific reference to study material archiving as defined in site procedures.

18.4 USE OF INFORMATION AND PUBLICATION OF THE STUDY RESULTS

The study is sponsored by the "Fondazione per la Medicina Personalizzata" and therefore the Foundation has the ownership of the results and the commitment to publish them. The study will be registered in the ClinicalTrials.gov database, as well as in the EU Clinical Trial Register and in all the applicable international databases dedicated to clinical trials, in order to comply with the transparency obligations.
All data and results and all intellectual property rights included in the data and results derived from the study will be the property of the Sponsor who may utilize them in various ways, such as, but not limited to, for submission to government regulatory authorities or disclosure to other investigators. The Sponsor recognizes the right of the

investigator to publish the results upon completion of the study. However, the investigator, whilst free to utilize study data derived from his/her center for scientific purposes, must obtain written consent of the Sponsor on the intended publication manuscript before its submission. To this end, the investigator must send a draft of the publication manuscript to the Sponsor within a time period specified in the contract. The Sponsor will review the manuscript promptly and will discuss its content with the investigator to reach a mutually agreeable final manuscript.

18.5 CIVIL LIABILITY INSURANCE

The Sponsor will activate an insurance coverage for the trial, as per the current version of the GCP and local laws pertaining the management of clinical trials requirements.

18.6 FINANCING OF THE STUDY

The study will be partially supported by grants provided by different Companies, whose magnitude is in compliance with the requirements of the fundings acceptable according to the Italian law on Non-Profit Studies (“Decreto 17 Dicembre 2004”).

19 INVESTIGATOR’S RESPONSIBILITY

The Principal Investigator is aware of his/her responsibility for all the actions delegated by him/her to other members of his/her staff assigned to the conduct of the study. The Principal Investigator is obliged to conduct the study in compliance with the study protocol and in adherence to the current version of the Good Clinical Practice (ICH-E6-R2) and with the principles of the Declaration of Helsinki (1964) and subsequent revisions as well as in respect of the applicable legislation.

20 FINAL STUDY REPORT

A final clinical study report (CSR) will be generated on the basis of the Statistical Report of the study. The Final Clinical Study Report will be uploaded in the ClinicalTrials.gov database, as well as in the EU Clinical Trial Register and in all the applicable international databases dedicated to clinical trials, in order to comply with the transparency obligations.

21 BIBLIOGRAPHY

1. SW Grayet al. Attitudes of patients with cancer about personalized medicine and somatic genetic testing J Oncol Pract, 8 (2012), pp. 329–335
2. SW Gray et al. Physicians' attitudes about multiplex tumor genomic testing J Clin Oncol, 32 (2014), pp. 1317–1323
3. M Schwaederle, M Zhao, JJ Lee, et al. Impact of precision medicine in diverse cancers: a meta-analysis of phase II clinical trials J Clin Oncol, 33 (2015), pp. 3817–3825
4. DL Fontes Jardim, M Schwaederle, C Wei, et al.Impact of a biomarker-based strategy on oncology drug development: a meta-analysis of clinical trials leading to FDA approval J Natl Cancer Inst, 107 (2015)
5. MK Krzyzanowska Off-label use of cancer drugs: a benchmark is established J Clin Oncol, 31 (2013), pp. 1125–1127
6. AM Tsimberidou , et al. Personalized medicine in a phase I clinical trials program: The M. D. Anderson Cancer Center Initiative Clin Cancer Res, 18 (2012), pp. 6373–6383
7. Schwaederle M et al On the Road to Precision Cancer Medicine: Analysis of Genomic Biomarker Actionability in 439 Patients. Mol Cancer Ther. 2015 Jun;14(6):1488-94. doi: 10.1158/1535-7163.MCT-14-1061. Epub 2015 Apr 7.
8. Wheler JJ et al. Cancer Therapy Directed by Comprehensive Genomic Profiling: A Single Center Study. Cancer Res. 2016 Jul 1;76(13):3690-701. doi: 10.1158/0008-5472.CAN-15-3043. Epub 2016 May 18.
9. M Gerlinger, et al. Intratumor heterogeneity and branched evolution revealed by multiregion sequencing N Engl J Med, 366 (2012), pp. 883–892
10. G Cirielloet al. Emerging landscape of oncogenic signatures across human cancers Nat Genet, 45 (2013), pp. 1127–1133
11. Drilon A et al. Broad, Hybrid Capture-Based Next-Generation Sequencing Identifies Actionable Genomic Alterations in Lung Adenocarcinomas Otherwise Negative for Such Alterations by Other Genomic Testing Approaches.Clin Cancer Res. 2015 Aug 15;21(16):3631-9. doi: 10.1158/1078-0432.CCR-14-2683. Epub 2015 Jan 7.
12. Schrock AB et al Comprehensive Genomic Profiling Identifies Frequent Drug-Sensitive EGFR Exon 19 Deletions in NSCLC not Identified by Prior Molecular Testing. Clin Cancer Res. 2016 Jul 1;22(13):3281-5. doi: 10.1158/1078-0432.CCR-15-1668. Epub 2016 Mar 1.
13. Alexandrov LB Signatures of mutational processes in human cancer.Nature. 2013 Aug 22;500(7463):415-21. doi: 10.1038/nature12477. Epub 2013 Aug 14.

14. AT Shaw, et al. Crizotinib versus chemotherapy in advanced ALK-positive lung cancer N Engl J Med, 368 (2013), pp. 2385–2394
15. JA Sosman, KB Kim, et al. Survival in BRAF V600-mutant advanced melanoma treated with vemurafenib N Engl J Med, 366 (2012), pp. 707–714
16. BJ Druker et al. Efficacy and safety of a specific inhibitor of the BCR-ABL tyrosine kinase in chronic myeloid leukemia N Engl J Med, 344 (2001), pp. 1031–1037
17. A Sekulic et al. Efficacy and safety of vismodegib in advanced basal-cell carcinoma N Engl J Med, 366 (2012), pp. 2171–2179

18. D Slamon, et al. Adjuvant trastuzumab in HER2-positive breast cancer N Engl J Med, 365 (2011), pp. 1273–1283
19. M Maemondo, , et al. Gefitinib or chemotherapy for non-small-cell lung cancer with mutated EGFR N Engl J Med, 362 (2010), pp. 2380–2388
20. Ali SM et al Comprehensive Genomic Profiling Identifies a Subset of Crizotinib-Responsive ALK-Rearranged Non-Small Cell Lung Cancer Not Detected by Fluorescence In Situ Hybridization. Oncologist. 2016 Jun;21(6):762-70. doi: 10.1634/theoncologist.2015-0497. Epub 2016 May 31.
21. Papadimitrakopoulou V, The BATTLE-2 Study: A Biomarker-Integrated Targeted Therapy Study in Previously Treated Patients With Advanced Non-Small-Cell Lung Cancer. J Clin Oncol. 2016 Aug 1. pii: JCO660084.
22. Le Tourneau C et al. Molecularly targeted therapy based on tumour molecular profiling versus conventional therapy for advanced cancer 8SHIVA): a multicenter open-label, proof of concept randomized controlled phase 2 trial. Lancet Oncol 2015; 16:1324-34
23. IMPACT 2: Randomized Study Evalutating Molecular Profilingand Targeted Agents in Metastatic Cancer NCT02152254
24. Herbert Hurwitz, Pertuzumab + trastuzumab for HER2-amplified/overexpressed metastatic colorectal cancer (mCRC): Interim data from MyPathway DOI: 10.1200/JCO.2017.35.4_suppl.676 Journal of Clinical Oncology 35, no. 4_suppl (February 2017) 676-676
25. Herbert Hurwitz Targeted therapy for gastrointestinal (GI) tumors based on molecular profiles: Early results from MyPathway, an open-label phase IIa basket study in patients with advanced solid tumors. ASCO 2016
26. Parsons HA, Individualized Molecular Analyses Guide Efforts (IMAGE): A Prospective Study of Molecular Profiling of Tissue and Blood in Metastatic Triple Negative Breast Cancer. Clin Cancer Res. 2016 Aug 3. pii: clincanres.1543.2016. [Epub ahead of print]
27. DD von Hoff et al. Pilot study using molecular profiling of patients' tumors to find potential targets and select treatments for their refractory cancers J Clin Oncol, 28 (2010), pp. 4877–4883
28. Klempner SJ, BRAFV600E Mutations in High-Grade Colorectal Neuroendocrine Tumors May Predict Responsiveness to BRAF-MEK Combination Therapy. Cancer Discov. 2016 Jun;6(6):594-600. doi: 10.1158/2159-8290.CD-15-1192. Epub 2016 Apr 5.
29. Pal KS et al Characterization of Clinical Cases of Collecting Duct Carcinoma of the Kidney Assessed by Comprehensive Genomic Profiling. *European Urology.* 3 July 2015.
30. Disel, Alexis Durable clinical benefit to trastuzumab and chemotherapy in a patient with metastatic colon adenocarcinoma harboring ERBB2 amplification. Umut *Oncoscience.*1 July 2015.
31. Doble B Cost-effectiveness of precision medicine in the fourth-line treatment of metastatic lung adenocarcinoma: An early decision analytic model of multiplex targeted sequencing. Lung Cancer. 2017 May;107:22-35. doi: 10.1016/j.lungcan.2016.05.024. Epub 2016 Jun
32. Li Y Cost Effectiveness of Sequencing 34 Cancer-Associated Genes as an Aid for Treatment Selection in Patients with Metastatic Melanoma. Mol Diagn Ther. 2015 Jun;19(3):169-77. doi: 10.1007/s40291-015-0140-9.
33. Gandara DR, Kowanetz M, Mok T, et al. Blood-based biomarkers for cancer immunotherapy: tumor mutational burden in blood (bTMB) is associated with improved atezolizumab (atezo) efficacy in 2L+

NSCLC (POPLAR and OAK). Presented at: 2017 ESMO Congress; Madrid, Spain; September 9-12, 2017. Abstract 1295O.

34. Pivotal Phase III Check Mate 227 , press release BMS
35. Rizvi NA1, Cancer immunology. Mutational landscape determines sensitivity to PD-1 blockade in non-small cell lung cancer. Science. 2015 Apr 3;348(6230):124-8. doi: 10.1126/science.aaa1348. Epub 2015 Mar 12.
36. J Clin Oncol. 2016 May 1;34(13):1510-7. doi: 10.1200/JCO.2015.64.0391. Epub 2016 Mar 7.Evaluation of Immune-Related Response Criteria and RECIST v1.1 in Patients With Advanced Melanoma Treated With Pembrolizumab.
37. Hodi FS1, Hwu WJ2, Kefford R2, Weber JS2, Daud A2, Hamid O2, Patnaik A2, Ribas A2, Robert C2, Gangadhar TC2, Joshua AM2, Hersey P2, Dronca R2, Joseph R2, Hille D2, Xue D2, Li XN2, Kang SP2, Ebbinghaus S2, Perrone A2, Wolchok JD2.
38. Belin L, Kamal M, Mauborgne C. Randomized phase II trial comparing molecularly targeted therapy based on tumor molecular profiling versus conventional therapy in patients with refractory cancer: cross-over analysis from the SHIVA trial.Ann Oncol. 2017 Mar 1;28(3):590-596.
39. BATTLE2 trial: “A Biomarker-Integrated Targeted Therapy Study in Previously Treated Patients With Advanced Non-Small Cell Lung Cancer” (ClinicalTrials.gov Identifier: NCT01248247).
40. Therasse P et al., New guidelines to evaluate the response to treatment in solid tumors. J Natl Cancer Inst. 2000 Feb 2;92(3):205-16.
41. ProfiLER trial: “Program to Establish the Genetic and Immunologic Profile of Patient's Tumor for All Types of Advanced Cancer” (ClinicalTrials.gov Identifier: NCT01774409).
42. MATCHT study: “Targeted Therapy Directed by Genetic Testing in Treating Patients With Advanced Refractory Solid Tumors, Lymphomas, or Multiple Myeloma” (ClinicalTrials.gov Identifier: NCT02465060).
43. TAPUR study: “Testing the Use of Food and Drug Administration (FDA) Approved Drugs That Target a Specific Abnormality in a Tumor Gene in People With Advanced Stage Cancer” (ClinicalTrials.gov Identifier: NCT02693535).
44. IMPACT 2 study: “Molecular Profiling and Targeted Therapy in Treating Patients With Metastatic Cancer” (ClinicalTrials.gov Identifier: NCT02152254).
45. SAFIRO 02_breast study: “Efficacy of Genome Analysis as a Therapeutic Decision Tool for Patients With Metastatic Breast Cancer ” (ClinicalTrials.gov Identifier: NCT02299999).
46. SAFIRO 02_lung study: “Efficacy of Targeted Drugs Guided by Genomic Profiles in Metastatic NSCLC Patients” (ClinicalTrials.gov Identifier: NCT02117167).
47. Lan KKG, Simon R, Halperin M. Stochastically curtailed tests in long-term clinical trials. Comm Statistical-Sequential Analysis 1982;1:207-219.
48. Wang C, Keller DS and Lan KKG. Sample size re-estimation for binary data via conditional power. Joint Statistical Meetings-Biopharmaceutical Section 2002:3621-3626.
49. Siu CO and Lan KKG. Flexible interim analysis method for size re-estimation and early stopping: a conditional power approach. Proceeding of the Annual Meeting of the American Statistical Association, August 5-9, 2001.
50. Schneeweiss, A. et al. Pertuzumab plus trastuzumab in combination with standard neoadjuvant anthracycline-containing and anthracycline-free chemotherapy regimens in patients with HER2-

positive early breast cancer: A randomized phase II cardiac safety study (TRYPHAENA). Ann. Oncol. 24, (2013).

51. Rosell, R. et al. Erlotinib versus standard chemotherapy as first-line treatment for European patients with advanced EGFR mutation-positive non-small-cell lung cancer (EURTAC): A multicentre, open-label, randomised phase 3 trial. Lancet Oncol. (2012). doi:10.1016/S1470-2045(11)70393-X
52. Solomon, B. J. et al. First-line crizotinib versus chemotherapy in ALK-positive lung cancer. N. Engl. J. Med. (2014). doi:10.1056/NEJMoa1408440
53. Douillard, J. Y. et al. Randomized, Phase III trial of panitumumab with infusional fluorouracil, leucovorin, and oxaliplatin (FOLFOX4) Versus FOLFOX4 alone as first-line treatment in patients with previously untreated metastatic colorectal cancer: The PRIME study. J. Clin. Oncol. (2010). doi:10.1200/JCO.2009.27.4860
54. Sartore-Bianchi, A. et al. Dual-targeted therapy with trastuzumab and lapatinib in treatment-refractory, KRAS codon 12/13 wild-type, HER2-positive metastatic colorectal cancer (HERACLES): a proof-of-concept, multicentre, open-label, phase 2 trial. Lancet Oncol. (2016). doi:10.1016/S1470-2045(16)00150-9
55. Robert, C. et al. Improved Overall Survival in Melanoma with Combined Dabrafenib and Trametinib. N. Engl. J. Med. 372, 141116004513004 (2014)
56. Marcus, L., Lemery, S. J., Keegan, P. & Pazdur, R. FDA approval summary: Pembrolizumab for the treatment of microsatellite instability-high solid tumors. Clin. Cancer Res. (2019). doi:10.1158/1078-0432.CCR-18-4070
57. Drilon, A. et al. Safety and antitumor activity of the multitargeted pan-TRK, ROS1, and ALK inhibitor entrectinib: Combined results from two phase I trials (ALKA-372-001 and STARTRK-1). Cancer Discov. (2017). doi:10.1158/2159-8290.CD-16-1237
58. Cardoso, F. et al. 4th ESO-ESMO international consensus guidelines for advanced breast cancer (ABC 4). Ann. Oncol. 29, (2018).
59. Mohammed, A. A., Rashied, H. & Elsayed, F. M. CDK4/6 inhibitors in advanced breast cancer, what is beyond? Oncol. Rev. (2019). doi:10.4081/oncol.2019.416
60. Rossi, L. et al. Clinical outcomes after palbociclib with or without endocrine therapy in postmenopausal women with hormone receptor positive and HER2-negative metastatic breast cancer enrolled in the TREnd trial. Breast Cancer Res. (2019). doi:10.1186/s13058-019-1149-5
61. Condorelli, R. et al. Polyclonal RB1 mutations and acquired resistance to CDK 4/6 inhibitors in patients with metastatic breast cancer. Ann. Oncol. (2018). doi:10.1093/annonc/mdx784
62. Vora, S. R. et al. CDK 4/6 Inhibitors Sensitize PIK3CA Mutant Breast Cancer to PI3K Inhibitors. Cancer Cell (2014). doi:10.1016/j.ccr.2014.05.020
63. Schmid, P. et al. Atezolizumab and nab-paclitaxel in advanced triple-negative breast cancer. N. Engl. J. Med. (2018). doi:10.1056/NEJMoa1809615
64. Villarreal-Garza, C. et al. The prevalence of BRCA1 and BRCA2 mutations among young Mexican women with triple-negative breast cancer. Breast Cancer Res. Treat. (2015). doi:10.1007/s10549-015-3312-8
65. Telli, M. L., Gradishar, W. J. & Ward, J. H. NCCN Guidelines Updates: Breast Cancer. J. Natl. Compr. Canc. Netw. (2019). doi:10.6004/jnccn.2019.5006

66. Peters, S. et al. Alectinib versus crizotinib in untreated ALK-positive non–small-cell lung cancer. N. Engl. J. Med. (2017). doi:10.1056/NEJMoa1704795
67. Soria, J.-C. et al. Osimertinib in Untreated EGFR -Mutated Advanced Non–Small-Cell Lung Cancer. N. Engl. J. Med. NEJMoa1713137 (2017). doi:10.1056/NEJMoa1713137
68. Planchard, D. et al. Dabrafenib in patients with BRAFV600E-positive advanced non-small-cell lung cancer: A single-arm, multicentre, open-label, phase 2 trial. Lancet Oncol. (2016). doi:10.1016/S1470-2045(16)00077-2
69. Shaw, A. T. et al. Lorlatinib in non-small-cell lung cancer with ALK or ROS1 rearrangement: an international, multicentre, open-label, single-arm first-in-man phase 1 trial. Lancet Oncol. (2017). doi:10.1016/S1470-2045(17)30680-0
70. Reck, M. et al. Pembrolizumab versus Chemotherapy for PD-L1-Positive Non-Small-Cell Lung Cancer. N. Engl. J. Med. 375, 1823–1833 (2016).
71. Langer, C. J. et al. Carboplatin and pemetrexed with or without pembrolizumab for advanced, non-squamous non-small-cell lung cancer: a randomised, phase 2 cohort of the open-label KEYNOTE-021 study. Lancet Oncol. (2016). doi:10.1016/S1470-2045(16)30498-3
72. Reck, M. et al. Updated analysis of KEYNOTE-024: Pembrolizumab versus platinum-based chemotherapy for advanced non–small-cell lung cancer with PD-L1 tumor proportion score of 50% or greater. J. Clin. Oncol. (2019). doi:10.1200/JCO.18.00149
73. Hong, D. S. et al. Larotrectinib in patients with TRK fusion-positive solid tumours: a pooled analysis of three phase 1/2 clinical trials. Lancet Oncol. (2020). doi:10.1016/S1470-2045(19)30856-3
74. Van Cutsem, E. et al. ESMO consensus guidelines for the management of patients with metastatic colorectal cancer. Ann. Oncol. (2016). doi:10.1093/annonc/mdw235
75. Grothey, A. et al. Regorafenib monotherapy for previously treated metastatic colorectal cancer (CORRECT): An international, multicentre, randomised, placebo-controlled, phase 3 trial. Lancet (2013). doi:10.1016/S0140-6736(12)61900-X
76. Mayer, R. J. et al. Randomized trial of TAS-102 for refractory metastatic colorectal cancer. N. Engl. J. Med. (2015). doi:10.1056/NEJMoa1414325
77. Dummer, R. et al. Encorafenib plus binimetinib versus vemurafenib or encorafenib in patients with BRAF-mutant melanoma (COLUMBUS): a multicentre, open-label, randomised phase 3 trial. Lancet Oncol. 19, 603–615 (2018).
78. Le, D. T. et al. PD-1 blockade in tumors with mismatch-repair deficiency. N. Engl. J. Med. (2015). doi:10.1056/NEJMoa1500596

22 APPENDICES

APPENDIX 1	Tailored Therapy: Period of Contraception/Abstinence after last dose of treatment
APPENDIX 2	Tailored Therapy: Information and recommendations for the use of TT IMPs
APPENDIX 3	Declaration of Helsinki
APPENDIX 4	ICH E6 –Good Clinical Practice (E6-R2)

APPENDIX I

Tailored Therapy: Period of Contraception/Abstinence after last dose of treatment

Active Substance	Trade name (if available)	Period of contraception/abstinence after the last dose of treatment	
		Female	Male
Erlotinib	Tarceva	3 months	3 months
Trastuzumab	Herceptin	7 months	3 months
Pertuzumab	Parjeta	6 months	3 months
Trastuzumab Emtansine	Kadcyla	7 months	7 months
Entrectinib	-	3 months	3 months
Lapatinib	Tyverb	3 months	3 months
Everolimus	Afinitor	3 months	3 months
Vemurafenib	Zelboraf	3 months	3 months
Cobimetinib	Cotellic	3 months	3 months
Alectinib	Alecensa	3 months	3 months
Palbociclib	Ibrance	3 months	3 months
Ponatinib	Iclusig	3 months	3 months
Vismodegib	Erivedge	24 months	3 months
Atezolizumab	Tecentriq	5 months	3 months
Nivolumab	Opdivo	5 months	3 months
Ipilimumab	Yervoy	3 months	3 months
Brigatinib	Alunbrig	4 months	3 months
Itacitinib (INCB039110)	-	3 months	3 months
Pemigatinib (INCB054828)	-	3 months	3 months
Ipatasertib (GDC-0068)	-	3 months	3 months
Alpelisib	Piqray	3 months	3 months
Tepotinib	-	3 months	3 months
Pralsetinib	Gavreto	3 months	3 months
Talazoparib	Talzenna	7 months	4 months
Selpercatinib	Retevmo	6 months	6 months

APPENDIX 2

Tailored Therapy: Information and recommendations for the use of TT IMPs

(PLEASE REFER TO A SEPARATE ENCLOSURE)

APPENDIX 3 Declaration of Helsinki

Adopted by the 18th WMA General Assembly, Helsinki, Finland, June 1964
and amended by the:
29th WMA General Assembly, Tokyo, Japan, October 1975
35th WMA General Assembly, Venice, Italy, October 1983
41st WMA General Assembly, Hong Kong, September 1989
48th WMA General Assembly, Somerset West, Republic of South Africa, October 1996
52nd WMA General Assembly, Edinburgh, Scotland, October 2000
53rd WMA General Assembly, Washington DC, USA, October 2002 (Note of Clarification added)
55th WMA General Assembly, Tokyo, Japan, October 2004 (Note of Clarification added)
59th WMA General Assembly, Seoul, Republic of Korea, October 2008
64th WMA General Assembly, Fortaleza, Brazil, October 2013

Preamble

1. The World Medical Association (WMA) has developed the Declaration of Helsinki as a statement of ethical principles for medical research involving human subjects, including research on identifiable human material and data.

The Declaration is intended to be read as a whole and each of its constituent paragraphs should be applied with consideration of all other relevant paragraphs.

2. Consistent with the mandate of the WMA, the Declaration is addressed primarily to physicians. The WMA encourages others who are involved in medical research involving human subjects to adopt these principles.

General Principles

3. The Declaration of Geneva of the WMA binds the physician with the words, “The health of my patient will be my first consideration,” and the International Code of Medical Ethics declares that, “A physician shall act in the patient's best interest when providing medical care.”

4. It is the duty of the physician to promote and safeguard the health, well-being and rights of patients, including those who are involved in medical research. The physician's knowledge and conscience are dedicated to the fulfilment of this duty.

5. Medical progress is based on research that ultimately must include studies involving human subjects.

6. The primary purpose of medical research involving human subjects is to understand the causes, development and effects of diseases and improve preventive, diagnostic and therapeutic interventions (methods, procedures and treatments). Even the best proven interventions must be evaluated continually through research for their safety, effectiveness, efficiency, accessibility and quality.

7. Medical research is subject to ethical standards that promote and ensure respect for all human subjects and protect their health and rights.

8. While the primary purpose of medical research is to generate new knowledge, this goal can never take precedence over the rights and interests of individual research subjects.

9. It is the duty of physicians who are involved in medical research to protect the life, health, dignity, integrity, right to self-determination, privacy, and confidentiality of personal information of research subjects. The responsibility for the protection of research subjects must always rest with the physician or other health care professionals and never with the research subjects, even though they have given consent.

10. Physicians must consider the ethical, legal and regulatory norms and standards for research involving human subjects in their own countries as well as applicable international norms and standards. No national or international

ethical, legal or regulatory requirement should reduce or eliminate any of the protections for research subjects set forth in this Declaration.

11. Medical research should be conducted in a manner that minimises possible harm to the environment.

12. Medical research involving human subjects must be conducted only by individuals with the appropriate ethics and scientific education, training and qualifications. Research on patients or healthy volunteers requires the supervision of a competent and appropriately qualified physician or other health care professional.

13. Groups that are underrepresented in medical research should be provided appropriate access to participation in research.

14. Physicians who combine medical research with medical care should involve their patients in research only to the extent that this is justified by its potential preventive, diagnostic or therapeutic value and if the physician has good reason to believe that participation in the research study will not adversely affect the health of the patients who serve as research subjects.

15. Appropriate compensation and treatment for subjects who are harmed as a result of participating in research must be ensured.

Risks, Burdens and Benefits

16. In medical practice and in medical research, most interventions involve risks and burdens.

Medical research involving human subjects may only be conducted if the importance of the objective outweighs the risks and burdens to the research subjects.

17. All medical research involving human subjects must be preceded by careful assessment of predictable risks and burdens to the individuals and groups involved in the research in comparison with foreseeable benefits to them and to other individuals or groups affected by the condition under investigation.

Measures to minimise the risks must be implemented. The risks must be continuously monitored, assessed and documented by the researcher.

18. Physicians may not be involved in a research study involving human subjects unless they are confident that the risks have been adequately assessed and can be satisfactorily managed.

When the risks are found to outweigh the potential benefits or when there is conclusive proof of definitive outcomes, physicians must assess whether to continue, modify or immediately stop the study.

Vulnerable Groups and Individuals

19. Some groups and individuals are particularly vulnerable and may have an increased likelihood of being wronged or of incurring additional harm.

All vulnerable groups and individuals should receive specifically considered protection.

20. Medical research with a vulnerable group is only justified if the research is responsive to the health needs or priorities of this group and the research cannot be carried out in a non-vulnerable group. In addition, this group should stand to benefit from the knowledge, practices or interventions that result from the research.

Scientific Requirements and Research Protocols

21. Medical research involving human subjects must conform to generally accepted scientific principles, be based on a thorough knowledge of the scientific literature, other relevant sources of information, and adequate laboratory and, as appropriate, animal experimentation. The welfare of animals used for research must be respected.

22. The design and performance of each research study involving human subjects must be clearly described and justified in a research protocol.

The protocol should contain a statement of the ethical considerations involved and should indicate how the principles in this Declaration have been addressed. The protocol should include information regarding funding, sponsors, institutional affiliations, potential conflicts of interest, incentives for subjects and information regarding provisions for treating and/or compensating subjects who are harmed as a consequence of participation in the research study.

In clinical trials, the protocol must also describe appropriate arrangements for post-trial provisions.

Research Ethics Committees

23. The research protocol must be submitted for consideration, comment, guidance and approval to the concerned research ethics committee before the study begins. This committee must be transparent in its functioning, must be independent of the researcher, the sponsor and any other undue influence and must be duly qualified. It must take into consideration the laws and regulations of the country or countries in which the research is to be performed as well as applicable international norms and standards but these must not be allowed to reduce or eliminate any of the protections for research subjects set forth in this Declaration.

The committee must have the right to monitor ongoing studies. The researcher must provide monitoring information to the committee, especially information about any serious adverse events. No amendment to the protocol may be made without consideration and approval by the committee. After the end of the study, the researchers must submit a final report to the committee containing a summary of the study's findings and conclusions.

Privacy and Confidentiality

24. Every precaution must be taken to protect the privacy of research subjects and the confidentiality of their personal information.

Informed Consent

25. Participation by individuals capable of giving informed consent as subjects in medical research must be voluntary. Although it may be appropriate to consult family members or community leaders, no individual capable of giving informed consent may be enrolled in a research study unless he or she freely agrees.

26. In medical research involving human subjects capable of giving informed consent, each potential subject must be adequately informed of the aims, methods, sources of funding, any possible conflicts of interest, institutional affiliations of the researcher, the anticipated benefits and potential risks of the study and the discomfort it may entail, post-study provisions and any other relevant aspects of the study. The potential subject must be informed of the right to refuse to participate in the study or to withdraw consent to participate at any time without reprisal. Special attention should be given to the specific information needs of individual potential subjects as well as to the methods used to deliver the information.

After ensuring that the potential subject has understood the information, the physician or another appropriately qualified individual must then seek the potential subject's freely-given informed consent, preferably in writing. If the consent cannot be expressed in writing, the non-written consent must be formally documented and witnessed.

All medical research subjects should be given the option of being informed about the general outcome and results of the study.

27. When seeking informed consent for participation in a research study the physician must be particularly cautious if the potential subject is in a dependent relationship with the physician or may consent under duress. In such situations the informed consent must be sought by an appropriately qualified individual who is completely independent of this relationship.

28. For a potential research subject who is incapable of giving informed consent, the physician must seek informed consent from the legally authorised representative. These individuals must not be included in a research study that has no likelihood of benefit for them unless it is intended to promote the health of the group represented by the potential subject, the research cannot instead be performed with persons capable of providing informed consent, and the research entails only minimal risk and minimal burden.

29. When a potential research subject who is deemed incapable of giving informed consent is able to give assent to decisions about participation in research, the physician must seek that assent in addition to the consent of the legally authorised representative. The potential subject's dissent should be respected.

30. Research involving subjects who are physically or mentally incapable of giving consent, for example, unconscious patients, may be done only if the physical or mental condition that prevents giving informed consent is a necessary

characteristic of the research group. In such circumstances the physician must seek informed consent from the legally authorised representative. If no such representative is available and if the research cannot be delayed, the study may proceed without informed consent provided that the specific reasons for involving subjects with a condition that renders them unable to give informed consent have been stated in the research protocol and the study has been approved by a research ethics committee. Consent to remain in the research must be obtained as soon as possible from the subject or a legally authorised representative.

31. The physician must fully inform the patient which aspects of their care are related to the research. The refusal of a patient to participate in a study or the patient's decision to withdraw from the study must never adversely affect the patient-physician relationship.

32. For medical research using identifiable human material or data, such as research on material or data contained in biobanks or similar repositories, physicians must seek informed consent for its collection, storage and/or reuse. There may be exceptional situations where consent would be impossible or impracticable to obtain for such research. In such situations the research may be done only after consideration and approval of a research ethics committee.

Use of Placebo

33. The benefits, risks, burdens and effectiveness of a new intervention must be tested against those of the best proven intervention(s), except in the following circumstances:

Where no proven intervention exists, the use of placebo, or no intervention, is acceptable; or

Where for compelling and scientifically sound methodological reasons the use of any intervention less effective than the best proven one, the use of placebo, or no intervention is necessary to determine the efficacy or safety of an intervention and the patients who receive any intervention less effective than the best proven one, placebo, or no intervention will not be subject to additional risks of serious or irreversible harm as a result of not receiving the best proven intervention.

Extreme care must be taken to avoid abuse of this option.

Post-Trial Provisions

34. In advance of a clinical trial, sponsors, researchers and host country governments should make provisions for post-trial access for all participants who still need an intervention identified as beneficial in the trial. This information must also be disclosed to participants during the informed consent process.

Research Registration and Publication and Dissemination of Results

35. Every research study involving human subjects must be registered in a publicly accessible database before recruitment of the first subject.

36. Researchers, authors, sponsors, editors and publishers all have ethical obligations with regard to the publication and dissemination of the results of research. Researchers have a duty to make publicly available the results of their research on human subjects and are accountable for the completeness and accuracy of their reports. All parties should adhere to accepted guidelines for ethical reporting. Negative and inconclusive as well as positive results must be published or otherwise made publicly available. Sources of funding, institutional affiliations and conflicts of interest must be declared in the publication. Reports of research not in accordance with the principles of this Declaration should not be accepted for publication.

Unproven Interventions in Clinical Practice

37. In the treatment of an individual patient, where proven interventions do not exist or other known interventions have been ineffective, the physician, after seeking expert advice, with informed consent from the patient or a legally authorised representative, may use an unproven intervention if in the physician's judgement it offers hope of saving life, re-establishing health or alleviating suffering. This intervention should subsequently be made the object of research, designed to evaluate its safety and efficacy. In all cases, new information must be recorded and, where appropriate, made publicly available.

APPENDIX 4 GOOD CLINICAL PRACTICES (E6-R2)

https://www.ich.org/fileadmin/Public_Web_Site/ICH_Products/Guidelines/Efficacy/E6/E6_R2__Step_4_2016_1109.pdf

APPENDIX 2

Tailored Therapy: Information and recommendations for the use of TT IMPs

	Active Ingredient	Trade Name (if available)	Funding Company
1.	Erlotinib	Tarceva	Roche
2.	Pertuzumab	Parjeta	Roche
3.	Vemurafenib	Zelboraf	Roche
4.	Trastuzumab Emtansine	Kadcyla	Roche
5.	Alectinib	Alecensa	Roche
6.	Vismodegib	Erivedge	Roche
7.	Cobimetinib	Cotellic	Roche
8.	Atezolizumab	Tecentriq	Roche
9.	Trastuzumab	Herceptin	Roche
10.	Ipatasertib (GDC-0068)	-	Roche
11.	Entrectinib	Rodslytrek	Roche
12.	Everolimus	Afinitor	Novartis
13.	Palbociclib	Ibrance	Pfizer
14.	Lapatinib	Tyverb	Novartis
15.	Ipilimumab	Yervoy	Bristol-Myers Squibb
16.	Nivolumab	Opdivo	Bristol-Myers Squibb
17.	Brigatinib	Alunbrig	Takeda Pharmaceutical Co.
18.	Ponatinib	Iclusig	Incyte
19.	Itacitinib (INCB039110)	-	Incyte
20.	Pemigatinib (INCB054828)	-	Incyte
21.	Alpelisib	Piqray	Novartis
22.	Tepotinib	-	Merck Healthcare KGaA
23.	Pralsetinib	Gavreto	Roche
24.	Talazoparib	Talzenna	Pfizer
25.	Selpercatinib	Retevmo	Eli Lilly

1 TARCEVA (ERLOTINIB)

Investigators should also consult the local prescribing information if the IMP is approved in your country.

1.1 Standard dose

Tarceva will be used at the standard dose of 150 mg/die (as approved for EGFR mutated NSCLC). First dose reduction: 100 mg/die.

1.2 Contraindications

Tarceva is contraindicated in patients with severe hypersensitivity to erlotinib or to any component of Tarceva.

1.3 Warnings and Precautions

General

<u>Interstitial Lung Disease</u>

Cases of ILD-like events, including fatalities, have been reported uncommonly in patients receiving erlotinib for treatment of NSCLC, pancreatic cancer or other advanced solid tumors. In the pivotal study BR.21 in NSCLC, the incidence of serious ILD-like events was 0.8% in each of the placebo/control and erlotinib arms. The incidence of ILD and ILD-like events in erlotinib-treated patients calculated from NSCLC studies using a meta-analysis of randomized controlled clinical trials (excluding phase 1 and single-arm phase 2 studies due to lack of control groups) was 0.9% compared to 0.4% in patients on placebo [Shi et al, 2014].
In the pancreatic cancer study in combination with gemcitabine (Study PA.3), the
incidence of ILD-like events was 2.5% in the erlotinib plus gemcitabine group versus 0.4% in the placebo plus gemcitabine-treated group.
Some examples of reported diagnoses in patients suspected of having ILD-like events include pneumonitis, radiation pneumonitis, hypersensitivity pneumonitis, interstitial pneumonia, ILD, obliterative bronchiolitis, pulmonary fibrosis, acute respiratory distress syndrome, lung infiltration and alveolitis. These ILD-like events started from a few days to several months after initiating erlotinib therapy. Most of the cases were associated with confounding or contributing factors such as concomitant or prior chemotherapy, prior radiotherapy, pre-existing parenchymal lung disease, metastatic lung disease, or pulmonary infections.
In patients who develop acute onset of new and/or progressive unexplained pulmonary symptoms, such as dyspnea, cough and fever, erlotinib therapy should be interrupted pending diagnostic evaluation. If ILD is diagnosed, erlotinib should be discontinued and appropriate treatment initiated as necessary.

<u>Diarrhea, Dehydration, Electrolyte Imbalance and Renal Failure</u>

Diarrhea has occurred in patients on erlotinib, and moderate or severe diarrhea should be treated with loperamide. In some cases, dose reduction may be necessary. In the event of severe or persistent diarrhea, nausea, anorexia or vomiting associated with dehydration, erlotinib therapy should be interrupted and appropriate measures should be taken to treat the dehydration. There have been rare reports of hypokalemia and renal failure (including fatalities). Some reports of renal failure were secondary to severe dehydration due to diarrhea, vomiting and/or anorexia while others were confounded by concomitant chemotherapy. In more severe or persistent cases of diarrhea, or cases leading to dehydration, particularly in groups of patients with aggravating risk factors (concomitant medications, symptoms or diseases or other predisposing conditions including advanced age), erlotinib therapy should be interrupted and appropriate measures should be taken to intensively rehydrate the patients intravenously. In addition, renal function and serum electrolytes including potassium should be monitored in patients at risk of dehydration.

Hepatitis, Hepatic Failure

Rare cases of hepatic failure (including fatalities) have been reported during use of erlotinib. Confounding factors have included pre-existing liver disease or concomitant hepatotoxic medications. Therefore, in such patients, periodic liver function testing should be considered. Erlotinib dosing should be interrupted if changes in liver function are severe.

Gastrointestinal Perforation: Patients receiving erlotinib are at increased risk of developing gastrointestinal perforation, which was observed uncommonly (including some cases with a fatal outcome). Patients receiving concomitant anti-angiogenic agents, corticosteroids, nonsteroidal anti-inflammatory drugs and/or taxane based chemotherapy, or who have prior history of peptic ulceration or diverticular disease are at increased risk.

Erlotinib should be permanently discontinued in patients who develop gastrointestinal perforation.

Bullous and Exfoliative Skin Disorders

Bullous, blistering and exfoliative skin conditions have been reported, including very rare cases suggestive of Stevens-Johnson syndrome/Toxic epidermal necrolysis, which in some cases were fatal. Erlotinib treatment should be interrupted or discontinued if the patient develops severe bullous, blistering or exfoliating conditions.

Ocular Disorders

Very rare cases of corneal perforation or ulceration have been reported during use of erlotinib. Other ocular disorders including abnormal eyelash growth, keratoconjunctivitis sicca or keratitis have been observed with erlotinib treatment, which are also risk factors for corneal perforation/ulceration [Horton, 2008; Papadopoulos et al, 2008; Perry, 2008]. Erlotinib therapy should be interrupted or discontinued if patients present with acute/worsening ocular disorders such as eye pain.

Table: Summary of ADRs per frequency category:

**Body System**	**Very common (≥1/10)**	**Common (≥1/100 to <1/10)**	**Uncommon (≥1/1,000 to <1/100)**	**Rare (≥1/10,000 to <1/1,000)**	**Very rare (<1/10,000)**
Eye disorders		-Keratitis<br>-Conjunctivitis[1]	-Eyelash changes [2]		-Corneal perforations<br>-Corneal ulcerations<br>-Uveitis
Respiratory, thoracic and mediastinal disorders		-Epistaxis	-Interstitial lung disease (ILD)[3]		
Gastro-intestinal disorders	-Diarrhoea[7]	-Gastro-intestinal bleeding[4, 7]	-Gastro-intestinal perforations[7]		
Hepato biliary disorders	-Liver function test abnormalities 5			-Hepatic failure [6]	

Skin and subcutaneous tissue disorders	-Rash	-Alopecia<br>-Dry skin[1]<br>-Paronychia<br>-Folliculitis<br>-Acne/ Dermatitis acneiform<br>-Skin fissures	-Hirsutism<br>-Eyebrow changes<br>-Brittle and Loose nails<br>-Mild skin reactions such as hyperpigmentation	-Palmar plantar erythrodys-aesthesia syndrome	-Stevens-Johnson syndrome/Toxic epidermal necrolysis[7]
Renal and urinary disorders		-Renal insufficiency[1]	-Nephritis[1]<br>-Proteinuria[1]		

[1] In clinical study PA.3.
[2] Including in-growing eyelashes, excessive growth and thickening of the eyelashes.
[3] Including fatalities, in patients receiving Tarceva for treatment of NSCLC or other advanced solid tumours
[4] In clinical studies, some cases have been associated with concomitant warfarin administration and some with concomitant NSAID administration
[5] Including increased alanine aminotransferase [ALT], aspartate aminotransferase [AST] and bilirubin. These were very common in clinical study PA.3 and common in clinical study BR.21. Cases were mainly mild to moderate in severity, transient in nature or associated with liver metastases.
[6] Including fatalities. Confounding factors included pre-existing liver disease or concomitant hepatotoxic medications
[7] Including fatalities

Interactions: Erlotinib has a potential for clinically significant drug-drug interactions.

Drug Abuse and Dependence

Not known.

Ability to Drive and Use Machines

Erlotinib has no or negligible influence on the ability to drive and use machines.

Interactions with Other Medicinal Products and Other Forms of Interaction

Erlotinib is metabolized in the liver by the hepatic CYPs in humans, primarily CYP3A4 and to a lesser extent by CYP1A2, and the pulmonary isoform CYP1A1. Potential interactions may occur with drugs that are metabolized by, or are inhibitors or inducers of these enzymes.

Potent inhibitors of CYP3A4 activity decrease erlotinib metabolism and increase erlotinib plasma concentrations. Inhibition of CYP3A4 metabolism by ketoconazole (200 mg po bid for 5 days) resulted in increased exposure to erlotinib (86% in median erlotinib exposure [AUC] and a 69% increase in Cmax) when compared to erlotinib alone. When erlotinib was coadministered with ciprofloxacin, an inhibitor of both CYP3A4 and CYP1A2, the erlotinib exposure (AUC) and maximum concentration (Cmax) increased by 39% and 17%, respectively (Study BP20045). Therefore caution should be used when administering erlotinib with potent CYP3A4 or combined CYP3A4/CYP1A2 inhibitors.

In these situations, the dose of erlotinib should be reduced if toxicity is observed (Study NP16612).

Potent inducers of CYP3A4 activity increase erlotinib metabolism and significantly decrease erlotinib plasma concentrations. Induction of CYP3A4 metabolism by

rifampicin (600 mg po qd for 7 days) resulted in a 69% decrease in the median erlotinib AUC, following a 150 mg dose of erlotinib as compared to erlotinib alone
(Study NP16638). Pretreatment and coadministration of rifampicin with a single 450 mg dose of erlotinib resulted in a mean erlotinib exposure (AUC) of 57.5% of that after a single 150 mg erlotinib dose in the absence of rifampicin treatment (Study OSI-774-105).
Alternative treatments lacking potent CYP3A4 inducing activity should be considered when possible. For patients who require concomitant treatment with erlotinib and a potent CYP3A4 inducer such as rifampicin, an increase in dose to 300 mg should be considered while their safety is closely monitored, and if well tolerated for more than 2 weeks, further increase to 450 mg could be considered with close safety monitoring.
Higher doses have not been studied in this setting.
Pretreatment or coadministration of erlotinib did not alter the clearance of the
prototypical CYP3A4 substrates midazolam and erythromycin. Significant interactions with the clearance of other CYP3A4 substrates are therefore unlikely (Study NP17536).
Oral availability of midazolam did appear to decrease by up to 24%, which was however not attributed to effects on CYP3A4 activity.
The solubility of erlotinib is pH dependent. Erlotinib solubility decreases as pH
increases. Drugs that alter the pH of the upper gastrointestinal tract may alter the
solubility of erlotinib and hence its bioavailability. Coadministration of erlotinib with
omeprazole, a proton pump inhibitor, decreased the erlotinib exposure (AUC) and Cmax by 46% and 61%, respectively. There was no change to tmax or half-life (Study BP20046). Concomitant administration of erlotinib with 300 mg ranitidine, an H2-receptor antagonist, decreased erlotinib exposure (AUC) and Cmax by 33% and 54%, respectively (Study BP21502). Therefore, coadministration of drugs reducing gastric acid production with erlotinib should be avoided where possible. Increasing the dose of erlotinib when coadministered with such agents is not likely to compensate for this loss of exposure. However, when erlotinib was dosed in a staggered manner 2 hours before or 10 hours after ranitidine 150 mg twice a day, erlotinib exposure (AUC) and Cmax decreased only by 15% and 17%, respectively (Study BP21502). If patients need to be treated with such drugs, then an H2-receptor antagonist such as ranitidine should be considered and used in a staggered manner. Erlotinib must be taken at least 2 hours before or 10 hours after the H2-receptor antagonist dosing.
Interaction with coumarin-derived anticoagulants, including warfarin, leading to
increased INR and bleeding events, which in some cases were fatal, have been reported in patients receiving erlotinib. Patients taking coumarin-derived anticoagulants should be monitored regularly for any changes in prothrombin time or INR (Studies BR.21 and OSI2298g).
The combination of erlotinib and a statin may increase the potential for statin-induced myopathy, including rhabdomyolysis, which was observed rarely.
Smokers should be advised to stop smoking as cigarette smoking, which is known to induce CYP1A1 and CYP1A2, has been shown to reduce erlotinib exposure by 50-60%.
Concomitant use of Tarceva with moderate or strong CYP1A2 inducers should be
avoided. In 2 phase 1b studies, there were no significant effects of gemcitabine on the pharmacokinetics of erlotinib (Studies OSI-774-155 and OSI-774-108) nor were there significant effects of erlotinib on the pharmacokinetics of gemcitabine
(Study OSI-774-155).

1.4 Special Populations

Pregnancy

Refer to appendix 1. There are no adequate or well-controlled studies in pregnant women using erlotinib. Studies in animals have shown some reproductive toxicity. The potential risk for humans is unknown. Women of childbearing potential must be advised to avoid pregnancy while on erlotinib. Adequate contraceptive methods should be used during therapy and for at least 2 weeks after completing therapy. Treatment should only be continued in pregnant women if the potential benefit to the mother outweighs the risk to the fetus.

Nursing Mothers
It is not known whether erlotinib is excreted in human milk. No studies have been conducted to assess the impact of Tarceva on milk production or its presence in breast milk. As the potential for harm to the nursing infant is unknown, mothers should be advised against breast-feeding while receiving erlotinib therapy and for at least 2 weeks after the final dose.

Hepatic Impairment
Erlotinib exposure was similar in patients with moderately impaired hepatic function (Child-Pugh score 7-9) compared with patients with adequate hepatic function including patients with primary liver cancer or hepatic metastases (Study OSI-774-104). The safety and efficacy of erlotinib have not been studied in patients with severe hepatic impairment.

Geriatric Use
No information available.

1.5 Overdose

Single oral doses of erlotinib up to 1000 mg in healthy subjects (Study 248-001), and up to 1600 mg given as a single dose once weekly in cancer patients (Study 248-005), have been tolerated. Repeated twice daily doses of 200 mg in healthy subjects were poorly tolerated after only a few days of dosing (Study 248-002). Based on the data from these studies, severe AEs such as diarrhea, rash and possibly liver transaminase elevation may occur above the recommended dose. In case of suspected overdose, erlotinib should be withheld and symptomatic treatment administered.

2 PERJETA (PERTUZUMAB)

Investigators should also consult the local prescribing information if the IMP is approved in your country.

2.1 Standard dose:

The recommended starting loading dose of pertuzumab is 840 mg to be administered by intravenous infusion over 60 minutes, followed subsequently by a maintenance dose of 420 mg administered every 3 weeks over 30-60 minutes. After each infusion of Perjeta, an observation period of 30-60 minutes is recommended after the completion of each infusion, which must be completed before subsequent infusions of trastuzumab or chemotherapy. Dose reductions are not recommended for Perjeta.

Table 1: Recommendations regarding delayed or missed doses

Time between two sequential infusions	Perjeta	trastuzumab	
		IV	SC
< 6 weeks	The 420 mg dose of pertuzumab should be administered as soon as possible. Do not wait until the next planned dose. Thereafter, revert to the original planned schedule.	The 6 mg/kg dose of trastuzumab IV should be administered as soon as possible. Do not wait until the next planned dose. Thereafter, revert to the original planned schedule.	The fixed dose of 600mg trastuzumab SC should be administered as soon as possible. Do not wait until the next planned dose.
≥ 6 weeks	The 840 mg loading dose of pertuzumab should be re-administered as a 60 minute infusion, followed by a maintenance dose of 420 mg IV administered every 3 weeks thereafter.	The loading dose of 8 mg/kg of trastuzumab IV should be re-administered over approximately 90 minutes, followed by a maintenance dose of 6 mg/kg IV administered every 3 weeks thereafter.	

2.2 Contraindications

Perjeta is contraindicated in patients with known hypersensitivity to Perjeta or to any of its excipients.

2.3 Warnings and Precautions

Infusion-Related Reactions, Hypersensitivity Reactions and Anaphylaxis

Perjeta has been associated with infusion reactions. Close observation of the patient during and for 60 minutes after the first infusion and during and for 30 minutes following subsequent infusions of Perjeta is recommended. If a significant infusion reaction occurs, the infusion should be slowed down or interrupted and appropriate medical therapies should be administered as per local practice. Patients should be evaluated and carefully monitored until complete resolution of signs and symptoms. Permanent discontinuation should be considered in patients with severe infusion reactions. This clinical assessment should be based on the severity of the preceding reaction and response to administered treatment for the adverse reaction. Patients should be observed closely for hypersensitivity reactions. Severe hypersensitivity, including anaphylaxis, has been observed in clinical trials with treatment of Perjeta.

Medications to treat such reactions, as well as emergency equipment, should be available for immediate use. Perjeta is contraindicated in patients with known hypersensitivity to Perjeta or to any of its excipients.

Left Ventricular Dysfunction
Patients should have a pre-treatment left ventricular ejection fraction (LVEF) of ≥ 50%. Perjeta and trastuzumab should be withheld for at least 3 weeks for:

- a drop in LVEF to less than 40%
- a LVEF of 40%-45% associated with a fall of ≥ 10% points below pre-treatment value.

Perjeta and trastuzumab may be resumed if the LVEF has recovered to > 45%, or to 40-45% associated with a difference of < 10% points below pre-treatment values.

Decreases in LVEF have been reported with drugs that block HER2 activity, including Perjeta. In the pivotal Phase III trial WO20698/TOC4129g (CLEOPATRA) in patients with HER2-positive metastatic breast cancer, Perjeta in combination with Herceptin and docetaxel was not associated with increases in the incidence of symptomatic left ventricular systolic dysfunction (LVD [congestive heart failure]) or decreases in LVEF compared with placebo in combination with Herceptin and docetaxel. However, patients who have received prior anthracyclines or prior radiotherapy to the chest area may be at higher risk of a LVEF decrease. In neoadjuvant treated patients Study WO20697 (NEOSPHERE study), the incidence of left ventricular dysfunction was higher in the Perjeta-treated groups than the Herceptin and docetaxel treated group. An increased incidence of LVEF declines was observed in patients treated with Perjeta in combination with Herceptin and docetaxel; LVEF recovered to ≥50% in all patients.
In the adjuvant treatment setting Study BO25126/TOC4939g (APHINITY), the incidence of primary cardiac events (Cardiac death [definite or probable] or NYHA III/IV Heart Failure with a drop in LVEF [of at least 10 EF points from baseline and to below 50%] was low (<1%) in both treatment arms. The rate of secondary cardiac events (defined as an asymptomatic or mildly symptomatic [NYHA Class II] significant LVEF drop of at least 10 EF points below baseline and to below 50%) was low (<3%) and similar between the treatment arms.
Cardiac exclusion criteria are specified in the protocols and should be closely followed. LVEF must be assessed prior to initiation of Perjeta and at regular intervals during treatment to ensure that LVEF is within the institution’s normal limits. Further monitoring of left ventricular systolic function during the study is specified in the protocol as well as the cardiac algorithm for when Perjeta and Herceptin need to be withheld and a repeat assessment to be performed; these instructions should be closely followed. The same applies for criteria of permanent discontinuation of Perjeta and Herceptin.

Diarrhoea
Perjeta may elicit severe diarrhoea. Diarrhoea is most frequent during concurrent administration with taxane therapy. Elderly patients (> 65 years) may have a higher risk of diarrhoea compared with younger patients (< 65 years). Treat diarrhoea according to standard practice and guidelines. Early intervention with loperamide, fluids and electrolyte replacement should be considered, particularly in elderly patients, and in case of severe or prolonged diarrhoea. Interruption of treatment with pertuzumab should be considered if no improvement in the patient’s condition is achieved. When the diarrhoea is under control treatment with pertuzumab may be reinstated.

Table 2: ADRs related to pertuzumab

**System organ class**	***Very Common***	***Common***	***Uncommon***	***Rare***

Skin and subcutaneous tissue disorders	Alopecia<br>Rash<br>Nail disorder<br>Pruritus<br>Dry skin			
Musculoskeletal and connective tissue disorders	Myalgia<br>Arthralgia<br>Pain in extremity			
General disorders and administration site conditions	Mucosal inflammation<br>Oedema peripheral<br>Pyrexia<br>Fatigue<br>Asthenia	Chills<br>Pain<br>Oedema		

**System organ class**	***Very Common***	***Common***	***Uncommon***	***Rare***
Skin and subcutaneous tissue disorders	Alopecia<br>Rash<br>Nail disorder<br>Pruritus<br>Dry skin			
Musculoskeletal and connective tissue disorders	Myalgia<br>Arthralgia<br>Pain in extremity			
General disorders and administration site conditions	Mucosal inflammation<br>Oedema peripheral<br>Pyrexia<br>Fatigue<br>Asthenia	Chills<br>Pain<br>Oedema		

2.4 Special Populations

Pregnancy:

Refer to appendix 1. No fetal studies in humans have been performed but Perjeta caused oligohydramnios, delayed renal development, and embryo-fetal deaths in pregnant cynomolgus monkeys. Moreover, in the post-marketing setting, cases of oligohydramnios, some associated with fatal pulmonary hypoplasia of the fetus, have been reported in pregnant women receiving Herceptin (for further details, see Herceptin Prescribing Information).Therefore, Perjeta should not be used in pregnant women.

Nursing Mothers

It is not known whether pertuzumab is excreted in human milk. As human IgG1 is secreted into human milk, and the potential for harm to the infant is unknown, Perjeta should not be administered to nursing women.

Geriatric Patients

No dedicated studies of Perjeta have been conducted in elderly patients. In a population PK analysis, age was not found to significantly affect PK of pertuzumab. In the population PK analysis, 32.5% (n=143) patients were ≥ 65 years of age and 9.1% (n=40) patients were ≥ 75 years of age (DSR number 1091765). Based on the findings from Drug safety report 1091765 (a pooled analysis of the safety in patients ≥ 65 and ≤65 years of age from CLEOPATRA, NEOSPHERE, TRYPHAENA, and APHINITY, evidence from literature, and evidence from the company safety database), the incidence of the following all grade adverse events was at least 5% higher in patients aged ≥ 65 years of age, compared to patients aged <65 years of age treated with Perjeta: decreased appetite, anemia, weight decreased, asthenia, dysgeusia, neuropathy peripheral, hypomagnesemia and diarrhea.

Renal Impairment

No formal PK study has been conducted in patients with renal impairment. Based on the population PK analysis, renal impairment is not expected to influence pertuzumab exposure; however, only limited data from patients with moderate and severe renal impairment were includd in the population PK analysis.

Hepatic Impairment

The safety and efficacy of Perjeta has not been studied in patients with hepatic impairment.

2.5 Concomitant Use with other medications

A sub-study in 37 patients in the pivotal trial WO20698/TOC4129g (CLEOPATRA) showed no evidence of drug drug interaction between pertuzumab and trastuzumab and between pertuzumab and docetaxel. In addition, no clinical relevant pharmacokinetic interaction of co-administered docetaxel or trastuzumab on pertuzumab was evident, based on the population pharmacokinetics analysis. The lack of DDI between pertuzumab and trastuzumab and between pertuzumab and docetaxel was further confirmed by pharmacokinetic data from the Study WO209697 (NEOSPHERE).

Five studies have evaluated the effects of pertuzumab on the pharmacokinetics of coadministered cytotoxic agents, docetaxel, paclitaxel, gemcitabine, capecitabine, carboplatin, and erlotinib. There was no evidence of any PK interaction between pertuzumab and any of these agents. The pharmacokinetics of pertuzumab in these studies was comparable to those observed in single agent studies.

2.6 Overdose

Safety in Phase I Studies / Information on MTD

There is no experience with overdosage in human clinical trials. Single doses higher than 25 mg/kg (1727 mg) have not been tested.

3 ZELBORAF (VEMURAFENIB)

Investigators should also consult the local prescribing information if the IMP is approved in your country.

3.1 Standard dose

The recommended dose of vemurafenib is 960 mg (4 tablets of 240 mg) twice daily (equivalent to a total daily dose of 1,920 mg). Vemurafenib may be taken with or without food, but consistent intake of both daily doses on an empty stomach should be avoided. Treatment with vemurafenib should continue until disease progression or the development of unacceptable toxicity. If a dose is missed, it can be taken up to 4 hours prior to the next dose to maintain the twice daily regimen. Both doses should not be taken at the same time. In case of vomiting after vemurafenib administration the patient should not take an additional dose of the medicinal product but the treatment should be continued as usual. The tablets are to be swallowed whole with water. They should not be chewed or crushed.

3.2 Contraindications

Hypersensitivity to the active substance or to any of the excipients.

3.3 Warnings and Precautions

Management of adverse drug reactions or QTc prolongation may require dose reduction, temporary interruption and/or treatment discontinuation (see tables 1 and 2). Posology adjustments resulting in a dose below 480 mg twice daily are not recommended. In the event the patient develops Cutaneous Squamous Cell Carcinoma (cuSCC), it is recommended to continue the treatment without modifying the dose of vemurafenib.

Table 1: Dose modification schedule based on the grade of any Adverse Events (AEs)

**Grade (CTC-AE)** [(a)]	**Recommended dose modification**
**Grade 1 or Grade 2 (tolerable)**	Maintain vemurafenib at a dose of 960 mg twice daily.
**Grade 2 (intolerable) or Grade 3**	
1st occurrence of any grade 2 or 3 AE	Interrupt treatment until grade 0 – 1. Resume dosing at 720 mg twice daily (or 480 mg twice daily if the dose has already been lowered).
2nd occurrence of any grade 2 or 3 AE or persistence after treatment interruption	Interrupt treatment until grade 0 – 1. Resume dosing at 480 mg twice daily (or discontinue permanently if the dose has already been lowered to 480 mg twice daily).
3rd occurrence of any grade 2 or 3 AE or persistence after 2nd dose reduction	Discontinue permanently.
**Grade 4**	
1st occurrence of any grade 4 AE	Discontinue permanently or interrupt vemurafenib treatment until grade 0 – 1.<br>Resume dosing at 480 mg twice daily (or discontinue permanently if the dose has already been lowered to 480 mg twice daily).
2nd occurrence of any grade 4 AE or persistence of any grade 4 AE after 1st dose reduction	Discontinue permanently.

(a) The intensity of clinical adverse events graded by the Common Terminology Criteria for Adverse Events v4.0 (CTC-AE)

Table 2: Dose modification schedule based on prolongation of the QT interval

**QTc value**	**Recommended dose modification**

QTc>500 ms at baseline	Treatment not recommended.
QTc increase meets values of both >500 ms and >60 ms change from pre-treatment values	Discontinue permanently.
1st occurrence of QTc>500 ms during treatment and change from pre-treatment value remains <60 ms	Temporarily interrupt treatment until QTc decreases below 500 ms.<br><br>Resume dosing at 720 mg twice daily (or 480 mg twice daily if the dose has already been lowered).
2nd occurrence of QTc>500 ms during treatment and change from pre-treatment value remains <60 ms	Temporarily interrupt treatment until QTc decreases below 500 ms.<br><br>Resume dosing at 480 mg twice daily (or discontinue permanently if the dose has already been lowered to 480 mg twice daily).
3rd occurrence of QTc>500 ms during treatment and change from pre-treatment value remains <60 ms	Discontinue permanently.

Hypersensitivity reaction

Serious hypersensitivity reactions, including anaphylaxis have been reported in association with vemurafenib. Severe hypersensitivity reactions may include Stevens-Johnson syndrome, generalised rash, erythema or hypotension. In patients who experience severe hypersensitivity reactions, vemurafenib treatment should be permanently discontinued.

Dermatologic reactions

Severe dermatologic reactions have been reported in patients receiving vemurafenib, including rare cases of Stevens-Johnson syndrome and toxic epidermal necrolysis in the pivotal clinical trial. Drug reaction with eosinophilia and systemic symptoms (DRESS) has been reported in association with vemurafenib in the post-marketing setting. In patients who experience a severe dermatologic reaction, vemurafenib treatment should be permanently discontinued.

Potentiation of radiation toxicity

Cases of radiation recall and radiation sensitization have been reported in patients treated with radiation either prior, during, or subsequent to vemurafenib treatment. Most cases were cutaneous in nature but some cases involving visceral organs had fatal outcomes. Vemurafenib should be used with caution when given concomitantly or sequentially with radiation treatment.

QT prolongation

Exposure-dependent QT prolongation was observed in an uncontrolled, open-label phase II study in previously treated patients with metastatic melanoma. QT prolongation may lead to an increased risk of ventricular arrhythmias including Torsade de Pointes. Treatment with vemurafenib is not recommended in patients with uncorrectable electrolyte abnormalities (including magnesium), long QT syndrome or who are taking medicinal products known to prolong the QT interval. Electrocardiogram (ECG) and electrolytes (including magnesium) must be monitored in all patients before treatment with vemurafenib, after one month of treatment and after dose modification. Further monitoring is recommended in particular in patients with moderate to severe hepatic impairment monthly during the first 3 months of treatment followed by every 3 months thereafter or more often as clinically indicated. Initiation of treatment with vemurafenib is not recommended in patients with QTc>500 milliseconds (ms). If during treatment the QTc exceeds 500 ms, vemurafenib treatment should be temporarily interrupted,

electrolyte abnormalities (including magnesium) should be corrected, and cardiac risk factors for QT prolongation (e.g. congestive heart failure, bradyarrhythmias) should be controlled. Re-initiation of treatment should occur once the QTc decreases below 500 ms and at a lower dose as described in table 2. Permanent discontinuation of vemurafenib treatment is recommended if the QTc increase meets values of both >500 ms and >60 ms change from pre-treatment values. (Table 2)

Ophthalmologic reactions
Serious ophthalmologic reactions, including uveitis, iritis and retinal vein occlusion, have been reported. Monitor patients routinely for ophthalmologic reactions.

Cutaneous Squamous Cell Carcinoma (cuSCC)
Cases of cuSCC (which include those classified as keratoacanthoma or mixed keratoacanthoma subtype) have been reported in patients treated with vemurafenib. It is recommended that all patients receive a dermatologic evaluation prior to initiation of therapy and be monitored routinely while on therapy. Any suspicious skin lesions should be excised, sent for dermatopathologic evaluation and treated as per local standard of care. The prescriber should examine the patient monthly during and up to six months after treatment for cuSCC. In patients who develop cuSCC, it is recommended to continue the treatment without dose adjustment. Monitoring should continue for 6 months following discontinuation of vemurafenib or until initiation of another anti -neoplastic therapy. Patients should be instructed to inform their physicians upon the occurrence of any skin changes.

Non-Cutaneous Squamous Cell Carcinoma (non-cuSCC)
Cases of non-cuSCC have been reported in clinical trials where patients received vemurafenib. Patients should undergo a head and neck examination, consisting of at least a visual inspection of oral mucosa and lymph node palpation prior to initiation of treatment and every 3 months during treatment. In addition, patients should undergo a chest Computerised Tomography (CT) scan, prior to treatment and every 6 months during treatment._Anal examinations and pelvic examinations (for women) are recommended before and at the end of treatment or when considered clinically indicated. Following discontinuation of vemurafenib, monitoring for non-cuSCC should continue for up to_6 months or until initiation of another anti-neoplastic therapy. Abnormal findings should be managed according to clinical practices.

New primary melanoma
New primary melanomas have been reported in clinical trials. Cases were managed with excision and patients continued treatment without dose adjustment. Monitoring for skin lesions should occur as outlined above for cutaneous squamous cell carcinoma.

Other malignancies
Based on mechanism of action, vemurafenib may cause progression of cancers associated with RAS mutations. Carefully consider benefits and risks before administering vemurafenib to patients with a prior or concurrent cancer associated with RAS mutation.

Pancreatitis
Pancreatitis has been reported in vemurafenib-treated subjects. Unexplained abdominal pain should be promptly investigated (including measurement of serum amylase and lipase). Patients should be closely monitored when re-starting vemurafenib after an episode of pancreatitis.

Liver injury
Liver injury, including cases of severe liver injury, has been reported with vemurafenib. Liver enzymes (transaminases and alkaline phosphatase) and bilirubin should be measured before initiation of treatment and monitored monthly during treatment, or as clinically indicated. Laboratory abnormalities should be managed with dose reduction, treatment interruption or with treatment discontinuation.

Renal toxicity

Renal toxicity, ranging from serum creatinine elevations to acute interstitial nephritis and acute tubular necrosis, has been reported with vemurafenib. Serum creatinine should be measured before initiation of treatment and monitored during treatment as clinically indicated.

Photosensitivity

Mild to severe photosensitivity was reported in patients who received vemurafenib in clinical studies. All patients should be advised to avoid sun exposure while taking vemurafenib. While taking the medicinal product, patients should be advised to wear protective clothing and use a broad spectrum Ultraviolet A (UVA)/Ultraviolet B (UVB) sunscreen and lip balm (Sun Protection Factor ≥ 30) when outdoors to help protect against sunburn._For photosensitivity grade 2 (intolerable) or greater, dose modifications are recommended.

Dupuytren's contracture and plantar fascial fibromatosis

Dupuytren's contracture and plantar fascial fibromatosis have been reported with vemurafenib. The majority of cases were mild to moderate, but severe, disabling cases of Dupuytren's contracture have also been reported._Events should be managed with dose reduction with treatment interruption or with treatment discontinuation.

3.4 Special Populations

Elderly

No special dose adjustment is required in patients aged > 65 years old.

Renal impairment

Limited data are available in patients with renal impairment. A risk for increased exposure in patients with severe renal impairment cannot be excluded. Patients with severe renal impairment should be closely monitored.

Hepatic impairment

Limited data are available in patients with hepatic impairment. As vemurafenib is cleared by the liver, patients with moderate to severe hepatic impairment may have increased exposure and should be closely monitored.

Reproductive and Developmental Toxicity, Pregnancy

Refer to appendix 1.

3.5 Concomitant use with other medications

Effects of vemurafenib on Drug Metabolizing Enzymes

Results from an in vivo drug-drug interaction study in metastatic melanoma patients demonstrated that vemurafenib is a moderate CYP1A2 inhibitor and a CYP3A4 inducer. Concomitant use of vemurafenib with agents metabolized by CYP1A2 with narrow therapeutic windows (e.g. agomelatine, alosetron, duloxetine, melatonin, ramelteon, tacrine, tizanidine, theophylline) is not recommended. If co-administration cannot be avoided, exercise caution, as vemurafenib may increase plasma exposure of CYP1A2 substrate drugs. Dose reduction of the concomitant CYP1A2 substrate drug may be considered, if clinically indicated. Co-administration of vemurafenib increased the plasma exposure (AUC) of caffeine (CYP1A2 substrate) 2.6-fold. In another clinical trial, vemurafenib increased Cmax and AUC of a single 2 mg dose of tizanidine (CYP1A2 substrate) approximately 2.2-fold and 4.7-fold, respectively.

Concomitant use of vemurafenib with agents metabolized by CYP3A4 with narrow therapeutic windows is not recommended. If co-administration cannot be avoided, it needs to be considered that vemurafenib may decrease plasma concentrations of CYP3A4 substrates and thereby their efficacy may be impaired. On this basis, the efficacy of contraceptive pills metabolized by CYP3A4 used concomitantly with vemurafenib might be decreased. Dose adjustments for CYP3A4 substrates with narrow therapeutic window may be considered, if clinically indicated.

In a clinical trial, co-administration of vemurafenib decreased the AUC of midazolam (CYP3A4 substrate) by an average 39% (maximum decrease up to 80%).

Mild induction of CYP2B6 by vemurafenib was noted in vitro at a vemurafenib concentration of 10 µM. It is currently unknown whether vemurafenib at a plasma level of 100 µM observed in patients at steady state (approximately 50 µg/ml) may decrease plasma concentrations of concomitantly administered CYP2B6 substrates, such as bupropion.
Co-administration of vemurafenib resulted in an 18% increase in AUC of S-warfarin (CYP2C9 substrate). Exercise caution and consider additional INR (international normalized ratio) monitoring when vemurafenib is used concomitantly with warfarin.
Vemurafenib moderately inhibited CYP2C8 in vitro. The in vivo relevance of this finding is unknown, but a risk for a clinically relevant effect on concomitantly administered CYP2C8 substrates cannot be excluded. Concomitant administration of CYP2C8 substrates with a narrow therapeutic window should be made with caution since vemurafenib may increase their concentrations.
Due to the long half-life of vemurafenib, the full inhibitory effect of vemurafenib on a concomitant medicinal product might not be observed before 8 days of vemurafenib treatment.
After cessation of vemurafenib treatment, a washout of 8 days might be necessary to avoid an interaction with a subsequent treatment.
Radiation treatment
Potentiation of radiation treatment toxicity has been reported in patients receiving vemurafenib. In the majority of cases, patients received radiotherapy regimens greater than or equal to 2 Gy/day (hypofractionated regimens).

Effects of vemurafenib on drug transport systems
In vitro studies have demonstrated that vemurafenib is an inhibitor of the efflux transporters P-glycoprotein (P-gp) and breast cancer resistance protein (BCRP).

A clinical drug interaction study demonstrated that multiple oral doses of vemurafenib (960 mg twice daily) increased the exposure of a single oral dose of the P-gp substrate digoxin, approximately 1.8 and 1.5 fold for digoxin AUClast and Cmax, respectively. Caution should be exercised when dosing vemurafenib concurrently with P-gp substrates (e.g. aliskiren, ambrisentan, colchicine, dabigatran etexilate, digoxin, everolimus, fexofenadine, lapatinib, maraviroc, nilotinib, posaconazole, ranolazine, sirolimus, sitagliptin, talinolol, topotecan) and dose reduction of the concomitant medicinal product may be considered, if clinically indicated. Consider additional drug level monitoring for P-gp substrate medicinal products with a narrow therapeutic index (NTI) (e.g. digoxin, dabigatran etexilate, aliskiren). The effects of vemurafenib on medicinal products that are substrates of BCRP are unknown. It cannot be excluded that vemurafenib may increase the exposure of medicines transported by BCRP (e.g. methotrexate, mitoxantrone, rosuvastatin). Many anticancer medicinal products are substrates of BCRP and therefore there is a theoretical risk for an interaction with vemurafenib.
The possible effect of vemurafenib on other transporters is currently unknown.

Effects of concomitant medicines on vemurafenib
In vitro studies suggest that CYP3A4 metabolism and glucuronidation are responsible for the metabolism of vemurafenib. Biliary excretion appears to be another important elimination pathway. In vitro studies have demonstrated that vemurafenib is a substrate of the efflux transporters P-gp and BCRP. It is currently unknown whether vemurafenib is a substrate also to other transport proteins. Concomitant administration of strong CYP3A4 inhibitors or inducers or inhibitors/inducer of transport protein activity may alter vemurafenib concentrations. Co-administration of itraconazole, a strong CYP3A4/Pgp inhibitor, increased steady state vemurafenib AUC by approximately 40%. Vemurafenib should be used with caution in combination with strong inhibitors of CYP3A4, glucuronidation and/or transport proteins (e.g. ritonavir, saquinavir, telithromycin, ketoconazole, itraconazole, voriconazole, posaconazole, nefazodone, atazanavir). Patients co-treated with such agents should be carefully monitored for safety and dose modifications applied if clinically indicated. In a clinical study, co-administration of a single dose 960 mg of vemurafenib with rifampicin, significantly decreased the plasma exposure of vemurafenib by approximately 40%. Concomitant administration of strong inducers of P-gp, glucuronidation, and/or CYP3A4 (e.g. rifampicin,

rifabutin, carbamazepine, phenytoin or St John's Wort [Hypericum perforatum]) may lead to suboptimal exposure to vemurafenib and should be avoided.
The effects of P-gp and BCRP inhibitors that are not also strong CYP3A4 inhibitors are unknown. It cannot be excluded that vemurafenib pharmacokinetics could be affected by such medicines through influence on P-gp (e.g. verapamil, cyclosporine, quinidine) or BCRP (e.g. cyclosporine, gefitinib).

3.6 Overdose

There is no specific antidote for overdose of vemurafenib. Patients who develop adverse reactions should receive appropriate symptomatic treatment. No cases of overdose have been observed with vemurafenib in clinical trials. In case of suspected overdose, vemurafenib should be withheld and supportive care initiated.

4 KADCYLA (TRASTUZUMAB EMTANSINE)

Investigators should also consult the local prescribing information if the IMP is approved in your country.

4.2 Standard dose

The recommended dose of trastuzumab emtansine is 3.6 mg / kg body weight, administered by intravenous infusion every 3 weeks (21 day cycle). The initial dose should be administered as a 90 minute intravenous infusion. Patients should be observed during the infusion and for at least 90 minutes following the initial infusion for fever, chills, or other infusion-related reactions. The infusion site should be closely monitored for possible subcutaneous infiltration during administration. If the prior infusion was well tolerated, subsequent doses of trastuzumab emtansine may be administered as 30 minute infusions. Patients should be observed during the infusion and for at least 30 minutes after infusion.

4.3 Contraindications

Trastuzumab emtansine is contraindicated in patients with a known hypersensitivity to trastuzumab emtansine or any of the excipients.

4.4 Warnings and Precautions

Patients treated with trastuzumab emtansine must have confirmed HER2-positive tumor status as assessed by either HER2 protein over-expression or gene amplification.

Pulmonary Toxicity

Cases of ILD, including pneumonitis, some leading to acute respiratory distress syndrome or a fatal outcome, have been reported in clinical trials with trastuzumab emtansine. Signs and symptoms include dyspnea, cough, fatigue, and pulmonary infiltrates. It is recommended that treatment with trastuzumab emtansine be permanently discontinued in patients who are diagnosed with ILD or pneumonitis, except for radiation pneumonitis in the adjuvant setting, where trastuzumab emtansine should be permanently discontinued for ≥ Grade 3 or for Grade 2 not responding to standard treatment. Patients with dyspnea at rest due to complications of advanced malignancy, comorbidities and receiving concurrent pulmonary radiation therapy may be at increased risk of pulmonary events.

Hepatotoxicity

Hepatotoxicity, predominantly in the form of asymptomatic increases in the concentrations of serum transaminases (Grade 1-4 transaminitis), has been observed while on treatment with trastuzumab emtansine in clinical trials. Transaminase elevations were generally transient with peak elevation at day 8 after therapy and subsequent recovery to Grade 1 or less prior to the next cycle. A cumulative effect of trastuzumab emtansine on transaminases has also been observed. Patients with elevated transaminases improved to Grade 1 or normal within 30 days of the last dose of trastuzumab emtansine in the majority of the cases. Serious hepatobiliary disorders, including NRH of the liver and some with a fatal outcome due to drug-induced liver injury have been observed in patients treated with trastuzumab emtansine in clinical trials. Observed cases may have been confounded by comorbidities and/or concomitant medications with known hepatotoxic potential. Liver function should be monitored prior to initiation of treatment and each trastuzumab emtansine dose. Trastuzumab emtansine has not been studied in patients with serum transaminases > 2.5 x upper limit of normal (ULN) or total bilirubin > 1.5 x ULN prior to initiation of treatment. Trastuzumab emtansine treatment in patients with serum transaminases > 3 x ULN and concurrent total bilirubin > 2 x ULN should be permanently discontinued.

Cases of NRH of the liver have been identified from liver biopsies in patients treated with trastuzumab emtansine. NRH is a rare liver condition characterized by widespread benign transformation of hepatic parenchyma into small regenerative nodules; NRH may lead to non-cirrhotic portal hypertension. Diagnosis of NRH can be confirmed only by histopathology. NRH should be considered in all patients with clinical symptoms of portal hypertension and/or cirrhosis-like pattern seen on the computed tomography (CT) scan

of the liver but with normal transaminases and no other manifestations of cirrhosis. Upon diagnosis of NRH, trastuzumab emtansine treatment must be permanently
discontinued.

Left Ventricular Dysfunction

Patients treated with trastuzumab emtansine are at increased risk of developing left ventricular dysfunction. Left ventricular ejection fraction ≤ 40% has been observed in patients treated with trastuzumab emtansine and therefore symptomatic CHF is a potential risk. Standard cardiac function testing (ECHO or MUGA scanning) should be performed prior to initiation and at regular intervals (e.g. every three months) during treatment with trastuzumab emtansine. Treatment with trastuzumab emtansine has not been studied in patients with LVEF < 50% prior to initiation of treatment.

Infusion-related Reactions

Treatment with trastuzumab emtansine has not been studied in patients who had
trastuzumab permanently discontinued due to IRRs/hypersensitivity; treatment with trastuzumab emtansine is not recommended for these patients. Infusion-related reactions, characterized by one or more of the following symptoms - flushing, chills, pyrexia, dyspnea, hypotension, wheezing, bronchospasm, and tachycardia - have been reported in clinical trials of trastuzumab emtansine. In general, these symptoms were not severe. In most patients, these reactions resolved over the course of several hours to a day after the infusion was terminated.
Trastuzumab emtansine treatment should be interrupted in patients with severe IRR. Trastuzumab emtansine treatment should be permanently discontinued in the event of a life threatening IRR.

Hypersensitivity Reactions

Patients should be observed closely for hypersensitivity reactions, especially during the first infusion. Hypersensitivity, including serious anaphylactic-like reactions, has been observed in clinical trials with treatment of trastuzumab emtansine. Medications to treat such reactions, as well as emergency equipment, should be available for immediate use.

Hemorrhage

Cases of hemorrhagic events, including central nervous system, respiratory, and
gastrointestinal hemorrhage, have been reported with trastuzumab emtansine. Some of these bleeding events resulted in fatal outcomes. In some of the observed cases the patients were also receiving anti-coagulation therapy, antiplatelet therapy, or had thrombocytopenia; in others there were no known additional risk factors. Caution should be used with these agents and consider additional monitoring when concomitant use with trastuzumab emtansine is medically necessary.

Thrombocytopenia

Thrombocytopenia, or decreased platelet counts, was reported in patients in clinical trials of trastuzumab emtansine. The majority of these patients had Grade 1 or 2 events (≥ 50,000/mm3), with the nadir occurring by day 8 and generally improving to Grade 0 or 1 (≥75,000/mm3) by the next scheduled dose. In clinical trials, the incidence and severity of thrombocytopenia were higher in Asian patients. Patients with thrombocytopenia (≤ 100,000/mm3) and patients on anti-coagulant treatment should be monitored closely while on treatment with trastuzumab emtansine. It is recommended that platelet counts are monitored prior to each trastuzumab emtansine dose. Trastuzumab emtansine has not been studied in patients with platelet counts ≤ 100,000/mm3 prior to initiation of treatment. In the event of decreased platelet count to Grade 3 or greater (≤ 50,000/mm3), do not administer trastuzumab emtansine until platelet counts recover to Grade 1 (≥75,000/mm3).

Neurotoxicity

Peripheral neuropathy, mainly Grade 1 and predominantly sensory, has been reported in clinical trials of trastuzumab emtansine. Treatment with trastuzumab emtansine should be temporarily discontinued in

patients experiencing Grade 3 or 4 peripheral neuropathy until symptoms resolve or improve to ≤ Grade 2. Patients should be clinically monitored on an ongoing basis for signs/symptoms of neurotoxicity.

Extravasation

In trastuzumab emtansine clinical studies, reactions secondary to extravasation have been observed. These reactions were usually mild and comprised erythema,
tenderness, skin irritation, pain, or swelling at the infusion site. These reactions have been observed more frequently within 24 hours of infusion. Specific treatment for trastuzumab emtansine extravasation is unknown at this time. The infusion site should be closely monitored for possible subcutaneous infiltration during drug administration.

Table 1 Dose Modification Guidelines

Dose Modifications for Patients with MBC		
**Adverse reaction**	**Severity**	**Treatment modification**
Thrombocytopenia	Grade 3<br>(25,000 to<br>< 50,000/mm^3)	Do not administer trastuzumab emtansine until platelet count recovers to £ Grade 1 (≥ 75,000/mm^3), and then treat at the same dose level
	Grade 4<br>(< 25,000/mm^3)	Do not administer trastuzumab emtansine until platelet count recovers to £ Grade 1 (≥ 75,000/mm^3), and then reduce one dose level
Increased Transaminase (AST/ALT)	Grade 2<br>(> 2.5 to ≤ 5´ the ULN)	Treat at the same dose level
	Grade 3<br>(> 5 to ≤ 20´ the ULN)	Do not administer trastuzumab emtansine until AST/ALT recovers to Grade ≤ 2, and then reduce one dose level
	Grade 4<br>(> 20´ the ULN)	Discontinue trastuzumab emtansine
Hyperbilirubinemia	Grade 2<br>(> 1.5 to ≤ 3´ the ULN)	Do not administer trastuzumab emtansine until total bilirubin recovers to Grade ≤ 1, and then treat at the same dose level.
	Grade 3<br>(> 3 to ≤ 10´ the ULN)	Do not administer trastuzumab emtansine until total bilirubin recovers to Grade ≤ 1 and then reduce one dose level.
	Grade 4<br>(> 10´ the ULN)	Discontinue trastuzumab emtansine
Drug Induced Liver Injury (DILI)	Serum transaminases > 3 x ULN and concomitant total bilirubin > 2´ ULN	Permanently discontinue trastuzumab emtansine in the absence of another likely cause for the elevation of liver enzymes and bilirubin, e.g. liver metastasis or concomitant medication
Left Ventricular	Symptomatic CHF	Discontinue trastuzumab emtansine
	LVEF <40%	Do not administer trastuzumab emtansine. Repeat LVEF assessment within 3 weeks. If LVEF <40% is confirmed, discontinue trastuzumab emtansine
	LVEF 40% to ≤ 45%	Do not administer trastuzumab emtansine.

	and decrease is ≥ 10% points from baseline	Repeat LVEF assessment within 3 weeks. If the LVEF has not recovered to within 10% points from baseline, discontinue trastuzumab emtansine.
	LVEF 40% to ≤ 45% and decrease is < 10% points from baseline	Continue treatment with trastuzumab emtansine. Repeat LVEF assessment within 3 weeks.
	LVEF > 45%	Continue treatment with trastuzumab emtansine.

Table 2 Dose reduction schedule

**Dose reduction schedule**<br><br>**(Starting dose is 3.6 mg/kg)**	**Dose to be administered**
First dose reduction	3 mg/kg
Second dose reduction	2.4 mg/kg
Requirement for further dose reduction	Discontinue treatment

4.4 Special Populations

Pregnancy, Females and Males of Reproductive Potential

Refer to appendix 1. No clinical studies of trastuzumab emtansine in pregnant women have been performed. Trastuzumab emtansine is not recommended to be administered to pregnant women.

Nursing Mothers

It is not known whether trastuzumab emtansine is excreted in human milk. Since many drugs are excreted in human milk and because of the potential for serious adverse drug reactions in nursing infants from trastuzumab emtansine, women should discontinue nursing prior to initiating treatment with trastuzumab emtansine. Women may begin nursing 7 months following the last dose of trastuzumab emtansine.

Geriatric Patients

There are insufficient data to establish the safety of trastuzumab emtansine in patients 75 years of age or older.

Renal Impairment

The population pharmacokinetic analysis of trastuzumab emtansine showed that creatinine clearance does not affect pharmacokinetics of trastuzumab emtansine.

Pharmacokinetics of trastuzumab emtansine in patients with mild (CLcr 60 [illegible]89 mL/min, n [illegible]254) or moderate (CLcr 30 [illegible]59 mL/min, n [illegible]53) renal impairment were similar to those in patients with normal renal function (CLcr [illegible]90 mL/min, n [illegible]361).

No dose adjustments to the starting dose of trastuzumab emtansine are therefore required in these patients. Pharmacokinetic data in patients with severe renal impairment (CLcr 15 [illegible]29 mL/min) is limited (n [illegible]1), therefore no dosage recommendations can be made. The potential need for dose adjustment in patients with severe renal impairment cannot be determined due to insufficient data

Hepatic Impairment

Study BO25499 included MBC patients with normal hepatic function (n [illegible]10) and with mild hepatic dysfunction (Child-Pugh Class A) (n [illegible]10) and moderate hepatic dysfunction (Child-Pugh Class B) (n [illegible]8). The results demonstrated that the systemic exposures (AUC) of trastuzumab emtansine at Cycle 1 in patients with mild and moderate hepatic impairment were approximately 38% and 67% lower than that of patients with normal hepatic function, respectively. Trastuzumab emtansine exposure (AUC) at Cycle 3 after repeated dosing in patients with mild or moderate hepatic dysfunction was within the range observed in patients with normal hepatic function. Plasma concentrations of DM1 and DM1-containing catabolites were comparable across all three cohorts, indicating that hepatic impairment did not impact the PK of DM1 and

DM1-containing catabolites in a clinically meaningful way. No increase in systemic DM1 concentrations was observed in patients with mild or moderate hepatic dysfunction. Therefore, no dose adjustments are required for patients with mild or moderate hepatic impairment at starting dose. The incidence of high grade AEs and hepatotoxicity was higher in patients with hepatic impairment compared with patients with normal hepatic function. Therefore, treatment with caution is warranted.
Trastuzumab emtansine has not been studied in patients with severe hepatic impairment (Child-Pugh class C). In patients with moderate or severe hepatic impairment, close clinical monitoring for safety and efficacy is advised. The potential need for dose adjustment in patients with severe renal impairment cannot be determined due to insufficient data.
Based on a population PK analysis (n [illegible]671), although the liver function related parameters albumin and AST were identified as statistically significant covariates for trastuzumab emtansine pharmacokinetics, the magnitude of their effect on trastuzumab emtansine exposure suggests they are unlikely to have any clinically meaningful effect on trastuzumab emtansine exposure.
Liver function should be monitored prior to initiation of treatment and each trastuzumab emtansine dose. Trastuzumab emtansine has not been studied in patients with serum transaminases >2.5 [illegible]ULN or total bilirubin >1.5 [illegible]ULN prior to initiation of treatment. Trastuzumab emtansine treatment in patients with serum transaminases >3x ULN and concomitant total bilirubin >2x ULN should be permanently discontinued.

4.5 Concomitant use with other medications

No formal drug-drug interaction studies with trastuzumab emtansine in humans have been conducted. In vitro metabolism studies in human liver microsomes suggest that DM1, a component of trastuzumab emtansine, is metabolized mainly by CYP3A4 and, to a lesser extent, by CYP3A5. DM1 does not induce or inhibit P450-mediated metabolism in vitro. Caution should be taken when trastuzumab emtansine is co-administered with potent CYP3A inhibitors.
Trastuzumab emtansine has been studied in combination with taxanes, namely docetaxel (Study BP22572) and paclitaxel (Study TDM4652g) and separately with pertuzumab (Study TDM4788g/BO22589), a monoclonal antibody that blocks the association of HER2 with other HER family members, including EGFR, HER3, and HER4. PK analyses show that co-administration of docetaxel, paclitaxel, and pertuzumab do not appear to affect the pharmacokinetics of trastuzumab emtansine or DM1. Further, the pharmacokinetics of docetaxel, paclitaxel, and pertuzumab are similar with or without co-administration of trastuzumab emtansine.
A Phase I Study (GDC4627) studied the combination of trastuzumab emtansine with a phosphoinositide (PI3K) inhibitor, pictilisib. Co-administration of trastuzumab emtansine or trastuzumab had no meaningful impact on pictilisib PK. Similarly, pictilisib did not appear to alter the pharmacokinetics of trastuzumab or trastuzumab emtansine.

4.6 Overdose

There is no known antidote for trastuzumab emtansine overdose. In case of overdose, the patient should be closely monitored. Cases of overdose due to medication error have been reported with trastuzumab emtansine treatment, most associated with thrombocytopenia, and there was one death. In the fatal case, the patient incorrectly received trastuzumab emtansine 6 mg/kg and died approximately 3 weeks following the medication error; a cause of death and a causal relationship to trastuzumab emtansine were not established.

5 ALECENSA (ALECTINIB)

Investigators should also consult the local prescribing information if the IMP is approved in your country.

5.2 Approved Indications

The recommended dose of Alecensa is 600 mg (4 x 150 mg capsules) to be taken twice a day at meals (total daily dose of 1200 mg).
Patients with pre-existing severe hepatic impairment (Child-Pugh C) should receive an initial dose of 450 mg twice daily at meals (total daily dose of 900 mg). Treatment with Alecensa should be continued until disease progression or unacceptable toxicity. If a planned dose of Alecensa is missed, patients can make up that dose unless the next dose is due within 6 hours. Patients should not take two doses at the same time to make up for a missed dose. If vomiting occurs after taking a dose of Alecensa, patients should take the next dose at the scheduled time.

5.3 Contraindications

Alectinib is contraindicated in patients with a known hypersensitivity to alectinib or any of the excipients.

5.4 Warnings and Precautions

Interstitial Lung Disease/Pneumonitis

Cases of ILD/pneumonitis have been reported in clinical trials with alectinib. Patients should be monitored for pulmonary symptoms indicative of pneumonitis. Alectinib should be immediately interrupted in patients diagnosed with ILD/pneumonitis and should be permanently discontinued if no other potential causes of ILD/pneumonitis have been identified.

Hepatotoxity

Elevations in ALT and AST greater than 5 times the ULN as well as bilirubin elevations of more than 3 times the ULN occurred in patients in clinical trials with alectinib. A time-to-event analysis based on data from the pivotal alectinib clinical
studies (NP28673, NP28761, and BO28984) showed that the majority of transaminase and bilirubin elevations occurred during the first 3 months of treatment (NP28673 and NP28761: 76% of the patients with hepatic transaminase elevations and 68% of the patients with bilirubin elevations; BO28984: 56% of the patients with hepatic transaminase elevations and 69% of the patients with bilirubin elevations). In the pivotal alectinib clinical trials (NP28673, NP28761, and BO28984) two patients with Grade 3-4 AST/ALT elevations had documented drug induced liver injury by liver biopsy.
In addition, one patient experienced a Grade 4 adverse event of drug-induced liver injury and another patient experienced Grade 4 hepatotoxicity. Concurrent elevations in ALT or AST greater than or equal to three times the ULN and total bilirubin greater than or equal to two times the ULN, with normal alkaline phosphatase, occurred in 1 patient treated in alectinib clinical trials.
Liver function, including ALT, AST, and total bilirubin should be monitored routinely. Patients should be advised to report any symptoms of hepatotoxicity (e.g., unexplained tiredness, pruritus, decreased appetite, nausea or vomiting, jaundice, pain on the right side of stomach area, dark urine, bleeding or bruising more easily than normal).

Severe Myalgia and CPK Elevations

Myalgia or musculoskeletal pain was reported in patients in pivotal clinical trials with alectinib, including Grade 3 events. Elevation of CPK also occurred in pivotal trials with alectinib, including Grade 3 events. Median time to Grade 3 CPK elevation was 14 days in the pivotal Phase II trials (NP28761, NP28673) and 27.5 days in the pivotal Phase III trial (BO28984).
CPK should be monitored routinely. Patients should be advised to report any unexplained muscle pain, tenderness, or weakness.

Bradycardia

Symptomatic bradycardia can occur with alectinib. Heart rate and blood pressure should be monitored as clinically indicated. In case of bradycardia, concomitant medications have to be evaluated to identify if there are any known medications that could cause bradycardia or any anti-hypertensive medications that could have contributed to the event. In case such concomitant medications are identified, dose reduction or discontinuation should be considered.

Photosensitivity

Photosensitivity to sunlight has been reported with alectinib administration. Patients should be advised to avoid prolonged sun exposure while taking alectinib and for at least 7 days after discontinuation of treatment. Patients should also be advised to use a broad-spectrum Ultraviolet A (UVA)/Ultraviolet B (UVB) sun screen and lip balm (sun protection factor [SPF] ≥50) to help protect against potential sunburn.

General

Management of adverse events may require dose reduction, temporary interruption, or discontinuation of treatment with Alecensa. The dose of Alecensa should be reduced in steps of 150 mg twice daily based on tolerability. Alecensa treatment should be permanently discontinued if patients are unable to tolerate the 300 mg twice daily dose.

Table 1: Dose reduction schedule

Dose reduction schedule	Dose level
Dose	600 mg twice daily
First dose reduction	450 mg twice daily
Second dose reduction	300 mg twice daily

Table 2: Dose modification advice for specified Adverse Drug Reactions

CTCAE grade	Alecensa treatment
ILD/pneumonitis of any severity grade	Immediately interrupt and permanently discontinue Alecensa if no other potential causes of ILD/pneumonitis have been identified.
ALT or AST elevation of Grade ≥ 3 (> 5 times ULN) with total bilirubin ≤ 2 times ULN	Temporarily withhold until recovery to baseline or ≤ Grade 1 (≤ 3 times ULN), then resume at reduced dose
ALT or AST elevation of Grade ≥ 2 (> 3 times ULN) with total bilirubin elevation > 2 times ULN in the absence of cholestasis or haemolysis	Permanently discontinue Alecensa.
Bradycardia[a] Grade 2 or Grade 3 (symptomatic, may be severe and medically significant, medical intervention indicated)	Temporarily withhold until recovery to ≤ Grade 1 (asymptomatic) bradycardia or to a heart rate of ≥ 60 bpm. Evaluate concomitant medicinal products known to cause bradycardia, as well as anti-hypertensive medicinal products.<br><br>If a contributing concomitant medicinal product is identified and discontinued, or its dose is adjusted,

	resume at previous dose upon recovery to ≤ Grade 1 (asymptomatic) bradycardia or to a heart rate of ≥ 60 bpm.<br><br>If no contributing concomitant medicinal product is identified, or if contributing concomitant medicinal products are not discontinued or dose modified, resume at reduced dose (see Table 1) upon recovery to ≤ Grade 1 (asymptomatic) bradycardia or to a heart rate of ≥ 60 bpm.
Bradycardia Grade 4 (life-threatening consequences, urgent intervention indicated)	Permanently discontinue if no contributing concomitant medicinal product is identified.<br>If a contributing concomitant medicinal product is identified and discontinued, or its dose is adjusted, resume at reduced dose (see Table 1) upon recovery to ≤ Grade 1 (asymptomatic) bradycardia or to a heart rate of ≥ 60 bpm, with frequent monitoring as clinically indicated.<br>Permanently discontinue in case of recurrence.
CPK elevation > 5 times ULN	Temporarily withhold until recovery to baseline or to ≤ 2.5 times ULN, then resume at the same dose.
CPK elevation > 10 times ULN or second occurrence of CPK elevation of > 5 times ULN	Temporarily withhold until recovery to baseline or to ≤ 2.5 times ULN, then resume at reduced dose as per Table 1.

ALT = alanine aminotransferase; AST = aspartate aminotransferase; CPK = creatine phosphokinase; CTCAE = NCI Common Terminology Criteria for Adverse Events; ILD = interstitial lung disease; ULN = upper limit of normal

a Heart rate less than 60 beats per minute (bpm).

5.4 Special Populations

Patients with severe renal impairment, children, pregnant women and breastfeeding mothers were excluded from clinical studies to date. Elderly subjects (≥ 65 years) were included in clinical trials.

Pregnancy

Women of child-bearing potential must be advised to avoid pregnancy while on alectinib. No clinical studies of alectinib in pregnant women have been performed. Based on its mechanism of action, alectinib may cause fetal harm when administered to a pregnant woman. Refer to appendix 1.

Nursing Mothers

It is not known whether alectinib is excreted in human breast milk. As many drugs are excreted in human milk and because of the potential harm to the infant, mothers should be advised against breastfeeding while receiving alectinib.

Geriatric Patients

The safety of alectinib has not been specifically tested in a geriatric population.
However, clinical trials with alectinib did not exclude patients > 65 years old. In the

pivotal Phase II studies (NP28761 and NP28673), the pivotal Phase III study ALEX (BO28984) and in Study AF001-JP, a total of 14%, 24% and 9% of the patients, respectively, were ≥ 65 years of age. The overall safety profile was generally consistent between age subgroups (< 65, ≥ 65 years). Age was tested as a covariate in the popPK analysis. For patients aged between 21 and 83 years old, results of the popPK analysis showed that there was no effect of age on the PK of alectinib and M4.

Other

Renal Impairment

No dose adjustment is required in patients with mild or moderate renal impairment. Alectinib has not been studied in patients with severe renal impairment, however since alectinib elimination via the kidney is negligible, no dose adjustment is required in patients with severe renal impairment.

Hepatic Impairment

A study to evaluate alectinib in patients with moderate to severe hepatic impairment showed that moderate and severe hepatic impairment lead to higher systemic exposure of alectinib + M4 compared to matched subjects with normal hepatic function. No dose adjustment is required in patients with mild or moderate hepatic impairment, Study NP29783 CSR]. Patients with severe hepatic impairment should receive a starting dose of 450 mg taken orally twice daily [Study NP29783 CSR].

5.5 Concomitant use with other medications

Effects of Alectinib on Other Drugs

CYP Substrates

In vitro studies indicate that neither alectinib nor its major active metabolite (M4) inhibits CYP1A2, CYP2B6, CYP2C9, CYP2C19, or CYP2D6 at clinically relevant concentrations.

Alectinib and M4 showed weak time-dependent inhibition of CYP3A4. In vitro, alectinib exhibits a weak induction potential of CYP3A4 and CYP2B6 at clinical concentrations.

Results from a clinical DDI study in ALK-positive NSCLC patients demonstrated that multiple doses of alectinib had no influence on the exposure of MDZ, a sensitive CYP3A substrate. Therefore, no dose adjustment is required for co-administered CYP3A substrates.

Although in vitro studies indicate that alectinib is an inhibitor of CYP2C8, PBPK modeling supports that at clinically relevant concentrations alectinib does not have the potential to increase plasma concentrations of co-administered substrates of CYP2C8.

P-gp and BCRP Substrates

In vitro, alectinib and M4 are inhibitors of the efflux transporters P-gp and BCRP.

Therefore, alectinib may have the potential to increase plasma concentrations of co-administered substrates of P-gp or BCRP transporters (the increase in exposure is not expected to be more than 2-fold). When alectinib is co-administered with P-gp or BCRP substrates with narrow therapeutic index (e.g., digoxin, dabigatran, methotrexate), appropriate monitoring is recommended.

Effects of Other Drugs on Alectinib

Based on in vitro data, CYP3A4 is the primary enzyme mediating the metabolism of both alectinib and its major active metabolite M4, and CYP3A contributes to 40% to 50% of total hepatic metabolism. M4 has shown similar in vitro potency and activity to alectinib against ALK.

CYP3A Inducers

Co-administration of multiple oral doses of 600 mg rifampicin once daily, a strong CYP3A inducer, with a single oral dose of 600 mg alectinib exhibited a minor effect on combined exposure of alectinib and M4 (GMR with/without rifampicin [90% CI]: Cmax: 0.96 [0.88 - 1.05], AUCinf: 0.82 [0.74 - 0.90]). Therefore, no dose adjustments are required when alectinib is co-administered with CYP3A inducers.

CYP3A Inhibitors

Co-administration of multiple oral doses of 400 mg posaconazole BID, a strong CYP3A inhibitor, with a single oral dose of 300 mg alectinib had a minor effect on combined exposure of alectinib and M4 (GMR with/without posaconazole [90% CI]: Cmax: 0.93 [0.81 – 1.08], AUCinf: 1.36 [1.24 - 1.49]). Therefore, no dose adjustments are required when alectinib is co-administered with CYP3A inhibitors.

Medicinal Products that Increase Gastric pH

Although the aqueous solubility of alectinib in vitro is pH-dependent, a dedicated clinical DDI study with 40 mg esomeprazole once daily, a PPI, demonstrated no clinically relevant effect on the combined exposure of alectinib and M4. Therefore, no dose adjustments are required when alectinib is co-administered with PPIs or other drugs which raise gastric pH (e.g., H2 receptor antagonists or antacids).

Effect of Transporters on Alectinib Disposition

Based on in vitro data, alectinib is not a substrate of P-gp. Alectinib and M4 are not substrates of BCRP or OATP 1B1/B3. In contrast, M4 is a substrate of P-gp. alectinib inhibits P-gp, and therefore, it is not expected that co-medication with P-gp inhibitors will have a relevant effect on M4 exposure.

Drug Abuse and Dependence

No data to report.

Ability to Drive and Use Machines

No studies on the effects on the ability to drive and to use machines have been performed.

5.6 Overdose

No experience with overdosage is available from the pivotal clinical trials. Patients who experience overdose should be closely supervised and supportive care instituted. There is no specific antidote for overdose with alectinib.

6 ERIVEDGE (VISMODEGIB)

Investigators should also consult the local prescribing information if the IMP is approved in your country.

6.1 Standard dose:

The recommended dose is one 150 mg capsule per day. Erivedge is for oral use.The capsules must be swallowed whole with water, with or without food. The capsules must not be opened, to avoid unintended exposure to patients and health care providers. If a dose is missed, patients should be instructed not to take the missed dose but to resume with the next scheduled dose. In clinical trials, treatment with Erivedge was continued until disease progression or until unacceptable toxicity. Treatment interruptions of up to 4 weeks were allowed based on individual tolerability.

6.2 Contraindications

- Vismodegib is contraindicated in nursing mothers during the course of treatment and for 24 months after the last dose because of the potential to cause serious development defects in breastfed infants and children.
- Hypersensitivity to the active substance or to any of the excipients
- Coadministration of St John's wort (Hypericum perforatum)

6.3 Warnings and Precautions

Severe cutaneous adverse reactions

Severe cutaneous adverse reactions (SCARs) including cases of Stevens-Johnson syndrome/Toxic epidermal necrolysis (SJS/TEN), Drug reaction with eosinophilia and systemic symptoms (DRESS) and acute generalised exanthematous pustulosis (AGEP), which can be life-threatening, have been reported during post-marketing use. If the patient has developed any of these reactions with the use of vismodegib, treatment with vismodegib must not be restarted in this patient at any time.

Cutaneous squamous cell carcinoma (cuSCC)

Patients with advanced BCC have an increased risk of developing cuSCC. Cases of cuSCC have been reported in advanced BCC patients treated with Erivedge. It has not been determined whether cuSCC is related to Erivedge treatment. Therefore, all patients should be monitored routinely while taking Erivedge, and cuSCC should be treated according to the standard of care.

Embryo-fetal death or severe birth defects

Vismodegib may cause embryo-fetal death or severe birth defects when administered to a pregnant woman. Hh pathway inhibitors such as vismodegib have been demonstrated to be embryotoxic and/or teratogenic in multiple animal species and can cause severe midline defects, missing digits, and other irreversible malformations in the developing embryo or fetus. Vismodegib must not be used during pregnancy, except in severe life-threatening cases, where the potential benefit to the patient outweighs the risk to the fetus.

Blood Donation

Patients should not donate blood or blood products while on study and for 24 months after the last dose of vismodegib.

Fertility

Vismodegib may impair fertility. Amenorrhea has been observed in clinical trials in
women of childbearing potential. Based on animal studies, reversibility of fertility impairment is unknown. Fertility preservation strategies should be discussed with
women of childbearing potential prior to starting treatment with vismodegib.

Excipients

Vismodegib capsules contain **lactose monohydrate**. Patients with the rare hereditary problems of galactose intolerance, primary hypolactasia, or glucose-galactose malabsorption should not take this medicine. This medicinal product contains less than 1 mmol sodium (23 mg) per dose, i.e. essentially ‘sodium free’.

Table 1 ADRs occurring in patients treated with Erivedge

MedDRA SOC	Very common	Common	Frequency not known
**Metabolism and nutrition disorders**	Decreased appetite	Dehydration	
**Nervous system disorder**	Dysgeusia<br>Ageusia	Hypogeusia	
**Gastrointestinal disorders**	Nausea<br>Diarrhoea<br>Constipation<br>Vomiting<br>Dyspepsia	Abdominal pain upper<br>Abdominal pain	
**Hepatobiliary disorders**		Hepatic enzymes increased**	Drug induced liver injury*****
**Skin and subcutaneous tissue disorders**	Alopecia<br>Pruritus<br>Rash	Madarosis<br>Abnormal hair growth	Stevens-Johnson Syndrome (SJS)/Toxic Epidermal Necrolysis (TEN), Drug Reaction with Eosinophilia and Systemic Symptoms (DRESS) and Acute Generalised Exanthematous Pustulosis (AGEP)******
**Musculoskeletal and connective tissue disorders**	Muscle spasms<br>Arthralgia<br>Pain in extremity	Back pain<br>Musculoskeletal chest pain<br>Myalgia<br>Flank pain<br>Musculoskeletal pain<br>Blood creatine phosphokinase increased***	Epiphyses premature fusion****
**Endocrine disorders**			Precocious puberty****
**Reproductive system and breast disorders**	Amenorrhoea*		
**General disorders and administration site conditions**	Weight decreased<br>Fatigue<br>Pain	Asthenia	

All reporting is based on ADRs of all grades using National Cancer Institute - Common Terminology Criteria for Adverse Events v 3.0 except where noted.
*Of the 138 patients with advanced BCC, 10 were WCBP. Amongst these women, amenorrhoea was observed in 3 patients (30 %).
MedDRA = Medical Dictionary for Regulatory Activities.
**Includes preferred terms: liver function test abnormal, blood bilirubin increased, gamma-glutamyl transferase increased, aspartate aminotransferase increased, alkaline phosphatase increased, liver hepatic enzyme increased.
*** Observed in patients during a post-approval study with 1215 safety evaluable patients.
****Individual cases have been reported in patients with medulloblastoma during post-marketing use
***** Cases of drug induced liver injury have been reported in patients during post-marketing use.

******Cases of SCAR (including SJS/TEN, DRESS and AGEP) have been reported in patients during post-marketing use.

6.4 Special Populations

Geriatric Patients
There are limited data in geriatric patients. Of the total number of patients in clinical studies of vismodegib with aBCC, approximately 40% of patients were ≥ 65 years of age and no overall differences in safety and efficacy were observed between these patients and younger patients. The safety profile in these patients was consistent with that of younger patients. Population PK analysis suggested that age did not have a clinically significant impact on steady-state plasma concentration of vismodegib.
Hepatic Impairment
The PK, safety, and tolerability of vismodegib were evaluated in patients with mild, moderate, or severe hepatic impairment in a dedicated clinical study (Study GP27839), following multiple doses of vismodegib. Results demonstrated no impact of hepatic impairment on the PK of vismodegib. No dose adjustment is required in patients with mild, moderate, or severe hepatic impairment.
Renal Impairment
No dedicated clinical study has been conducted to evaluate the effect of renal impairment on the PK of vismodegib. Results of a population PK analysis demonstrated no impact of renal impairment on the PK of vismodegib. No dose adjustment is required in patients with renal impairment (Population Pharmacokinetic Report. Population Pharmacokinetic Analysis of Vismodegib in Cancer Patients and Healthy Volunteers of Studies SHH3925g, SHH4433g, SHH4683g, SHH4610g, and SHH4476g. Report No 11- 2188. August 2011).
Pregnancy
Refer to appendix 1.

6.5 Concomitant Use with other medications

Effect of other drugs on Vismodegib
Clinically significant PK interactions between vismodegib and CYP450 inhibitors are not expected. Results from Study GP28465 demonstrated no clinically significant PK interaction between vismodegib and fluconazole (a moderate CYP2C9 inhibitor) or itraconazole (a strong CYP3A4 inhibitor) in healthy volunteers. Inducers of CYP3A4 are not predicted to alter vismodegib systemic exposure since similar steady-state plasma vismodegib concentrations were observed in patients in clinical studies concomitantly treated with CYP3A4 inducers (i.e., carbamazepine, modafinil, phenobarbital) and those concomitantly treated with CYP3A4 inhibitors (i.e., erythromycin, fluconazole).
Drugs that Inhibit Drug Transport Systems
Clinically significant PK interactions between vismodegib and P-gp inhibitors are not expected. Results from Study GP28465 demonstrated no clinically significant PK interaction between vismodegib and itraconazole (a strong P-glycoprotein inhibitor) in healthy volunteers.
Drugs that Affect Gastric pH
Clinically significant PK interactions between vismodegib and pH elevating agents are not expected. Results from Study GP28465 demonstrated no clinically significant PK interaction between vismodegib and rabeprazole (a proton pump inhibitor) in healthy volunteers.
Effects of Vismodegib on Other Drugs
Clinically significant PK interactions between vismodegib and CYP450 substrates are not expected. Results from the drug-drug interaction Study SHH4593g conducted in patients with cancer demonstrated no clinically significant PK interaction between vismodegib and rosiglitazone (a CYP2C8 substrate). Thus, inhibition of CYP enzymes by vismodegib may be excluded.
Results of the drug-drug interaction Study SHH4593g conducted in cancer patients demonstrated no clinically significant PK interaction between vismodegib and the oral contraceptives ethinyl estradiol and norethindrone.

Clinically significant PK interactions between vismodegib and BCRP substrates are not expected. In vitro data indicate that vismodegib is an inhibitor of the BCRP transporter; however, the in vitro concentrations at which inhibition occurred are significantly greater than the unbound vismodegib concentrations observed in patients.

6.6 Overdose

Vismodegib has been administered at doses 3.6 x higher than the recommended
150-mg daily dose. No increases in plasma drug levels or toxicity were observed during these clinical studies.

Missed Doses

If a dose of vismodegib is missed, patients should be instructed not to take the missed capsule, but to resume with the next scheduled dose.

6.7 First Aid Measure

- Eye contact: Rinse immediately with tap water for 10 minutes; open eyelids forcibly
- Skin contact: Remove immediately contaminated clothes; wash affected skin with water and soap. Do not use any solvents
- Inhalation: Take fresh air and keep calm. In the event of symptoms, seek medical treatment

Note to physician: Treat symptomatically after accidental exposure. Women of childbearing potential who are not taking vismodegib actively or pregnant women
should get medical advice from a physician.

6.8 Accidental capsule Breakage

In case of accidental capsule breakage, these instructions should be followed by sites and/or patients:

- Keep your face away from the open container
- Close the container and return the medication to the pharmacists for proper handling
- In case of eye or skin contact or accidental inhalation followed first aid measures
- If the contents of the capsule have spilled outside the container, wear protective gloves and protective mask, remove the spilled material with a wet towel, wash the area with soap and water, and dispose any material that had contact with the substance in a closed container
- Observe local/national regulations regarding waste disposal

Do not allow drug to enter drains or waterways.

7 COTELLIC (COBIMETINIB)

Investigators should also consult the local prescribing information if the IMP is approved in your country.

7.1 Standard dose:

The recommended dose of Cotellic is 60 mg (3 x 20 mg tablets) once a day. Cotellic is taken on a 28 day cycle. Each dose consists of three 20 mg tablets (60 mg) and should be taken once daily for 21 consecutive days (Days 1 to 21-treatment period); followed by a 7-day break (Days 22 to 28-treatment break). Each subsequent Cotellic treatment cycle should start after the 7-day treatment break has elapsed. Treatment with Cotellic should continue until the patient no longer derives benefit or until the development of unacceptable toxicity. If a dose is missed, it can be taken up to 12 hours prior to the next dose to maintain the once-daily regimen. In case of vomiting after administration of Cotellic, the patient should not take an additional dose on that day and treatment should be continued as prescribed the following day.

7.2 Contraindications

Cobimetinib is contraindicated in patients with known hypersensitivity to cobimetinib or any of the excipients.

7.3 Warnings and Precautions

Hemorrhage

Hemorrhage, including major hemorrhages, defined as symptomatic bleeding in a critical area or organ, can occur with cobimetinib. In clinical studies with cobimetinib, events of cerebral hemorrhage, GI tract hemorrhage, reproductive tract hemorrhage, and hematuria have been reported.

In the Phase III Study GO28141, Grade 1-4 hemorrhagic events were reported in 13.0 % of patients treated with cobimetinib + vemurafenib and in 7.3% of patients treated with placebo + vemurafenib. The majority of hemorrhagic events were Grade 1 or 2 and non-serious. Grade 3-4 hemorrhage events were reported in 1.2% of patients receiving cobimetinib + vemurafenib and 0.8% of patients receiving placebo + vemurafenib. Caution should be used in patients with additional risk factors for bleeding, such as brain metastases, and in patients that use concomitant medications that increase the risk of bleeding (including antiplatelet or anticoagulant therapy).

Serous Retinopathy

Serous retinopathy (fluid accumulation within the layers of the retina) has been observed with MEK inhibitors, including cobimetinib. The majority of events were reported as chorioretinopathy or retinal detachment.

Median time to initial onset of serous retinopathy events was 1 month (range: 0-9 months). Most events observed in clinical trials were resolved or improved to
asymptomatic grade 1 following dose interruption or reduction.

For patients reporting new or worsening visual disturbances, an ophthalmologic
examination is strongly recommended. If serous retinopathy is diagnosed, cobimetinib treatment should be withheld until visual symptoms improve to Grade ≤ 1. Serious retinopathy can be managed with treatment interruption, dose reduction or with treatment discontinuation.

Patients should be advised to use caution when driving or using machines if their vision is impaired.

Left Ventricular Dysfunction

Decrease in left ventricular ejection fraction (LVEF) from baseline has been reported in patients receiving cobimetinib (see Table 48 for summarized safety data). Mean time to initial onset of events was 4 months (range: 1-13 months).

LVEF should be evaluated before initiation of treatment to establish baseline values, then (at a minimum) after the first month of treatment and at least every 3 months, or as clinically indicated until treatment discontinuation. Decrease in LVEF from baseline can be managed using treatment interruption, dose reduction or with treatment discontinuation.

All patients restarting treatment with a dose reduction of cobimetinib should have LVEF measurements taken at approximately 2 weeks, 4 weeks, 10 weeks, and 16 weeks, and then as clinically indicated until treatment discontinuation.
Permanent discontinuation of cobimetinib treatment should be considered if cardiac symptoms are attributed to cobimetinib and do not improve after temporary interruption of cobimetinib.
Patients with a baseline LVEF either below institutional lower limit of normal or below 50% have not been studied.

Table 1: Recommended dose modifications for Cotellic in patients with left ventricular ejection fraction (LVEF) decrease from baseline

**Patient**	**LVEF value**	**Recommended Cotellic dose modification**	**LVEF value following treatment break**	**Recommended Cotellic daily dose**
Asymptomatic	≥ 50% (or 40-49% and < 10% absolute decrease from baseline)	Continue at current dose	N/A	N/A
	< 40% (or 40-49% and ≥ 10% absolute decrease from baseline)	Interrupt treatment for 2 weeks	< 10% absolute decrease from baseline	1st occurrence: 40 mg
				2nd occurrence: 20 mg
				3rd occurrence: permanent discontinuation
			< 40% (or ≥ 10% absolute decrease from baseline)	Permanent discontinuation
Symptomatic	N/A	Interrupt treatment for 4 weeks	Asymptomatic and < 10% absolute decrease from baseline	1st occurrence: 40 mg
				2nd occurrence: 20 mg
				3rd occurrence: permanent discontinuation
			Asymptomatic and < 40% (or ≥ 10% absolute decrease from	Permanent discontinuation

			baseline)	
			Symptomatic regardless of LVEF	Permanent discontinuation

N/A = Not Applicable

Rhabdomyolysis and CPK Elevations

CPK elevations have been observed in patients who received cobimetinib monotherapy, as well as in patients administered cobimetinib in combination with other agents. The majority of CPK elevations reported were asymptomatic, non-serious, and resolved with or without study drug interruption. One event of rhabdomyolysis was reported in the Phase III Study GO28141 (cobimetinib + vemurafenib), and rhabdomyolysis has been reported in postmarketing experience.

In Study GO28141, CPK elevations was reported as an AE more frequently in patients treated with cobimetinib + vemurafenib (32.4% all grades, 11.3% Grade ≥ 3 events) than with placebo + vemurafenib (8.1% all grades, 0% Grade ≥ 3 events).

Monitor CPK at baseline and monthly during treatment or as clinically indicated.

Liver Laboratory Abnormalities

Liver laboratory abnormalities can occur when cobimetinib is used with vemurafenib, and when vemurafenib is used as a single agent.

Liver laboratory abnormalities, specifically increases in ALT, AST, and ALP, have been observed in patients treated with cobimetinib + vemurafenib.

Monitor liver laboratory tests before initiation of treatment and monthly during treatment, or more frequently as clinically indicated.

Manage Grade 3 and 4 liver laboratory abnormalities with dose interruption, reduction, or discontinuation of both cobimetinib and vemurafenib.

Table 2: Recommended Cotellic dose modifications

Grade (CTC-AE)*	Recommended Cotellic dose
**Grade 1 or Grade 2 (tolerable)**	No dose reduction. Maintain Cotellic at a dose of 60 mg once daily (3 tablets)
**Grade 2 (intolerable) or Grade 3/4**	
1st Appearance	Interrupt treatment until Grade ≤ 1, restart treatment at 40 mg once daily (2 tablets)
2nd Appearance	Interrupt treatment until Grade ≤ 1, restart treatment at 20 mg once daily (1 tablet)
3rd Appearance	Consider permanent discontinuation

7.4 Special Populations

Pregnancy

Refer to appendix 1. There are no data regarding the use of cobimetinib in pregnant women.

Nursing Mothers

It is not known whether cobimetinib is excreted in human milk. Because many drugs are excreted in human milk and because of the potential for serious ADRs in nursing infants, cobimetinib should not be administered to nursing mothers.

Geriatric Patients
Age does not have an effect on cobimetinib exposure.
Other
Hepatic Impairment
No dose adjustment is recommended in patients with hepatic impairment. Liver laboratory abnormalities can occur when cobimetinib is used in combination with vemurafenib.
Renal Impairment
No dose adjustment is recommended in patients with mild or moderate renal impairment, based on popPK analysis. The safety and efficacy of cobimetinib has not been established in patients with severe renal impairment.

7.5 Concomitant use with other medications

Effects of Concomitant Medications on Cobimetinib
CYP3A Inhibitors/Inducers
Cobimetinib is metabolized by CYP3A and exposures increased approximately 7-fold in the presence of a potent CYP3A inhibitor (itraconazole) in healthy subjects. Because cobimetinib is a sensitive substrate of CYP3A, it is likely that cobimetinib exposures will be significantly lower in the presence of CYP3A inducers. Therefore concomitant administration of potent CYP3A inducers and inhibitors is not recommended. Caution should be exercised when cobimetinib is co-administered with moderate CYP3A inducers and inhibitors.
Acid Reducing Agents
Cobimetinib PK is not altered by the co-administration of a proton pump inhibitor. Thus, gastric pH elevations do not affect cobimetinib absorption.
Effects of Cobimetinib on Concomitant Medications
CYP Substrates
Coadministration of cobimetinib 60 mg once daily for 15 days with a single 30 mg dose of dextromethorphan (sensitive CYP2D6 substrate) or a 2 mg dose of midazolam (sensitive CYP3A substrate) to 20 patients with solid tumors did not change dextromethorphan or midazolam systemic exposure. In vitro data indicate that cobimetinib may inhibit CYP3A and CYP2D6. Cobimetinib is not an inhibitor of CYP1A2, 2B6, 2C8, 2C9 and 2C19 or inducer of CYP1A2, 2B6 and 3A4 at clinically relevant concentrations.
Other Anti-Cancer Agents
Zelboraf
There is no evidence of any clinically significant DDI between cobimetinib and vemurafenib in unresectable or metastatic melanoma patients.
Effects of Cobimetinib on Drug Transport Systems
Effect of Transporters on Cobimetinib: Cobimetinib is a substrate of efflux transporter of P-gp, but is not a substrate of Breast Cancer Resistance Protein (BCRP), Organic Anion Transporting Polypeptide (OATP1B1 or OATP1B3) or Organic Cation Transporter (OCT1) in vitro. Drugs that inhibit P-gp may increase cobimetinib concentrations.
Effect of Cobimetinib on Transporters: In vitro data suggest that cobimetinib does
not inhibit P-gp, BCRP, OATP1B1, OATP1B3, or OCT1 at clinical concentrations.

7.6 Overdose

There is no clinical experience with an overdose of cobimetinib. No experiments have been performed to determine whether the effects of an overdose can be reversed, and there are no known antidotes.
However, there is a risk of overexposure from reconstitution of cobimetinib powder for oral suspension. If more than 10 reconstitutions of cobimetinib powder for oral
suspension are made per day, wearing respiratory protection during the reconstitution or using an exhaust hood (e.g. ventilation booth) is recommended.
In the event of an overdose, no further cobimetinib should be dispensed; the individual should be monitored clinically, supportive measures should be undertaken as clinically indicated, and ECG and clinical

laboratory evaluations, including blood glucose, hepatic enzymes, creatinine, BUN, CPK, and complete blood counts, should be performed and followed until they return to baseline.

8 TECENTRIQ (ATEZOLIZUMAB)

Investigators should also consult the local prescribing information if the IMP is approved in your country.

8.1 Standard dose:

The recommended dose of Tecentriq corresponds to:
• 840 mg administered intravenously every two weeks or
• 1200 mg administered intravenously every three weeks or
• 1680 mg administered intravenously every four weeks.

8.2 Contraindications

Atezolizumab is contraindicated for patients with the following:

- History of severe allergic anaphylactic reactions to chimeric, human or humanized antibodies, or fusion proteins
- Known hypersensitivity to CHO cell products or any component of the atezolizumab formulation.

8.3 Warnings and precautions

Toxicities associated or possibly associated with atezolizumab treatment should be managed according to standard medical practice. Additional tests, such as autoimmune serology or biopsies, should be used to evaluate for a possible immunogenic etiology.
Although most immune-mediated adverse events observed with immunomodulatory agents have been mild and self-limiting, such events should be recognized early and treated promptly to avoid potential major complications. Discontinuation of atezolizumab may not have an immediate therapeutic effect, and in severe cases, immune-mediated toxicities may require acute management with topical corticosteroids, systemic corticosteroids, or other immunosuppressive agents.
The investigator should consider the benefit–risk balance a given patient may be experiencing prior to further administration of atezolizumab. In patients who have met the criteria for permanent discontinuation, resumption of atezolizumab may be considered if the patient is deriving benefit and has fully recovered from the immune-mediated event. Patients can be re-challenged with atezolizumab only after approval has been documented by both the investigator (or an appropriate delegate) and the Medical Monitor.

<u>Pulmonary Events</u>

Dyspnea, cough, fatigue, hypoxia, pneumonitis, and pulmonary infiltrates have been associated with the administration of atezolizumab. Patients will be assessed for pulmonary signs and symptoms throughout the study.
All pulmonary events should be thoroughly evaluated for other commonly reported etiologies such as pneumonia or other infection, lymphangitic carcinomatosis, pulmonary embolism, heart failure, chronic obstructive pulmonary disease, or pulmonary hypertension. Management guidelines for pulmonary events are provided in table 1.

Table 1. Management Guidelines for Pulmonary Events, Including Pneumonitis

Event	Management
Pulmonary event, Grade 1	• Continue atezolizumab and monitor closely.<br>• Re-evaluate on serial imaging.<br>• Consider patient referral to pulmonary specialist.
Pulmonary event, Grade 2	• Withhold atezolizumab for up to 12 weeks after event onset. [a]<br>• Refer patient to pulmonary and infectious disease specialists and consider bronchoscopy or BAL.<br>• Initiate treatment with corticosteroids equivalent to 1–2 mg/kg/day oral prednisone.<br>• If event resolves to Grade 1 or better, resume atezolizumab. [b]<br>• If event does not resolve to Grade 1 or better while withholding atezolizumab, permanently discontinue atezolizumab and contact Medical Monitor. [c]<br>• For recurrent events, treat as a Grade 3 or 4 event.
Pulmonary event, Grade 3 or 4	• Permanently discontinue atezolizumab and contact Medical Monitor. [c]<br>• Bronchoscopy or BAL is recommended.<br>• Initiate treatment with corticosteroids equivalent to 1–2 mg/kg/day oral prednisone.<br>• If event does not improve within 48 hours after initiating corticosteroids, consider adding an immunosuppressive agent.<br>• If event resolves to Grade 1 or better, taper corticosteroids over $\geq$ 1 month.

BAL ⍰ bronchoscopic alveolar lavage.

[a] Atezolizumab may be withheld for a longer period of time (i.e., $>$ 12 weeks after event onset) to allow for corticosteroids (if initiated) to be reduced to the equivalent of $\leq$ 10 mg/day oral prednisone. The acceptable length of the extended period of time must be agreed upon by the investigator and the Medical Monitor.

[b] If corticosteroids have been initiated, they must be tapered over $\geq$ 1 month to the equivalent of $\leq$ 10 mg/day oral prednisone before atezolizumab can be resumed.

[c] Resumption of atezolizumab may be considered in patients who are deriving benefit and have fully recovered from the immune-mediated event. Patients can be re-challenged with atezolizumab only after approval has been documented by both the investigator (or an appropriate delegate) and the Medical Monitor.

Hepatic Events

Immune-mediated hepatitis has been associated with the administration of atezolizumab. Eligible patients must have adequate liver function, as manifested by measurements of total bilirubin and hepatic transaminases, and liver function will be monitored throughout study treatment. Management guidelines for hepatic events are provided in Table 2.

Patients with right upper-quadrant abdominal pain and/or unexplained nausea or vomiting should have liver function tests (LFTs) performed immediately and reviewed before administration of the next dose of study drug.

For patients with elevated LFTs, concurrent medication, viral hepatitis, and toxic or neoplastic etiologies should be considered and addressed, as appropriate.

Table 2. Management Guidelines for Hepatic Events

Event	Management
Hepatic event, Grade 1	• Continue atezolizumab.<br>• Monitor LFTs until values resolve to within normal limits or to baseline values.
Hepatic event, Grade 2	**All events:**<br>• Monitor LFTs more frequently until return to baseline values.<br>**Events of > 5 days' duration:**<br>• Withhold atezolizumab for up to 12 weeks after event onset. [a]<br>• Initiate treatment with corticosteroids equivalent to 1–2 mg/kg/day oral prednisone.<br>• If event resolves to Grade 1 or better, resume atezolizumab. [b]<br>• If event does not resolve to Grade 1 or better while withholding atezolizumab, permanently discontinue atezolizumab and contact Medical Monitor. [c]

LFT ⍰ liver function test.

[a] Atezolizumab may be withheld for a longer period of time (i.e., > 12 weeks after event onset) to allow for corticosteroids (if initiated) to be reduced to the equivalent of ≤ 10 mg/day oral prednisone. The acceptable length of the extended period of time must be agreed upon by the investigator and the Medical Monitor.

[b] If corticosteroids have been initiated, they must be tapered over ≥ 1 month to the equivalent of ≤ 10 mg/day oral prednisone before atezolizumab can be resumed.

[c] Resumption of atezolizumab may be considered in patients who are deriving benefit and have fully recovered from the immune-mediated event. Patients can be re-challenged with atezolizumab only after approval has been documented by both the investigator (or an appropriate delegate) and the Medical Monitor.

Table 2. Management Guidelines for Hepatic Events (cont.)

Event	Management
Hepatic event, Grade 3 or 4	• Permanently discontinue atezolizumab and contact Medical Monitor. [c]<br>• Consider patient referral to gastrointestinal specialist for evaluation and liver biopsy to establish etiology of hepatic injury.<br>• Initiate treatment with corticosteroids equivalent to 1–2 mg/kg/day oral prednisone.<br>• If event does not improve within 48 hours after initiating corticosteroids, consider adding an immunosuppressive agent.<br>• If event resolves to Grade 1 or better, taper corticosteroids over ≥ 1 month.

LFT liver function test.

[a] Atezolizumab may be withheld for a longer period of time (i.e., > 12 weeks after event onset) to allow for corticosteroids (if initiated) to be reduced to the equivalent of ≤ 10 mg/day oral prednisone. The acceptable length of the extended period of time must be agreed upon by the investigator and the Medical Monitor.

[b] If corticosteroids have been initiated, they must be tapered over ≥ 1 month to the equivalent of ≤ 10 mg/day oral prednisone before atezolizumab can be resumed.

[c] Resumption of atezolizumab may be considered in patients who are deriving benefit and have fully recovered from the immune-mediated event. Patients can be re-challenged with atezolizumab only after approval has been documented by both the investigator (or an appropriate delegate) and the Medical Monitor.

Gastrointestinal Events

Immune-mediated colitis has been associated with the administration of atezolizumab. Management guidelines for diarrhea or colitis are provided in Table 3.

All events of diarrhea or colitis should be thoroughly evaluated for other more common etiologies. For events of significant duration or magnitude or associated with signs of systemic inflammation or acute-phase reactants (e.g., increased C-reactive protein, platelet count, or bandemia): Perform sigmoidoscopy (or colonoscopy, if appropriate) with colonic biopsy, with three to five specimens for standard paraffin block to check for inflammation and lymphocytic infiltrates to confirm colitis diagnosis.

Table 3. Management Guidelines for Gastrointestinal Events (Diarrhea or Colitis)

Event	Management
Diarrhea or colitis, Grade 1	• Continue atezolizumab.<br>• Initiate symptomatic treatment.<br>• Endoscopy is recommended if symptoms persist for $>$ 7 days.<br>• Monitor closely.
Diarrhea or colitis, Grade 2	• Withhold atezolizumab for up to 12 weeks after event onset. [a]<br>• Initiate symptomatic treatment.<br>• Patient referral to GI specialist is recommended.<br>• For recurrent events or events that persist □ 5 days, initiate treatment with corticosteroids equivalent to 1–2 mg/kg/day oral prednisone.<br>• If event resolves to Grade 1 or better, resume atezolizumab. [b]<br>• If event does not resolve to Grade 1 or better while withholding atezolizumab, permanently discontinue atezolizumab and contact Medical Monitor. [c]
Diarrhea or colitis, Grade 3	• Withhold atezolizumab for up to 12 weeks after event onset. [a]<br>• Refer patient to GI specialist for evaluation and confirmatory biopsy.<br>• Initiate treatment with corticosteroids equivalent to 1–2 mg/kg/day IV methylprednisolone and convert to 1–2 mg/kg/day oral prednisone or equivalent upon improvement.<br>• If event resolves to Grade 1 or better, resume atezolizumab. [b]<br>• If event does not resolve to Grade 1 or better while withholding atezolizumab, permanently discontinue atezolizumab and contact Medical Monitor. [c]

GI = gastrointestinal.

[a] Atezolizumab may be withheld for a longer period of time (i.e., $>$ 12 weeks after event onset) to allow for corticosteroids (if initiated) to be reduced to the equivalent of $\leq$ 10 mg/day oral prednisone. The acceptable length of the extended period of time must be agreed upon by the investigator and the Medical Monitor.

[b] If corticosteroids have been initiated, they must be tapered over $\geq$ 1 month to the equivalent of $\leq$ 10 mg/day oral prednisone before atezolizumab can be resumed.

[c] Resumption of atezolizumab may be considered in patients who are deriving benefit and have fully recovered from the immune-mediated event. Patients can be re-challenged with atezolizumab only after approval has been documented by both the investigator (or an appropriate delegate) and the Medical Monitor.

Table 3. Management Guidelines for Gastrointestinal Events (Diarrhea or Colitis) (cont.)

Event	Management
Diarrhea or colitis, Grade 4	• Permanently discontinue atezolizumab and contact Medical Monitor. [c]<br>• Refer patient to GI specialist for evaluation and confirmation biopsy.<br>• Initiate treatment with corticosteroids equivalent to 1–2 mg/kg/day IV methylprednisolone and convert to 1–2 mg/kg/day oral prednisone or equivalent upon improvement.<br>• If event does not improve within 48 hours after initiating corticosteroids, consider adding an immunosuppressive agent.<br>• If event resolves to Grade 1 or better, taper corticosteroids over ≥ 1 month.

GI = gastrointestinal.

[a] Atezolizumab may be withheld for a longer period of time (i.e., > 12 weeks after event onset) to allow for corticosteroids (if initiated) to be reduced to the equivalent of ≤ 10 mg/day oral prednisone. The acceptable length of the extended period of time must be agreed upon by the investigator and the Medical Monitor.

[b] If corticosteroids have been initiated, they must be tapered over ≥ 1 month to the equivalent of ≤ 10 mg/day oral prednisone before atezolizumab can be resumed.

[c] Resumption of atezolizumab may be considered in patients who are deriving benefit and have fully recovered from the immune-mediated event. Patients can be re-challenged with atezolizumab only after approval has been documented by both the investigator (or an appropriate delegate) and the Medical Monitor.

Endocrine Events

Thyroid disorders, adrenal insufficiency, diabetes mellitus, and pituitary disorders have been associated with the administration of atezolizumab. Management guidelines for endocrine events are provided in Table 4.

Patients with unexplained symptoms such as headache, fatigue, myalgias, impotence, constipation, or mental status changes should be investigated for the presence of thyroid, pituitary, or adrenal endocrinopathies. The patient should be referred to an endocrinologist if an endocrinopathy is suspected. Thyroid-stimulating hormone (TSH) and free triiodothyronine and thyroxine levels should be measured to determine whether thyroid abnormalities are present. Pituitary hormone levels and function tests (e.g., TSH, growth hormone, luteinizing hormone, follicle-stimulating hormone, testosterone, prolactin, adrenocorticotropic hormone [ACTH] levels, and ACTH stimulation test) and magnetic resonance imaging (MRI) of the brain (with detailed pituitary sections) may help to differentiate primary pituitary insufficiency from primary adrenal insufficiency.

Table 4. Management Guidelines for Endocrine Events

Event	Management
Asymptomatic hypothyroidism	• Continue atezolizumab.<br>• Initiate treatment with thyroid replacement hormone.<br>• Monitor TSH weekly.
Symptomatic hypothyroidism	• Withhold atezolizumab.<br>• Initiate treatment with thyroid replacement hormone.<br>• Monitor TSH weekly.<br>• Consider patient referral to endocrinologist.<br>• Resume atezolizumab when symptoms are controlled and thyroid function is improving.
Asymptomatic hyperthyroidism	**TSH $\geq$ 0.1 mU/L and $<$ 0.5 mU/L:**<br>• Continue atezolizumab.<br>• Monitor TSH every 4 weeks.<br>**TSH $<$ 0.1 mU/L:**<br>• Follow guidelines for symptomatic hyperthyroidism.
Symptomatic hyperthyroidism	• Withhold atezolizumab.<br>• Initiate treatment with anti-thyroid drug such as methimazole or carbimazole as needed.<br>• Consider patient referral to endocrinologist.<br>• Resume atezolizumab when symptoms are controlled and thyroid function is improving.<br>• Permanently discontinue atezolizumab and contact Medical Monitor for life-threatening immune-mediated hyperthyroidism. [c]

MRI = magnetic resonance imaging; TSH = thyroid-stimulating hormone.

[a] Atezolizumab may be withheld for a longer period of time (i.e., > 12 weeks after event onset) to allow for corticosteroids (if initiated) to be reduced to the equivalent of ≤ 10 mg/day oral prednisone. The acceptable length of the extended period of time must be agreed upon by the investigator and the Medical Monitor.

[b] If corticosteroids have been initiated, they must be tapered over ≥ 1 month to the equivalent of ≤ 10 mg/day oral prednisone before atezolizumab can be resumed.

[c] Resumption of atezolizumab may be considered in patients who are deriving benefit and have fully recovered from the immune-mediated event. Patients can be re-challenged with atezolizumab only after approval has been documented by both the investigator (or an appropriate delegate) and the Medical Monitor.

Table 4. Management Guidelines for Endocrine Events (cont.)

Event	Management
Symptomatic adrenal insufficiency, Grade 2–4	• Withhold atezolizumab for up to 12 weeks after event onset. [a]<br>• Refer patient to endocrinologist.<br>• Perform appropriate imaging.<br>• Initiate treatment with corticosteroids equivalent to 1–2 mg/kg/day IV methylprednisolone and convert to 1–2 mg/kg/day oral prednisone or equivalent upon improvement.<br>• If event resolves to Grade 1 or better and patient is stable on replacement therapy, resume atezolizumab. [b]<br>• If event does not resolve to Grade 1 or better or patient is not stable on replacement therapy while withholding atezolizumab, permanently discontinue atezolizumab and contact Medical Monitor. [c]
Hyperglycemia, Grade 1 or 2	• Continue atezolizumab.<br>• Investigate for diabetes. If patient has Type 1 diabetes, treat as a Grade 3 event. If patient does not have Type 1 diabetes, treat as per institutional guidelines.<br>• Monitor for glucose control.
Hyperglycemia, Grade 3 or 4	• Withhold atezolizumab.<br>• Initiate treatment with insulin.<br>• Monitor for glucose control.<br>• Resume atezolizumab when symptoms resolve and glucose levels are stable.

MRI = magnetic resonance imaging; TSH = thyroid-stimulating hormone.

[a] Atezolizumab may be withheld for a longer period of time (i.e., > 12 weeks after event onset) to allow for corticosteroids (if initiated) to be reduced to the equivalent of ≤ 10 mg/day oral prednisone. The acceptable length of the extended period of time must be agreed upon by the investigator and the Medical Monitor.

[b] If corticosteroids have been initiated, they must be tapered over ≥ 1 month to the equivalent of ≤ 10 mg/day oral prednisone before atezolizumab can be resumed.

[c] Resumption of atezolizumab may be considered in patients who are deriving benefit and have fully recovered from the immune-mediated event. Patients can be re-challenged with atezolizumab only after approval has been documented by both the investigator (or an appropriate delegate) and the Medical Monitor.

Table 4. Management Guidelines for Endocrine Events (cont.)

Event	Management
Hypophysitis (pan-hypopituitarism), Grade 2 or 3	• Withhold atezolizumab for up to 12 weeks after event onset. [a]<br>• Refer patient to endocrinologist.<br>• Perform brain MRI (pituitary protocol).<br>• Initiate treatment with corticosteroids equivalent to 1–2 mg/kg/day IV methylprednisolone and convert to 1–2 mg/kg/day oral prednisone or equivalent upon improvement.<br>• Initiate hormone replacement if clinically indicated.<br>• If event resolves to Grade 1 or better, resume atezolizumab. [b]<br>• If event does not resolve to Grade 1 or better while withholding atezolizumab, permanently discontinue atezolizumab and contact Medical Monitor. [c]<br>• For recurrent hypophysitis, treat as a Grade 4 event.
Hypophysitis (pan-hypopituitarism), Grade 4	• Permanently discontinue atezolizumab and contact Medical Monitor. [c]<br>• Refer patient to endocrinologist.<br>• Perform brain MRI (pituitary protocol).<br>• Initiate treatment with corticosteroids equivalent to 1–2 mg/kg/day IV methylprednisolone and convert to 1–2 mg/kg/day oral prednisone or equivalent upon improvement.<br>• Initiate hormone replacement if clinically indicated.

MRI = magnetic resonance imaging; TSH = thyroid-stimulating hormone.

[a] Atezolizumab may be withheld for a longer period of time (i.e., > 12 weeks after event onset) to allow for corticosteroids (if initiated) to be reduced to the equivalent of ≤ 10 mg/day oral prednisone. The acceptable length of the extended period of time must be agreed upon by the investigator and the Medical Monitor.

[b] If corticosteroids have been initiated, they must be tapered over ≥ 1 month to the equivalent of ≤ 10 mg/day oral prednisone before atezolizumab can be resumed.

[c] Resumption of atezolizumab may be considered in patients who are deriving benefit and have fully recovered from the immune-mediated event. Patients can be re-challenged with atezolizumab only after approval has been documented by both the investigator (or an appropriate delegate) and the Medical Monitor.

Ocular Events

An ophthalmologist should evaluate visual complaints (e.g., uveitis, retinal events). Management guidelines for ocular events are provided in Table 5.

Table 5. Management Guidelines for Ocular Events

Event	Management
Ocular event, Grade 1	• Continue atezolizumab.<br>• Patient referral to ophthalmologist is strongly recommended.<br>• Initiate treatment with topical corticosteroid eye drops and topical immunosuppressive therapy.<br>• If symptoms persist, treat as a Grade 2 event.
Ocular event, Grade 2	• Withhold atezolizumab for up to 12 weeks after event onset. [a]<br>• Patient referral to ophthalmologist is strongly recommended.<br>• Initiate treatment with topical corticosteroid eye drops and topical immunosuppressive therapy.<br>• If event resolves to Grade 1 or better, resume atezolizumab. [b]<br>• If event does not resolve to Grade 1 or better while withholding atezolizumab, permanently discontinue atezolizumab and contact Medical Monitor. [c]
Ocular event, Grade 3 or 4	• Permanently discontinue atezolizumab and contact Medical Monitor. [c]<br>• Refer patient to ophthalmologist.<br>• Initiate treatment with corticosteroids equivalent to 1–2 mg/kg/day oral prednisone.<br>• If event resolves to Grade 1 or better, taper corticosteroids over ≥ 1 month.

[a] Atezolizumab may be withheld for a longer period of time (i.e., > 12 weeks after event onset) to allow for corticosteroids (if initiated) to be reduced to the equivalent of ≤ 10 mg/day oral prednisone. The acceptable length of the extended period of time must be agreed upon by the investigator and the Medical Monitor.

[b] If corticosteroids have been initiated, they must be tapered over ≥ 1 month to the equivalent of ≤ 10 mg/day oral prednisone before atezolizumab can be resumed.

[c] Resumption of atezolizumab may be considered in patients who are deriving benefit and have fully recovered from the immune-mediated event. Patients can be re-challenged with atezolizumab only after approval has been documented by both the investigator (or an appropriate delegate) and the Medical Monitor.

Immune-mediated myocarditis

Immune-mediated myocarditis has been associated with the administration of atezolizumab. Immune-mediated myocarditis should be suspected in any patient presenting with signs or symptoms suggestive of myocarditis, including, but not limited to, laboratory (e.g., B-type natriuretic peptide) or cardiac imaging abnormalities, dyspnea, chest pain, palpitations, fatigue, decreased exercise tolerance, or syncope. Immune-mediated myocarditis needs to be distinguished from myocarditis resulting from infection (commonly viral, e.g., in a patient who reports a recent history of gastrointestinal illness), ischemic events, underlying arrhythmias, exacerbation of preexisting cardiac conditions, or progression of malignancy.

All patients with possible myocarditis should be urgently evaluated by performing cardiac enzyme assessment, an ECG, a chest X-ray, an echocardiogram, and a cardiac MRI as appropriate per institutional guidelines. A cardiologist should be consulted. An endomyocardial biopsy may be considered to enable a definitive diagnosis and appropriate treatment, if clinically indicated.

Patients with signs and symptoms of myocarditis, in the absence of an identified alternate etiology, should be treated according to the guidelines in Table 6.

Table 6. Management Guidelines for Immune-Mediated Myocarditis

Event	Management
Immune-mediated myocarditis, Grade 2	• Withhold atezolizumab for up to 12 weeks after event onset [a] and contact Medical Monitor.<br>• Refer patient to cardiologist.<br>• Initiate treatment as per institutional guidelines and consider antiarrhythmic drugs, temporary pacemaker, ECMO, or VAD as appropriate.<br>• Consider treatment with corticosteroids equivalent to 1–2 mg/kg/day IV methylprednisolone and convert to 1–2 mg/kg/day oral prednisone or equivalent upon improvement.<br>• If event resolves to Grade 1 or better, resume atezolizumab. [b]<br>• If event does not resolve to Grade 1 or better while withholding atezolizumab, permanently discontinue atezolizumab and contact Medical Monitor. [c]
Immune-mediated myocarditis, Grade 3-4	• Permanently discontinue atezolizumab and contact Medical Monitor. [c]<br>• Refer patient to cardiologist.<br>• Initiate treatment as per institutional guidelines and consider antiarrhythmic drugs, temporary pacemaker, ECMO, or VAD as appropriate.<br>• Initiate treatment with corticosteroids equivalent to 1–2 mg/kg/day IV methylprednisolone and convert to 1–2 mg/kg/day oral prednisone or equivalent upon improvement.<br>• If event does not improve within 48 hours after initiating corticosteroids, consider adding an immunosuppressive agent.<br>• If event resolves to Grade 1 or better, taper corticosteroids over ≥ 1 month.

ECMO = extracorporeal membrane oxygenation; VAD = ventricular assist device.

[a] Atezolizumab may be withheld for a longer period of time (i.e., > 12 weeks after event onset) to allow for corticosteroids (if initiated) to be reduced to the equivalent of ≤ 10 mg/day oral prednisone. The acceptable length of the extended period of time must be agreed upon by the investigator and the Medical Monitor.

[b] If corticosteroids have been initiated, they must be tapered over ≥ 1 month to the equivalent of ≤ 10 mg/day oral prednisone before atezolizumab can be resumed.

[c] Resumption of atezolizumab may be considered in patients who are deriving benefit and have fully recovered from the immune-mediated event. Patients can be re-challenged with atezolizumab only after approval has been documented by both the investigator (or an appropriate delegate) and the Medical Monitor.

<u>Infusion-related reactions and cytokine-release syndrome</u>

No premedication is indicated for the administration of Cycle 1 of atezolizumab. However, patients who experience an infusion-related reaction (IRR) or cytokine-release syndrome (CRS) with atezolizumab may receive premedication with antihistamines, anti-pyretics, and/or analgesics (e.g., acetaminophen) for subsequent infusions. Metamizole (dipyrone) is prohibited in treating atezolizumab-associated IRRs because of its potential for causing agranulocytosis.

IRRs are known to occur with the administration of monoclonal antibodies and have been reported with atezolizumab. These reactions, which are thought to be due to release of cytokines and/or other chemical

mediators, occur within 24 hours of atezolizumab administration and are generally mild to moderate in severity.

CRS is defined as a supraphysiologic response following administration of any immune therapy that results in activation or engagement of endogenous or infused T cells and/or other immune effector cells. Symptoms can be progressive, always include fever at the onset, and may include hypotension, capillary leak (hypoxia), and end-organ dysfunction (Lee et al. 2019). CRS has been well documented with chimeric antigen receptor T-cell therapies and bispecific T-cell engager antibody therapies but has also been reported with immunotherapies that target PD-1 or PD-L1 (Rotz et al. 2017; Adashek and Feldman 2019), including atezolizumab.

There may be significant overlap in signs and symptoms of IRRs and CRS, and in recognition of the challenges in clinically distinguishing between the two, consolidated guidelines for medical management of IRRs and CRS are provided in Table 7.

Table 7. Management Guidelines for Infusion-Related Reactions and Cytokine-Release Syndrome

Event	Management
Grade 1 [a]<br>Fever [b] with or without constitutional symptoms	• Immediately interrupt infusion.<br>• Upon symptom resolution, wait for 30 minutes and then restart infusion at half the rate being given at the time of event onset.<br>• If the infusion is tolerated at the reduced rate for 30 minutes, the infusion rate may be increased to the original rate.<br>• If symptoms recur, discontinue infusion of this dose.<br>• Administer symptomatic treatment, [c] including maintenance of IV fluids for hydration.<br>• In case of rapid decline or prolonged CRS (> 2 days) or in patients with significant symptoms and/or comorbidities, consider managing as per Grade 2.<br>• For subsequent infusions, consider administration of oral premedication with antihistamines, anti-pyretics, and/or analgesics, and monitor closely for IRRs and/or CRS.
Grade 2 [a]<br>Fever [b] with hypotension not requiring vasopressors<br>**and/or**<br>Hypoxia requiring low-flow oxygen [d] by nasal cannula or blow-by	• Immediately interrupt infusion.<br>• Upon symptom resolution, wait for 30 minutes and then restart infusion at half the rate being given at the time of event onset.<br>• If symptoms recur, discontinue infusion of this dose.<br>• Administer symptomatic treatment. [c]<br>• For hypotension, administer IV fluid bolus as needed.<br>• Monitor cardiopulmonary and other organ function closely (in the ICU, if appropriate). Administer IV fluids as clinically indicated, and manage constitutional symptoms and organ toxicities as per institutional practice.<br>• Rule out other inflammatory conditions that can mimic CRS (e.g., sepsis). If no improvement within 24 hours, initiate workup and assess for signs and symptoms of HLH or MAS as described in this appendix.<br>• Consider IV corticosteroids (e.g., methylprednisolone 2 mg/kg/day or dexamethasone 10 mg every 6 hours).<br>• Consider anti-cytokine therapy. [e]<br>• Consider hospitalization until complete resolution of symptoms. If no improvement within 24 hours, manage as per Grade 3, that is, hospitalize patient (monitoring in the ICU is recommended), permanently discontinue atezolizumab, and contact Medical Monitor.<br>• If symptoms resolve to Grade 1 or better for 3 consecutive days, the next dose of atezolizumab may be administered. For subsequent infusions, consider administration of oral premedication with antihistamines, anti-pyretics, and/or analgesics and monitor closely for IRRs and/or CRS.<br>• If symptoms do not resolve to Grade 1 or better for 3 consecutive days, contact Medical Monitor.

Table 7. Management Guidelines for Infusion-Related Reactions and Cytokine-Release Syndrome (cont.)

Event	Management
Grade 3 [a]<br>Fever [b] with hypotension requiring a vasopressor (with or without vasopressin)<br>**and/or**<br>Hypoxia requiring high-flow oxygen [d] by nasal cannula, face mask, non-rebreather mask, or Venturi mask	• Permanently discontinue atezolizumab and contact Medical Monitor. [f]<br>• Administer symptomatic treatment. [c]<br>• For hypotension, administer IV fluid bolus and vasopressor as needed.<br>• Monitor cardiopulmonary and other organ function closely; monitoring in the ICU is recommended. Administer IV fluids as clinically indicated, and manage constitutional symptoms and organ toxicities as per institutional practice.<br>• Rule out other inflammatory conditions that can mimic CRS (e.g., sepsis). If no improvement within 24 hours, initiate workup and assess for signs and symptoms of HLH or MAS as described in this appendix.<br>• Administer IV corticosteroids (e.g., methylprednisolone 2 mg/kg/day or dexamethasone 10 mg every 6 hours).<br>• Consider anti-cytokine therapy. [e]<br>• Hospitalize patient until complete resolution of symptoms. If no improvement within 24 hours, manage as per Grade 4, that is, admit patient to ICU and initiate hemodynamic monitoring, mechanical ventilation, and/or IV fluids and vasopressors as needed; for patients who are refractory to anti-cytokine therapy, experimental treatments may be considered at the discretion of the investigator and in consultation with the Medical Monitor.
Grade 4 [a]<br>Fever [b] with hypotension requiring multiple vasopressors (excluding vasopressin)<br>**and/or**<br>Hypoxia requiring oxygen by positive pressure (e.g., CPAP, BiPAP, intubation and mechanical ventilation)	• Permanently discontinue atezolizumab and contact Medical Monitor. [f]<br>• Administer symptomatic treatment. [c]<br>• Admit patient to ICU and initiate hemodynamic monitoring, mechanical ventilation, and/or IV fluids and vasopressors as needed. Monitor other organ function closely. Manage constitutional symptoms and organ toxicities as per institutional practice.<br>• Rule out other inflammatory conditions that can mimic CRS (e.g., sepsis). If no improvement within 24 hours, initiate workup and assess for signs and symptoms of HLH or MAS as described in this appendix.<br>• Administer IV corticosteroids (e.g., methylprednisolone 2 mg/kg/day or dexamethasone 10 mg every 6 hours).<br>• Consider anti-cytokine therapy. [e] For patients who are refractory to anti-cytokine therapy, experimental treatments [g] may be considered at the discretion of the investigator and in consultation with the Medical Monitor.<br>• Hospitalize patient until complete resolution of symptoms.

Table 7. Management Guidelines for Infusion-Related Reactions and Cytokine-Release Syndrome (cont.)

ASTCT = American Society for Transplantation and Cellular Therapy; BiPAP = bi-level positive airway pressure; CAR = chimeric antigen receptor; CPAP = continuous positive airway pressure; CRS = cytokine-release syndrome; CTCAE = Common Terminology Criteria for Adverse Events; eCRF = electronic Case Report Form; HLH hemophagocytic lymphohistiocytosis; ICU = intensive care unit; IRR infusion-related reaction; MAS macrophage activation syndrome; NCCN = National Cancer Comprehensive Network; NCI = National Cancer Institute.

Note: The management guidelines have been adapted from NCCN guidelines for management of CAR T-cell–related toxicities (Version 2.2019).

[a] Grading system for management guidelines is based on ASTCT consensus grading for CRS. NCI CTCAE should be used when reporting severity of IRRs, CRS, or organ toxicities associated with CRS on the Adverse Event eCRF. Organ toxicities associated with CRS should not influence overall CRS grading.

[b] Fever is defined as temperature ≥ 38°C not attributable to any other cause. In patients who develop CRS and then receive anti-pyretic, anti-cytokine, or corticosteroid therapy, fever is no longer required when subsequently determining event severity (grade). In this case, the grade is driven by the presence of hypotension and/or hypoxia.

[c] Symptomatic treatment may include oral or IV antihistamines, anti-pyretics, analgesics, bronchodilators, and/or oxygen. For bronchospasm, urticaria, or dyspnea, additional treatment may be administered as per institutional practice.

[d] Low flow is defined as oxygen delivered at ≤ 6 L/min, and high flow is defined as oxygen delivered at > 6 L/min.

[e] There are case reports where anti-cytokine therapy has been used for treatment of CRS with immune checkpoint inhibitors (Rotz et al. 2017; Adashek and Feldman 2019), but data are limited, and the role of such treatment in the setting of antibody-associated CRS has not been established.

[f] Resumption of atezolizumab may be considered in patients who are deriving benefit and have fully recovered from the event. Patients can be re-challenged with atezolizumab only after approval has been documented by both the investigator (or an appropriate delegate) and the Medical Monitor. For subsequent infusions, administer oral premedication with antihistamines, anti-pyretics, and/or analgesics, and monitor closely for IRRs and/or CRS. Premedication with corticosteroids and extending the infusion time may also be considered after consulting the Medical Monitor and considering the benefit–risk ratio.

[g] Refer to Riegler et al. (2019) for information on experimental treatments for CRS.

Pancreatic Events

Symptoms of abdominal pain associated with elevations of amylase and lipase, suggestive of pancreatitis, have been associated with the administration of atezolizumab. The differential diagnosis of acute abdominal pain should include pancreatitis. Appropriate workup should include an evaluation for ductal obstruction, as well as serum amylase and lipase tests. Management guidelines for pancreatic events, including pancreatitis, are provided in Table 8.**Error! Reference source not found.** Management Guidelines for Pancreatic Events, Including Pancreatitis

Event	Management
Amylase and/or lipase elevation, Grade 2	**Amylase and/or lipase > 1.5–2.0 × ULN:**<br>• Continue atezolizumab.<br>• Monitor amylase and lipase weekly.<br>• For prolonged elevation (e.g., > 3 weeks), consider treatment with corticosteroids equivalent to 10 mg/day oral prednisone.<br>**Asymptomatic with amylase and/or lipase > 2.0–5.0 × ULN:**<br>• Treat as a Grade 3 event.
Amylase and/or lipase elevation, Grade 3 or 4	• Withhold atezolizumab for up to 12 weeks after event onset. [a]<br>• Refer patient to GI specialist.<br>• Monitor amylase and lipase every other day.<br>• If no improvement, consider treatment with corticosteroids equivalent to 1–2 mg/kg/day oral prednisone.<br>• If event resolves to Grade 1 or better, resume atezolizumab. [b]<br>• If event does not resolve to Grade 1 or better while withholding atezolizumab, permanently discontinue atezolizumab and contact Medical Monitor. [c]<br>• For recurrent events, permanently discontinue atezolizumab and contact Medical Monitor. [c]

GI = gastrointestinal.

[a] Atezolizumab may be withheld for a longer period of time (i.e., > 12 weeks after event onset) to allow for corticosteroids (if initiated) to be reduced to the equivalent of ≤ 10 mg/day oral prednisone. The acceptable length of the extended period of time must be agreed upon by the investigator and the Medical Monitor.

[b] If corticosteroids have been initiated, they must be tapered over ≥ 1 month to the equivalent of ≤ 10 mg/day oral prednisone before atezolizumab can be resumed.

[c] Resumption of atezolizumab may be considered in patients who are deriving benefit and have fully recovered from the immune-mediated event. Patients can be re-challenged with atezolizumab only after approval has been documented by both the investigator (or an appropriate delegate) and the Medical Monitor.

Table 8. Management Guidelines for Pancreatic Events, Including Pancreatitis (cont.)

Event	Management
Immune-mediated pancreatitis, Grade 2 or 3	• Withhold atezolizumab for up to 12 weeks after event onset. [a]<br>• Refer patient to GI specialist.<br>• Initiate treatment with corticosteroids equivalent to 1–2 mg/kg/day IV methylprednisolone and convert to 1–2 mg/kg/day oral prednisone or equivalent upon improvement.<br>• If event resolves to Grade 1 or better, resume atezolizumab. [b]<br>• If event does not resolve to Grade 1 or better while withholding atezolizumab, permanently discontinue atezolizumab and contact Medical Monitor. [c]<br>• For recurrent events, permanently discontinue atezolizumab and contact Medical Monitor. [c]
Immune-mediated pancreatitis, Grade 4	• Permanently discontinue atezolizumab and contact Medical Monitor. [c]<br>• Refer patient to GI specialist.<br>• Initiate treatment with corticosteroids equivalent to 1–2 mg/kg/day IV methylprednisolone and convert to 1–2 mg/kg/day oral prednisone or equivalent upon improvement.<br>• If event does not improve within 48 hours after initiating corticosteroids, consider adding an immunosuppressive agent.<br>• If event resolves to Grade 1 or better, taper corticosteroids over ≥ 1 month.

GI = gastrointestinal.

[a] Atezolizumab may be withheld for a longer period of time (i.e., > 12 weeks after event onset) to allow for corticosteroids (if initiated) to be reduced to the equivalent of ≤ 10 mg/day oral prednisone. The acceptable length of the extended period of time must be agreed upon by the investigator and the Medical Monitor.

[b] If corticosteroids have been initiated, they must be tapered over ≥ 1 month to the equivalent of ≤ 10 mg/day oral prednisone before atezolizumab can be resumed.

[c] Resumption of atezolizumab may be considered in patients who are deriving benefit and have fully recovered from the immune-mediated event. Patients can be re-challenged with atezolizumab only after approval has been documented by both the investigator (or an appropriate delegate) and the Medical Monitor.

Dermatologic Events

Treatment-emergent rash has been associated with atezolizumab. The majority of cases of rash were mild in severity and self limited, with or without pruritus. A dermatologist should evaluate persistent and/or severe rash or pruritus. A biopsy should be considered unless contraindicated. Management guidelines for dermatologic events are provided in Table 9.

Table 9. Management Guidelines for Dermatologic Events

Event	Management
Dermatologic event, Grade 1	• Continue atezolizumab.<br>• Consider treatment with topical corticosteroids and/or other symptomatic therapy (e.g., antihistamines).
Dermatologic event, Grade 2	• Continue atezolizumab.<br>• Consider patient referral to dermatologist.<br>• Initiate treatment with topical corticosteroids.<br>• Consider treatment with higher-potency topical corticosteroids if event does not improve.
Dermatologic event, Grade 3	• Withhold atezolizumab for up to 12 weeks after event onset. [a]<br>• Refer patient to dermatologist.<br>• Initiate treatment with corticosteroids equivalent to 10 mg/day oral prednisone, increasing dose to 1–2 mg/kg/day if event does not improve within 48–72 hours.<br>• If event resolves to Grade 1 or better, resume atezolizumab. [b]<br>• If event does not resolve to Grade 1 or better while withholding atezolizumab, permanently discontinue atezolizumab and contact Medical Monitor. [c]
Dermatologic event, Grade 4	• Permanently discontinue atezolizumab and contact Medical Monitor. [c]

[a] Atezolizumab may be withheld for a longer period of time (i.e., > 12 weeks after event onset) to allow for corticosteroids (if initiated) to be reduced to the equivalent of ≤ 10 mg/day oral prednisone. The acceptable length of the extended period of time must be agreed upon by the investigator and the Medical Monitor.

[b] If corticosteroids have been initiated, they must be tapered over ≥ 1 month to the equivalent of ≤ 10 mg/day oral prednisone before atezolizumab can be resumed.

[c] Resumption of atezolizumab may be considered in patients who are deriving benefit and have fully recovered from the immune-mediated event. Patients can be re-challenged with atezolizumab only after approval has been documented by both the investigator (or an appropriate delegate) and the Medical Monitor.

Neurologic Events

Myasthenia gravis and Guillain-Barré syndrome have been observed with single-agent atezolizumab. Patients may present with signs and symptoms of sensory and/or motor neuropathy. Diagnostic workup is essential for an accurate characterization to differentiate between alternative etiologies. Management guidelines for neurologic disorders are provided in Table 10.

Table 10. Management Guidelines for Neurologic Disorders

Event	Management
Immune-mediated neuropathy, Grade 1	• Continue atezolizumab.<br>• Investigate etiology.
Immune-mediated neuropathy, Grade 2	• Withhold atezolizumab for up to 12 weeks after event onset. [a]<br>• Investigate etiology.<br>• Initiate treatment as per institutional guidelines.<br>• If event resolves to Grade 1 or better, resume atezolizumab. [b]<br>• If event does not resolve to Grade 1 or better while withholding atezolizumab, permanently discontinue atezolizumab and contact Medical Monitor. [c]
Immune-mediated neuropathy, Grade 3 or 4	• Permanently discontinue atezolizumab and contact Medical Monitor. [c]<br>• Initiate treatment as per institutional guidelines.
Myasthenia gravis and Guillain-Barré syndrome (any grade)	• Permanently discontinue atezolizumab and contact Medical Monitor. [c]<br>• Refer patient to neurologist.<br>• Initiate treatment as per institutional guidelines.<br>• Consider initiation of corticosteroids equivalent to 1–2 mg/kg/day oral or IV prednisone.

[a] Atezolizumab may be withheld for a longer period of time (i.e., $>$ 12 weeks after event onset) to allow for corticosteroids (if initiated) to be reduced to the equivalent of $\leq$ 10 mg/day oral prednisone. The acceptable length of the extended period of time must be agreed upon by the investigator and the Medical Monitor.

[b] If corticosteroids have been initiated, they must be tapered over $\geq$ 1 month to the equivalent of $\leq$ 10 mg/day oral prednisone before atezolizumab can be resumed.

[c] Resumption of atezolizumab may be considered in patients who are deriving benefit and have fully recovered from the immune-mediated event. Patients can be re-challenged with atezolizumab only after approval has been documented by both the investigator (or an appropriate delegate) and the Medical Monitor.

Immune-mediated meningoencephalitis

Immune-mediated meningoencephalitis is an identified risk associated with the administration of atezolizumab. Immune-mediated meningoencephalitis should be suspected in any patient presenting with signs or symptoms suggestive of meningitis or encephalitis, including, but not limited to, headache, neck pain, confusion, seizure, motor or sensory dysfunction, and altered or depressed level of consciousness. Encephalopathy from metabolic or electrolyte imbalances needs to be distinguished from potential meningoencephalitis resulting from infection (bacterial, viral, or fungal) or progression of malignancy, or secondary to a paraneoplastic process.

All patients being considered for meningoencephalitis should be urgently evaluated with a CT scan and/or MRI scan of the brain to evaluate for metastasis, inflammation, or edema. If deemed safe by the treating physician, a lumbar puncture should be performed and a neurologist should be consulted.

Patients with signs and symptoms of meningoencephalitis, in the absence of an identified alternate etiology, should be treated according to the guidelines in Table 11.

Table 11 Management Guidelines for Immune-Mediated Meningoencephalitis

Event	Management
Immune-mediated meningoencephalitis, all grades	• Permanently discontinue atezolizumab and contact Medical Monitor. [a]<br>• Refer patient to neurologist.<br>• Initiate treatment with corticosteroids equivalent to 1–2 mg/kg/day IV methylprednisolone and convert to 1–2 mg/kg/day oral prednisone or equivalent upon improvement.<br>• If event does not improve within 48 hours after initiating corticosteroids, consider adding an immunosuppressive agent.<br>• If event resolves to Grade 1 or better, taper corticosteroids over ≥ 1 month.

[a] Resumption of atezolizumab may be considered in patients who are deriving benefit and have fully recovered from the immune-mediated event. Patients can be re-challenged with atezolizumab only after approval has been documented by both the investigator (or an appropriate delegate) and the Medical Monitor.

Renal Events

Immune-mediated nephritis has been associated with the administration of atezolizumab. Eligible patients must have adequate renal function. Renal function, including serum creatinine, should be monitored throughout study treatment. Patients with abnormal renal function should be evaluated and treated for other more common etiologies (including prerenal and postrenal causes, and concomitant medications such as non-steroidal anti-inflammatory drugs). Refer the patient to a renal specialist if clinically indicated. A renal biopsy may be required to enable a definitive diagnosis and appropriate treatment.

Patients with signs and symptoms of nephritis, in the absence of an identified alternate etiology, should be treated according to the guidelines in Table12.

Table 12. Management Guidelines for Renal Events

Event	Management
Renal event, Grade 1	• Continue atezolizumab.<br>• Monitor kidney function, including creatinine, closely until values resolve to within normal limits or to baseline values.
Renal event, Grade 2	• Withhold atezolizumab for up to 12 weeks after event onset. [a]<br>• Refer patient to renal specialist.<br>• Initiate treatment with corticosteroids equivalent to 1–2 mg/kg/day oral prednisone.<br>• If event resolves to Grade 1 or better, resume atezolizumab. [b]<br>• If event does not resolve to Grade 1 or better while withholding atezolizumab, permanently discontinue atezolizumab and contact Medical Monitor. [c]
Renal event, Grade 3 or 4	• Permanently discontinue atezolizumab and contact Medical Monitor.<br>• Refer patient to renal specialist and consider renal biopsy.<br>• Initiate treatment with corticosteroids equivalent to 1–2 mg/kg/day oral prednisone.<br>• If event does not improve within 48 hours after initiating corticosteroids, consider adding an immunosuppressive agent.<br>• If event resolves to Grade 1 or better, taper corticosteroids over ≥ 1 month.

[a] Atezolizumab may be withheld for a longer period of time (i.e., > 12 weeks after event onset) to allow for corticosteroids (if initiated) to be reduced to the equivalent of ≤ 10 mg/day oral prednisone. The acceptable length of the extended period of time must be agreed upon by the investigator and the Medical Monitor.

[b] If corticosteroids have been initiated, they must be tapered over ≥ 1 month to the equivalent of ≤ 10 mg/day oral prednisone before atezolizumab can be resumed.

[c] Resumption of atezolizumab may be considered in patients who are deriving benefit and have fully recovered from the immune-mediated event. Patients can be re-challenged with atezolizumab only after approval has been documented by both the investigator (or an appropriate delegate) and the Medical Monitor.

Immune-mediated myositis

Immune-mediated myositis has been associated with the administration of atezolizumab. Myositis or inflammatory myopathies are a group of disorders sharing the common feature of inflammatory muscle injury; dermatomyositis and polymyositis are among the most common disorders. Initial diagnosis is based on clinical (muscle weakness, muscle pain, skin rash in dermatomyositis), biochemical (serum creatine kinase increase), and imaging (electromyography/MRI) features, and is confirmed with a muscle biopsy.

Patients with signs and symptoms of myositis, in the absence of an identified alternate etiology, should be treated according to the guidelines in 13.

Table 13. Management Guidelines for Immune-Mediated Myositis

Event	Management
Immune-mediated myositis, Grade 1	• Continue atezolizumab.<br>• Refer patient to rheumatologist or neurologist.<br>• Initiate treatment as per institutional guidelines.
Immune-mediated myositis, Grade 2	• Withhold atezolizumab for up to 12 weeks after event onset [a] and contact Medical Monitor.<br>• Refer patient to rheumatologist or neurologist.<br>• Initiate treatment as per institutional guidelines.<br>• Consider treatment with corticosteroids equivalent to 1–2 mg/kg/day IV methylprednisolone and convert to 1–2 mg/kg/day oral prednisone or equivalent upon improvement.<br>• If corticosteroids are initiated and event does not improve within 48 hours after initiating corticosteroids, consider adding an immunosuppressive agent.<br>• If event resolves to Grade 1 or better, resume atezolizumab. [b]<br>• If event does not resolve to Grade 1 or better while withholding atezolizumab, permanently discontinue atezolizumab and contact Medical Monitor. [c]

[a] Atezolizumab may be withheld for a longer period of time (i.e., > 12 weeks after event onset) to allow for corticosteroids (if initiated) to be reduced to the equivalent of ≤ 10 mg/day oral prednisone. The acceptable length of the extended period of time must be agreed upon by the investigator and the Medical Monitor.

[b] If corticosteroids have been initiated, they must be tapered over ≥ 1 month to the equivalent of ≤ 10 mg/day oral prednisone before atezolizumab can be resumed.

[c] Resumption of atezolizumab may be considered in patients who are deriving benefit and have fully recovered from the immune-mediated event. Patients can be re-challenged with atezolizumab only after approval has been documented by both the investigator (or an appropriate delegate) and the Medical Monitor.

Table 13. Management Guidelines for Immune-Mediated Myositis (cont.)

Immune-mediated myositis, Grade 3	• Withhold atezolizumab for up to 12 weeks after event onset [a] and contact Medical Monitor.<br>• Refer patient to rheumatologist or neurologist.<br>• Initiate treatment as per institutional guidelines.<br>• Respiratory support may be required in more severe cases.<br>• Initiate treatment with corticosteroids equivalent to 1–2 mg/kg/day IV methylprednisolone, or higher-dose bolus if patient is severely compromised (e.g., cardiac or respiratory symptoms, dysphagia, or weakness that severely limits mobility); convert to 1–2 mg/kg/day oral prednisone or equivalent upon improvement.<br>• If event does not improve within 48 hours after initiating corticosteroids, consider adding an immunosuppressive agent.<br>• If event resolves to Grade 1 or better, resume atezolizumab. [b]<br>• If event does not resolve to Grade 1 or better while withholding atezolizumab, permanently discontinue atezolizumab and contact Medical Monitor. [c]<br>• For recurrent events, treat as a Grade 4 event.
Immune-mediated myositis, Grade 4	• Permanently discontinue atezolizumab and contact Medical Monitor. [c]<br>• Refer patient to rheumatologist or neurologist.<br>• Initiate treatment as per institutional guidelines.<br>• Respiratory support may be required in more severe cases.<br>• Initiate treatment with corticosteroids equivalent to 1–2 mg/kg/day IV methylprednisolone, or higher-dose bolus if patient is severely compromised (e.g., cardiac or respiratory symptoms, dysphagia, or weakness that severely limits mobility); convert to 1–2 mg/kg/day oral prednisone or equivalent upon improvement.<br>• If event does not improve within 48 hours after initiating corticosteroids, consider adding an immunosuppressive agent.<br>• If event resolves to Grade 1 or better, taper corticosteroids over ≥ 1 month.

[a] Atezolizumab may be withheld for a longer period of time (i.e., > 12 weeks after event onset) to allow for corticosteroids (if initiated) to be reduced to the equivalent of ≤ 10 mg/day oral prednisone. The acceptable length of the extended period of time must be agreed upon by the investigator and the Medical Monitor.

[b] If corticosteroids have been initiated, they must be tapered over ≥ 1 month to the equivalent of ≤ 10 mg/day oral prednisone before atezolizumab can be resumed.

[c] Resumption of atezolizumab may be considered in patients who are deriving benefit and have fully recovered from the immune-mediated event. Patients can be re-challenged with atezolizumab only after approval has been documented by both the investigator (or an appropriate delegate) and the Medical Monitor.

<u>Hemophagocytic Lymphohistiocytosis and Macrophage Activation Syndrome</u>

Immune-mediated reactions may involve any organ system and may lead to hemophagocytic lymphohistiocytosis (HLH) and macrophage activation syndrome (MAS).

Clinical and laboratory features of severe CRS overlap with HLH, and HLH should be considered when CRS presentation is atypical or prolonged.

Patients with suspected HLH should be diagnosed according to published criteria by McClain and Eckstein (2014). A patient should be classified as having HLH if five of the following eight criteria are met:

- Fever ≥ 38.5°C
- Splenomegaly
- Peripheral blood cytopenia consisting of at least two of the following:
  - Hemoglobin < 90 g/L (9 g/dL) (< 100 g/L [10 g/dL] for infants < 4 weeks old)
  - Platelet count < 100×10^9/L (100,000/μL)
  - ANC < 1.0×10^9/L (1000/μL)
- Fasting triglycerides > 2.992 mmol/L (265 mg/dL) and/or fibrinogen < 1.5 g/L (150 mg/dL)
- Hemophagocytosis in bone marrow, spleen, lymph node, or liver
- Low or absent natural killer cell activity
- Ferritin > 500 mg/L (500 ng/mL)
- Soluble interleukin 2 (IL-2) receptor (soluble CD25) elevated ≥ 2 standard deviations above age-adjusted laboratory-specific norms

Patients with suspected MAS should be diagnosed according to published criteria for systemic juvenile idiopathic arthritis by Ravelli et al. (2016). A febrile patient should be classified as having MAS if the following criteria are met:

- Ferritin > 684 mg/L (684 ng/mL)
- At least two of the following:
  - Platelet count ≤ 181×10^9/L (181,000/μL)
  - AST ≥ 48 U/L
  - Triglycerides > 1.761 mmol/L (156 mg/dL)
  - Fibrinogen ≤ 3.6 g/L (360 mg/dL)

Patients with suspected HLH or MAS should be treated according to the guidelines in Table 14.

Table 14. Management Guidelines for Suspected Hemophagocytic Lymphohistiocytosis or Macrophage Activation Syndrome

Event	Management
Suspected HLH or MAS	• Permanently discontinue atezolizumab and contact Medical Monitor.<br>• Consider patient referral to hematologist.<br>• Initiate supportive care, including intensive care monitoring if indicated per institutional guidelines.<br>• Consider initiation of IV corticosteroids, an immunosuppressive agent, and/or anti-cytokine therapy.<br>• If event does not respond to treatment within 24 hours, contact Medical Monitor and initiate treatment as appropriate according to published guidelines (La Rosée 2015; Schram and Berliner 2015; La Rosée et al. 2019).<br>• If event resolves to Grade 1 or better, taper corticosteroids over ≥ 1 month.

HLH = hemophagocytic lymphohistiocytosis; MAS = macrophage activation syndrome.

9 HERCEPTIN (TRASTUZUMAB)

Investigators should also consult the local prescribing information if the IMP is approved in your country.

9.1 Standard dose:

• Administration every three weeks

The recommended initial loading dose is 8 mg / kg body weight. The recommended maintenance dose at three week intervals is 6 mg / kg body weight, starting three weeks after the loading dose.

• Weekly administration

The recommended initial loading dose of Herceptin is 4 mg / kg body weight. The recommended weekly maintenance dose of Herceptin is 2 mg / kg body weight, starting one week after the loading dose.
Herceptin loading dose should be administered as a 90- minute intravenous infusion. If the initial loading dose was well tolerated, the subsequent doses can be administered as a 30-minute infusion.

For patients with metastatic disease, treatment with Herceptin should be continued until progression of disease
No reductions in the dose of Herceptin were made during clinical trials. Patients may continue therapy during periods of reversible, chemotherapy-induced myelosuppression but they should be monitored carefully for complications of neutropenia during this time.
If the patient has missed a dose of Herceptin by one week or less, then the usual maintenance dose (weekly regimen: 2 mg/kg; three-weekly regimen: 6 mg/kg) should be administered as soon as possible. Do not wait until the next planned cycle. Subsequent maintenance doses should be administered 7 days or 21 days later according to the weekly or three-weekly schedules, respectively.
If the patient has missed a dose of Herceptin by more than one week, a re-loading dose of Herceptin should be administered over approximately 90 minutes (weekly regimen: 4 mg/kg; three-weekly regimen: 8 mg/kg) as soon as possible. Subsequent Herceptin maintenance doses (weekly regimen:
2 mg/kg; three-weekly regimen 6 mg/kg respectively) should be administered 7 days or 21 days later according to the weekly or three-weekly schedules respectively.

9.2 Contraindications

Herceptin is contraindicated in patients with known hypersensitivity to Herceptin or to any of its excipients.

9.3 Warnings and Precautions

Patients with dyspnea at rest due to complications of advanced malignancy and co-morbidities may be at increased risk of pulmonary events and of a fatal infusion reaction (severe reactions include bronchospasm, anaphylaxis, angioedema, hypoxia, and severe hypotension). Therefore, these patients should not be treated with Herceptin.

Infusion/ARRs and Hypersensitivity

Infusion/ARRs and hypersensitivity are known to occur with the administration of Herceptin. Pre-medication may be used to reduce risk of occurrence of ARRs. (hypersensitivity, drug hypersensitivity, infusion-related reaction, and injection site hypersensitivity). ARRs may be clinically difficult to distinguish from hypersensitivity reactions. Serious ARRs to Herceptin including dyspnea, hypotension, wheezing, bronchospasm, tachycardia, reduced oxygen saturation and respiratory distress, supraventricular tachyarrhythmia and urticaria have been reported. Patients should be observed for ARRs. Interruption of an IV infusion may help to control such symptoms and the infusion may be resumed when symptoms abate. These symptoms can be treated with an analgesic/antipyretic such as meperidine or paracetamol, or an antihistamine such as diphenhydramine. Serious reactions have been treated successfully with supportive therapy such as oxygen, beta-agonists and corticosteroids. In rare cases, these reactions are associated with a clinical course culminating in a fatal outcome.

Cardiac dysfunction

Patients treated with Herceptin are at increased risk of developing CHF NYHA Class II-IV or asymptomatic cardiac dysfunction. These events have been observed in patients receiving Herceptin therapy alone or in

combination with taxane following anthracycline (doxorubicin or epirubicin)–containing chemotherapy. This may be moderate to severe and has been associated with death. In addition, caution should be exercised in treating patients with increased cardiac risk (e.g., hypertension, documented coronary artery disease, CHF, diastolic dysfunction, older age). Population pharmacokinetic model simulations indicate that trastuzumab may persist in the circulation for up to 7 months after stopping Herceptin IV or SC treatment. Patients who receive anthracycline after stopping Herceptin may also be at increased risk of cardiac dysfunction.

If possible, physicians should avoid anthracycline-based therapy for up to 7 months after stopping Herceptin. If anthracyclines are used, the patient's cardiac function should be monitored carefully.

Candidates for treatment with Herceptin, especially those with prior exposure to an anthracycline, should undergo baseline cardiac assessment including history and physical examination, and ECG echocardiogram, and/or multigated acquisition scanning (MUGA) scan. Monitoring may help to identify patients who develop cardiac dysfunction, including signs and symptoms of CHF. Cardiac assessments, as performed at baseline, should be repeated every 3 months during treatment and every 6 months following discontinuation of treatment until 24 months from the last administration of Herceptin.

If LVEF drops 10 ejection points from baseline and to below 50%, Herceptin should be withheld and a repeat LVEF assessment performed within approximately 3 weeks. If LVEF has not improved, or declined further, or clinically significant CHF has developed, discontinuation of Herceptin should be strongly considered, unless the benefits for the individual patient are deemed to outweigh the risks. Patients who develop asymptomatic cardiac dysfunction may benefit from more frequent monitoring (e.g. every 6–8 weeks). If patients have a continued decrease in left ventricular function, but remain asymptomatic, the physician should consider discontinuing therapy if no clinical benefit of Herceptin therapy has been seen. The safety of continuation or resumption of trastuzumab in patients who experience cardiac dysfunction has not been prospectively studied. If symptomatic cardiac failure develops during Herceptin therapy, it should be treated with standard medications for heart failure (HF). In the pivotal trials, most patients who developed HF or asymptomatic cardiac dysfunction improved with standard HF treatment consisting of an angiotensin converting enzyme inhibitor or angiotensin receptor blocker and a β-blocker. The majority of patients with cardiac symptoms and evidence of a clinical benefit of Herceptin treatment continued with Herceptin without additional clinical cardiac events.

Risk factors for a cardiac event identified in four large adjuvant studies included advanced age (> 50 years), low level of baseline and declining LVEF (< 55%), low LVEF prior to or following the initiation of paclitaxel treatment, Herceptin treatment, and prior or concurrent use of anti-hypertensive medications. In patients receiving Herceptin after completion of adjuvant chemotherapy the risk of cardiac dysfunction was associated with a higher cumulative dose of anthracycline given prior to initiation of Herceptin and a high body mass index (BMI > 25 kg/m2).

Pulmonary adverse drug reactions

Severe pulmonary events have been reported with the use of Herceptin IV in the post-marketing setting. These events have occasionally resulted in fatal outcome and may occur as part of an IRR/ARR or with a delayed onset. In addition, cases of interstitial lung disease including lung infiltrates acute respiratory distress syndrome, pneumonia, pneumonitis, pleural effusion, respiratory distress, acute pulmonary oedema and respiratory insufficiency have been reported. Risk factors associated with interstitial lung disease include prior or concomitant therapy with other anti-neoplastic therapies known to be associated with it such as taxanes, gemcitabine, vinorelbine and radiation therapy.

Benzyl alcohol

When administering Herceptin to a patient with a known hypersensitivity to benzyl alcohol, Herceptin should be reconstituted with water for injection. In case Herceptin is reconstituted with water for injection, only one dose per Herceptin vial should be used. The reconstituted solution should be used immediately. Any unused portion must be discarded.

Table 1: trastuzumab adverse events

System organ class	Adverse reaction	Frequency

Infections and infestations	Infection	Very common
	Nasopharyngitis	Very common
	Neutropenic sepsis	Common
	Cystitis	Common
	Herpes zoster	Common
	Influenza	Common
	Sinusitis	Common
	Skin infection	Common
	Rhinitis	Common
	Upper respiratory tract infection	Common
	Urinary tract infection	Common
	Erysipelas	Common
	Cellulitis	Common
	Pharyngitis	Common
	Sepsis	Uncommon
Neoplasms benign, malignant and unspecified (incl. Cysts and polyps)	Malignant neoplasm progression	Not known
	Neoplasm progression	Not known
Blood and lymphatic system disorders	Febrile neutropenia	Very common
	Anaemia	Very common
	Neutropenia	Very common
	White blood cell count decreased/leukopenia	Very common
	Thrombocytopenia	Very common
	Hypoprothrombinaemia	Not known
	Immune thrombocytopenia	Not known
Immune system disorders	Hypersensitivity	Common
	+Anaphylactic reaction	Not known
	+Anaphylactic shock	Not known
Metabolism and nutrition disorders	Weight decreased/Weight loss	Very common
	Anorexia	Very common
	Tumour lysis syndrome	Not known
	Hyperkalaemia	Not known
Psychiatric disorders	Insomnia	Very common
	Anxiety	Common
	Depression	Common
	Thinking abnormal	Common
Nervous system disorders	[1]Tremor	Very common
	Dizziness	Very common
	Headache	Very common
	Paraesthesia	Very common
	Dysgeusia	Very common
	Peripheral neuropathy	Common

	Hypertonia	Common
	Somnolence	Common
	Ataxia	Common
	Paresis	Rare
	Brain oedema	Not known
Eye disorders	Conjunctivitis	Very common
	Lacrimation increased	Very common
	Dry eye	Common
	Papilloedema	Not known
	Retinal haemorrhage	Not known
Ear and labyrinth disorders	Deafness	Uncommon
Cardiac disorders	[1] Blood pressure decreased	Very common
	[1] Blood pressure increased	Very common
	[1] Heart beat irregular	Very common
	[1]Palpitation	Very common
	[1]Cardiac flutter	Very common
	Ejection fraction decreased*	Very common
	[+]Cardiac failure (congestive)<br>[+1]Supraventricular tachyarrhythmia	Common<br>Common
	Cardiomyopathy	Common
	Pericardial effusion	Uncommon
	Cardiogenic shock	Not known
	Pericarditis	Not known
	Bradycardia	Not known
	Gallop rhythm present	Not known
Vascular disorders	Hot flush	Very common
	[+1] Hypotension	Common
	Vasodilatation	Common
Respiratory, thoracic and mediastinal disorders	[+1]Wheezing<br>[+]Dyspnoea	Very common<br>Very common
	Cough	Very common
	Epistaxis	Very common
	Rhinorrhoea	Very common
	[+]Pneumonia	Common
	Asthma	Common
	Lung disorder<br>[+]Pleural effusion	Common<br>Common
	Pneumonitis	Rare
	[+]Pulmonary fibrosis<br>[+]Respiratory distress	Not known<br>Not known
	[+]Respiratory failure	Not known
	[+]Lung infiltration<br>[+]Acute pulmonary oedema	Not known<br>Not known
	[+]Acute respiratory distress syndrome<br>[+]Bronchospasm	Not known<br>Not known
	[+]Hypoxia	Not known

	+Oxygen saturation decreased	Not known
	Laryngeal oedema	Not known
	Orthopnoea	Not known
	Pulmonary oedema	Not known
	Interstitial lung disease	Not known
Gastrointestinal disorders	Diarrhoea	Very common
	Vomiting	Very common
	Nausea	Very common
	[1] Lip swelling	Very common
	Abdominal pain	Very common
	Dyspepsia	Very common
	Constipation	Very common
	Stomatitis	Very common
	Haemorrhoids	Common
	Dry mouth	Common
Hepatobiliary disorders	Hepatocellular injury	Common
	Hepatitis	Common
	Liver tenderness	Common
	Jaundice	Rare
	Hepatic failure	Not known
Skin and subcutaneous tissue disorders	Erythema	Very common
	Rash	Very common
	[1] Swelling face	Very common
	Alopecia	Very common
	Nail disorder	Very common
	Palmar-plantar erythrodysaesthesia syndrome	Very common
	Acne	Common
	Dry skin	Common
	Ecchymosis	Common
	Hyperhydrosis	Common
	Maculopapular rash	Common
	Pruritus	Common
	Onychoclasis	Common
	Dermatitis	Common
	Urticaria	Uncommon
	Angioedema	Not known
Musculoskeletal and connective tissue disorders	Arthralgia	Very common
	[1]Muscle tightness	Very common
	Myalgia	Very common
	Arthritis	Common
	Back pain	Common

	Bone pain	Common
	Muscle spasms	Common
	Neck Pain	Common
	Pain in extremity	Common
Renal and urinary disorders	Renal disorder	Common
	Glomerulonephritis membranous	Not known
	Glomerulonephropathy	Not known
	Renal failure	Not known
Pregnancy, puerperium and perinatal conditions	Oligohydramnios	Not known
	Renal hypoplasia	Not known
	Pulmonary hypoplasia	Not known
Reproductive system and breast disorders	Breast inflammation/mastitis	Common
General disorders and administration site conditions	Asthenia	Very common
	Chest pain	Very common
	Chills	Very common
	Fatigue	Very common
	Influenza-like symptoms	Very common
	Infusion related reaction	Very common
	Pain	Very common
	Pyrexia	Very common
	Mucosal inflammation	Very common
	Peripheral oedema	Very common
	Malaise	Common
	Oedema	Common
Injury, poisoning and procedural complications	Contusion	Common

+ Denotes adverse reactions that have been reported in association with a fatal outcome.
1 Denotes adverse reactions that are reported largely in association with Infusion-related reactions. Specific percentages for these are not available.

* Observed with combination therapy following anthracyclines and combined with taxanes

9.4 Special Populations

Pregnancy

Refer to appendix 1.

Nursing Mothers

It is not known whether Herceptin is secreted in human milk. As human IgG is secreted into human milk, and the potential for harm to the infant is unknown, breast-feeding should be avoided during Herceptin therapy.

Geriatric Patients

The safety of Herceptin has not been specifically tested in a geriatric population.
Detailed PK studies have not been conducted in elderly patients. Based on PPK analysis, age (< 65 [n = 1294]; > 65 [n = 288]) does not have a clinically meaningful effect on the pharmacokinetics of Herceptin.

Data suggest that the disposition of trastuzumab is not altered based on age. In clinical trials, elderly patients did not receive reduced doses of Herceptin.

9.5 Concomitant use with other medications

There have been no formal drug interaction studies performed with Herceptin in humans. Clinically significant interactions between Herceptin and the concomitant medications used in clinical trials have not been observed.

9.6 Overdose

Herceptin IV formulation:
There is no experience with overdosage in human clinical trials. Single doses higher than 10 mg/kg have not been tested.
Herceptin SC formulation:
Single doses of up to 960 mg have been administered with no reported untoward effect.

10 IPATASERTIB

Investigators should also consult the local prescribing information if the IMP is approved in your country.

10.1 Standard dose

Ipatasertib is currently not approved in any country or territory for any indication.

Continued clinical development is ongoing in clinical studies in combination with other anticancer therapies in patients with metastatic prostate and breast cancers.

Ipatasertib is used in ongoing phase III trial (IPATunity130 https://clinicaltrials.gov/ct2/show/NCT03337724) at 400 mg administered orally daily on Days 1-21 of each 28-day cycle, along with paclitaxel (80 mg/m2 IV over 1 hour) on Days 1, 8, and 15 of each 28-day cycle.

If the patient does not tolerate the once a day (QD) dosing of ipatasertib, dosing with food may be used to alleviate gastrointestinal symptoms, including nausea, vomiting, and/or diarrhea. No more than two dose reductions of patasertib per patient (i.e., doses < 200 mg/day of ipatasertib) will be allowed. Dose re-escalation is not permitted; if the patient continues to experience specified study drug-related adverse events after the second reduction, treatment should be discontinued.

Table 1 Suggested Dose Reductions for Ipatasertib, Placebo, and/or Paclitaxel

Dose Level [a]	Ipatasertib/Placebo	Paclitaxel
Starting dose	400 mg	80 mg/m^2
First dose reduction[b]	300 mg	65 mg/m^2
Second dose reduction[b]	200 mg	50 mg/m^2
Third dose reduction[b]	Discontinue	Discontinue

[a] If the patient continues to experience specified drug-related adverse events after second reduction, the treatment should be discontinued.

[b] *Note that dose modification(s) for ipatasertib/placebo and paclitaxel are independent (e.g., adverse event may lead to dose reduction for ipatasertib/placebo with no dose modification for paclitaxel).*

10.2 Contraindications

There are no known contraindications identified for ipatasertib.

10.3 Warnings and Precautions

<u>Hyperglicemia</u>

Because the PI3K-Akt-mTOR pathway is involved in glucose metabolism, inhibition of this signaling network and its target mTOR can cause hyperglycemia, which is a toxicity common to the class of PI3K-Akt-mTOR inhibitors. Ipatasertib-induced hyperglycemia is associated with transient insulin concentration changes and is generally asymptomatic.

Hyperglycemia, including cases of Grade 3, Grade 4, and a single case of Grade 5 hyperglycemia (from Study CO39303 when ipatasertib was administered in conjunction with prednisone/prednisolone (steroid) and abiraterone), has been reported in patients receiving ipatasertib treatment. Isolated cases of hyperglycemia associated with ketoacidosis or hyperosmolar conditions resulting in dehydration and renal insufficiency have also been observed, particularly in diabetic patients.

In general, patients with diabetes either requiring insulin therapy or with a baseline fasting glucose ≥150 mg/dL (8.3 mmol/L) or high HbA1c, suggesting poorly controlled diabetes, should be excluded from studies with ipatasertib. Fasting glucose levels should be carefully monitored Patients should be instructed to report symptoms associated with hyperglycemia such as thirst, frequent urination, and blurred vision. Hyperglycemia should be managed per institutional standards of care. Use of oral anti-hyperglycemic agents (e.g., metformin) for patients experiencing Grade ≥2 hyperglycemia should be considered. For Grade ≥3 hyperglycemia, ipatasertib dosing may be interrupted or reduced.

To diminish the risk of hypoglycemia, insulin should not be administered for asymptomatic hyperglycemia of any grade.

- Grade 1 fasting hyperglycemia (Range: > ULN−160 mg/dL [> ULN−8.9 mmol/L]): The patient may continue treatment with the ipatasertib or placebo, and chemotherapy. The patient may receive education on a diabetic diet and consider beginning home glucose monitoring at the discretion of the investigator. If glucose home monitoring is instituted, the ipatasertib or placebo treatment decision should be based on the morning fasting glucose value obtained prior to the dose of ipatasertib or placebo. The patient may be started on an oral anti-diabetic medication (e.g., metformin) at the discretion of the investigator.
- Grade 2 fasting hyperglycemia (Range: > 160−250 mg/dL [> 8.9−13.9 mmol/L]): The patient should be started on an oral anti-diabetic medication (e.g., metformin) and should receive education on a diabetic diet. The ipatasertib or placebo treatment decision should be based on the morning fasting glucose value obtained prior to the dose of ipatasertib or placebo. The patient may also continue treatment with the ipatasertib or placebo, and/or chemotherapy.
- Grade ≥ 3 fasting hyperglycemia (Range: > 250−500 mg/dL [> 13.9−27.8 mmol/L]): The ipatasertib or placebo should be held until fasting hyperglycemia has resolved to Grade ≤ 2, but may continue chemotherapy. The patient should start a diabetic diet and should start an oral anti-diabetic medication (e.g., metformin). The patient may begin home glucose monitoring (with fasting glucose checked prior to the ipatasertib daily dose). If fasting glucose levels recover to Grade ≤ 2 within 3 days, the ipatasertib or placebo may be resumed at the same dose; alternatively, the dose of ipatasertib or placebo may be reduced by one dose level (see Table 1) at the discretion of the investigator. If Grade ≥ 3 fasting hyperglycemia recurs within the same cycle, or if recovery of fasting glucose levels to Grade ≤ 2 takes 4 or more days, the dose of ipatasertib or placebo must be reduced by one dose level when treatment resumes.

<u>Gastrointestinal Toxicity</u>

Gastrointestinal toxicities, primarily manifested as nausea, vomiting, diarrhea, and oral mucositis, have been commonly observed in patients receiving ipatasertib treatment.

Severe GI toxicities may require dose delay and/or dose reduction and may potentially lead to volume depletion.

In general, patients should be closely monitored for GI effects and patients experiencing nausea, vomiting, stomatitis/oral mucositis, or diarrhea are to be treated and managed per standard of care, including hydration if clinically indicated to prevent renal insufficiency due to fluid depletion. If the GI effects are attributable to ipatasertib as assessed by the investigator, the ipatasertib dose may be interrupted or reduced. Patients whose GI symptoms cannot be adequately managed should discontinue ipatasertib.

Nausea/vomiting:

- Grade 1 nausea and/or vomiting: Maximum supportive care should be administered as needed at the discretion of the investigator.
- Grade 2 nausea and/or vomiting: Ondansetron (or equivalent anti-emetic) should be administered as needed.
- Grade ≥ 3 nausea and/or vomiting: Ondansetron (or equivalent anti-emetic) should be administered as needed. The investigator may employ a BID dosing of ipatasertib administration (equivalent to the total daily dose divided by half); alternatively, the dose of ipatasertib or placebo may be reduced by one dose level (see Table 1). Dose re-escalation of the ipatasertib may be permitted in subsequent cycles for patients who exhibit Grade ≤ 1 nausea/vomiting through at least one cycle.

Diarrhea: diarrhea should be managed with loperamide or per local guidelines and standard of care, including but not limited to therapy with diphenoxylate and atropine, codeine, or octreotide. Dose reductions for diarrhea should occur only if the symptoms persist despite treatment with adequate (combination) anti-diarrheal medications. If persistent diarrhea (more than 48 hours despite optimal medical treatment or dose hold) is attributable to the ipatasertib dosage modification guidelines for ipatasertib are outlined below:

- Grade 2 diarrhea: The investigator should initiate optimal medical management with loperamide as early as possible. If diarrhea is persistent (lasting longer than 48 hours despite medical management), second-line therapy may include (but not limited to) Lomotil (diphenoxylate and atropine), codeine or octreotide per local guidelines and standard of care. Ipatasertib dosing should be interrupted until improvement of

diarrhea to Grade ≤ 1, at which time ipatasertib may be resumed at the same dose for initial occurrence with consideration of maintenance loperamide dosing (i.e., 2 mg, 2 to 4 times daily). Investigators may reduce ipatasertib by one dose level (see Table 1) for recurrent Grade 2 diarrhea. Paclitaxel dose reduction or discontinuation can be considered if diarrhea persists even after ipatasertib discontinuation (see Table 1).

- Grade ≥ 3 diarrhea: Medical management of diarrhea per Grade 2 diarrhea above should be initiated as early as possible. Ipatasertib should be held until the diarrhea resolves to Grade ≤ 1, at which time maintenance loperamide dosing (i.e., 2 mg, 2 to 4 times daily) is recommended. The dose of ipatasertib will be reduced by one dose level when treatment resumes (see Table 1). If diarrhea persists following dose reduction of ipatasertib, an additional dose hold or reduction with ipatasertib should be considered (see Table 1).

Rash

Treatment-related rash, including cases of Grade 3 and Grade 4 rash, has occurred in patients receiving ipatasertib treatment. In general, the rash commonly manifested as a maculopapular type with or without pruritus.

Most of the observed Grade 3 rashes occurred approximately 1-3 weeks after the first dose of ipatasertib. In most of the cases with Grade 3 rash requiring dose interruption, ipatasertib dosing was resumed with dose reduction without recurrence of rash. Rash and other dermatologic events should be closely monitored and managed per standard of care. In general, for severe rash, dosing of ipatasertib should be held and patients should be treated with supportive therapy per standard of care; use of topical antihistamines as well as topical or systemic corticosteroids should be considered.

Dosage modification guidelines for skin toxicity attributable to the ipatasertib are outlined below:

- Grade 1 or Grade 2 skin toxicity, asymptomatic: Investigator may prescribe topical or oral steroids or per institutional guidelines.
- Grade 2 skin toxicity, symptomatic: Investigator should consider topical or oral steroids per institutional guidelines. The ipatasertib treatment may be held until resolution to Grade ≤ 1 or resolution such that the skin toxicity is no longer clinically significant.
- Grade ≥ 3 skin toxicity: The ipatasertib or should be held for a maximum of 4 weeks (approximately 28 days). If the skin toxicity resolves to Grade ≤ 2 or resolution such that the skin toxicity is no longer clinically significant in less than 4 weeks, dosing of the ipatasertib may resume, but the dose of ipatasertib should be reduced by one dose level (see Table 1). If recovery of the skin toxicity to Grade ≤ 2 or resolution such that the skin toxicity is no longer clinically significant does not occur within a maximum of 4 weeks, the patient will permanently discontinue the ipatasertib. Dose re-escalation of the ipatasertib may be permitted in subsequent cycles for patients who exhibit Grade ≤ 1 skin toxicity for at least one cycle. The ipatasertib should be permanently discontinued for rash due to Stevens-Johnson syndrome, toxic epidermal necrolysis, or other suspected severe hypersensitivity or allergic reaction that is related to ipatasertib.

Drug-Drug Interactions

A clinical study (midazolam interaction cohort in Study PAM4743g) demonstrated that ipatasertib is a moderate inhibitor of CYP3A. Thus, sensitive substrates of CYP3A with a narrow therapeutic window should be avoided. In addition, in vivo DDI data with enzalutamide (a potent CYP3A4 inducer) and itraconazole (a strong CYP3A4 inducer) markedly decreased and increased plasma exposure of ipatasertib, respectively. This indicates that CYP3A4 is the main enzyme that metabolizes ipatasertib, and therefore strong CYP3A inhibitors and inducers should be avoided.

10.4 Special Populations

Pregnancy

Refer to appendix 1.

Nursing Mothers

It is not known whether ipatasertib is excreted in human milk. Because many drugs are excreted in human milk and because of the potential for serious adverse drug reactions in nursing infants, ipatasertib should not be administered to nursing mothers.

Geriatric Patients

Although many patients enrolled in the clinical studies were □□65 years of age, the safety of ipatasertib has not been tested specifically in geriatric populations.

10.5 Concomitant use with other medications

Ipatasertib is expected to be mild to moderate inhibitor of CYP3A in vivo. A clinical study in patients showed that ipatasertib at a dose of 600 mg resulted in a 2.22-fold increase in midazolam (sensitive CYP3A substrate) exposures. Ipatasertib is primarily metabolized by CYP3A, and hence, strong inhibitors and inducers of CYP3A may increase or decrease ipatasertib exposures, respectively. Therefore, the following drugs should be avoided, when administering ipatasertib. If the use of one of these drugs is necessary, a risk/benefit assessment should be made prior to its concomitant use with ipatasertib:

- Strong CYP3A inhibitors: such as, but not limited to, atazanavir, clarithromycin, indinavir, itraconazole, ketoconazole, nefazodone, nelfinavir, ritonavir, saquinavir, telithromycin, troleandamycin, voriconazole, and/or grapefruit juice or grapefruit supplements
- Strong CYP3A inducers: such as, but not limited to, rifampin, carbamazepine, rifapentine, phenytoin, phenobarbital, and/or St. John’s wort or hyperforin
- CYP3A4 substrates with a narrow therapeutic index: such as, but not limited to, alfentanil, astemizole, terfenadine, cisapride, cyclosporine, fentanyl, pimozide, quinidine, sirolimus, tacrolimus, ergot alkaloids ergotamine, and/or dihydroergotamine

10.6 Overdose

In Study PAM4743g (single-agent, dose-escalation study), the MTD was exceeded at 800 mg with 2 DLTs of Grade 3 asthenia and Grade 3 nausea.
Cases of overdosing should be managed in accordance with best medical practice.

11 ENTRECTINIB (Rodslytrek)

Investigators should also consult the local prescribing information if the IMP is approved in your country.

11.1 Standard dose

The following dosing recommendations are proposed based on consideration of clinical pharmacology together with clinical efficacy and safety data:
A 600 mg daily dose with or without food is recommended for all adults patients with ROS1-positive . There is no requirement to alter dose according to age, sex, race, or bodyweight.

11.2 Contraindications

Entrectinib is contraindicated in patients with a known hypersensitivity to entrectinib or any of the excipients.

11.3 Warnings and Precautions

The evaluation of safety described in this section of the guidance for the Investigators is derived from an integrated analysis of 504 patients across four clinical studies (STARTKR-1, ALKA, STARTK-2, and STARTRK-NG) with a data cutoff date of 31 October 2018.

Dosage Modifications

Dosage modifications for adverse reactions:

- First dose reduction: 400 mg qDay
- Second dose reduction: 200 mg qDay
- Permanently discontinue if toxicities persist or recur following 2 dose reductions

Congestive Heart Failure

Congestive heart failure (CHF) has been reported across clinical trials with entrectinib. These reactions were observed in patients with or without a history of cardiac disease and resolved upon treatment with diuretics and/or dose reduction/interruption.
For patients with symptoms or known risk factors of CHF, left ventricular ejection fraction should be assessed prior to initiation of entrectinib treatment. Patients receiving entrectinib should be carefully monitored for signs and symptoms of CHF, including shortness of breath or edema, and those with clinical signs and symptoms of CHF should be evaluated and treated as clinically appropriate.

- Grade 2 or 3: Withhold until recovered to Grade ≤1; resume at reduced dose
- Grade 4: Permanently discontinue

QTc Interval Prolongation

QT interval prolongation has been observed in patients treated with entrectinib in clinical trials.
Use of entrectinib should be avoided in patients with congenital long QT syndrome and in patients taking medications that are known to prolong QT interval. Assessment of ECG at baseline and periodic monitoring of ECGs and electrolytes are recommended.

- Withhold until QTc interval recovers to baseline
- Resume at same dose if causes of QT prolongation are identified and corrected
- Resume at reduced dose if other causes of QT prolongation are not identified

Cognitive Disorders

Cognitive disorders, including confusion, mental status changes, memory impairment, and hallucinations, were reported in clinical trials with entrectinib. Patients should be monitored for signs of cognitive changes. Patients should be counseled on the potential for cognitive changes with entrectinib treatment. Patients should be instructed not to drive or use machines until symptoms resolve if they experience symptoms of cognitive disorders.

- Intolerable Grade 2: Withhold until recovered to Grade ≤1; resume at reduced dose, as clinically appropriate
- Grade 3: Withhold until recovered to Grade ≤1; resume at reduced dose
- Grade 4: Permanently discontinue

Hepatotoxicity

- Grade 3 Withhold until recovered to Grade ≤1; resume at reduced dose; If resolution occurs within 4 weeks, resume at same dose If adverse reaction persists after 4 weeks, permanently discontinue For recurrent Grade 3 events that resolve within 4 weeks, resume at a reduced dose.
- Grade 4 Withhold until recovered to Grade ≤1; resume at reduce dose; If adverse reaction does not resolve within 4 weeks or Grade 4 events recurs, permanently discontinue.

Elevated ALT or AST

ALT or AST >3x ULN with concurrent total bilirubin >1.5x ULN (in the absence of cholestasis or hemolysis): Permanently discontinue.

Fractures

Entrectinib use has been associated with an increased risk of fractures. Patients with signs or symptoms (e.g., pain, changes in mobility, deformity) of fractures should be evaluated promptly. In adult patients, some fractures occurred in the setting of a fall or other trauma to the affected area, while in pediatric patients fractures occurred in patients with minimal or no trauma. There are no data on the effects of entrectinib on healing of known fractures and the risk of future fractures. In the majority of pediatric patients treatment was continued with entrectinib and the fracture healed.

Vision disorders

Grade ≥2: Withhold until improvement or stabilization; resume at same dose or reduced dose, as clinically appropriate.

Anemia or neutropenia

Grade 3 or 4: Withhold until recovery to Grade≤2; resume at same or reduced dose, as clinically appropriate.

Other clinically relevant adverse reactions

Grade 3 or 4: Withhold until adverse reaction resolves to Grade 1 or baseline
Resume at same or reduced dose if resolved within 4 weeks. Permanently discontinue if adverse reaction does not resolve within 4 weeks or Grade 4 events recurs.

11.4 Special Populations

No formal PK study has been conducted in renally impaired patients. However, since entrectinib elimination via the kidney is negligible, no dose adjustment is required in patients with renal impairment.
Limited clinical data is available in patients with hepatic impairment and a dedicated PK study in patients with hepatic impairment has not yet been conducted. No specific guidance is given for hepatic impairment patients.

Reproductive and Developmental Toxicity, Pregnancy

Refer to appendix 1.

Nursing Mothers

It is not known whether entrectinib or its metabolites are excreted in human breast milk. No studies have been conducted to assess the effects of entrectinib on milk production or its presence in breast milk. As the potential for harm to the nursing infant is unknown, mothers should be advised to discontinue breast-feeding during treatment with entrectinib. Nursing mothers are currently excluded from entrectinib studies.

Geriatric Patients

No differences in safety or efficacy were observed between patients ≥65 years of age and younger patients. Ongoing clinical studies may include patients>65 years old.

11.5 Concomitant use with other medications

Drug Interactions

- No dose adjustments are required when entrectinib is co-administered with PPIs or other drugs that raise gastric pH (e.g., H_2 receptor antagonists or antacids).
- In adult a 100 mg dose of entrectinib is recommended when a strong CYP3A4 inhibitor is co-administered. A 200 mg dose of entrectinib is recommended when co-administered with a moderate CYP3A4 inhibitor. The concomitant use of strong or moderate CYP3A inhibitors in pediatric patients should be avoided.

- No dose adjustment is recommended when entrectinib is administered with a weak CYP3A4 inhibitor.
- Avoid concomitant use of entrectinib with CYP3A inducers.
- No dose adjustment is required when entrectinib is co-administered with CYP3A substrates.
- No dose adjustment of entrectinib is required when entrectinib is co-administered with digoxin or P-gp substrates.
- As with P-gp, a mild inhibition of BCRP was observed in in vitro studies. Given that no clinically significant interaction was observed with the P-gp substrate digoxin an interaction with BCRP is not predicted. No dose adjustment required when entrectinib is co-administered with BCRP substrates.

CYP3A inhibitors
Because of this potential, entrectinib should be administered with caution with the drugs listed in Table 1.
Adult patients
The concomitant use of strong or moderate CYP3A inhibitors and entrectinib in adults should be avoided or limited to 14 days or less. If concomitant use of strong or moderate CYP3A inhibitors cannot be avoided, entrectinib dose should be reduced to 100 mg once daily for use with strong CYP3A inhibitors and to 200 mg once daily for use with moderate CYP3A inhibitors.
After discontinuation of the concomitant strong or moderate CYP3A inhibitors, entrectinib dose that was taken prior to initiating the strong or moderate CYP3A inhibitor can be resumed. A wash out period may be required for CYP3A4 inhibitors with long half-life
Pediatric patients
The concomitant use of strong or moderate CYP3A inhibitors in pediatric patients should be avoided.
Concomitant CYP3A inducers
Co-administration of Entrectinib with CYP3A inducers in adult and pediatric patients should be avoided.

Table 1. Cytochrome P450 CYP3A4 Inhibitors and Inducers

Inhibitors	Inducers
Strong: Boceprevir, clarithromycin, conivaptan, grapefruit juice, indinavir, itraconazole, ketoconazole, lopinavir/ritonavir, mibefradil, nefazodone, nelfinavir, posaconazole, ritonavir, saquinavir, telaprevir, telithromycin, voriconazole<br><br>Moderate: Aprepitant, cimetidine, ciprofloxacin, clotrimazole, crizotinib, cyclosporine, dronedarone, erythromycin, fluconazole, fluvoxamine, imatinib, tofisopam, verapamil	Strong: Alfentanil, cyclosporine, dihydroergotamine, ergotamine, fentanyl, pimozide, quinidine, sirolimus, tacrolimus, rifampin, St. John’s Wort<br><br>Moderate: Bosentan, efavirenz, etravirine, modafinil

11.6 Overdose

There are no known antidotes for entrectinib overdose. In the case of an overdose, major toxicities (e.g., bone marrow, liver toxicity, or clinical signs of CNS toxicity) or other unexpected toxicities might occur. The investigator must monitor for both acute and long-term effects.

12 AFINITOR (Everolimus)

Investigators should also consult the local prescribing information if the IMP is approved in your country.

12.1 Standard dose

The recommended dose is 10 mg everolimus once daily. Treatment should continue as long as clinical benefit is observed or until unacceptable toxicity occurs. If a dose is missed, the patient should not take an additional dose, but take the next prescribed dose as usual. Afinitor should be administered orally once daily at the same time every day, consistently either with or without food. Afinitor tablets should be swallowed whole with a glass of water. The tablets should not be chewed or crushed.

12.2 Contraindications

Hypersensitivity to the active substance, to other rapamycin derivatives or to any of the excipients listed: Butylhydroxytoluene, Magnesium stearate, Lactose monohydrate, Hypromellose, Crospovidone type A, Lactose anhydrous.

12.3 Warnings and Precautions

Non-infectious pneumonitis

Non-infectious pneumonitis is a class effect of rapamycin derivatives, including everolimus. Non-infectious pneumonitis (including interstitial lung disease) has been frequently reported in patients taking Afinitor. Some cases were severe and on rare occasions, a fatal outcome was observed. A diagnosis of non-infectious pneumonitis should be considered in patients presenting with non-specific respiratory signs and symptoms such as hypoxia, pleural effusion, cough or dyspnoea, and in whom infectious, neoplastic and other non-medicinal causes have been excluded by means of appropriate investigations. Opportunistic infections such as pneumocystis jirovecii (carinii) pneumonia (PJP/PCP) should be ruled out in the differential diagnosis of non-infectious pneumonitis. Patients should be advised to report promptly any new or worsening respiratory symptoms. Patients who develop radiological changes suggestive of non-infectious pneumonitis and have few or no symptoms may continue Afinitor therapy without dose adjustments. If symptoms are moderate (Grade 2) or severe (Grade 3) the use of corticosteroids may be indicated until clinical symptoms resolve. For patients who require use of corticosteroids for treatment of non-infectious pneumonitis, prophylaxis for PJP/PCP may be considered.

Infections

Everolimus has immunosuppressive properties and may predispose patients to bacterial, fungal, viral or protozoan infections, including infections with opportunistic pathogens. Localised and systemic infections, including pneumonia, other bacterial infections, invasive fungal infections such as aspergillosis, candidiasis or PJP/PCP and viral infections including reactivation of hepatitis B virus, have been described in patients taking Afinitor. Some of these infections have been severe (e.g. leading to sepsis, respiratory or hepatic failure) and occasionally fatal. Physicians and patients should be aware of the increased risk of infection with Afinitor. Pre-existing infections should be treated appropriately and should have resolved fully before starting treatment with Afinitor. While taking Afinitor, be vigilant for symptoms and signs of infection; if a diagnosis of infection is made, institute appropriate treatment promptly and consider interruption or discontinuation of Afinitor. If a diagnosis of invasive systemic fungal infection is made, the Afinitor treatment should be promptly and permanently discontinued and the patient treated with appropriate antifungal therapy. Cases of PJP/PCP, some with fatal outcome, have been reported in patients who received everolimus. PJP/PCP may be associated with concomitant use of corticosteroids or other immunosuppressive agents. Prophylaxis for PJP/PCP should be considered when concomitant use of corticosteroids or other immunosuppressive agents are required.

Hypersensitivity reactions

Hypersensitivity reactions manifested by symptoms including, but not limited to, anaphylaxis, dyspnoea, flushing, chest pain or angioedema (e.g. swelling of the airways or tongue, with or without respiratory impairment) have been observed with everolimus.

Concomitant use of angiotensin-converting enzyme (ACE) inhibitors
Patients taking concomitant ACE inhibitor (e.g. ramipril) therapy may be at increased risk for angioedema (e.g. swelling of the airways or tongue, with or without respiratory impairment).

Stomatitis
Stomatitis, including mouth ulcerations and oral mucositis, is the most commonly reported adverse reaction in patients treated with Afinitor. Stomatitis mostly occurs within the first 8 weeks of treatment. A single-arm study in postmenopausal breast cancer patients treated with Afinitor plus exemestane suggested that an alcohol-free corticosteroid oral solution, administered as a mouthwash during the initial 8 weeks of treatment, may decrease the incidence and severity of stomatitis. Management of stomatitis may therefore include prophylactic and/or therapeutic use of topical treatments, such as an alcohol-free corticosteroid oral solution as a mouthwash. However products containing alcohol, hydrogen peroxide, iodine and thyme derivatives should be avoided as they may exacerbate the condition. Monitoring for and treatment of fungal infection is recommended, especially in patients being treated with steroid-based medicinal products. Antifungal agents should not be used unless fungal infection has been diagnosed.

Renal failure events
Cases of renal failure (including acute renal failure), some with a fatal outcome, have been observed in patients treated with Afinitor. Renal function should be monitored particularly where patients have additional risk factors that may further impair renal function.

Laboratory tests and monitoring

- Renal function: Elevations of serum creatinine, usually mild, and proteinuria have been reported. Monitoring of renal function, including measurement of blood urea nitrogen (BUN), urinary protein or serum creatinine, is recommended prior to the start of Afinitor therapy and periodically thereafter.
- Blood glucose: Hyperglycaemia has been reported. Monitoring of fasting serum glucose is recommended prior to the start of Afinitor therapy and periodically thereafter. More frequent monitoring is recommended when Afinitor is co-administered with other medicinal products that may induce hyperglycaemia. When possible optimal glycaemic control should be achieved before starting a patient on Afinitor.
- Blood lipids: Dyslipidaemia (including hypercholesterolaemia and hypertriglyceridaemia) has been reported. Monitoring of blood cholesterol and triglycerides prior to the start of Afinitor therapy and periodically thereafter, as well as management with appropriate medical therapy, is recommended.
- Haematological parameters: Decreased haemoglobin, lymphocytes, neutrophils and platelets have been reported. Monitoring of complete blood count is recommended prior to the start of Afinitor therapy and periodically thereafter.

Wound healing complications
Impaired wound healing is a class effect of rapamycin derivatives, including everolimus. Caution should therefore be exercised with the use of Afinitor in the peri-surgical period.

Table 1 Afinitor dose adjustment recommendations

Adverse reaction	Severity[1]	Afinitor dose adjustment
Non-infectious pneumonitis	Grade 2	Consider interruption of therapy until symptoms improve to Grade 1.<br>Re-initiate treatment at 5 mg daily.<br>Discontinue treatment if failure to recover within 4 weeks.
	Grade 3	Interrupt treatment until symptoms resolve to Grade 1.<br>Consider re-initiating treatment at 5 mg daily. If toxicity recurs at Grade 3, consider discontinuation.
	Grade 4	Discontinue treatment.
Stomatitis	Grade 2	Temporary dose interruption until recovery to Grade 1.

		Re-initiate treatment at same dose.<br>If stomatitis recurs at Grade 2, interrupt dose until recovery to Grade 1. Re-initiate treatment at 5 mg daily.
	Grade 3	Temporary dose interruption until recovery to Grade 1.<br>Re-initiate treatment at 5 mg daily.
	Grade 4	Discontinue treatment.
Other non-haematological toxicities (excluding metabolic events)	Grade 2	If toxicity is tolerable, no dose adjustment required.<br>If toxicity becomes intolerable, temporary dose interruption until recovery to Grade 1. Re-initiate treatment at same dose.<br>If toxicity recurs at Grade 2, interrupt treatment until recovery to Grade 1. Re-initiate treatment at 5 mg daily.
	Grade 3	Temporary dose interruption until recovery to Grade 1.<br>Consider re-initiating treatment at 5 mg daily. If toxicity recurs at Grade 3, consider discontinuation.
	Grade 4	Discontinue treatment.
Metabolic events (e.g. hyperglycaemia, dyslipidaemia)	Grade 2	No dose adjustment required.
	Grade 3	Temporary dose interruption.<br>Re-initiate treatment at 5 mg daily.
	Grade 4	Discontinue treatment.
Thrombocytopenia	Grade 2<br>(<75, ≥50x10^9/l)	Temporary dose interruption until recovery to Grade 1 (≥75x10^9/l). Re-initiate treatment at same dose.
	Grade 3 & 4<br>(<50x10^9/l)	Temporary dose interruption until recovery to Grade 1 (≥75x10^9/l). Re-initiate treatment at 5 mg daily.
Neutropenia	Grade 2<br>(≥1x10^9/l)	No dose adjustment required.
	Grade 3<br>(<1, ≥0.5x10^9/l)	Temporary dose interruption until recovery to Grade 2 (≥1x10^9/l). Re-initiate treatment at same dose.
	Grade 4<br>(<0.5x10^9/l)	Temporary dose interruption until recovery to Grade 2 (≥1x10^9/l). Re-initiate treatment at 5 mg daily.
Febrile neutropenia	Grade 3	Temporary dose interruption until recovery to Grade 2 (≥1.25x10^9/l) and no fever.<br>Re-initiate treatment at 5 mg daily.
	Grade 4	Discontinue treatment.

1
Grading based on National Cancer Institute (NCI) Common Terminology Criteria for Adverse

12.4 Special Populations

Elderly patients (≥65 years)

No dose adjustment is required.

Renal impairment

No dose adjustment is required.

Hepatic impairment

Mild hepatic impairment (Child-Pugh A) – the recommended dose is 7.5 mg daily.

Moderate hepatic impairment (Child-Pugh B) – the recommended dose is 5 mg daily. Severe hepatic impairment (Child-Pugh C) – Afinitor is only recommended if the desired benefit outweighs the risk. In this

case, a dose of 2.5 mg daily must not be exceeded. Dose adjustments should be made if a patient's hepatic (Child-Pugh) status changes during treatment.

Reproductive and Developmental Toxicity, Pregnancy

Refer to appendix 1.

12.5 Concomitant use with other medications

Everolimus is a substrate of CYP3A4, and also a substrate and moderate inhibitor of PgP. Therefore, absorption and subsequent elimination of everolimus may be influenced by products that affect CYP3A4 and/or PgP. In vitro, everolimus is a competitive inhibitor of CYP3A4 and a mixed inhibitor of CYP2D6. Known and theoretical interactions with selected inhibitors and inducers of CYP3A4 and PgP are listed in Table 2 below.

CYP3A4 and PgP inhibitors increasing everolimus concentrations. Substances that are inhibitors of CYP3A4 or PgP may increase everolimus blood concentrations by decreasing metabolism or the efflux of everolimus from intestinal cells.

CYP3A4 and PgP inducers decreasing everolimus concentrations.

Substances that are inducers of CYP3A4 or PgP may decrease everolimus blood concentrations by increasing metabolism or the efflux of everolimus from intestinal cells.

Table 2 Effects of other active substances on everolimus

Active substance by interaction	Interaction – Change in Everolimus AUC/Cmax Geometric mean ratio (observed range)	Recommendations concerning co-administration
Potent CYP3A4/PgP inhibitors		
**Ketoconazole**	AUC ↑15.3-fold (range 11.2-22.5) Cmax ↑4.1-fold (range 2.6-7.0)	Concomitant treatment of Afinitor and potent inhibitors is not recommended.
**Itraconazole, posaconazole, voriconazole**	Not studied. Large increase in everolimus concentration is expected.	
**Telithromycin, clarithromycin**		
**Nefazodone**		
**Ritonavir, atazanavir, saquinavir, darunavir, indinavir, nelfinavir**		
Moderate CYP3A4/PgP inhibitors		
**Erythromycin**	AUC ↑4.4-fold (range 2.0-12.6) Cmax ↑2.0-fold (range 0.9-3.5)	Use caution when co-administration of moderate CYP3A4 inhibitors or PgP inhibitors cannot be avoided. If patients require co-administration of a moderate CYP3A4 or PgP inhibitor, dose reduction to 5 mg daily or 2.5 mg daily may be considered. However, there are no
**Imatinib**	AUC ↑ 3.7-fold Cmax ↑ 2.2-fold	
**Verapamil**	AUC ↑3.5-fold (range 2.2-6.3) Cmax ↑2.3-fold	

	(range1.3-3.8)	clinical data with this dose
**Ciclosporin oral**	AUC ↑2.7-fold<br>(range 1.5-4.7)<br>Cmax ↑1.8-fold<br>(range 1.3-2.6)	adjustment. Due to between subject variability the recommended dose adjustments may not be optimal in all
**Fluconazole**	Not studied. Increased exposure	individuals, therefore close
**Diltiazem**	expected.	monitoring of side effects is
**Dronedarone**	Not studied. Increased exposure expected.	recommended. If the moderate inhibitor is discontinued, consider
**Amprenavir, fosamprenavir**	Not studied. Increased exposure expected.	a washout period of at least 2 to 3 days (average elimination time for most commonly used moderate inhibitors) before the Afinitor dose is returned to the dose used prior to initiation of the co-administration.
**Grapefruit juice or other food affecting CYP3A4/PgP**	Not studied. Increased exposure expected (the effect varies widely).	Combination should be avoided.

**Active substance by interaction**	**Interaction – Change in Everolimus AUC/Cmax Geometric mean ratio (observed range)**	**Recommendations concerning co-administration**
***Potent and moderate* CYP3A4 inducers**		
**Rifampicin**	AUC ↓63%<br>(range 0-80%)<br>Cmax ↓58%<br>(range 10-70%)	Avoid the use of concomitant potent CYP3A4 inducers. If patients require co-administration of a potent CYP3A4 inducer, an Afinitor dose increase from 10 mg daily up to 20 mg daily should be considered using 5 mg increments or less applied on Day 4 and 8 following start of the inducer. This dose of Afinitor is predicted to adjust the AUC to the range observed without inducers. However, there are no clinical data with this dose adjustment. If treatment with the inducer is discontinued, consider a washout period of at least 3 to 5 days (reasonable time for significant
**Dexamethasone**	Not studied. Decreased exposure expected.	
**Carbamazepine, phenobarbital, phenytoin**	Not studied. Decreased exposure expected.	
**Efavirenz, nevirapine**	Not studied. Decreased exposure expected.	

		enzyme de-induction), before the Afinitor dose is returned to the dose used prior to initiation of the co-administration.
**St John's Wort (*Hypericum perforatum*)**	Not studied. Large decrease in exposure expected.	Preparations containing St John's Wort should not be used during treatment with everolimus

12.6 Overdose

Reported experience with overdose in humans is very limited. Single doses of up to 70 mg have been given with acceptable acute tolerability. General supportive measures should be initiated in all cases of overdose.

13 IBRANCE (palbociclib)

Investigators should also consult the local prescribing information if the IMP is approved in your country.

13.1 Standard dose

The recommended dose is 125 mg of palbociclib once daily for 21 consecutive days followed by 7 days off treatment (Schedule 3/1) to comprise a complete cycle of 28 days.
The treatment with IBRANCE should be continued as long as the patient is deriving clinical benefit from therapy or until unacceptable toxicity occurs. Patients should be encouraged to take their dose at approximately the same time each day. If the patient vomits or misses a dose, an additional dose should not be taken that day. The next prescribed dose should be taken at the usual time. IBRANCE is for oral use. It should be taken with food, preferably a meal to ensure consistent palbociclib exposure. Palbociclib should not be taken with grapefruit or grapefruit juice. IBRANCE capsules should be swallowed whole (should not be chewed, crushed, or opened prior to swallowing). No capsule should be ingested if it is broken, cracked, or otherwise not intact.

13.2 Contraindications

Hypersensitivity to the active substance or to any of the excipients listed: Microcrystalline cellulose, Colloidal silicon dioxide, Crospovidone, Magnesium stearate, Succinic acid, Hypromellose (E464), Titanium dioxide (E171), Triacetin, Indigo carmine aluminum lake (E132), Iron oxide red (E172) (75 mg and 125 mg tablets only), Iron oxide yellow (E172) (100 mg tablets only).
Use of preparations containing St. John’s Wort.

13.3 Warnings and Precautions

Dose modification of IBRANCE is recommended based on individual safety and tolerability. Management of some adverse reactions may require temporary dose interruptions/delays, and/or dose reductions, or permanent discontinuation as per dose reduction schedules.

Table 1: **IBRANCE recommended dose modifications for adverse reactions**

Dose level	Dose
Recommended dose	125 mg/day
First dose reduction	100 mg/day
Second dose reduction	75 mg/day*

<u>**Haematological disorders**</u>

Dose interruption, dose reduction, or delay in starting treatment cycles is recommended for patients who develop Grade 3 or 4 neutropenia. Appropriate monitoring should be performed. Complete blood count should be monitored prior to the start of IBRANCE therapy and at the beginning of each cycle, as well as on Day 15 of the first 2 cycles, and as clinically indicated. For patients who experience a maximum of Grade 1 or 2 neutropenia in the first 6 cycles, complete blood counts for subsequent cycles should be monitored every 3 months, prior to the beginning of a cycle and as clinically indicated. Absolute neutrophil counts (ANC) of ≥ 1,000/mm3 and platelet counts of ≥ 50,000/mm3 are recommended to receive IBRANCE.

Table 2: IBRANCE dose modification and management – Haematological toxicities

CTCAE grade	Dose modifications
Grade 1 or 2	No dose adjustment is required.
Grade 3[a]	Day 1 of cycle:<br>[redacted]<br>Withhold IBRANCE, until recovery to Grade ≤ 2, and repeat complete blood count monitoring within 1 week. When recovered to Grade ≤ 2, start the next cycle at the *same dose*.

	Day 15 of first 2 cycles:<br>If Grade 3 on Day 15, continue IBRANCE at the *current dose* to complete cycle and repeat complete blood count on Day 22.<br>If Grade 4 on Day 22, see Grade 4 dose modification guidelines below.<br><br>Consider dose reduction in cases of prolonged (> 1 week) recovery from Grade 3 neutropenia or recurrent Grade 3 neutropenia on Day 1 of subsequent cycles.
Grade 3 ANCb<br>(< 1,000 to 500/mm^3)<br>+ Fever ≥ 38.5 ºC<br>and/or infection	At any time:<br>Withhold IBRANCE until recovery to Grade ≤ 2<br>Resume at next lower dose.
Grade 4[a]	At any time:<br>Withhold IBRANCE until recovery to Grade ≤ 2.<br>Resume at next lower dose.

Grading according to CTCAE 4.0.
ANC=absolute neutrophil counts; CTCAE=Common Terminology Criteria for Adverse Events; LLN=lower limit of normal.
a Table applies to all haematological adverse reactions except lymphopenia (unless associated with clinical events, e.g., opportunistic infections).
b ANC: Grade 1: ANC < LLN – 1,500/mm3; Grade 2: ANC 1,000 - < 1,500/mm3; Grade 3: ANC 500 - < 1,000/mm3; Grade 4: ANC < 500/mm3.

Critical visceral disease
The efficacy and safety of palbociclib have not been studied in patients with critical visceral disease.

Interstitial lung disease/pneumonitis
Severe, life-threatening, or fatal ILD and/or pneumonitis can occur in patients treated with IBRANCE when taken in combination with endocrine therapy. Across clinical trials (PALOMA-1, PALOMA-2, PALOMA-3), 1.4% of IBRANCE-treated patients had ILD/pneumonitis of any grade, 0.1% had Grade 3, and no Grade 4 or fatal cases were reported. Additional cases of ILD/pneumonitis have been observed in the post-marketing setting, with fatalities reported. Monitor patients for pulmonary symptoms indicative of ILD/pneumonitis (e.g. hypoxia, cough, dyspnoea). In patients who have new or worsening respiratory symptoms and are suspected to have developed ILD/pneumonitis, interrupt IBRANCE immediately and evaluate the patient. Permanently discontinue IBRANCE in patients with severe ILD or pneumonitis.

Infections
Since IBRANCE has myelosuppressive properties, it may predispose patients to infections. Infections have been reported at a higher rate in patients treated with IBRANCE in randomised clinical studies compared to patients treated in the respective comparator arm. Grade 3 and Grade 4 infections occurred respectively in 4.5% and 0.7% of patients treated with IBRANCE in any combination. Patients should be monitored for signs and symptoms of infection and treated as medically appropriate. Physicians should inform patients to promptly report any episodes of fever.

Hepatic impairment
Administer IBRANCE with caution to patients with moderate or severe hepatic impairment, with close monitoring of signs of toxicity.

Renal impairment

Administer IBRANCE with caution to patients with moderate or severe renal impairment, with close monitoring of signs of toxicity

.

Lactose

This medicinal product contains lactose. Patients with rare hereditary problems of galactose intolerance, the Lapp lactase deficiency, or glucose-galactose malabsorption should not take this medicine.

General adverse events

The overall safety profile of IBRANCE is based on pooled data from 872 patients who received palbociclib in combination with endocrine therapy (N=527 in combination with letrozole and N=345 in combination with fulvestrant) in randomised clinical studies in HR-positive, HER2-negative advanced or metastatic breast cancer. The most common (≥ 20%) adverse reactions of any grade reported in patients receiving palbociclib in randomised clinical studies were neutropenia, infections, leukopenia, fatigue, nausea, stomatitis, anaemia, diarrhoea, alopecia and thrombocytopenia. The most common (≥ 2%) Grade ≥ 3 adverse reactions of palbociclib were neutropenia, leukopenia, infections, anaemia, aspartate aminotransferase (AST) increased, fatigue, and alanine aminotransferase (ALT) increased. Dose reductions or dose modifications due to any adverse reaction occurred in 38.4% of patients receiving IBRANCE in randomised clinical studies regardless of the combination. Permanent discontinuation due to an adverse reaction occurred in 5.2% of patients receiving IBRANCE in randomised clinical studies regardless of the combination.

Table 3: IBRANCE dose modification and management – Non-haematological toxicities

CTCAE grade	Dose modifications
Grade 1 or 2	No dose adjustment is required.
Grade ≥ 3 non-haematological toxicity (if persisting despite medical treatment)	Withhold until symptoms resolve to:<br>Grade ≤ 1;<br>Grade ≤ 2 (if not considered a safety risk for the patient)<br>Resume at the next lower dose.

CTCAE=Common Terminology Criteria for Adverse Events. Grading according to CTCAE 4.0.

13.4 Special Populations

Elderly

No dose adjustment of IBRANCE is necessary in patients ≥ 65 years of age.

Hepatic impairment

No dose adjustment of IBRANCE is required for patients with mild or moderate hepatic impairment (Child-Pugh classes A and B). For patients with severe hepatic impairment (Child-Pugh class C), the recommended dose of IBRANCE is 75 mg once daily on Schedule 3/1.

Renal impairment

No dose adjustment of IBRANCE is required for patients with mild, moderate or severe renal impairment (creatinine clearance [CrCl] ≥ 15 mL/min). Insufficient data are available in patients requiring haemodialysis to provide any dose adjustment recommendation in this patient population.

Reproductive Toxicity, Pregnancy

Refer to appendix 1.

13.5 Concomitant use with other medications

Palbociclib is primarily metabolised by CYP3A and sulphotransferase (SULT) enzyme SULT2A1. In vivo, palbociclib is a weak, time-dependent inhibitor of CYP3A.

Effects of other medicinal products on the pharmacokinetics of palbociclib Effect of CYP3A inhibitors: Coadministration of multiple 200 mg doses of itraconazole with a single 125 mg palbociclib dose increased palbociclib total exposure (AUCinf) and the peak concentration (Cmax) by approximately 87% and 34%, respectively, relative to a single 125 mg palbociclib dose given alone. The concomitant use of strong CYP3A inhibitors including, but not limited to: clarithromycin, indinavir, itraconazole, ketoconazole,

lopinavir/ritonavir, nefazodone, nelfinavir, posaconazole, saquinavir, telaprevir, telithromycin, voriconazole, and grapefruit or grapefruit juice, should be avoided. No dose adjustments are needed for mild and moderate CYP3A inhibitors.
Effect of CYP3A inducers: Coadministration of multiple 600 mg doses of rifampin with a single 125 mg palbociclib dose decreased palbociclib AUCinf and Cmax by 85% and 70%, respectively, relative to a single 125 mg palbociclib dose given alone. The concomitant use of strong CYP3A inducers including, but not limited to: carbamazepine, enzalutamide, phenytoin, rifampin, and St. John's Wort should be avoided. Coadministration of multiple 400 mg daily doses of modafinil, a moderate CYP3A inducer, with a single 125 mg IBRANCE dose decreased palbociclib AUCinf and Cmax by 32% and 11%, respectively, relative to a single 125 mg IBRANCE dose given alone. No dose adjustments are required for moderate CYP3A inducers.
Effect of acid reducing agents: Under fed conditions (intake of a moderate-fat meal), coadministration of multiple doses of the proton pump inhibitor (PPI) rabeprazole with a single dose of 125 mg IBRANCE decreased palbociclib Cmax by 41%, but had limited impact on AUCinf (13% decrease) compared with a single dose of 125 mg IBRANCE administered alone. Under fasting conditions, the coadministration of multiple doses of the proton pump inhibitor (PPI) rabeprazole with a single dose of 125 mg IBRANCE decreased palbociclib AUCinf and Cmax by 62% and 80%, respectively. Therefore, IBRANCE should be taken with food, preferably a meal. Given the reduced effect on gastric pH of H2-receptor antagonists and local antacids compared to PPIs, no clinically relevant effect of H2-receptor antagonists or local antacids on palbociclib exposure is expected when palbociclib is taken with food.

Effects of palbociclib on the pharmacokinetics of other medicinal products

Palbociclib is a weak, time-dependent inhibitor of CYP3A following daily 125 mg dosing at steady state. Coadministration of multiple doses of palbociclib with midazolam increased the midazolam AUCinf and Cmax values by 61% and 37%, respectively, as compared with administration of midazolam alone. The dose of sensitive CYP3A substrates with a narrow therapeutic index (e.g., alfentanil, cyclosporine, dihydroergotamine, ergotamine, everolimus, fentanyl, pimozide, quinidine, sirolimus, and tacrolimus) may need to be reduced when coadministered with IBRANCE as IBRANCE may increase their exposure.

Effects on ability to drive and use machines

IBRANCE has minor influence on the ability to drive and use machines. However, IBRANCE may cause fatigue and patients should exercise caution when driving or using machines.

13.6 Overdose

In the event of a palbociclib overdose, both gastrointestinal (e.g., nausea, vomiting) and haematological (e.g., neutropenia) toxicity may occur and general supportive care should be provided.

14 TYVERB (lapatinib)

Investigators should also consult the local prescribing information if the IMP is approved in your country.

14.1 Standard dose

The recommended dose of Tyverb is 1250 mg (i.e. five tablets) once daily continuously (in association with capecitabine); 1000 mg (i.e. four tablets) once daily continuously (in association with trastuzumab); 1500 mg (i.e. six tablets) once daily continuously (in association with aromatase inhibitors).
Tyverb is for oral use. The daily dose of Tyverb should not be divided. Tyverb should be taken either at least one hour before, or at least one hour after food. To minimise variability in the individual patient, administration of Tyverb should be standardised in relation to food intake, for example always to be taken one hour before a meal. Missed doses should not be replaced and the dosing should resume with the next scheduled daily dose.

14.2 Contraindications

Hypersensitivity to the active substance or to any of the excipients. Microcrystalline cellulose, Povidone (K30), Sodium starch glycolate (Type A), Magnesium stearate, Hypromellose, Titanium dioxide (E171), Macrogol (400), Polysorbate 80, Iron oxide yellow (E172), Iron oxide red (E172)

14.3 Warnings and Precautions

Cardiac events

Tyverb should be discontinued in patients with symptoms associated with decreased left ventricular ejection fraction (LVEF) that are National Cancer Institute Common Terminology Criteria for Adverse Events (NCI CTCAE) grade 3 or greater or if their LVEF drops below the institutions lower limit of normal. Tyverb may be restarted at a reduced dose (750 mg/day when administered with trastuzumab, 1000 mg/day when administered with capecitabine or 1250 mg/day when administered with an aromatase inhibitor) after a minimum of 2 weeks and if the LVEF recovers to normal and the patient is asymptomatic. Lapatinib has not been evaluated in patients with symptomatic cardiac failure. Caution should be taken if Tyverb is to be administered to patients with conditions that could impair left ventricular function (including co-administration with potentially cardiotoxic medicinal products). Evaluation of cardiac function, including LVEF determination, should be conducted for all patients prior to initiation of treatment with Tyverb to ensure that the patient has a baseline LVEF that is within the institutions normal limits. LVEF should continue to be evaluated during treatment with Tyverb to ensure that LVEF does not decline to an unacceptable level. In some cases, LVEF decrease may be severe and lead to cardiac failure. Fatal cases have been reported, causality of the deaths is uncertain. In studies across the clinical development programme for lapatinib, cardiac events including LVEF decreases were reported in approximately 1% of patients. Symptomatic LVEF decreases were observed in approximately 0.3% of patients who received lapatinib. However, when lapatinib was administered in combination with trastuzumab in the metastatic setting, the incidence of cardiac events including LVEF decreases was higher (7%) versus the lapatinib alone arm (2%) in the pivotal trial. The cardiac events observed in this study were comparable in nature and severity to those previously seen with lapatinib. A concentration-dependent increase of the QTc interval was demonstrated in a dedicated placebo-controlled crossover study in subjects with advanced solid tumours. Caution should be taken if Tyverb is administered to patients with conditions that could result in prolongation of QTc (including hypokalemia, hypomagnesemia, and congenital long QT syndrome), co-administration of other medicinal product known to cause QT prolongation, or conditions that increase the exposure of lapatinib, such as co-administration of strong CYP3A4 inhibitors. Hypokalemia or hypomagnesemia should be corrected prior to treatment. Electrocardiograms with QT measurement should be performed prior to and one to two weeks after the start of Tyverb therapy. When clinically indicated, e.g. after initiation of a concomitant treatment that might affect QT or that may interact with lapatinib, ECG measurement should also be considered.

Interstitial lung disease / pneumonitis

Tyverb should be discontinued in patients who experience pulmonary symptoms which are NCI CTCAE grade 3 or greater. Pulmonary toxicity may be severe and lead to respiratory failure.

Diarrhoea

Tyverb dosing should be interrupted in patients with diarrhoea which is NCI CTCAE grade 3 or grade 1 or 2 with complicating features (moderate to severe abdominal cramping, nausea or vomiting greater than or equal to NCI CTCAE grade 2, decreased performance status, fever, sepsis, neutropenia, frank bleeding or dehydration). Tyverb may be reintroduced at a lower dose (reduced from 1000 mg/day to 750 mg/day, from 1250 mg/day to 1000 mg/day or from 1500 mg/day to 1250 mg/day) when diarrhoea resolves to grade 1 or less. Tyverb dosing should be permanently discontinued in patients with diarrhoea which is NCI CTCAE grade 4.

Hepatotoxicity

Hepatotoxicity has occurred with Tyverb use and may in rare cases be fatal. The hepatotoxicity may occur days to several months after initiation of treatment. At the initiation of treatment, patients should be advised of the potential for hepatotoxicity. Liver function (transaminases, bilirubin and alkaline phosphatase) should be monitored before the initiation of treatment and monthly thereafter, or as clinically indicated. Tyverb dosing should be discontinued if changes in liver function are severe and patients should not be retreated. Patients who carry the HLA alleles DQA1*02:01 and DRB1*07:01 have increased risk of Tyverb-associated hepatotoxicity. In a large, randomised clinical trial of Tyverb monotherapy (n=1,194), the cumulative frequency of severe liver injury (ALT >5 times the upper limit of normal, NCI CTCAE grade 3) at 1 year of treatment was 2.8% overall. The cumulative frequency in DQA1*02:01 and DRB1*07:01 allele carriers was 10.3% and in non-carriers was 0.5%. Carriage of the HLA risk alleles is common (15 to 25%) in Caucasian, Asian, African and Hispanic populations but lower (1%) in Japanese populations.

Serious cutaneous reactions

Serious cutaneous reactions have been reported with Tyverb. If erythema multiforme or life-threatening reactions such as Stevens-Johnson syndrome, or toxic epidermal necrolysis (e.g. progressive skin rash often with blisters or mucosal lesions) are suspected, discontinue treatment with Tyverb.

Other toxicities

Discontinuation or interruption of dosing with Tyverb may be considered when a patient develops toxicity greater than or equal to grade 2 on the NCI CTCAE. Dosing can be restarted, when the toxicity improves to grade 1 or less, at 1000 mg/day when administered with trastuzumab, 1250 mg/day when administered with capecitabine or 1500 mg/day when administered with an aromatase inhibitor. If the toxicity recurs, then Tyverb should be restarted at a lower dose (750 mg/day when administered with trastuzumab, 1000 mg/day when administered with capecitabine or 1250 mg/day when administered with an aromatase inhibitor).

14.4 Special Populations

Renal impairment

No dose adjustment is necessary in patients with mild to moderate renal impairment. Caution is advised in patients with severe renal impairment as there is no experience of Tyverb in this population.

Hepatic impairment

Tyverb should be discontinued if changes in liver function are severe and patients should not be retreated. Administration of Tyverb to patients with moderate to severe hepatic impairment should be undertaken with caution due to increased exposure to the medicinal product. Insufficient data are available in patients with hepatic impairment to provide a dose adjustment recommendation.

Elderly

There are limited data on the use of Tyverb / capecitabine and Tyverb / trastuzumab in patients aged ≥65 years. In the phase III clinical study of Tyverb in combination with letrozole, of the total number of hormone receptor positive metastatic breast cancer patients (Intent to treat population N= 642), 44 % were ≥65 years of age. No overall differences in efficacy and safety of the combination of Tyverb and letrozole were observed between these patients and patients < 65 years of age.

Reproductive Toxicity, Pregnancy

Refer to appendix 1.

14.5 Concomitant use with other medications

Concomitant treatment with inhibitors or inducers of CYP3A4
Concomitant treatment with inducers of CYP3A4 should be avoided due to risk of decreased exposure to lapatinib. Concomitant treatment with strong inhibitors of CYP3A4 should be avoided due to risk of increased exposure to lapatinib. Grapefruit juice should be avoided during treatment with Tyverb. Co-administration of Tyverb with orally administered medicinal products with narrow therapeutic windows that are substrates of CYP3A4 and /or CYP2C8 should be avoided. Concomitant treatment with substances that increase gastric pH should be avoided, as lapatinib solubility and absorption may decrease.
Effects of other medicinal products on lapatinib
Lapatinib is predominantly metabolised by CYP3A. In healthy volunteers receiving ketoconazole, a strong CYP3A4 inhibitor, at 200 mg twice daily for 7 days, systemic exposure to lapatinib (100 mg daily) was increased approximately 3.6–fold, and half-life increased 1.7–fold. Co-administration of Tyverb with strong inhibitors of CYP3A4 (e.g. ritonavir, saquinavir, telithromycin, ketoconazole, itraconazole, voriconazole, posaconazole, nefazodone) should be avoided. Co-administration of Tyverb with moderate inhibitors of CYP3A4 should proceed with caution and clinical adverse reactions should be carefully monitored. In healthy volunteers receiving carbamazepine, a CYP3A4 inducer, at 100 mg twice daily for 3 days and 200 mg twice daily for 17 days, systemic exposure to lapatinib was decreased approximately 72%. Co-administration of Tyverb with known inducers of CYP3A4 (e.g. rifampicin, rifabutin, carbamazepine, phenytoin or Hypericum perforatum [St John's Wort]) should be avoided.
Lapatinib is a substrate for the transport proteins Pgp and BCRP. Inhibitors (ketoconazole, itraconazole, quinidine, verapamil, cyclosporine, and erythromycin) and inducers (rifampicin and St John's Wort) of these proteins may alter the exposure and/or distribution of lapatinib. The solubility of lapatinib is pH-dependent. Concomitant treatment with substances that increase gastric pH should be avoided, as lapatinib solubility and absorption may decrease. Pre-treatment with a proton pump inhibitor (esomeprazole) decreased lapatinib exposure by an average of 27% (range: 6% to 49%). This effect decreases with increasing age from approximately 40 to 60 years.
Effects of lapatinib on other medicinal products
Lapatinib inhibits CYP3A4 in vitro at clinically relevant concentrations. Co-administration of Tyverb with orally administered midazolam resulted in an approximate 45% increase in the AUC of midazolam. There was no clinically meaningful increase in AUC when midazolam was dosed intravenously. Co-administration of Tyverb with orally administered medicinal products with narrow therapeutic windows that are substrates of CYP3A4 (e.g. cisapride, pimozide and quinidine) should be avoided. Lapatinib inhibits CYP2C8 in vitro at clinically relevant concentrations. Co-administration of Tyverb with medicinal products with narrow therapeutic windows that are substrates of CYP2C8 (e.g. repaglinide) should be avoided. Co-administration of lapatinib with intravenous paclitaxel increased the exposure of paclitaxel by 23%, due to lapatinib inhibition of CYP2C8 and/or Pgp. An increase in the incidence and severity of diarrhoea and neutropenia has been observed with this combination in clinical studies. Caution is advised if lapatinib is co-administered with paclitaxel. Co-administration of lapatinib with intravenously administered docetaxel did not significantly affect the AUC or Cmax of either active substance. However, the occurrence of docetaxel-induced neutropenia was increased. Co-administration of Tyverb with irinotecan (when administered as part of the FOLFIRI regimen) resulted in an approximate 40% increase in the AUC of SN-38, the active metabolite of irinotecan. The precise mechanism of this interaction is unknown, but it is assumed to be due to inhibition of one or more transport proteins by lapatinib. Adverse reactions should be carefully monitored if Tyverb is co-administered with irinotecan, and a reduction in the dose of irinotecan should be considered.
Lapatinib inhibits the transport protein Pgp in vitro at clinically relevant concentrations. Co-administration of lapatinib with orally administered digoxin resulted in an approximate 80% increase in the AUC of digoxin. Caution should be exercised when dosing lapatinib concurrently with medicinal products with narrow therapeutic windows that are substrates of Pgp, and a reduction in the dose of the Pgp substrate should be considered.
Lapatinib inhibits the transport proteins BCRP and OATP1B1 in vitro. The clinical relevance of this effect has not been evaluated. It cannot be excluded that lapatinib will affect the pharmacokinetics of substrates of BCRP (e.g. topotecan) and OATP1B1 (e.g. rosuvastatin).

Concomitant administration of Tyverb with capecitabine, letrozole or trastuzumab did not meaningfully alter the pharmacokinetics of these medicinal products (or the metabolites of capecitabine) or lapatinib.

Interactions with food and drink

The bioavailability of lapatinib is increased up to about 4 times by food, depending on e.g. the fat content in the meal. Furthermore, depending on type of food the bioavailability is approximately 2-3 times higher when lapatinib is taken 1 hour after food compared with 1 hour before the first meal of the day.

Grapefruit juice may inhibit CYP3A4 in the gut wall and increase the bioavailability of lapatinib and should therefore be avoided during treatment with Tyverb.

14.6 Overdose

There is no specific antidote for the inhibition of EGFR (ErbB1) and/or HER2 (ErbB2) tyrosine phosphorylation. The maximum oral dose of lapatinib that has been administered in clinical studies is 1800 mg once daily. Asymptomatic and symptomatic cases of overdose have been reported in patients being treated with Tyverb. In patients who took up to 5000 mg of lapatinib, symptoms observed include known lapatinib associated events and in some cases sore scalp and/or mucosal inflammation. In a single case of a patient who took 9000 mg of Tyverb, sinus tachycardia (with otherwise normal ECG) was also observed. Lapatinib is not significantly renally excreted and is highly bound to plasma proteins, therefore haemodialysis would not be expected to be an effective method to enhance the elimination of lapatinib. Further management should be as clinically indicated or as recommended by the national poisons centre, where available.

15 YERVOY (ipilimumab)

Investigators should also consult the local prescribing information if the IMP is approved in your country.

15.1 Standard dose

YERVOY as monotherapy: The recommended induction regimen of YERVOY is 3 mg/kg administered intravenously over a 90-minute period every 3 weeks for a total of 4 doses. Patients should receive the entire induction regimen (4 doses) as tolerated, regardless of the appearance of new lesions or growth of existing lesions. Assessments of tumour response should be conducted only after completion of induction therapy.

YERVOY in combination with nivolumab:
Option 1 (melanoma): The recommended dose is 3 mg/kg ipilimumab in combination with 1 mg/kg nivolumab administered intravenously every 3 weeks for the first 4 doses. This is then followed by a second phase in which nivolumab monotherapy is administered intravenously at either 240 mg every 2 weeks or at 480 mg every 4 weeks. For the monotherapy phase, the first dose of nivolumab should be administered: 3 weeks after the last dose of the combination of nivolumab and ipilimumab if using 240 mg every 2 weeks; or 6 weeks after the last dose of the combination of nivolumab and ipilimumab if using 480 mg every 4 weeks.

Option 2 (renal cell carcinoma, NSCLC): The recommended dose is 1 mg/kg ipilimumab in combination with 3 mg/kg nivolumab administered intravenously every 3 weeks for the first 4 doses. This is then followed by a second phase in which nivolumab monotherapy is administered intravenously at either 240 mg every 2 weeks or at 480 mg every 4 weeks. For the monotherapy phase, the first dose of nivolumab should be administered: 3 weeks after the last dose of the combination of ipilimumab and nivolumab if using 240 mg every 2 weeks; or 6 weeks after the last dose of the combination of ipilimumab and nivolumab if using 480 mg every 4 weeks.

Treatment with YERVOY in combination with nivolumab, should be continued for the four doses of combination therapy as long as clinical benefit is observed or until treatment is no longer tolerated by the patient. Atypical responses (i.e., an initial transient increase in tumour size or small new lesions within the first few months followed by tumour shrinkage) have been observed. It is recommended to continue treatment with YERVOY in combination with nivolumab for clinically stable patients with initial evidence of disease progression until disease progression is confirmed.

15.2 Contraindications

Hypersensitivity to the active substance or to any of the excipients listed: Tris hydrochloride (2-amino-2-hydroxymethyl-1,3-propanediol hydrochloride), Sodium chlorid, Mannitol (E421), Pentetic acid (diethylenetriaminepentaacetic acid)
Polysorbate 80, Sodium hydroxide (for pH-adjustment), Hydrochloric acid (for pH-adjustment).

15.3 Warnings and Precautions

Management of immune-related adverse reactions may require withholding of a dose or permanent discontinuation of YERVOY therapy and institution of systemic high-dose corticosteroid. In some cases, addition of other immunosuppressive therapy may be considered. Dose escalation or reduction is not recommended. Dosing delay or discontinuation may be required based on individual safety and tolerability. Guidelines for permanent discontinuation or withholding of doses are described in Tables 1A and 1B for YERVOY as monotherapy, and in Table 1C for YERVOY in combination with nivolumab or administration of the second phase of treatment (nivolumab monotherapy) following combination treatment.

**Table 1A**	**When to permanently discontinue YERVOY as monotherapy**
**Permanently discontinue YERVOY in patients with the following adverse reactions. Management of these adverse reactions may also require systemic high-dose corticosteroid therapy if demonstrated or suspected to be immune-related.**	
**Severe or life-threatening adverse reactions**	**NCI-CTCAE v4 Grade[a]**
**Gastrointestinal:**<br>Severe symptoms (abdominal pain, severe diarrhoea or significant change in the number of stools, blood in stool, gastrointestinal haemorrhage, gastrointestinal perforation)	Grade 3 or 4 diarrhoea or colitis
**Hepatic:**<br>Severe elevations in aspartate aminotransferase (AST), alanine aminotransferase (ALT), or total bilirubin or symptoms of hepatotoxicity	Grade 3 or 4 elevation in AST, ALT, or total bilirubin
**Skin:**<br>Life threatening skin rash (including Stevens-Johnson syndrome or toxic epidermal necrolysis) or severe widespread pruritus interfering with activities of daily living or requiring medical intervention	Grade 4 rash or Grade 3 pruritus
**Neurologic:**<br>New onset or worsening severe motor or sensory neuropathy	Grade 3 or 4 motor or sensory neuropathy
**Other organ systems[b]:**<br>(e.g. nephritis, pneumonitis, pancreatitis, non-infectious myocarditis)	≥ Grade 3 immune-related reactions[c]<br>≥ Grade 2 for immune-related eye disorders NOT responding to topical immunosuppressive therapy

a. Toxicity grades are in accordance with National Cancer Institute Common Terminology Criteria for Adverse Events

b. Any other adverse reactions that are demonstrated or suspected to be immune-related should be graded according to Version 4.0 (NCI-CTCAE v4).

c. Patients with severe (Grade 3 or 4) endocrinopathy controlled with hormone replacement therapy may remain on therapy.

**Table 1B**	**When to withhold dose of YERVOY as monotherapy**
**Withhold YERVOY dose[a] in patients with the following immune-related adverse reactions.**	

Mild to moderate Immune-related adverse reactions	Action
**Gastrointestinal:**<br>Moderate diarrhoea or colitis that either is not controlled with medical management or that persists (5-7 days) or recurs	1. Withhold dose until an adverse reaction resolves to Grade 1 or Grade 0 (or returns to baseline).<br>2. If resolution occurs, resume therapy.[d]<br>3. If resolution has not occurred, continue to withhold doses until resolution then resume treatment.[d]<br>4. Discontinue YERVOY if resolution to Grade 1 or Grade 0 or return to baseline does not occur.
**Hepatic:**<br>Grade 2 elevation in AST, ALT, or total bilirubin	
**Skin:**<br>Moderate to severe (Grade 3)[b] skin rash or (Grade 2) widespread/intense pruritus regardless of etiology	
**Endocrine:**<br>Severe adverse reactions in the endocrine glands, such as hypophysitis and thyroiditis that are not adequately controlled with hormone replacement therapy or high-dose immunosuppressive therapy	
**Neurological:**<br>Moderate (Grade 2)[b] unexplained motor neuropathy, muscle weakness, or sensory neuropathy (lasting more than 4 days)	
**Other moderate adverse reactions**[c]	

a No dose reduction of YERVOY is recommended.

b Toxicity grades are in accordance with National Cancer Institute Common Terminology Criteria for Adverse Events. Version 4.0 (NCI-CTCAE v4).

c Any other organ system adverse reactions that are considered immune-related should be graded according to CTCAE. Decision whether to withhold a dose should be based on severity.

d Until administration of all 4 doses or 16 weeks from first dose, whichever occurs earlier.

Table 1C: Recommended treatment modifications for YERVOY in combination with nivolumab or administration of the second phase of treatment (nivolumab monotherapy) following combination treatment

Immune-related adverse reaction	Severity	Treatment modification
Immune-related pneumonitis	Grade 2 pneumonitis	Withhold dose(s) until symptoms resolve, radiographic abnormalities improve, and management with corticosteroids is complete
	Grade 3 or 4 pneumonitis	Permanently discontinue treatment

Immune-related colitis	Grade 2 diarrhoea or colitis	Withhold dose(s) until symptoms resolve and management with corticosteroids, if needed, is complete
	Grade 3 or 4 diarrhoea or colitis	Permanently discontinue treatment
Immune-related hepatitis	Grade 2 elevation in aspartate aminotransferase (AST), alanine aminotransferase (ALT), or total bilirubin	Withhold dose(s) until laboratory values return to baseline and management with corticosteroids, if needed, is complete
	Grade 3 or 4 elevation in AST, ALT, or total bilirubin	Permanently discontinue treatment
Immune-related nephritis and renal dysfunction	Grade 2 or 3 creatinine elevation	Withhold dose(s) until creatinine returns to baseline and management with corticosteroids is complete
	Grade 4 creatinine elevation	Permanently discontinue treatment
Immune-related endocrinopathies	Symptomatic Grade 2 or 3 hypothyroidism, hyperthyroidism, hypophysitis,<br>Grade 2 adrenal insufficiency<br>Grade 3 diabetes	Withhold dose(s) until symptoms resolve and management with corticosteroids (if needed for symptoms of acute inflammation) is complete. Treatment should be continued in the presence of hormone replacement therapy[a] as long as no symptoms are present
	Grade 4 hypothyroidism<br>Grade 4 hyperthyroidism<br>Grade 4 hypophysitis<br>Grade 3 or 4 adrenal insufficiency<br>Grade 4 diabetes	Permanently discontinue treatment
Immune-related skin adverse reactions	Grade 3 rash	Withhold dose(s) until symptoms resolve and management with corticosteroids is complete
	Grade 4 rash	Permanently discontinue treatment
	Stevens-Johnson syndrome (SJS) or toxic epidermal necrolysis (TEN)	Permanently discontinue treatment
Immune-related myocarditis	Grade 2 myocarditis	Withhold dose(s) until symptoms resolve and management with corticosteroids is completeb
	Grade 3 or 4 myocarditis	Permanently discontinue treatment

Note: Toxicity grades are in accordance with National Cancer Institute Common Terminology Criteria for Adverse Events Version 4.0 (NCI-CTCAE v4).
a

b The safety of re-initiating ipilimumab in combination with nivolumab therapy in patients previously experiencing immune-related myocarditis is not known.

YERVOY in combination with nivolumab should be permanently discontinued for: Grade 4 or recurrent Grade 3 adverse reactions; Persistent Grade 2 or 3 adverse reactions despite management.
When YERVOY is administered in combination with nivolumab, if either agent is withheld, the other agent should also be withheld. If dosing is resumed after a delay, either the combination treatment or nivolumab monotherapy could be resumed based on the evaluation of the individual patient.

Immune-related gatroitestinal reactions
Management recommendations for diarrhoea or colitis are based on severity of symptoms (per NCI-CTCAE v4 severity grading classification). Patients with mild to moderate (Grade 1 or 2) diarrhoea (an increase of up to 6 stools per day) or suspected mild to moderate colitis (e.g. abdominal pain or blood in stools) may remain on ipilimumab. Symptomatic treatment (e.g. loperamide, fluid replacement) and close monitoring are advised. If mild to moderate symptoms recur or persist for 5-7 days, the scheduled dose of ipilimumab should be withheld and corticosteroid therapy (e.g. prednisone 1 mg/kg orally once daily or equivalent) should be initiated. If resolution to Grades 0-1 or return to baseline occurs, ipilimumab may be resumed. Ipilimumab must be permanently discontinued in patients with severe (Grade 3 or 4) diarrhoea or colitis and systemic high-dose intravenous corticosteroid therapy should be initiated immediately. (In clinical trials, methylprednisolone 2 mg/kg/day has been used). Once diarrhoea and other symptoms are controlled, the initiation of corticosteroid taper should be based on clinical judgment. In clinical trials, rapid tapering (over periods < 1 month) resulted in recurrence of diarrhoea or colitis in some patients. Patients must be evaluated for evidence of gastrointestinal perforation or peritonitis. The experience from clinical trials on the management of corticosteroid-refractory diarrhoea or colitis is limited. Addition of an alternative immunosuppressive agent to the corticosteroid regimen should be considered in corticosteroid-refractory immune-related colitis if other causes are excluded (including Cytomegalovirus (CMV) infection/reactivation evaluated with viral PCR on biopsy, and other viral, bacterial and parasitic etiology). In clinical trials, a single dose of infliximab 5 mg/kg was added unless contraindicated. Infliximab must not be used if gastrointestinal perforation or sepsis is suspected (see the Summary of Product Characteristics for infliximab).

Immune-related pneumonitis
Severe pneumonitis or interstitial lung disease, including fatal cases, has been observed with ipilimumab in combination with nivolumab. Patients should be monitored for signs and symptoms of pneumonitis such as radiographic changes (e.g., focal ground glass opacities, patchy filtrates), dyspnoea, and hypoxia. Infectious and disease-related aetiologies should be ruled out. For Grade 3 or 4 pneumonitis, ipilimumab in combination with nivolumab must be permanently discontinued, and corticosteroids should be initiated at a dose of 2 to 4 mg/kg/day methylprednisolone equivalents. For Grade 2 (symptomatic) pneumonitis, ipilimumab in combination with nivolumab should be withheld and corticosteroids initiated at a dose of 1 mg/kg/day methylprednisolone equivalents. Upon improvement, ipilimumab in combination with nivolumab may be resumed after corticosteroid taper. If worsening or no improvement occurs despite initiation of corticosteroids, corticosteroid dose should be increased to 2 to 4 mg/kg/day methylprednisolone equivalents and ipilimumab in combination with nivolumab must be permanently discontinued.

Immune-related hepatotoxicity
Ipilimumab as monotherapy
Ipilimumab is associated with serious immune-related hepatotoxicity. Fatal hepatic failure has been reported in clinical trials. In patients who received ipilimumab 3 mg/kg monotherapy in MDX010-20, time to onset of moderate to severe or fatal (Grade 2-5) immune-related hepatotoxicity ranged from 3 to 9 weeks from the start of treatment. With protocol-specified management guidelines, time to resolution ranged from 0.7 to 2 weeks.

Hepatic transaminase and bilirubin must be evaluated before each dose of ipilimumab, as early laboratory changes may be indicative of emerging immune-related hepatitis. Elevations in LFTs may develop in the absence of clinical symptoms. Increases in AST and ALT or total bilirubin should be evaluated to exclude other causes of hepatic injury, including infections, tumour progression, or concomitant medication and monitored until resolution. Liver biopsies from patients who had immune-related hepatotoxicity showed evidence of acute inflammation (neutrophils, lymphocytes, and macrophages). For patients with Grade 2 transaminase or total bilirubin elevation, the scheduled dose of ipilimumab should be withheld, and LFTs must be monitored until resolution. Upon improvement, ipilimumab may be resumed. For patients with Grade 3 or 4 transaminase or total bilirubin elevation, treatment must be permanently discontinued, and systemic high-dose intravenous corticosteroid therapy (e.g. methylprednisolone 2 mg/kg daily or equivalent) should be initiated immediately. In such patients, LFTs must be monitored until normalization. Once symptoms have resolved and LFTs show sustained improvement or return to baseline, the initiation of corticosteroid taper should be based on clinical judgment. Tapering should occur over a period of at least 1 month. Elevations in LFTs during taper may be managed with an increase in the dose of corticosteroid and a slower taper. For patients with significant LFT elevations that are refractory to corticosteroid therapy, addition of an alternative immunosuppressive agent to the corticosteroid regimen may be considered. In clinical trials, mycophenolate mofetil was used in patients without response to corticosteroid therapy, or who had an LFT elevation during corticosteroid tapering that was not responsive to an increase in the dose of corticosteroids (see the Summary of Product Characteristics for mycophenolate mofetil).

Ipilimumab in combination with nivolumab

Severe hepatitis has been observed with ipilimumab in combination with nivolumab. Patients should be monitored for signs and symptoms of hepatitis such as transaminase and total bilirubin elevations. Infectious and disease-related aetiologies should be ruled out. For Grade 3 or 4 transaminase or total bilirubin elevation, ipilimumab in combination with nivolumab must be permanently discontinued, and corticosteroids should be initiated at a dose of 1 to 2 mg/kg/day methylprednisolone equivalents. For Grade 2 transaminase or total bilirubin elevation, ipilimumab in combination with nivolumab should be withheld. Persistent elevations in these laboratory values should be managed with corticosteroids at a dose of 0.5 to 1 mg/kg/day methylprednisolone equivalents. Upon improvement, ipilimumab in combination with nivolumab may be resumed after corticosteroid taper, if needed. If worsening or no improvement occurs despite initiation of corticosteroids, corticosteroid dose should be increased to 1 to 2 mg/kg/day methylprednisolone equivalents and ipilimumab in combination with nivolumab must be permanently discontinued.

Immune-related skin adverse reactions

Caution should be used when considering the use of ipilimumab or ipilimumab in combination with nivolumab in a patient who has previously experienced a severe or life-threatening skin adverse reaction on a prior cancer immune stimulatory therapy).

Ipilimumab as monotherapy

Ipilimumab is associated with serious skin adverse reactions that may be immune-related. Rare cases of toxic epidermal necrolysis (TEN) (including Steven Johnson Syndrome) have been observed, some with fatal outcome. Rare cases of Drug Reaction with Eosinophilia and Systemic Symptoms (DRESS) have also been reported in clinical trials and during post-marketing use. DRESS presents as a rash with eosinophilia associated with one or more of the following features: fever, lymphadenopathy, facial oedema, and internal organ involvement (hepatic, renal, pulmonary). DRESS may be characterized by a long latency (two to eight weeks) between medicinal product exposure and disease onset. Ipilimumab-induced rash and pruritus were predominantly mild or moderate (Grade 1 or 2) and responsive to symptomatic therapy. In patients who received ipilimumab 3 mg/kg monotherapy in MDX010-20, the median time to onset of moderate to severe or fatal (Grade 2-5) skin adverse reactions was 3 weeks (range 0.9-16 weeks) from start of treatment. With protocol-specified management guidelines, resolution occurred in most cases (87%), with a median time from onset to resolution of 5 weeks (range 0.6 to 29 weeks). Ipilimumab-induced rash and pruritus should be managed based on severity. Patients with a mild to moderate (Grade

1 or 2) rash may remain on ipilimumab therapy with symptomatic treatment (e.g. antihistamines). For mild to moderate rash or mild pruritus that persists for 1 to 2 weeks and does not improve with topical corticosteroids, oral corticosteroid therapy should be initiated (e.g. prednisone 1 mg/kg once daily or equivalent).
For patients with a severe (Grade 3) rash, the scheduled dose of ipilimumab should be withheld. If initial symptoms improve to mild (Grade 1) or resolve, ipilimumab therapy may be resumed.
Ipilimumab must be permanently discontinued in patients with a very severe (Grade 4) rash or severe (Grade 3) pruritus, and systemic high-dose intravenous corticosteroid therapy (e.g. methylprednisolone 2 mg/kg/day) should be initiated immediately. Once rash or pruritus is controlled, initiation of corticosteroid taper should be based on clinical judgment. Tapering should occur over a period of at least 1 month.

Ipilimumab in combination with nivolumab
Severe rash has been observed with ipilimumab in combination with nivolumab. Ipilimumab in combination with nivolumab should be withheld for Grade 3 rash and discontinued for Grade 4 rash. Severe rash should be managed with high-dose corticosteroid at a dose of 1 to 2 mg/kg/day methylprednisolone equivalents. Rare cases of SJS and TEN, some of them with fatal outcome, have been observed. If symptoms or signs of SJS or TEN appear, treatment with ipilimumab in combination with nivolumab should be discontinued and the patient referred to a specialised unit for assessment and treatment. If the patient has developed SJS or TEN with the use of ipilimumab in combination with nivolumab, permanent discontinuation of treatment is recommended.

Immune-related neurological reactions
Ipilimumab as monotherapy
Ipilimumab is associated with serious immune -related neurological adverse reactions. Fatal Guillain-Barré syndrome has been reported in clinical trials. Myasthenia gravis-like symptoms have also been reported. Patients may present with muscle weakness. Sensory neuropathy may also occur. Unexplained motor neuropathy, muscle weakness, or sensory neuropathy lasting > 4 days must be evaluated, and non-inflammatory causes such as disease progression, infections, metabolic syndromes and concomitant medication should be excluded. For patients with moderate (Grade 2) neuropathy (motor with or without sensory) likely related to ipilimumab, the scheduled dose should be withheld. If neurologic symptoms resolve to baseline, the patient may resume ipilimumab.
Ipilimumab must be permanently discontinued in patients with severe (Grade 3 or 4) sensory neuropathy suspected to be related to ipilimumab. Patients must be treated according to institutional guidelines for management of sensory neuropathy, and intravenous corticosteroids (e.g. methylprednisolone 2 mg/kg/day) should be initiated immediately.
Progressive signs of motor neuropathy must be considered immune-related and managed accordingly. Ipilimumab must be permanently discontinued in patients with severe (Grade 3 or 4) motor neuropathy regardless of causality.

Immune-related nephritis and renal dysfunction
Ipilimumab in combination with nivolumab
Severe nephritis and renal dysfunction have been observed with ipilimumab in combination with nivolumab. Patients should be monitored for signs and symptoms of nephritis or renal dysfunction. Most patients present with asymptomatic increases in serum creatinine. Disease-related aetiologies should be ruled out.
For Grade 4 serum creatinine elevation, ipilimumab in combination with nivolumab must be permanently discontinued, and corticosteroids should be initiated at a dose of 1 to 2 mg/kg/day methylprednisolone equivalents.
For Grade 2 or 3 serum creatinine elevation, ipilimumab in combination with nivolumab should be withheld, and corticosteroids should be initiated at a dose of 0.5 to 1 mg/kg/day methylprednisolone equivalents. Upon improvement, ipilimumab in combination with nivolumab may be resumed after

corticosteroid taper. If worsening or no improvement occurs despite initiation of corticosteroids, corticosteroid dose should be increased to 1 to 2 mg/kg/day methylprednisolone equivalents, and ipilimumab in combination with nivolumab must be permanently discontinued.

Immune-related endocrinopathy

Ipilimumab as monotherapy

Ipilimumab can cause inflammation of the endocrine system organs, manifesting as hypophysitis, hypopituitarism, adrenal insufficiency, and hypothyroidism, and patients may present with nonspecific symptoms, which may resemble other causes such as brain metastasis or underlying disease. The most common clinical presentation includes headache and fatigue. Symptoms may also include visual field defects, behavioural changes, electrolyte disturbances, and hypotension. Adrenal crisis as a cause of the patient's symptoms must be excluded. Clinical experience with ipilimumab-associated endocrinopathy is limited.

For patients who received ipilimumab 3 mg/kg monotherapy in MDX010-20, time to onset of moderate to very severe (Grade 2-4) immune-related endocrinopathy ranged from 7 to nearly 20 weeks from the start of treatment. Immune-related endocrinopathy observed in clinical trials was generally controlled with immunosuppressive therapy and hormone replacement therapy.

If there are any signs of adrenal crisis such as severe dehydration, hypotension, or shock, immediate administration of intravenous corticosteroids with mineralocorticoid activity is recommended, and the patient must be evaluated for presence of sepsis or infections. If there are signs of adrenal insufficiency but the patient is not in adrenal crisis, further investigations should be considered including laboratory and imaging assessment. Evaluation of laboratory results to assess endocrine function may be performed before corticosteroid therapy is initiated. If pituitary imaging or laboratory tests of endocrine function are abnormal, a short course of high-dose corticosteroid therapy (e.g. dexamethasone 4 mg every 6 hrs or equivalent) is recommended to treat the inflammation of the affected gland, and the scheduled dose of ipilimumab should be withheld. It is currently unknown if the corticosteroid treatment reverses the gland dysfunction. Appropriate hormone replacement should also be initiated. Long-term hormone replacement therapy may be necessary.

Once symptoms or laboratory abnormalities are controlled and overall patient improvement is evident, treatment with ipilimumab may be resumed and initiation of corticosteroid taper should be based on clinical judgment. Tapering should occur over a period of at least 1 month.

Ipilimumab in combination with nivolumab

Severe endocrinopathies, including hypothyroidism, hyperthyroidism, adrenal insufficiency (including secondary adrenocortical insufficiency), hypophysitis (including hypopituitarism), diabetes mellitus, and diabetic ketoacidosis have been observed with ipilimumab in combination with nivolumab.

Patients should be monitored for clinical signs and symptoms of endocrinopathies and for hyperglycaemia and changes in thyroid function (at the start of treatment, periodically during treatment, and as indicated based on clinical evaluation). Patients may present with fatigue, headache, mental status changes, abdominal pain, unusual bowel habits, and hypotension, or nonspecific symptoms which may resemble other causes such as brain metastasis or underlying disease. Unless an alternate aetiology has been identified, signs or symptoms of endocrinopathies should be considered immune-related.

For symptomatic hypothyroidism, ipilimumab in combination with nivolumab should be withheld, and thyroid hormone replacement should be initiated as needed. For symptomatic hyperthyroidism, ipilimumab in combination with nivolumab should be withheld and antithyroid medication should be initiated as needed. Corticosteroids at a dose of 1 to 2 mg/kg/day methylprednisolone equivalents should also be considered if acute inflammation of the thyroid is suspected. Upon improvement, ipilimumab in combination with nivolumab may be resumed after corticosteroid taper, if needed. Monitoring of thyroid function should continue to ensure appropriate hormone replacement is utilised. Ipilimumab in combination with nivolumab must be permanently discontinued for life-threatening hyperthyroidism or hypothyroidism.

For symptomatic Grade 2 adrenal insufficiency, ipilimumab in combination with nivolumab should be withheld, and physiologic corticosteroid replacement should be initiated as needed. ipilimumab in combination with nivolumab must be permanently discontinued for severe (Grade 3) or life-threatening (Grade 4) adrenal insufficiency. Monitoring of adrenal function and hormone levels should continue to ensure appropriate corticosteroid replacement is utilised.

For symptomatic Grade 2 or 3 hypophysitis, ipilimumab in combination with nivolumab should be withheld, and hormone replacement should be initiated as needed. Corticosteroids at a dose of 1 to 2 mg/kg/day methylprednisolone equivalents should also be considered if acute inflammation of the pituitary gland is suspected. Upon improvement, ipilimumab in combination with nivolumab may be resumed after corticosteroid taper, if needed. Ipilimumab in combination with nivolumab must be permanently discontinued for life-threatening (Grade 4) hypophysitis. Monitoring of pituitary function and hormone levels should continue to ensure appropriate hormone replacement is utilised.

For symptomatic diabetes, ipilimumab in combination with nivolumab should be withheld, and insulin replacement should be initiated as needed. Monitoring of blood sugar should continue to ensure appropriate insulin replacement is utilised. Ipilimumab in combination with nivolumab must be permanently discontinued for life-threatening diabetes.

Infusion reaction

Ipilimumab as monotherapy or in combination with nivolumab

Severe infusion reactions have been reported in clinical trials of ipilimumab or ipilimumab in combination with nivolumab. In case of a severe or life-threatening infusion reaction, the ipilimumab or ipilimumab in combination with nivolumab infusion must be discontinued and appropriate medical therapy administered. Patients with mild or moderate infusion reaction may receive ipilimumab or ipilimumab in combination with nivolumab with close monitoring and use of premedication according to local treatment guidelines for prophylaxis of infusion reactions.

15.4 Special Populations

Elderly

No overall differences in safety or efficacy were reported between elderly (≥ 65 years) and younger patients (< 65 years). Data from first-line RCC patients 75 years of age or older are too limited to draw conclusions on this population. No specific dose adjustment is necessary in this population.

Renal impairment

The safety and efficacy of YERVOY have not been studied in patients with renal impairment. Based on population pharmacokinetic results, no specific dose adjustment is necessary in patients with mild to moderate renal dysfunction.

Hepatic impairment

The safety and efficacy of YERVOY have not been studied in patients with hepatic impairment. Based on the population pharmacokinetic results, no specific dose adjustment is necessary in patients with mild hepatic impairment. YERVOY must be administered with caution in patients with transaminase levels ≥ 5 x ULN or bilirubin levels > 3 x ULN at baseline.

Patients with autoimmune disease

Patients with a history of autoimmune disease (other than vitiligo and adequately controlled endocrine deficiencies such as hypothyroidism), including those who require systemic immunosuppressive therapy for pre-existing active autoimmune disease or for organ transplantation graft maintenance, were not evaluated in clinical trials. Ipilimumab is a T-cell potentiator that enables the immune response and may interfere with immunosuppressive therapy, resulting in an exacerbation of the underlying disease or increased risk of graft rejection. Ipilimumab should be avoided in patients with severe active autoimmune disease where further immune activation is potentially imminently life threatening. In other patients with a history of autoimmune

disease, ipilimumab should be used with caution after careful consideration of the potential risk-benefit on an individual basis.

Patients on controlled sodium diet

Each ml of this medicinal product contains 0.1 mmol (or 2.30 mg) sodium. To be taken into consideration when treating patients on a controlled sodium diet.

Reproductive Toxicity, Pregnancy

Refer to appendix 1.

15.5 Concomitant use with other medications

Concurrent administration with vemurafenib

In a Phase 1 trial, asymptomatic grade 3 increases in transaminases (ALT/AST > 5 × ULN) and bilirubin (total bilirubin > 3 × ULN) were reported with concurrent administration of ipilimumab (3 mg/kg) and vemurafenib (960 mg BID or 720 mg BID). Based on these preliminary data, the concurrent administration of ipilimumab and vemurafenib is not recommended.

Sequential administration with vemurafenib

In a Phase 2 trial, the sequential treatment with vemurafenib followed by 10 mg/kg ipilimumab in patients with BRAF-mutated metastatic melanoma showed a higher incidence of Grade 3+ skin adverse reactions than with ipilimumab alone. Caution should be used when ipilimumab is administered following prior vemurafenib

Ipilimumab is a human monoclonal antibody that is not metabolized by cytochrome P450 enzymes (CYPs) or other drug metabolizing enzymes.

A drug-interaction study in adults of ipilimumab administered alone and in combination with chemotherapy (dacarbazine or paclitaxel/carboplatin) was conducted evaluating interaction with CYP isozymes (particularly CYP1A2, CYP2E1, CYP2C8, and CYP3A4) in patients with treatment-naive advanced melanoma. No clinically relevant pharmacokinetic drug-drug interaction was observed between ipilimumab and paclitaxel/carboplatin, dacarbazine or its metabolite, 5-aminoimidazole-4-carboxamide (AIC).

Corticosteroids

The use of systemic corticosteroids at baseline, before starting ipilimumab, should be avoided because of their potential interference with the pharmacodynamic activity and efficacy of ipilimumab. However, systemic corticosteroids or other immunosuppressants can be used after starting ipilimumab to treat immune-related adverse reactions. The use of systemic corticosteroids after starting ipilimumab treatment does not appear to impair the efficacy of ipilimumab.

Anticoagulants

The use of anticoagulants is known to increase the risk of gastrointestinal haemorrhage. Since gastrointestinal haemorrhage is an adverse reaction with ipilimumab, patients who require concomitant anticoagulant therapy should be monitored closely.

15.6 Overdose

The maximum tolerated dose of ipilimumab has not been determined. In clinical trials, patients received up to 20 mg/kg without apparent toxic effects. In case of overdose, patients must be closely monitored for signs or symptoms of adverse reactions, and appropriate symptomatic treatment instituted.

16 OPDIVO (nivolumab)

Investigators should also consult the local prescribing information if the IMP is approved in your country.

16.1 Standard dose

OPDIVO as monotherapy
The recommended dose of OPDIVO is either nivolumab 240 mg every 2 weeks or 480 mg every 4 weeks.

OPDIVO in combination with ipilimumab: see Yervoy (ipilimumab) chapter 15

OPDIVO is for intravenous use only. It is to be administered as an intravenous infusion over a period of 30 or 60 minutes depending on the dose. The infusion must be administered through a sterile, non-pyrogenic, low protein binding in-line filter with a pore size of 0.2-1.2 µm. OPDIVO must not be administered as an intravenous push or bolus injection. The total dose of OPDIVO required can be infused directly as a 10 mg/mL solution or can be diluted with sodium chloride 9 mg/mL (0.9%) solution for injection or glucose 50 mg/mL (5%) solution for injection. When administered in combination with ipilimumab, OPDIVO should be given first followed by ipilimumab on the same day. Use separate infusion bags and filters for each infusion.

Atypical responses (i.e., an initial transient increase in tumour size or small new lesions within the first few months followed by tumour shrinkage) have been observed. It is recommended to continue treatment with nivolumab or nivolumab in combination with ipilimumab for clinically stable patients with initial evidence of disease progression until disease progression is confirmed.
Dose escalation or reduction is not recommended. Dosing delay or discontinuation may be required based on individual safety and tolerability.

16.2 Contraindications

Hypersensitivity to the active substance or to any of the excipients listed: Sodium citrate dehydrate, Sodium chloride, Mannitol (E421), Pentetic acid (diethylenetriaminepentaacetic acid), Polysorbate 80, Sodium hydroxide (for pH adjustment), Hydrochloric acid (for pH adjustment).

16.3 Warnings and Precautions

When nivolumab is administered in combination with ipilimumab see Warning and Precautions for Yervoy (Chapter 15).

Table 1: Recommended treatment modifications for OPDIVO monotherapy or in combination with YERVOY (ipilimumab)

Immune-related adverse reaction	Severity	Treatment modification
Immune-related pneumonitis	Grade 2 pneumonitis	Withhold dose(s) until symptoms resolve, radiographic abnormalities improve, and management with corticosteroids is complete
	Grade 3 or 4 pneumonitis	Permanently discontinue treatment
Immune-related colitis	Grade 2 diarrhoea or colitis	Withhold dose(s) until symptoms resolve and management with corticosteroids, if needed, is complete
	Grade 3 or 4 diarrhoea or colitis	Permanently discontinue treatment

Immune-related hepatitis	Grade 2 elevation in aspartate aminotransferase (AST), alanine aminotransferase (ALT), or total bilirubin	Withhold dose(s) until laboratory values return to baseline and management with corticosteroids, if needed, is complete
	Grade 3 or 4 elevation in AST, ALT, or total bilirubin	Permanently discontinue treatment
Immune-related nephritis and renal dysfunction	Grade 2 or 3 creatinine elevation	Withhold dose(s) until creatinine returns to baseline and management with corticosteroids is complete
	Grade 4 creatinine elevation	Permanently discontinue treatment
Immune-related endocrinopathies	Symptomatic Grade 2 or 3 hypothyroidism, hyperthyroidism, hypophysitis,<br>Grade 2 adrenal insufficiency<br>Grade 3 diabetes	Withhold dose(s) until symptoms resolve and management with corticosteroids (if needed for symptoms of acute inflammation) is complete. Treatment should be continued in the presence of hormone replacement therapy[a] as long as no symptoms are present
	Grade 4 hypothyroidism<br>Grade 4 hyperthyroidism<br>Grade 4 hypophysitis<br>Grade 3 or 4 adrenal insufficiency<br>Grade 4 diabetes	Permanently discontinue treatment
Immune-related skin adverse reactions	Grade 3 rash	Withhold dose(s) until symptoms resolve and management with corticosteroids is complete
	Grade 4 rash	Permanently discontinue treatment
	Stevens-Johnson syndrome (SJS) or toxic epidermal necrolysis (TEN)	Permanently discontinue treatment
Immune-related myocarditis	Grade 2 myocarditis	Withhold dose(s) until symptoms resolve and management with corticosteroids is completeb
	Grade 3 or 4 myocarditis	Permanently discontinue treatment

OPDIVO or OPDIVO in combination with ipilimumab should be permanently discontinued for:
- Grade 4 or recurrent Grade 3 adverse reactions;
- Persistent Grade 2 or 3 adverse reactions despite management.

Immune-related pneumonitis

Patients should be monitored for signs and symptoms of pneumonitis such as radiographic changes (e.g., focal ground glass opacities, patchy filtrates), dyspnoea, and hypoxia. Infectious and disease-related aetiologies should be ruled out.

For Grade 3 or 4 pneumonitis, nivolumab or nivolumab in combination with ipilimumab must be permanently discontinued, and corticosteroids should be initiated at a dose of 2 to 4 mg/kg/day methylprednisolone equivalents.
For Grade 2 (symptomatic) pneumonitis, nivolumab or nivolumab in combination with ipilimumab should be withheld and corticosteroids initiated at a dose of 1 mg/kg/day methylprednisolone equivalents. Upon improvement, nivolumab or nivolumab in combination with ipilimumab may be resumed after corticosteroid taper. If worsening or no improvement occurs despite initiation of corticosteroids, corticosteroid dose should be increased to 2 to 4 mg/kg/day methylprednisolone
equivalents and nivolumab or nivolumab in combination with ipilimumab must be permanently discontinued.

Immune-related colitis
For Grade 4 diarrhoea or colitis, nivolumab or nivolumab in combination with ipilimumab must be permanently discontinued, and corticosteroids should be initiated at a dose of 1 to 2 mg/kg/day methylprednisolone equivalents.
Nivolumab monotherapy should be withheld for Grade 3 diarrhoea or colitis, and corticosteroids initiated at a dose of 1 to 2 mg/kg/day methylprednisolone equivalents. Upon improvement, nivolumab monotherapy may be resumed after corticosteroid taper. If worsening or no improvement occurs despite initiation of corticosteroids, nivolumab monotherapy must be permanently discontinued. Grade 3 diarrhoea or colitis observed with nivolumab in combination with ipilimumab requires permanent discontinuation of treatment and initiation of corticosteroids at a dose of 1 to 2 mg/kg/day methylprednisolone equivalents.
For Grade 2 diarrhoea or colitis, nivolumab or nivolumab in combination with ipilimumab should be withheld. Persistent diarrhoea or colitis should be managed with corticosteroids at a dose of 0.5 to 1 mg/kg/day methylprednisolone equivalents. Upon improvement, nivolumab or nivolumab in combination with ipilimumab may be resumed after corticosteroid taper, if needed. If worsening or no improvement occurs despite initiation of corticosteroids, corticosteroid dose should be increased to 1 to 2 mg/kg/day methylprednisolone equivalents and nivolumab or nivolumab in combination with ipilimumab must be permanently discontinued.

Immune-related hepatitis
Severe hepatitis has been observed with nivolumab monotherapy or nivolumab in combination with ipilimumab. Patients should be monitored for signs and symptoms of hepatitis such as transaminase and total bilirubin elevations. Infectious and disease-related aetiologies should be ruled out.
For Grade 3 or 4 transaminase or total bilirubin elevation, nivolumab or nivolumab in combination with ipilimumab must be permanently discontinued, and corticosteroids should be initiated at a dose of 1 to 2 mg/kg/day methylprednisolone equivalents.
For Grade 2 transaminase or total bilirubin elevation, nivolumab or nivolumab in combination with ipilimumab should be withheld. Persistent elevations in these laboratory values should be managed with corticosteroids at a dose of 0.5 to 1 mg/kg/day methylprednisolone equivalents. Upon improvement, nivolumab or nivolumab in combination with ipilimumab may be resumed after corticosteroid taper, if needed. If worsening or no improvement occurs despite initiation of
corticosteroids, corticosteroid dose should be increased to 1 to 2 mg/kg/day methylprednisolone equivalents and nivolumab or nivolumab in combination with ipilimumab must be permanently discontinued.

Immune-related nephritis and renal dysfunction
Severe nephritis and renal dysfunction have been observed with monotherapy treatment or nivolumab in combination with ipilimumab. Patients should be monitored for signs and symptoms of nephritis or renal dysfunction. Most patients present with asymptomatic increases in serum creatinine. Disease-related aetiologies should be ruled out.

For Grade 4 serum creatinine elevation, nivolumab or nivolumab in combination with ipilimumab must be permanently discontinued, and corticosteroids should be initiated at a dose of 1 to 2 mg/kg/day methylprednisolone equivalents.
For Grade 2 or 3 serum creatinine elevation, nivolumab or nivolumab in combination with ipilimumab should be withheld, and corticosteroids should be initiated at a dose of 0.5 to 1 mg/kg/day methylprednisolone equivalents. Upon improvement, nivolumab or nivolumab in combination with ipilimumab may be resumed after corticosteroid taper. If worsening or no improvement occurs despite initiation of corticosteroids, corticosteroid dose should be increased to 1 to 2 mg/kg/day methylprednisolone equivalents, and nivolumab or nivolumab in combination with ipilimumab must be permanently discontinued.

Immune-related endocrinopathies

Severe endocrinopathies, including hypothyroidism, hyperthyroidism, adrenal insufficiency (including secondary adrenocortical insufficiency), hypophysitis (including hypopituitarism), diabetes mellitus, and diabetic ketoacidosis have been observed with nivolumab monotherapy or nivolumab in combination with ipilimumab. Patients should be monitored for clinical signs and symptoms of endocrinopathies and for hyperglycaemia and changes in thyroid function (at the start of treatment, periodically during treatment, and as indicated based on clinical evaluation). Patients may present with fatigue, headache, mental status changes, abdominal pain, unusual bowel habits, and hypotension, or nonspecific
symptoms which may resemble other causes such as brain metastasis or underlying disease. Unless an alternate aetiology has been identified, signs or symptoms of endocrinopathies should be considered immune-related.

For symptomatic hypothyroidism, nivolumab or nivolumab in combination with ipilimumab should be withheld, and thyroid hormone replacement should be initiated as needed. For symptomatic hyperthyroidism, nivolumab or nivolumab in combination with ipilimumab should be withheld and antithyroid medication should be initiated as needed. Corticosteroids at a dose of 1 to 2 mg/kg/day
methylprednisolone equivalents should also be considered if acute inflammation of the thyroid is suspected. Upon improvement, nivolumab or nivolumab in combination with ipilimumab may be resumed after corticosteroid taper, if needed. Monitoring of thyroid function should continue to ensure appropriate hormone replacement is utilised. Nivolumab or nivolumab in combination with
ipilimumab must be permanently discontinued for life-threatening hyperthyroidism or hypothyroidism.
For symptomatic Grade 2 adrenal insufficiency, nivolumab or nivolumab in combination with ipilimumab should be withheld, and physiologic corticosteroid replacement should be initiated as needed. Nivolumab or nivolumab in combination with ipilimumab must be permanently discontinued for severe (Grade 3) or life-threatening (Grade 4) adrenal insufficiency. Monitoring of adrenal function and hormone levels should continue to ensure appropriate corticosteroid replacement is utilised.
For symptomatic Grade 2 or 3 hypophysitis, nivolumab or nivolumab in combination with ipilimumab should be withheld, and hormone replacement should be initiated as needed. Corticosteroids at a dose of 1 to 2 mg/kg/day methylprednisolone equivalents should also be considered if acute inflammation
of the pituitary gland is suspected. Upon improvement, nivolumab or nivolumab in combination with ipilimumab may be resumed after corticosteroid taper, if needed. Nivolumab or nivolumab in combination with ipilimumab must be permanently discontinued for life-threatening (Grade 4) hypophysitis. Monitoring of pituitary function and hormone levels should continue to ensure
appropriate hormone replacement is utilised.
For symptomatic diabetes, nivolumab or nivolumab in combination with ipilimumab should bewithheld, and insulin replacement should be initiated as needed. Monitoring of blood sugar should continue to ensure appropriate insulin replacement is utilised. Nivolumab or nivolumab in combination with ipilimumab must be permanently discontinued for life-threatening diabetes.

Immune-related skin adverse reactions

Severe rash has been observed with nivolumab in combination with ipilimumab and, less commonly, with nivolumab as monotherapy. Nivolumab or nivolumab in combination with ipilimumab should be withheld for Grade 3 rash and discontinued for Grade 4 rash. Severe rash should be managed with high-dose corticosteroid at a dose of 1 to 2 mg/kg/day methylprednisolone equivalents.
Rare cases of SJS and TEN some of them with fatal outcome have been observed. If symptoms or signs of SJS or TEN appear, treatment with nivolumab or nivolumab in combination with ipilimumab should be discontinued and the patient referred to a specialised unit for assessment and treatment. If the patient has developed SJS or TEN with the use of nivolumab or nivolumab in combination with ipilimumab, permanent discontinuation of treatment is recommended. Caution should be used when considering the use of nivolumab in a patient who has previouslyexperienced a severe or life-threatening skin adverse reaction on prior treatment with other immune-stimulatory anticancer agents.

16.4 Special Populations

Elderly

No dose adjustment is required for elderly patients (≥ 65 years). Data from SCCHN, adjuvant melanoma and first-line RCC patients 75 years of age or older are too limited to draw conclusions on this population.

Renal impairment

Based on the population pharmacokinetic (PK) results, no dose adjustment is required in patients with mild or moderate renal impairment. Data from patients with severe renal impairment are too limited to draw conclusions on this population.

Hepatic impairment

Based on the population PK results, no dose adjustment is required in patients with mild hepatic impairment. Data from patients with moderate or severe hepatic impairment are too limited to draw conclusions on these populations. OPDIVO must be administered with caution in patients with moderate (total bilirubin > 1.5 × to 3 × the upper limit of normal [ULN] and any AST) or severe (total bilirubin > 3 × ULN and any AST) hepatic impairment.

Reproductive Toxicity, Pregnancy

Refer to appendix 1.

16.5 Concomitant use with other medications

Nivolumab is a human monoclonal antibody, as such pharmacokinetic interaction studies have not been conducted. As monoclonal antibodies are not metabolised by cytochrome P450 (CYP) enzymes or other drug metabolising enzymes, inhibition or induction of these enzymes by co-administered medicinal products is not anticipated to affect the pharmacokinetics of nivolumab.

Systemic immunosuppression

The use of systemic corticosteroids and other immunosuppressants at baseline, before starting nivolumab, should be avoided because of their potential interference with the pharmacodynamics activity. However, systemic corticosteroids and other immunosuppressants can be used after starting nivolumab to treat immune-related adverse reactions. The preliminary results show that systemic immunosuppression after starting nivolumab treatment does not appear to preclude the response on nivolumab.

16.6 Overdose

No cases of overdose have been reported in clinical trials. In case of overdose, patients should be closely monitored for signs or symptoms of adverse reactions, and appropriate symptomatic treatment instituted immediately.

17 ALUNBRING (Brigatinib)

Investigators should also consult the local prescribing information if the IMP is approved in your country.

18.1 Standard dose

Alunbrig is administred 90 mg orally once daily for the first 7 days; if tolerated, increase to 180 mg orally once daily. May be taken with or without food. Administer ALUNBRIG until disease progression or unacceptable toxicity. If ALUNBRIG is interrupted for 14 days or longer for reasons other than adverse reactions, resume treatment at 90 mg once daily for 7 days before increasing to the previously tolerated dose. ALUNBRIG may be taken with or without food. Instruct patients to swallow tablets whole. Do not crush or chew tablets. If a dose of ALUNBRIG is missed or vomiting occurs after taking a dose, do not administer an additional dose and take the next dose of ALUNBRIG at the scheduled time.

18.2 Contraindications

Hypersensitivity to the active substance or to any of the excipients.

18.3 Warnings and Precautions

Interstitial Lung Disease (ILD)/Pneumonitis

Occurred in 9.1% of patients at the recommended dose. Monitor for new or worsening respiratory symptoms, particularly during the first week of treatment. Withhold ALUNBRIG for new or worsening respiratory symptoms and promptly
evaluate for ILD/pneumonitis. Upon recovery, either dose reduce or
permanently discontinue ALUNBRIG.

Hypertension

Monitor blood pressure after 2 weeks and then at least monthly during treatment. For severe hypertension, withhold ALUNBRIG, then dose reduce or permanently discontinue.

Bradycardia

Monitor heart rate and blood pressure regularly during treatment. If symptomatic, withhold ALUNBRIG, then dose reduce or permanently discontinue.

Visual Disturbance

Advise patients to report visual symptoms. Withhold ALUNBRIG and obtain ophthalmologic evaluation, then dose reduce or permanently discontinue ALUNBRIG.

Creatine Phosphokinase (CPK) Elevation

Monitor CPK levels regularly during treatment. Based on the severity, withhold ALUNBRIG, then resume or reduce dose.

Pancreatic Enzyme Elevation

Monitor lipase and amylase levels regularly during treatment. Based on the severity, withhold ALUNBRIG, then resume or reduce dose.

Hyperglycemia

Assess fasting serum glucose prior to starting ALUNBRIG and regularly during treatment. If not adequately controlled with optimal medical management, withhold ALUNBRIG, then consider dose reduction or permanently discontinue, based on severity.

Table 1: Recommended ALUNBRIG Dose Reduction Levels

	**Dose**

Dose	Reduction Levels		
	First	Second	Third
90 mg once daily	60 mg once daily	permanently discontinue	N/A*
180 mg once daily	120 mg once daily	90 mg once daily	60 mg once daily

Once reduced for adverse reactions, do not subsequently increase the dose of ALUNBRIG. Permanently discontinue ALUNBRIG if patients are unable to tolerate the 60 mg once daily dose.

Table 2: Recommended ALUNBRIG Dose Modifications for Adverse Reactions

Adverse Reaction	Severity*	Dose Modification
Interstitial Lung Disease (ILD) /Pneumonitis	Grade 1	• If new pulmonary symptoms occur <u>during</u> the first 7 days of treatment, withhold ALUNBRIG until recovery to baseline, then resume at same dose and do not escalate to 180 mg if ILD/pneumonitis is suspected.<br>• If new pulmonary symptoms occur <u>after</u> the first 7 days of treatment, withhold ALUNBRIG until recovery to baseline, then resume at same dose.<br>• If ILD/pneumonitis recurs, permanently discontinue ALUNBRIG.
	Grade 2	• If new pulmonary symptoms occur <u>during</u> the first 7 days of treatment, withhold ALUNBRIG until recovery to baseline. Resume at next lower dose (Table 1) and do not dose escalate if ILD/pneumonitis is suspected.<br>• If new pulmonary symptoms occur <u>after</u> the first 7 days of treatment, withhold ALUNBRIG until recovery to baseline. If ILD/pneumonitis is suspected, resume at next lower dose (Table 1); otherwise, resume at same dose.<br>• If ILD/pneumonitis recurs, permanently discontinue ALUNBRIG.
	Grade 3 or 4	Permanently discontinue ALUNBRIG for ILD/pneumonitis.
Hypertension	Grade 3 hypertension (SBP greater than or equal to 160 mmHg or DBP greater than or equal to 100 mmHg, medical intervention indicated, more than one anti-hypertensive drug, or more intensive	• Withhold ALUNBRIG until hypertension has recovered to Grade 1 or less (SBP less than 140 mmHg and DBP less than 90 mmHg), then resume ALUNBRIG at next lower dose (Table 1).<br>• Recurrence: withhold ALUNBRIG until recovery to Grade 1 or less, and resume at next lower dose (Table 1) <u>or</u> permanently discontinue treatment.

	therapy than previously used indicated)	
	Grade 4 hypertension (life- threatening consequences, urgent intervention indicated)	• Withhold ALUNBRIG until recovery to Grade 1 or less, and resume at next lower dose (Table 1) or permanently discontinue treatment.<br>• Recurrence: permanently discontinue ALUNBRIG for recurrence of Grade 4 hypertension.
Bradycardia (HR less than 60 bpm)	Symptomatic bradycardia	• Withhold ALUNBRIG until recovery to asymptomatic bradycardia or to a resting heart rate of 60 bpm or above.<br>• If a concomitant medication known to cause bradycardia is identified and discontinued or dose-adjusted, resume ALUNBRIG at same dose upon recovery to asymptomatic bradycardia or to resting heart rate of 60 bpm or above.<br>• If no concomitant medication known to cause bradycardia is identified, or if contributing concomitant medications are not discontinued or dose-adjusted, resume ALUNBRIG at next lower dose (Table 1) upon recovery to asymptomatic bradycardia or to resting heart rate of 60 bpm or above.
	Bradycardia with life- threatening consequences, urgent intervention indicated	• Permanently discontinue ALUNBRIG if no contributing concomitant medication is identified.<br>• If contributing concomitant medication is identified and discontinued or dose-adjusted, resume ALUNBRIG at next lower dose (Table 1) upon recovery to asymptomatic bradycardia or to a resting heart rate of 60 bpm or above, with frequent monitoring as clinically indicated.<br>• Recurrence: permanently discontinue ALUNBRIG.
Visual Disturbance	Grade 2 or 3 visual disturbance	Withhold ALUNBRIG until recovery to Grade 1 or baseline, then resume at the next lower dose (Table 1).
	Grade 4 visual disturbance	Permanently discontinue ALUNBRIG.
Creatine	Grade 3 CPK elevation (greater	Withhold ALUNBRIG until recovery to Grade 1 or less (less
Phosphokinase (CPK) Elevation	than 5.0 × ULN)	than or equal to 2.5 × ULN) or to baseline, then resume ALUNBRIG at same dose.

	Grade 4 CPK elevation (greater than 10.0 × ULN) or recurrence of Grade 3 elevation	Withhold ALUNBRIG until recovery to Grade 1 or less (less than or equal to 2.5 × ULN) or to baseline, then resume ALUNBRIG at next lower dose (Table 1).
Lipase/Amylase Elevation	Grade 3 lipase or amylase elevation (greater than 2.0 × ULN)	Withhold ALUNBRIG until recovery to Grade 1 or less (less than or equal to 1.5 × ULN) or to baseline, then resume ALUNBRIG at same dose.
	Grade 4 lipase or amylase elevation (greater than 5.0 x ULN) or recurrence of Grade 3 elevation	Withhold ALUNBRIG until recovery to Grade 1 or less (less than or equal to 1.5 × ULN) or to baseline, then resume ALUNBRIG at next lower dose (Table 1).
Hyperglycemia	Grade 3 (greater than 250 mg/dL or 13.9 mmol/L) or greater	If adequate hyperglycemic control cannot be achieved with optimal medical management, withhold ALUNBRIG until adequate hyperglycemic control is achieved and consider reduction to the next dose (Table 1) or permanently discontinue ALUNBRIG.
Other	Grade 3	• Withhold ALUNBRIG until recovery to baseline, then resume at same dose.<br>• Recurrence: withhold ALUNBRIG until recovery to baseline, then resume at next lower dose or discontinue ALUNBRIG (Table 1).
	Grade 4	• First occurrence: either withhold ALUNBRIG until recovery to baseline and resume at next lower dose (Table 1) or permanently discontinue.<br>• Permanently discontinue ALUNBRIG for recurrence.
bpm = beats per minute; DBP = diastolic blood pressure; HR = heart rate; SBP = systolic blood pressure; ULN = upper limit of normal		

Graded per National Cancer Institute Common Terminology Criteria for Adverse Events. Version 4.0 (NCI CTCAE v4).

18.4 Special Populations

Elderly

Clinical studies of ALUNBRIG did not include sufficient numbers of patients aged 65 years and older to determine whether they respond differently from younger patients. Of the 222 patients in ALTA, 19.4% were 65-74 years and 4.1% were 75 years or older. No clinically relevant differences in safety or efficacy were observed between patients ≥65 years and younger patients.

Renal impairment

No dose adjustment is recommended for patients with mild and moderate renal impairment [creatinine clearance (CLcr) 30 to 89 mL/min estimated by Cockcroft-Gault)]. The pharmacokinetics and safety of ALUNBRIG in patients with severe renal impairment (CLcr 15 to 29 mL/min estimated by Cockcroft-Gault) have not been studied.

Hepatic impairment

No dose adjustment is recommended for patients with mild hepatic impairment (total bilirubin within upper limit of normal [ULN] and AST greater than ULN or total bilirubin greater than 1 and up to 1.5 times ULN

and any AST). The pharmacokinetics and safety of ALUNBRIG in patients with moderate or severe hepatic impairment have not been studied

Reproductive Toxicity, Pregnancy

Refer to appendix 1.

18.5 Concomitant use with other medications

Drugs That May Increase/decrease Brigatinib Plasma Concentrations

Strong CYP3A Inhibitors: Coadministration of itraconazole, a strong CYP3A inhibitor, increased brigatinib plasma concentrations and may result in increased adverse reactions. Avoid the concomitant use of strong CYP3A inhibitors with ALUNBRIG, including but not limited to certain antivirals (e.g., boceprevir, cobicistat, indinavir, lopinavir, nelfinavir, ritonavir, saquinavir), macrolide antibiotics (e.g., clarithromycin), antifungals (e.g., itraconazole, ketoconazole, posaconazole, voriconazole), and conivaptan. Avoid grapefruit or grapefruit juice as it may also increase plasma concentrations of brigatinib. If concomitant use of a strong CYP3A inhibitor cannot be avoided, reduce the dose of ALUNBRIG by approximately 50%.

Strong CYP3A Inducers: Coadministration of ALUNBRIG with rifampin, a strong CYP3A inducer, decreased brigatinib plasma concentrations and may result in decreased efficacy. Avoid the concomitant use of strong CYP3A inducers with ALUNBRIG, including but not limited to rifampin, carbamazepine, phenytoin, and St. John's Wort.

Drugs That May Have Their Plasma Concentrations Altered by Brigatinib

CYP3A Substrates: Brigatinib induces CYP3A in vitro and may decrease concentrations of CYP3A substrates. Coadministration of ALUNBRIG with CYP3A substrates, including hormonal contraceptives, can result in decreased concentrations and loss of efficacy of CYP3A substrates.

18.6 Overdose

No cases of overdose have been reported in clinical trials. In case of overdose, patients should be closely monitored for signs or symptoms of adverse reactions, and appropriate symptomatic treatment instituted immediately.

18 PONATINIB (ICLUSIG)

For this product specific reccomendations were given by the Funder (Incyte)

18.1 Standard dose

The recommended starting dose is 45 mg of ponatinib once daily. For the standard dose of 45 mg once daily, a 45 mg film-coated tablet is available. Treatment should be continued as long as the patient does not show evidence of disease progression or unacceptable toxicity. Patients should be monitored for response according to standard clinical guidelines.

Benefit/risk assessment

Clinical studies were only performed by the company in patients with hematological malignancies.

Compound-specific inclusion criteria

1. Patients must be at least 18 years old.
2. Adequate hepatic function defined as:
    a. Total bilirubin <1.5 × ULN,
    b. Alanine animotransferase (ALT [SGPT]) and aspartate aminotransferase (AST [SGOT]) < 2.5 × ULN for institution (< 5 x ULN if liver involvement with leukemia),
    c. Prothrombin time (PT) < 1.5 × ULN.
3. Normal pancreatic status defined as:
    a. Lipase ≤ 1.5 × ULN,
    b. Amylase ≤ 1.5 × ULN.
4. Normal QTcF interval on screening ECG evaluation, defined as QTcF of ≤ 450 ms in males or ≤ 470 ms in females.
5. Female and male patients who are of childbearing potential must agree to use an effective form of contraception with their sexual partners throughout participation in this study. Hormonal contraception may be susceptible to interaction with ponatinib, which may reduce the efficacy of the hormonal contraception methods. An alternative or additional method of contraception needs to be used with hormonal methods.

Compound-specific exclusion criteria

1. Take medications that are known to be associated with Torsades de Pointes. *(These medications should be listed. We can provide list from PACE protocol : attachment B)*
2. Have significant or active cardiovascular disease, specifically including, but not restricted to:
    a. Myocardial infarction within 3 months prior to first dose of ponatinib,
    b. History of clinically significant atrial arrhythmia or any ventricular arrhythmia,
    c. Unstable angina within 3 months prior to first dose of ponatinib,
    d. Congestive heart failure within 3 months prior to first dose of ponatinib.

3. Have a history of pancreatitis or alcohol abuse.
4. Have uncontrolled hypertriglyceridemia (triglycerides >450 mg/dL).
5. 14. Uncontrolled hypertension (ie >150 and >90 for SBP and DBP respectively). Patients with hypertension should be undertreated at study entry to ensure blood pressure control.
6. 15. Poorly controlled diabetes defined as HbA1c values of >7.5%. Patients with preexisting well controlled diabetes are not excluded.

Study Drug

Baseline risk factors for vascular occlusion events are history of ischaemia, hypertension, diabetes, or hyperlipidaemia.

Based on this analysis, the following supportive care recommendations are provided to decrease the risk of VOEs for patients taking ponatinib.

Diabetes Treatment

Patients with diabetes are at increased risk of experiencing arterial thrombotic events while being treated with ponatinib. Therefore, as a part of the assessment and management of the patient's cardiovascular risk factors, initiation of or modifications to diabetic care should be considered in patients being treated with ponatinib who have elevated glucose levels. The American Diabetes Association guidelines should be followed, and anti-diabetes treatment and lifestyle intervention (including but not limited to weight loss, decreased fat intake, calorie restriction, increased physical activity, and smoking cessation) should be started in any patient with fasting glucose > 130 mg/dL (7.2 mM/L) and/or HbA1c ≥ 7% (Diabetes Prevention Program Research Group, 2002; American Diabetes Association, Position Statement 2013).

Hypertension Treatment

Hypertension (HTN) may contribute to risk of vascular occlusive events. Patients who have HTN should be managed appropriately. During ponatinib treatment, blood pressure elevations should be monitored and managed. Hypertension should be treated to achieve a goal of < 150/90 mmHg. Initial antihypertensive treatment should generally include a thiazide-type diuretic, calcium channel blocker, angiotensin-converting enzyme inhibitor (ACEI), or angiotensin receptor blocker (ARB) (James et al, 2014). Ponatinib treatment should be temporarily interrupted if HTN is not medically controlled (refer to Section "Management of ADRs" below for additional management recommendations). Patients may require urgent clinical intervention for HTN associated with confusion, headache, chest pain, or shortness of breath.

Management of Adverse Drug Reactions

Dose reduction guidelines are outlined in Sections 5 and 6. This section provides additional guidance for management of selected AEs for ponatinib.

Comprehensive assessments of any study drug-related AEs (adverse drug reactions) experienced by the patient will be performed throughout the course of the study. Anticipated adverse drug reactions that may be experienced are described in the Investigator's Brochure. The severity of the event, as well as clinical judgment, will be utilized to determine appropriate management of the patient for any AE experienced while participating in this trial. Any medication, including those administered for therapy of symptoms considered to be associated with study drug administration, should be reported on the appropriate concomitant medication page of the patient's eCRF. The symptoms should be reported on the AE page.

1. Vascular Occlusion

Serious arterial and venous thrombotic and occlusive adverse events, including fatal myocardial infarction, stroke, stenosis of large arterial vessels of the brain, severe peripheral vascular disease, and the need for urgent revascularization procedures have occurred in ponatinib-treated patients. Patients with and without cardiovascular risk factors, including patients age 50 years or younger, experienced these events. Vascular occlusive adverse events were more frequent with increasing age and in patients with prior history of ischemia, hypertension, diabetes, or hyperlipidemia.

a. Arterial Occlusion and Thrombosis

Serious arterial thrombotic adverse events occurred in ponatinib-treated patients with some patients experiencing events of more than one type. Serious cardiovascular thrombotic adverse events included myocardial infarction and coronary artery disease. Some patients developed congestive heart failure concurrent or subsequent to the myocardial ischemic event.

Serious cerebrovascular adverse events were also reported in ponatinib-treated patients. There were patients who developed stenosis of large arterial vessels of the brain (eg, carotid, vertebral, middle cerebral artery).

Serious peripheral arterial adverse events were reported in ponatinib-treated patients. Cases of digital or distal extremity necrosis were reported in patients with diabetes mellitus and peripheral arterial disease; some of these required amputations.

Monitor and aggressively treat factors that increase cardiovascular risk, such as hypertension, cigarette smoking, hypercholesterolemia, and hyperglycemia. Interrupt and consider discontinuation of study drug in patients who develop arterial thrombotic adverse events. Any patient who experiences a serious adverse event of myocardial infarction, stroke, or urgent revascularization while on trial must be discontinued from the trial unless, for that individual patient, the investigator believes the potential benefits of ponatinib treatment are likely to exceed the risks of continued treatment and the patient has no other treatment options.

b. Venous Thromboembolism

Serious venous thromboembolic adverse events occurred in ponatinib-treated patients, including deep venous thrombosis, pulmonary embolism, superficial thrombophlebitis, and retinal vein thrombosis. Consider dose modification or discontinuation of ponatinib in patients who develop serious venous thromboembolic adverse events.

Ponatinib should not be restarted in patients with serious venous occlusive adverse events unless the potential benefit outweighs the risk of recurrent venous occlusions and the patient has no other treatment options.

2. Congestive Heart Failure and Left Ventricular Dysfunction

Severe congestive heart failure (CHF) and left ventricular (LV) dysfunction have been reported in patients taking ponatinib. Patients with cardiac disease or risk factors for cardiac disease should be monitored carefully and any patient with signs or symptoms consistent with cardiac failure should be evaluated and treated. Consider discontinuation of ponatinib in patients who develop serious CHF.

Table. Modifications for Adverse Events (AEs) Attributable to Study Drug

**LVEF/CHF**[1]	
Grade 2	First occurrence at any dose level:<br>Hold until event is ≤ grade 1, or has returned to baseline<br>Resume at current dose level<br><br>Recurrence* at 45 mg:<br>Hold until event is ≤ grade 1, or has returned to baseline<br>Resume at 30 mg<br><br>Recurrence at 30 mg:<br>Hold until event is ≤ grade 1, or has returned to baseline<br>Resume at 15 mg<br><br>Recurrence at 15 mg:<br>Discontinue ponatinib
Grade 3	Occurrence** at 45 mg:<br>Hold until event is ≤ grade 1, or has returned to baseline<br>Resume at 30 mg<br><br>Occurrence at 30 mg:<br>Hold until event is ≤ grade 1, or has returned to baseline<br>Resume at 15 mg<br><br>Occurrence at 15 mg:<br>Discontinue ponatinib
Grade 4	Discontinue ponatinib.

[illegible]

[1]Note: CTCAE criteria should be used to interrupt or discontinue study drug for grade 2, 3, or 4 events considered to be study drug related.

For grade 2: LVEF < 50% - 40%, grade 3: LVEF < 39 - 20%, grade 4: refractory CHF or LVEF < 20%.

3. Hypertension

Blood pressure should be monitored at each visit. Hypertension (HTN) detected by at least 2 blood pressure measurements should be graded according to CTCAE version 4.0, which defines HTN as a disorder characterized by a pathological increase in blood pressure; a repeated elevation in the blood pressure exceeding 140 mm Hg for systolic over 90 mm Hg for diastolic. For patients who develop HTN or worsening HTN during study treatment, aggressive antihypertensive medication should be initiated or optimized to achieve target blood pressure before interruption or dose reduction of the study treatment at the discretion of the investigator. If hypertension is persistent despite adequate anti-hypertensive therapy including titration of anti-hypertensive medication or introduction of additional anti-hypertensive medications, or if grade 4 HTN develops, dose interruption and reduction is recommended according to Dose Modification Guidelines for general non-hematologic AEs in Table below.

4. Cardiac Arrhythmias

Supraventricular tachyarrhythmias were reported in patients treated with ponatinib. Advise patients to report signs and symptoms of rapid heart rate (palpitations, dizziness). Symptomatic bradyarrhythmias

have also been reported. Advise patients to report signs and symptoms suggestive of slow heart rate (fainting, dizziness, or chest pain, see Table below).

5. QT Prolongation

The QT interval-prolonging potential of ponatinib was assessed in 39 leukemia patients, and no clinically significant QT prolongation was observed. This finding was confirmed in ECG observations of 140 patients in the ponatinib arm of the phase 3 trial. However, a thorough QT study has not been performed. Therefore, a clinically significant effect on QT cannot be excluded.

Table. Modifications for Adverse Events (AEs) Attributable to Study Drug

**Prolonged QTcF**	
Grade 2 (QTcF 481-500 ms)	First occurrence at any dose level:<br>Hold ponatinib<br>Perform serum electrolyte analysis (including potassium, calcium and magnesium) and correct with supplements if below normal limits<br>Review concomitant medications<br>Repeat ECG as clinically indicated, but at least daily until QTcF returns to ≤ grade 1 (480 ms)<br>Resume at 45 mg after recovery to ≤ grade 1<br>If no contributing reason was identified for QTcF elevation then weekly ECG monitoring is recommended for 4 weeks upon resumption of ponatinib, then monthly for 6 months, and then every 3 months for the remainder of the study, or more frequently as clinically indicated<br><br>Recurrence at 45 mg:<br>Repeat above<br>Resume at 30 mg after recovery to ≤ grade 1, or until event has returned to baseline<br><br>Recurrence at 30 mg:<br>Repeat above<br>Resume at 15 mg after recovery to ≤ grade 1, or until event has returned to baseline<br><br>Recurrence at 15 mg:<br>Discontinue ponatinib
Grade 3 (QTcF ≥ 501 ms on at least 2 separate ECGs)	First occurrence at any dose level:<br>Hold ponatinib<br>Perform serum electrolyte analysis (including potassium, calcium and magnesium) and correct with supplements if below normal limits<br>Review concomitant medications<br>Repeat ECG as clinically indicated, but at least daily until QTcF returns to ≤ grade 1 (480 ms)<br>Resume at 30 mg after recovery to ≤ grade 1<br>If no contributing reason was identified for QTcF elevation then weekly ECG monitoring is recommended for 4 weeks upon resumption of ponatinib, then monthly for 6 months, and then every 3 months for the remainder of the study, or more frequently as clinically indicated<br><br>Recurrence at 45 mg:<br>Repeat above<br>Resume at 30 mg after recovery to ≤ grade 1, or until event has returned to baseline<br><br>Recurrence at 30 mg:<br>Repeat above<br>Resume at 15 mg after recovery to ≤ grade 1, or until event has returned to baseline<br><br>Recurrence at 15 mg:<br>Discontinue ponatinib
Grade 4	Discontinue ponatinib<br>Consult Sponsor

6. Hemorrhage

Hemorrhagic events have occurred in patients receiving ponatinib. Most hemorrhagic events occurred in patients with grade 4 thrombocytopenia. Interrupt administration in the case of serious or severe hemorrhage.

Table. Modifications for Adverse Events (AEs) Attributable to Study Drug

**Hematologic**	
**ANC/platelets**	
Grade 3 or 4	First occurrence at any dose level:<br>Hold until event is ≤ grade 1, or has returned to baseline<br>Resume at current dose level<br><br>Recurrence at 45 mg:<br>Hold until event is ≤ grade 1, or has returned to baseline<br>Resume at 30 mg<br><br>Recurrence at 30 mg:<br>Hold until event is ≤ grade 1, or has returned to baseline<br>Resume at 15 mg<br><br>Recurrence at 15 mg:<br>Discontinue ponatinib

* "Recurrence" means the second time an AE is encountered by a patient at a given dose level.
** "Occurrence" means the first time an AE is encountered by a patient at a given dose level.
Definitions: ANC = absolute neutrophil count; CHF = congestive heart failure; CT = computed tomography; LVEF = left ventricular ejection fraction.

7. Compromised Wound Healing and Gastrointestinal Perforation

Based on its mechanism of action, ponatinib may compromise wound healing. Interrupt ponatinib for at least 1 week prior to major surgery. The decision when to resume ponatinib after surgery should be based on clinical judgment of adequate wound healing.

8. Neuropathy

Serious peripheral and cranial neuropathic adverse events have occurred in ponatinib-treated patients. In clinical trials, serious peripheral neuropathic adverse events reported included the following: peripheral neuropathy, paresthesia, hypoesthesia, and hyperesthesia. Of the patients who developed neuropathy, many developed neuropathy during the first month of treatment. Monitor patients for symptoms of neuropathy, such as hypoesthesia, hyperesthesia, paresthesia, discomfort, a burning sensation, neuropathic pain, or weakness. Consider interrupting ponatinib and evaluate any suspected neuropathy.

9. Ocular Toxicity

Serious ocular adverse event toxicities leading to blindness or blurred vision have occurred in ponatinib-treated patients. Retinal toxicities including macular edema, retinal vein occlusion, and retinal hemorrhage have also occurred in ponatinib-treated patients. Other ocular toxicities include cataracts, glaucoma, iritis, iridocyclitis, and ulcerative keratitis. Conduct comprehensive eye exams when clinically indicated. See Table below for details.

10. Hepatotoxicity

Hepatotoxicity, most commonly manifested by reversible transaminase and alkaline phosphatase elevation and hyperbilirubinemia, has been observed with ponatinib. Monitoring of hepatic function is recommended and management of laboratory abnormalities should be managed with dose interruption and/or dose reduction according to Table below.

Table. Modifications for Adverse Events (AEs) Attributable to Study Drug

**Hepatic Toxicity**	
Elevation of liver transaminase > 3 × ULN (grade 2 or higher)	Occurrence at 45 mg:<br>Hold and monitor hepatic function until event is ≤ grade 1 (≤3 × ULN), or has returned to baseline<br>Resume at 30 mg<br><br>Occurrence at 30 mg:<br>Hold until event is ≤ grade 1, or has returned to baseline<br>Resume at 15 mg<br><br>Occurrence at 15 mg:<br>Discontinue ponatinib
Elevation of AST or ALT ≥ 3 × ULN concurrent with an elevation of bilirubin > 2 × ULN and alkaline phosphatase < 2 × ULN	Discontinue ponatinib

11. Pancreatitis and Lipase or Amylase Elevations

Pancreatitis (symptomatic abdominal pain associated with pancreatic enzyme elevation) and/or elevations in lipase and amylase are known AEs associated with both ponatinib. Most cases of pancreatitis or elevated pancreatic enzymes occur within the first 2 months of treatment with ponatinib. The events are generally uncomplicated and reversible and can be managed with a brief interruption of treatment and standard medical therapies. Almost all patients are able to continue on with ponatinib treatment at the same or a reduced dose once the event has improved to grade 1 or resolved. Patients with low-grade (1 or 2) elevation in amylase can be continued without dose reduction but should be monitored closely with serial enzyme level determinations. See Table below for details.

Table. Modifications for Adverse Events (AEs) Attributable to Study Drug

**Pancreatitis and Elevation of Lipase**	
Asymptomatic grade 1 or 2 elevation of serum lipase	Consider interruption or dose reduction of ponatinib
Asymptomatic grade 3 or 4 elevation of lipase (> 2 × ULN) or asymptomatic radiologic pancreatitis (grade 2 pancreatitis)	Occurrence at 45 mg:<br>Hold until event is ≤ grade 1 (≤ 1.5 x ULN), or has returned to baseline<br>Resume at 30 mg<br><br>Occurrence at 30 mg:<br>Hold until event is ≤ grade 1, or has returned to baseline<br>Resume at 15 mg<br><br>Occurrence at 15 mg:<br>Discontinue ponatinib
Symptomatic grade 3 pancreatitis (severe pain, vomiting, medical intervention indicated [eg, analgesia, nutritional support])	Occurrence at 45 mg:<br>Hold until complete resolution of symptoms and lipase elevation is ≤ grade 1, or has returned to baseline<br>Resume at 30 mg<br><br>Occurrence at 30 mg:<br>Hold until complete resolution of symptoms and lipase elevation is ≤ grade 1, or has returned to baseline<br>Resume at 15 mg<br><br>Occurrence at 15 mg:<br>Discontinue ponatinib
Grade 4 pancreatitis	Discontinue ponatinib

12. Fluid Retention and Edema

Ponatinib is associated with edema and occasionally serious fluid retention. Patients should be weighed and monitored regularly for signs and symptoms of fluid retention. An unexpected rapid weight gain should be carefully investigated and appropriate treatment provided. Interrupt, reduce the dose of, or discontinue ponatinib as outlined in Table below.

13. Myelosuppression

Neutropenia, anemia, and thrombocytopenia have been observed in clinical studies of ponatinib in patients with CML. While myelosuppression can occur at any time during treatment, its onset in CML patients most commonly occurs within the first month on treatment. Myelosuppression can partially be attributed to the CML itself; however, treatment with ponatinib could also contribute. These events can typically be managed with supportive care and, if felt to be treatment-related, either a reduction or interruption of treatment with ponatinib should occur. Rarely, one or more cytopenias can lead to permanent discontinuation of treatment. The use of hematopoietic growth factors such as granulocyte colony-stimulating factor, and

granulocytemacrophage colony-stimulating factor is permitted on study; these agents may be used to support blood counts as clinically indicated to minimize treatment interruptions or repeated dose reductions. The important clinical AE of febrile neutropenia falls under the broad category of myelosuppression. If a patient's individual risk factors place them at high risk of developing febrile neutropenia, primary prophylactic use of colony-stimulating growth factors for the prevention or reduction of febrile neutropenia is recommended according to the published NCCN guidelines [NCCN Guidelines Version 1, 2012 – Myeloid Growth Factors].

14. Rash and/or Pruritus

Skin rashes have been commonly reported to be associated with ponatinib. The vast majority of the skin events are nonserious, either self-limiting or manageable with antihistamines or topical steroids, and do not result in discontinuation. In more severe cases, a short course of oral corticosteroids may be used until the rash has improved or resolved. In patients treated with ponatinib, the most common skin manifestations are a diffuse maculo-papular rash that is non-pruritic or an acneiform dermititis. Occasionally, patients treated with ponatinib have been reported to have a dry, flaky or exfoliative type of rash or a psoriasiform dermatitis. Rarely, an erythema multiforme type of rash has been associated with ponatinib.

Most patients can be maintained on the current dose of ponatinib, uninterrupted, and if necessary their symptoms can be managed with antihistamines, emollients, or topical steroids. If dose interruption is indicated, patients can resume the same dose of ponatinib typically without recurrence of symptoms once the original episode has improved or resolved. Interrupt administration in the case of serious or severe (grade 3 or 4) rash and follow the dose modification guidelines for non-hematologic toxicity in Table below.

Table. Modifications for Adverse Events (AEs) Attributable to Study Drug

**Skin Rash**	
Grade 2 persistent despite optimal symptomatic therapy	First occurrence at any dose level:<br>Hold until event is ≤ grade 1, or has returned to baseline<br>Resume at current dose level<br><br>Recurrence at 45 mg:<br>Hold until event is ≤ grade 1, or has returned to baseline<br>Resume at 30 mg<br><br>Recurrence at 30 mg:<br>Hold until event is ≤ grade 1, or has returned to baseline<br>Resume at 15 mg<br><br>Recurrence at 15 mg:<br>Discontinue ponatinib
Grade 3 persistent despite optimal symptomatic therapy	First occurrence at any dose level:<br>Hold until event is ≤ grade 1, or has returned to baseline<br>Resume at current dose level<br><br>Recurrence at 45 mg:<br>Hold until event is ≤ grade 1, or has returned to baseline<br>Resume at 30 mg<br><br>Recurrence at 30 mg:<br>Hold until event is ≤ grade 1, or has returned to baseline<br>Resume at 15 mg<br><br>Recurrence at 15 mg:<br>Discontinue ponatinib

Dose Delay and/or Reduction for Adverse Events (AEs) Attributable to the Study Drug

The tables on Modifications for Adverse Events (AEs) Attributable to Study Drug describes guidelines for dose modification due to study-drug-related toxicity, graded according to NCI CTCAE v4.0. These guidelines should be followed by clinical investigators; however, for an individual patient, dose interruptions, reductions and treatment discontinuation should also be based on the clinical circumstance. Deviation from

these guidelines should be documented and communicated with the sponsor. When the observed toxicity has resolved to ≤ grade 1, the investigator may resume full dosing if clinically indicated. There will be no dose modifications for grade 1 or 2 non-hematologic toxicities (except for pancreatitis and QTcF prolongation) attributable to the study drug that are manageable with supportive care or do not interfere with normal daily activities of the patient. In the event of a persistent grade 1 or 2 non-hematologic adverse drug reaction that is 1) intolerable due to clinical symptoms or interferes with normal daily activities, or 2) not controlled by optimal supportive care, the patient may be managed by dose delay or reduction as described in each table. There are no suggested dose modifications for grade 1 or 2 hematologic toxicities. Guidelines for assessment and management of pancreatitis and QTcF prolongation are described in Dose Modification Table also.

Table. Modifications for Adverse Events (AEs) Attributable to Study Drug

**Non-hematologic Toxicity**	
Grade 2<br>Persistent 7 days with optimal care	First occurrence at any dose level:<br>Hold until event is ≤ grade 1, or has returned to baseline<br>Resume at current dose level<br><br>Recurrence* at 45 mg:<br>Hold until event is ≤ grade 1, or has returned to baseline<br>Resume at 30 mg<br><br>Recurrence at 30 mg:<br>Hold until event is ≤ grade 1, or has returned to baseline<br>Resume at 15 mg<br><br>Recurrence at 15 mg:<br>Discontinue ponatinib
Grade 3 or 4	Occurrence** at 45 mg:<br>Hold until event is ≤ grade 1, or has returned to baseline<br>Resume at 30 mg<br><br>Occurrence at 30 mg:<br>Hold until event is ≤ grade 1, or has returned to baseline<br>Resume at 15 mg<br><br>Occurrence at 15 mg:<br>Discontinue ponatinib

18.2 Special Populations

Elderly patients

Of the 449 patients in the clinical study of Iclusig, 155 (35%) were ≥ 65 years of age. Compared to patients < 65 years, older patients are more likely to experience adverse reactions.

Hepatic impairment

Patients with hepatic impairment may receive the recommended starting dose. Caution is recommended when administering Iclusig to patients with hepatic impairment.

Renal impairment

Renal excretion is not a major route of ponatinib elimination. Iclusig has not been studied in patients with renal impairment. Patients with estimated creatinine clearance of ≥ 50 mL/min should be able to safely

receive Iclusig with no dosage adjustment. Caution is recommended when administering Iclusig to patients with estimated creatinine clearance of < 50 mL/min, or end-stage renal disease.

Lactose

This medicinal product contains lactose monohydrate. Patients with rare hereditary problems of galactose intolerance, the Lapp lactase deficiency or glucose-galactose malabsorption should not take this medicinal product.

Effects on ability to drive and use machines

Iclusig has minor influence on the ability to drive and use machines. Adverse reactions such as lethargy, dizziness, and vision blurred have been associated with Iclusig. Therefore, caution should be recommended when driving or operating machines.

Reproductive Toxicity, Pregnancy

Refer to appendix 1.

18.3 Concomitant use with other medications

Substances that may increase ponatinib serum concentrations

CYP3A inhibitors: Ponatinib is metabolized by CYP3A4. Co-administration of a single 15 mg oral dose of Iclusig in the presence of ketoconazole (400 mg daily), a strong CYP3A inhibitor, resulted in modest increases in ponatinib systemic exposure, with ponatinib AUC0-∞ and Cmax values that were 78% and 47% higher, respectively, than those seen when ponatinib was administered alone. Caution should be exercised and a reduction of the starting dose of Iclusig to 30 mg should be considered with concurrent use of strong CYP3A inhibitors such as clarithromycin, indinavir, itraconazole, ketoconazole, nefazodone, nelfinavir, ritonavir, saquinavir, telithromycin, troleandomycin, voriconazole, and grapefruit juice.

Substances that may decrease ponatinib serum concentrations

CYP3A inducers: Co-administration of a single 45 mg dose of Iclusig in the presence of rifampin (600 mg daily), a strong CYP3A inducer, to 19 healthy volunteers, decreased the AUC0-∞ and Cmax of ponatinib by 62% and 42%, respectively, when compared to administration of ponatinib alone. Co-administration of strong CYP3A4 inducers such as carbamazepine, phenobarbital, phenytoin, rifabutin, rifampicin, and St. John's Wort with ponatinib should be avoided, and alternatives to the CYP3A4 inducer should be sought, unless the benefit outweighs the possible risk of ponatinib underexposure.

Substances that may have their serum concentrations altered by ponatinib

Transporter substrates: In vitro, ponatinib is an inhibitor of P-gp and BCRP. Therefore, ponatinib may have the potential to increase plasma concentrations of co-administered substrates of P-gp (e.g., digoxin, dabigatran, colchicine, pravastatin) or BCRP (e.g., methotrexate, rosuvastatin, sulfasalazine) and may increase their therapeutic effect and adverse reactions. Close clinical surveillance is recommended when ponatinib is administered with these medicinal products.

18.4 Overdose

Isolated reports of unintentional overdose with Iclusig were reported in clinical trials. Single doses of 165 mg and an estimated 540 mg in two patients did not result in any clinically significant adverse reactions. Multiple doses of 90 mg per day for 12 days in a patient resulted in pneumonia, systemic inflammatory response, atrial fibrillation, and asymptomatic, moderate pericardial effusion. Treatment was interrupted, the events resolved, and Iclusig was restarted at 45 mg, once daily. In the event of an overdose of Iclusig, the patient should be observed and appropriate supportive treatment given.

19 ITACITINIB

19.1 Standard dose

Itacitinib has been used from 200 mg to 600 mg/die.

19.2 Contraindications

Hypersensitivity to the active substance or to any of the excipients.

19.3 Warnings and Precautions

For this product specific reccomendations were given by the Funder (Incyte)

Benefit/risk assessment

Compound-specific exclusion criteria

1. Presence of an active uncontrolled infection. An active uncontrolled infection is defined as hemodynamic instability attributable to sepsis or new symptoms, worsening physical signs, or radiographic findings attributable to infection. Persisting fever without signs or symptoms will not be interpreted as an active uncontrolled infection.
2. Known human immunodeficiency virus infection.
3. Active HBV or HCV infection that requires treatment, or at risk for HBV reactivation (ie, positive HBsAg). Subjects with negative HbsAg and positive total HB core antibody may be included if HBV DNA is undetectable at the time of screening. Subjects who are positive for HCV antibody are eligible only if polymerase chain reaction test is negative for HCV RNA. Subjects whose immune status is unknown or uncertain must have results confirming immune status before enrollment. Prior serology results are acceptable for determining eligibility.
4. Receipt of live (including attenuated) vaccines or anticipation of need for such a vaccine during the study.

Dose Modifications

Dose interruptions and modifications may occur for individual study subjects based on the emergence or resolution of toxicity.

a) Criteria and Procedures for Dose Interruptions and Adjustments of Study Drug

Treatment with itacitinib may be delayed up to 14 days to allow for resolution of toxicity. After interruption, subjects should be evaluated on a weekly basis until resolution/improvement of the AE. Subjects may resume treatment if no medical condition or other circumstance exists that, in the opinion of the investigator, would make the subject unsuitable for further participation in the study. The investigator should contact the medical monitor to discuss cases where treatment has been delayed for more than 14 days before restarting treatment.

Guidelines for Interruption and Restarting of Itacitinib

ADVERSE EVENT	ACTION TAKEN
**Chemistry**	
• AST and/or ALT > 3.0 × ULN in subjects with normal ALT/AST at baseline.	• **Interrupt** for up to 14 days until the toxicity has resolved to ≤ Grade 1. Exceptions require sponsor approval.<br>• **Restart** at previous dose. If assessed as related to itacitinib/placebo, restart at next lower dose and monitor as clinically indicated.<br>• **NOTE**: In subjects with GVHD-related chemistry elevations at baseline, contact the sponsor medical monitor to discuss clinical management and possible dose reductions.
• Total bilirubin elevations that occur in the presence of GVHD response that cannot be attributed to new liver GVHD or concomitant therapy.	Total bilirubin 3.0-5.0 × ULN:<br>• **Repeat** assessment within 7 days. If elevation persists:<br>– **Reduce** dose by 1 level until bilirubin ≤ 1.5 × ULN.<br>– **Resume** previous dose if resolved in 14 days; if > 14 days, maintain reduced dose.<br>Total bilirubin > 5.0-10.0 × ULN:<br>• **Repeat** assessment within 7 days. If elevation persists:<br>– **Interrupt** until bilirubin ≤ 1.5 × ULN.<br>– **Monitor** LFTs weekly or more frequently as appropriate.<br>– **Resume** previous dose if resolved in 14 days; if > 14 days, resume at reduced dose.<br>Total bilirubin > 10.0 × ULN:<br>• **Repeat** assessment within 7 days. If elevation persists:<br>– **Interrupt** until bilirubin ≤ 1.5 × ULN.<br>– **Resume** at reduced dose if resolved in 14 days; if > 14 days, discontinue treatment and monitor as appropriate.
• Total bilirubin elevations that occur in subjects with Stage 1/2 liver GVHD that cannot be attributed to worsening liver GVHD or concomitant therapy.	Total bilirubin > 3.0 × ULN:<br>• **Repeat** assessment within 7 days. If elevation persists:<br>– **Reduce** dose by 1 dose level.<br>– **Resume** previous dose if bilirubin ≤ 3.0 × ULN.
**Hematology**	
• ANC < 0.5 × 10^9/L, suspected as unrelated to study treatment (eg, GVHD, active cytomegalovirus viremia).	• **Reduce** dose by 1 dose level.<br>• **Monitor** ANC count as clinically indicated.<br>• **Resume** previous dose if ANC count is ≥ 0.5 × 10^9/L for more than 7 days.
• ANC < 0.5 × 10^9/L, suspected as related to study treatment.	• **Interrupt** for up to 14 days.<br>• **Monitor** ANC count as clinically indicated.<br>• **Resume** at a reduced dose if ANC count is ≥ 0.5 × 10^9/L for more than 7 days. If the subject's ANC count remains at ≥ 0.5 × 10^9/L for more than 7 days after resuming treatment at a lower dose, the previous dose may be resumed.

ADVERSE EVENT	ACTION TAKEN
• Platelet count is < 10 × 10⁹/L, or platelet count has decreased by ≥ 50% from baseline, suspected as unrelated to study treatment.	• **Reduce** dose by 1 dose level.<br>• **Monitor** platelet count as clinically indicated.<br>• **Resume** at previous dose if platelet count returns to ≥ 20 × 10⁹/L or within 75% of baseline for more than 7 days.
• Platelet count is < 10 × 10⁹/L, or platelet count has decreased by ≥ 50% from baseline, suspected as related to study treatment.	• **Interrupt** for up to 14 days.<br>• **Monitor** platelet count as clinically indicated.<br>• **Resume** at a reduced dose if platelet count returns to ≥ 20 × 10⁹/L or within 75% of baseline for more than 7 days. If the subject's platelet count remains stable for an additional 7 days, the previous dose of may be resumed.
**Other toxicities**	
• Any Grade 1 or Grade 2 toxicity.	• **Continue** treatment and manage the toxicity.<br>• **Monitor** as clinically indicated.
• Any Grade 3 toxicity, if clinically significant and not manageable by supportive care.	• **Interrupt** up to 14 days until toxicity resolves to ≤ Grade 1.<br>• **Restart** at same dose; if assessed as related to itacitinib/placebo, restart at next lower dose and monitor as clinically indicated.
• Any recurrent Grade 3 toxicity at 100 mg QD dose.	• **Discontinue** study treatment; follow-up per Protocol. Exceptions require sponsor approval.
• Any other Grade 4 toxicity.	• **Discontinue** study treatment; follow-up per Protocol.

ALT = alanine aminotransferase; ANC = absolute neutrophil count; AST = aspartate aminotransferase; ULN = upper limit of normal.

19.4 Special Populations

Reproductive Toxicity, Pregnancy

Refer to appendix 1.

19.5 Concomitant use with other medications

Coadministration of a strong CYP3A4 inhibitor (itraconazole) increased itacitinib exposure in healthy participants by approximately 5-fold. Concomitant use of strong CYP3A inhibitors should be avoided if clinically feasible. If coadministration of itacitinib with a strong CYP3A inhibitor cannot be avoided, use of an agent with less CYP3A4 inhibition in a class, for example, posaconazole for fungal infection, or prophylaxis is recommended over other choices like voriconazole or itraconazole. Careful monitoring of hematology parameters and clinical signs and symptoms of itacitinib-related adverse reactions is recommended upon initiation of a strong CYP3A4 inhibitor. Participants receiving itacitinib should avoid pomegranates, pomegranate juice, grapefruit, and grapefruit juice, all of which are known to inhibit CYP3A enzymes and may increase the exposure to itacitinib.

Coadministration of a strong CYP3A4 inducer (rifampin) decreased the plasma exposure of itacitinib in healthy participants by approximately 80%. Concomitant use of strong CYP3A inducers (such as, but not limited to, phenytoin, rifampin, carbamazepine, and St John's Wort (Hypericum perforatum) should be avoided.

19.6 Overdose

The highest total daily dose evaluated in clinical studies was 1200 mg (600 mg BID) in the multiple-dose and renal function healthy participant studies. No unexpected events were associated with doses of this level. Treatment of suspected overdose with itacitinib should consist of general supportive measures.

20 PEMIGATINIB

20.1 Standard dose

The recommended dosage of pemigatinib is 13.5 mg orally once daily for 14 consecutive days followed by 7 days off therapy, in 21-day cycles. Continue treatment until disease progression or unacceptable toxicity occurs. Take pemigatinib with or without food at approximately the same time every day. Swallow tablets whole. Do not crush, chew, split, or dissolve tablets. If the patient misses a dose of pemigatinib by 4 or more hours or if vomiting occurs, resume
dosing with the next scheduled dose.

20.2 Contraindications

Hypersensitivity to the active substance or to any of the excipients.

20.3 Warnings and Precautions

For this product specific reccomendations were given by the Funder (Incyte)

Benefit/risk assessment
Compound-specific inclusion criteria

1. FGFR mutations/fusions

Compound-specific exclusion criteria

1. Prior receipt of a selective FGFR inhibitor.
2. Use of any potent CYP3A4 inhibitors or inducers or moderate CYP3A4 inducers within 14 days or five half-lives (whichever is longer) before the first dose of study drug/treatment.
3. Current evidence of clinically significant corneal (including but not limited to bullous/band keratopathy, corneal abrasion, inflammation/ulceration, and keratoconjunctivitis) or retinal disorder (including but not limited to central serous retinopathy, macular/retinal degeneration, diabetic retinopathy, retinal detachment) as confirmed by ophthalmologic examination
4. History of calcium and phosphate hemostasis disorder or systemic mineral imbalance with ectopic calcification of soft tissues (exception: commonly observed calcifications in soft tissues, such as the skin, kidney, tendons or vessels due to injury, disease, and aging, in the absence of systemic mineral imbalance).
5. Serum Calcium: Outside of normal range or serum albumin-corrected calcium outside of the normal range when serum albumin is outside of the normal range
6. Serum Phosphate: > ULN of the institution
7. Patients with VUS alterations as they are usually not driver and are of unknown significance?
    a. These were just recommendations based on what we have observed in our Phase I studies in terms of prevalence of FGFR alterations and which ones seem to enrich for response to FGFRi (fusions > mutations > amplifications). Since we don’t know the oncogenic potency of VUS, it is recommended not to include these. However, these are not mandatory and investigator is free to include these or not, depending on the goal of the study and the FGFR alterations they are including.

Treatment requirements/conditions/limitations

Phosphate should be monitored on C1D1, C1D8 and C1D15 to assess presence of Hyperphosphatemia. Hyperphosphatemia in Cycle 1 requires Day 8 testing of serum phosphate in Cycle ≥2 until phosphate is < 7 mg/dL on Day 8 for at least 2 consecutive cycles on stable dose of binders (please see below).

- Please see recommendations for management of Hyperphosphatemia:
    - Hyperphosphatemia is an expected on-target pharmacologic effect of FGFR inhibition. Hyperphosphatemia should be managed with diet modifications, phosphate binders and diuretics, or a dose modifications. The use of diet modifications alone include food exchanges from high-phosphate foods to low-phosphate foods and can be implemented once serum phosphate levels are above the ULN but do not exceed 7.0 mg/dL.

- Diet modification should continue with the inclusion of phosphate binders once serum phosphate levels exceed 7.0 mg/dL. Examples of phosphate binders are sevelamer HCl (examples of name brands: Renegel® or Renvela®); administration of phosphate binders should be 3 times per day (eg, with each meal) to reduce absorption of phosphate. Doses and frequency of doses must be based on the participant's tolerance for the binder and the control of the serum phosphate.
- If binders are used to manage hyperphosphatemia during treatment, it is recommended to stop binders at the same time pemigatinib is stopped to reduce the risk of hypophosphatemia.
  See Table Below.

Recommended Approach for Hyperphosphatemia Management

**Serum Phosphate Level**	**Supportive Care**	**Guidance for Interruption/Discontinuation of Pemigatinib**	**Guidance for Restarting Pemigatinib**
> 5.5 mg/dL and ≤ 7 mg/dL	Initiate a low-phosphate diet	No action.	Not applicable.
> 7 mg/dL and ≤ 10 mg/dL	Initiate/continue a low-phosphate diet and initiate phosphate binding therapy. Monitor serum phosphate approximately twice a week and adjust the dose of binders as needed; continue to monitor serum phosphate at least twice a week until return to ≤ 7 mg/dL.	If serum phosphate level continues to be > 7 mg/dL and ≤ 10 mg/dL with concomitant phosphate-binding therapy for 2 weeks, or if there is recurrence of serum phosphate level in this range, *interrupt* pemigatinib for up to 2 weeks	Restart at the same dose when serum phosphate is < 7 mg/dL. If serum phosphate level recurs at > 7 mg/dL, restart pemigatinib with dose reduction.
> 10 mg/dL	Continue to maintain a low-phosphate diet, adjust phosphate- binding therapy, and start/continue phosphaturic agent. Continue to monitor serum phosphate approximately twice a week until return to ≤ 7 mg/dL.	If serum phosphate level is > 10 mg/dL for 1 week following phosphate-binding therapy and low-phosphate diet, *interrupt* pemigatinib. If there is recurrence of serum phosphate level in this range following 2 dose reductions, *permanently discontinue* pemigatinib.	Restart pemigatinib at reduced dose with phosphate binders when serum phosphate is < 7 mg/dL.

Comprehensive Eye Examination

Due to the infrequent but potential serious adverse event of retinal pigmented epithelium detachment (RPED) seen with FGFR inhibitors (<5% across pemigatinib studies), a comprehensive eye examination at screening and during treatment is required.

Most cases of RPED reported are self-limiting, frequently without treatment discontinuation. Nevertheless, subjects should be monitored for signs and symptoms of serous retinal detachment, and the retina should be assessed with optical coherence tomography, if indicated.
Mild and moderate intensity events should be monitored with ophthalmology examination, without treatment interruptions, and, in case of worsening of vision and/or in severe (Grade 3) cases of serous retinal detachment, INCB054828 treatment should be interrupted.
Please see recommendation of monitoring below:
A comprehensive eye examination should be performed by a qualified ophthalmologist at screening, once every 3 cycles (± 7 days, starting at Cycle 3), at EOT, and as clinically indicated. The eye examination should include a visual acuity test, slit-lamp examination, and funduscopy with digital imaging. Additional assessments (eg, OCT) should be performed if clinically relevant retinal findings are observed on ophthalmologic exams and in participants with reported visual AEs or change in visual acuity, if the events or changes are suspected to be of retinal origin. Every effort should be made to ensure that all subsequent examinations are performed by the same ophthalmologist.

The recommended dose reductions for adverse reactions are provided in Table 1

Table 1: Recommended Dose Reductions for Pemigatinib for Adverse Reactions

**Dose Reduction**	**Recommended Dosage**
First	9 mg once daily for first 14 days of each 21-day cycle
Second*	4.5 mg once daily for first 14 days of each 21-day cycle

Permanently discontinue Pemigatinib if unable to tolerate 4.5 mg once daily.

Table 2: Recommended Dosage Modifications for pemigatinib Adverse Reactions

**Adverse Reaction**	**Severity***	**Pemigatinib Dosage Modification**
Retinal Pigment Epithelial Detachment (RPED)	RPED	• If asymptomatic and stable on serial examination, continue pemigatinib.<br>• If symptomatic or worsening on serial examination, withhold pemigatinib.<br>• If asymptomatic and improved on subsequent examination, resume pemigatinib at a lower dose.<br>• If symptoms persist or examination does not improve, consider permanent discontinuation of pemigatinib, based on clinical status.

Hyperphosphatemia	Serum phosphate > 7 mg/dL- ≤10 mg/dL	• Initiate phosphate lowering therapy and monitor serum phosphate weekly.<br>• Withhold pemigatinib if levels are not < 7 mg/dL within 2 weeks of starting phosphate lowering therapy.<br>• Resume pemigatinib at the same dose when phosphate levels are < 7 mg/dL for first occurrence; resume at a lower dose level for subsequent recurrences.
	Serum phosphate >10 mg/dL	• Initiate phosphate lowering therapy and monitor serum phosphate weekly.<br>• Withhold pemigatinib if levels are not ≤ 10 mg/dL within 1 week after starting phosphate lowering therapy.<br>• Resume pemigatinib at the next lower dose level when phosphate levels are < 7 mg/dL.
		• Permanently discontinue pemigatinib for recurrence of serum phosphate > 10mg/dL following 2 dose reductions.
Other Adverse Reactions	Grade 3	• Withhold pemigatinib until resolves to Grade 1 or baseline.<br>• Resume pemigatinib at next lower dose if resolves within 2 weeks.<br>• Permanently discontinue pemigatinib if does not resolve within 2 weeks.<br>• Permanently discontinue pemigatinib for recurrent Grade 3 after 2 dose reductions.
	Grade 4	• Permanently discontinue pemigatinib.

Severity as defined by National Cancer Institute Common Terminology Criteria for Adverse Events (NCI CTCAE) version 4.03.

20.4 Special Populations

Elderly

In FIGHT-202, 32% of patients were 65 years and older, and 8% of patients were 75 years and older. No overall differences in safety or effectiveness were observed between these patients and younger patients.

Renal impairment

No dose adjustment is recommended for patients with mild or moderate renal impairment (glomerular filtration rate (GFR) ≥ 30 to <90 mL/min estimated by Modification of Diet in Renal Disease (MDRD) equation). The recommended dose of pemigatinib has not been established for patients with severe renal impairment (GFR <30 mL/min)

Hepatic impairment

No dose adjustment is recommended for patients with mild (total bilirubin > upper limit of normal (ULN) to 1.5 × ULN or AST > ULN) or moderate hepatic impairment (total bilirubin>1.5–3 × ULN with any AST). The recommended dose of pemigatinib has not been established for patients with severe hepatic impairment (total bilirubin >3 × ULN with any AST)

Reproductive Toxicity, Pregnancy

Refer to appendix 1.

20.5 Concomitant use with other medications

Avoid concomitant use of strong and moderate CYP3A inhibitors with pemigatinib. If concomitant use with a strong or moderate CYP3A inhibitor cannot be avoided:

- Reduce pemigatinib dose from 13.5 mg to 9 mg.
- Reduce pemigatinib dose from 9 mg to 4.5 mg.

If concomitant use of a strong or moderate CYP3A inhibitor is discontinued, increase the pemigatinib dose (after 3 plasma half-lives of the CYP3A inhibitor) to the dose that was used before starting the strong inhibitor.

20.6 Overdose

No data on overdose.

21 PIQRAY (Alpelisib)

Posology and method of administration

Treatment with Piqray should be initiated by a physician experienced in the use of anticancer therapies.

Posology

The recommended dose is 300 mg alpelisib (2x 150 mg film-coated tablets) taken once daily on a continuous basis. Piqray should be taken immediately after food, at approximately the same time each day (see section 5.2). The maximum recommended daily dose of Piqray is 300 mg.
If a dose of Piqray is missed, it can be taken immediately following food and within 9 hours after the time it is usually administered. After more than 9 hours, the dose should be skipped for that day. On the next day, Piqray should be taken at the usual time. If the patient vomits after taking the Piqray dose, the patient should not take an additional dose on that day and should resume the usual dosing schedule the next day at the usual time.
Piqray should be co-administered with fulvestrant. The recommended dose of fulvestrant is 500 mg administered intramuscularly on days 1, 15 and 29, and once monthly thereafter. Please refer to the full prescribing information of fulvestrant.
Treatment should continue as long as clinical benefit is observed or until unacceptable toxicity occurs. Dose modifications may be necessary to improve tolerability.
Dose modifications
Management of severe or intolerable adverse drug reactions (ADRs) may require temporary dose interruption, reduction, and/or discontinuation of Piqray. If dose reduction is required, the dose reduction guidelines for ADRs are listed in Table 1. A maximum of 2 dose reductions are recommended, after which the patient should be permanently discontinued from treatment with Piqray. Dose reduction should be based on the worst preceding toxicity.

**Table 1 Recommended dose reduction guidelines for ADRs for Piqray1 Piqray dose level**	**Dose and schedule**	**Number and strength of tablets**
Starting dose	300 mg/day continuously	2x 150 mg tablets
First dose reduction	250 mg/day continuously	1x 200 mg tablet and 1x 50 mg tablet
Second dose reduction	200 mg/day continuously	1x 200 mg tablet
1 Only one dose reduction is permitted for pancreatitis.		

Tables 2-5 summarise the recommendations for dose interruption, reduction or discontinuation of Piqray in the management of specific ADRs. The clinical judgement of the treating physician, including confirmation of laboratory values if deemed necessary, should guide the management plan of each patient based on the individual benefit/risk assessment for treatment with Piqray.

Hyperglycaemia

- **Table 2 Dose modification and management for hyperglycaemia[1]**

**Fasting glucose values[1]**	**Recommendation**
**Dose reductions should only be based on fasting glucose (plasma/blood) values.**	

	Consultation with a healthcare professional experienced in the treatment of hyperglycaemia should always be considered and is recommended for patients who are pre-diabetic or those with fasting glucose (FG) >250 mg/dl or 13.9 mmol/l, body mass index (BMI) ≥30 or age ≥75 years.<br>Consultation with a diabetologist or a healthcare professional experienced in the treatment of hyperglycaemia should always take place for patients with diabetes.<br>All patients should be instructed on lifestyle changes that may reduce hyperglycaemia (e.g. dietary restrictions and physical activity).
>ULN-160 mg/dl or<br>>ULN-8.9 mmol/l	No Piqray dose adjustment required.<br>Initiate or intensify oral antidiabetic treatment[2].
>160-250 mg/dl or<br>>8.9-13.9 mmol/l	No Piqray dose adjustment required.<br>Initiate or further intensify oral antidiabetic treatment[2].<br>If FG does not decrease to ≤160 mg/dl or 8.9 mmol/l within 21 days with appropriate oral antidiabetic treatment[2,3], reduce Piqray dose by 1 dose level and follow FG-value-specific recommendations.
>250-500 mg/dl or<br>>13.9-27.8 mmol/l	Interrupt Piqray.<br>Initiate or intensify oral antidiabetic treatment[2] and consider additional antidiabetic medicinal products (such as insulin[3]) for 1-2 days until hyperglycaemia resolves.<br>Administer intravenous hydration and consider appropriate treatment (e.g. intervention for electrolyte / ketoacidosis / hyperosmolar disturbances).<br>If FG decreases to ≤160 mg/dl or 8.9 mmol/l within 3 to 5 days under appropriate antidiabetic treatment, resume Piqray at next lower dose level.<br>If FG does not decrease to ≤160 mg/dl or 8.9 mmol/l within 3 to 5 days under appropriate antidiabetic treatment, consultation with a healthcare professional with expertise in the treatment of hyperglycaemia is recommended.<br>If FG does not decrease to ≤160 mg/dl or 8.9 mmol/l within 21 days following appropriate antidiabetic treatment[2,3], permanently discontinue Piqray treatment.
500 mg/dl or<br>≥27.8 mmol/l	Interrupt Piqray.<br>Initiate or intensify appropriate antidiabetic treatment[2,3] (administer intravenous hydration and consider appropriate treatment [e.g. intervention for electrolyte / ketoacidosis / hyperosmolar disturbances]), re-check within 24 hours and as clinically indicated.<br>If FG decreases to ≤500 mg/dl or ≤27.8 mmol/l, then follow FG-value-specific recommendations for <500 mg/dl.<br>If FG is confirmed at >500 mg/dl or ≥27.8 mmol/l after 24 hours, permanently discontinue Piqray treatment.

1	Fasting glucose levels reflect hyperglycaemia grading according to CTCAE Version 4.03 CTCAE = Common Terminology Criteria for Adverse Events.
2	Applicable antidiabetic medicinal products should be initiated and the respective prescribing information should be reviewed for dosing and dose titration recommendations, including local diabetic treatment guidelines. Metformin was recommended in the phase III clinical study with the following guidance: Metformin should be initiated at 500 mg once daily. Based on tolerability, the metformin dose may be increased to 500 mg twice daily, followed by 500 mg with breakfast, and 1000 mg with the evening meal, followed by further increase to 1000 mg twice daily if needed (see section 4.4).
3	As recommended in the phase III clinical study, insulin may be used for 1-2 days until hyperglycaemiaresolves. However, this may not be necessary in the majority of cases of alpelisib-induced hyperglycaemia, given the short half-life of alpelisib and the expectation that glucose levels will normalise following interruption of Piqray.

Baseline diabetic and pre-diabetic status, baseline BMI ≥30 and baseline age ≥75 years have been found to be risk factors for hyperglycaemia in patients treated with alpelisib. These risk factors werepresent in 74.7% of patients with any grade of hyperglycaemia and in 86.2% of patients with grade 3or 4 hyperglycaemia

Rash
Oral antihistamine administration may be considered prophylactically, at the time of initiation of treatment with Piqray. Additionally, antihistamines are recommended to manage symptoms of rash.

Topical corticosteroid treatment should be initiated at the first signs of rash and oral corticosteroids should be considered for moderate to severe rashes. Based on the severity of rash, Piqray may requiredose interruption, reduction or discontinuation as described in Table 3.

- **Table 3 Dose modification and management for rash[1]**

**Grade**	**Recommendation**
All grades	Consultation with a dermatologist should always be considered.
Grade 1<br>(<10% body surface area [BSA] with active skin toxicity)	No Piqray dose adjustment required.<br>Initiate topical corticosteroid treatment.<br>Consider adding oral antihistamine treatment to manage symptoms.
Grade 2<br>(10-30% BSA with active skin toxicity)	No Piqray dose adjustment required.<br>Initiate or intensify topical corticosteroid and oral antihistamine treatment.<br>Consider low-dose oral corticosteroid treatment.
Grade 3 (e.g. severe rash not responsive to medical management)<br>(>30% BSA with active skin toxicity)	Interrupt Piqray until rash is grade ≤1.<br>Initiate or intensify topical/oral corticosteroid and antihistamine treatment.<br>Once improved to grade ≤1, then resume Piqray at the same dose level for first occurrence of rash and at next lower dose level, in case of second occurrence.

Grade 4 (e.g. severe bullous, blistering or exfoliating skin conditions) (any % BSA associated with extensive superinfection, with intravenous antibiotics indicated; life-threatening consequences)	Permanently discontinue Piqray.
[1] Grading according to CTCAE Version 5.0	

Diarrhoea

- **Table 4 Dose modification and management for diarrhoea**

Grade[1]	Recommendation
Grade 1	No Piqray dose adjustment is required. Initiate appropriate medical therapy and monitor as clinically indicated.
Grade 2	Initiate or intensify appropriate medical therapy and monitor as clinically indicated. Interrupt Piqray dose until recovery to grade ≤1, then resume Piqray at same dose level.
Grade 3 or 4[2]	Initiate or intensify appropriate medical therapy and monitor as clinically indicated. Interrupt Piqray dose until recovery to grade ≤1, then resume Piqray at the next lower dose level.
[1] Grading according to CTCAE Version 5.0. [2] Patients should additionally be managed according to local standard of care, including electrolyte monitoring, administration of antiemetics and antidiarrhoeal medicinal products and/or fluid replacement and electrolyte supplements, as clinically indicated.	

Other toxicities

- **Table 5 Dose modification and management for other toxicities (excluding hyperglycaemia,rash and diarrhoea)[1]**

Grade	Recommendation
Grade 1 or 2	No Piqray dose adjustment required. Initiate appropriate medical therapy and monitor as clinically indicated[2,3].
Grade 3	Interrupt Piqray dose until improvement to grade ≤1, then resume Piqray at the next lower dose level[2].
Grade 4	Permanently discontinue Piqray[3].
[1] Grading according to CTCAE Version 5.0 [2] For grade 2 and 3 pancreatitis, interrupt Piqray dose until recovery to grade ≤1 and resume at next lower dose level. Only one dose reduction is permitted. If toxicity recurs, permanently discontinuePiqray treatment. [3] For grade 2 total bilirubin elevation, interrupt Piqray dose until recovery to grade ≤1 and resume at the same dose if resolved in ≤14 days or resume at the next lower dose level if resolved in >14 days.	

<u>*Special populations*</u>

Elderly

No dose regimen adjustment is required in patients aged 65 years or above (see section 5.2). There arelimited data in patients aged ≥75 years, and especially for those ≥85 years.

Renal impairment
Based on population pharmacokinetic analysis, no dose adjustment is necessary in patients with mildor moderate renal impairment (see section 5.2). Caution should be used in patients with severe renal impairment as there is no experience with Piqray in this population.

Hepatic impairment
Based on a hepatic impairment study in non-cancer subjects with impaired hepatic function, no doseadjustment is necessary in patients with mild, moderate or severe hepatic impairment (Child-Pugh class A, B or C, respectively) (see section 5.2).

Paediatric population
The safety and efficacy of Piqray in children aged 0-18 years have not been established. No data areavailable.

<u>Method of administration</u>

Piqray is for oral use. The tablets should be swallowed whole. They should not be chewed, crushed orsplit prior to swallowing. Tablets that are broken, cracked or otherwise not intact should not be ingested.

All patients should be instructed on lifestyle changes that may reduce hyperglycaemia (e.g. dietaryrestrictions).

Table 6 Schedule of fasting glucose monitoring

	**Recommended schedule for the monitoring of fasting glucose and HbA1c levels in all patients treated with Piqray**	**Recommended schedule of monitoring of fasting glucose and HbA1c levels in patients with diabetes, pre-diabetes, BMI ≥30 or age ≥75 years treated with Piqray**
**At screening, before initiating treatment with Piqray**	Test for fasting plasma glucose (FPG), HbA1c, and optimise the patient's level of blood glucose (see Table 2).	
**After initiating treatment with Piqray**	Monitor FPG at weeks 1, 2, 4, 6 and 8 after treatment start and monthly thereafter.	
	Monitor/self-monitor fasting glucose regularly, more frequently in the first 4 weeks and especially within the first 2 weeks of treatment, according to the instructions of a healthcare professional*.	Monitor/self-monitor fasting glucose daily for the first 2 weeks of treatment. Then continue to monitor fasting glucose as frequently as needed to manage hyperglycaemia according to the instructions of a healthcare professional*.
	HbA1c should be monitored after 4 weeks of treatment and every 3 months thereafter.	
**If hyperglycaemia develops after initiating treatment**	Monitor fasting glucose regularly, as per local standard of care and at least until fasting glucose decreases to normal levels.	

**with Piqray**	During treatment with antidiabetic medication, continue monitoring fasting glucose at least once a week for 8 weeks, followed by once every 2 weeks, and monitor fasting glucose according to the instructions of a healthcare professional with expertise in the treatment of hyperglycaemia.
* All glucose monitoring should be performed at the physician's discretion as clinically indicated.	

Patients should be advised of the signs and symptoms of hyperglycaemia (e.g. excessive thirst, urinating more often than usual or greater amount of urine than usual, increased appetite with weightloss).

Interaction with other medicinal products and other forms of interaction

Medicinal products that may increase alpelisib plasma concentrations

BCRP inhibitors
Alpelisib is a substrate for BCRP *in vitro*. BCRP is involved in the hepatobiliary export and intestinalsecretion of alpelisib, therefore inhibition of BCRP in the liver and in the intestine during eliminationmay lead to an increase in systemic exposure of alpelisib. Therefore, caution and monitoring for toxicity are advised during concomitant treatment with inhibibitors of BCRP (e.g. eltrombopag, lapatinib, pantoprazole).

Medicinal products that may decrease alpelisib plasma concentrations

Acid-reducing agents
The co-administration of the H2 receptor antagonist ranitidine in combination with a single 300 mg oral dose of alpelisib slightly reduced the bioavailability of alpelisib and decreased overall exposure ofalpelisib. In the presence of a low-fat low-calorie (LFLC) meal, AUC_{inf} was decreased on average by 21% and C_{max} by 36% with ranitidine. In the absence of food, the effect was more pronounced with a 30% decrease in AUC_{inf} and a 51% decrease in C_{max} with ranitidine compared to the fasted state without co-administration of ranitidine. Population pharmacokinetic analysis showed no significant effect of co-administration of acid-reducing agents, including proton pump inhibitors, H2 receptor antagonists and antacids, on the pharmacokinetics of alpelisib. Therefore, alpelisib can be co-administered with acid-reducing agents, provided alpelisib is taken immediately after food (seesection 4.2).

Medicinal products whose plasma concentrations may be altered by alpelisib

Based on the results of metabolic *in vitro* induction and inhibition studies, alpelisib may induce the metabolic clearance of co-administered medicinal products metabolised by CYP2B6, CYP2C9 and CYP3A and may inhibit the metabolic clearance of co-administered medicinal products metabolisedby CYP2C8, CYP2C9, CYP2C19 and CYP3A4 (time-dependent inhibition) if sufficiently high concentrations are achieved *in vivo*.

CYP3A4 substrates
No dose adjustment is required when co-administering Piqray with CYP3A4 substrates (e.g.everolimus, midazolam).

In a drug-drug interaction study, co-administration of alpelisib with everolimus, a sensitive CYP3A4substrate, confirmed that there are no clinically significant pharmacokinetic interactions (increase in AUC by 11.2%) between alpelisib and CYP3A4 substrates. No change in everolimus exposure was observed at alpelisib doses ranging from 250 to 300 mg.

Caution is recommended when Piqray is used in combination with CYP3A4 substrates that also possess an additional time-dependent inhibition and induction potential on CYP3A4 that affects theirown metabolism (e.g. rifampicin, ribociclib, encorafenib).

CYP2C9 substrates with narrow therapeutic index
In the absence of clinical data on CYP2C9, caution is recommended. *In vitro* evaluations indicatedthat the pharmacological activity of CYP2C9 substrates with a narrow therapeutic index such as warfarin may be reduced by the CYP2C9 induction effects of alpelisib.

CYP2B6 sensitive substrates with narrow therapeutic index
Sensitive CYP2B6 substrates (e.g. bupropion) or CYP2B6 substrates with a narrow therapeutic window should be used with caution in combination with Piqray, as alpelisib may reduce the clinicalactivity of such medicinal products.

Substances that are substrates of transporters
In vitro evaluations indicated that alpelisib (and/or its metabolite BZG791) has a potential to inhibitthe activities of OAT3 drug transporters and intestinal BCRP and P-gp. Piqray should be used with caution in combination with sensitive substrates of these transporters which exhibit a narrow therapeutic index because Piqray may increase the systemic exposure of these substrates.

Hormonal contraceptives

No clinical studies were conducted assessing the drug-drug-interaction potential between alpelisib andhormonal contraceptives.

ADRs observed in phase III clinical study and during post-marketing experience

**Adverse drug reaction**	**Any grade (%)**		**Grade 3 or 4 (%)**
**Infections and infestations**			
Urinary tract infection[1]	Very common	29 (10.2)	2 (0.7)*
**Blood and lymphatic system disorders**			
Anaemia	Very common	125 (44.0)	14 (4.9)*
Lymphocyte count decreased	Very common	157 (55.3)	26 (9.2)
Platelet count decreased	Very common	43 (15.1)	4 (1.4)*
**Immune system disorders**			
Hypersensitivity[2]	Common	11 (3.9)	2 (0.7)*
**Metabolism and nutrition disorders**			
Glucose plasma increased	Very common	225 (79.2)	111 (39.1)
Glucose plasma decreased	Very common	76 (26.8)	1 (0.4)
Decreased appetite	Very common	102 (35.9)	2 (0.7)*
Hypokalaemia	Very common	42 (14.8)	18 (6.3)

Hypocalcaemia	Very common	79 (27.8)	6 (2.1)
Magnesium decreased	Very common	34 (12.0)	1 (0.4)
Dehydration	Common	10 (3.5)	1 (0.4)*
Ketoacidosis[3]	Uncommon	2 (0.7)	2 (0.7)
**Psychiatric disorders**			
Insomnia	Common	22 (7.7)	
**Nervous system disorders**			
Headache	Very common	55 (19.4)	2 (0.7)*
Dysgeusia[4]	Very common	44 (15.5)	1 (0.4)*
**Eye disorders**			
Vision blurred	Common	15 (5.3)	1 (0.4)*
Dry eye	Common	10 (3.5)	
**Vascular disorders**			
Hypertension	Common	27 (9.5)	13 (4.6)
Lymphoedema	Common	16 (5.6)	
**Respiratory, thoracic and mediastinal disorders**			
Pneumonitis[5]	Common	5 (1.8)	1 (0.4)*
**Gastrointestinal disorders**			
Diarrhoea	Very common	169 (59.5)	20 (7.0)*
Nausea	Very common	133 (46.8)	8 (2.8)*
Stomatitis[6]	Very common	86 (30.3)	7 (2.5)*
Vomiting	Very common	81 (28.5)	2 (0.7)*
Abdominal pain	Very common	50 (17.6)	4 (1.4)*
Dyspepsia	Very common	33 (11.6)	
Toothache	Common	13 (4.6)	1 (0.4)*
Gingivitis	Common	11 (3.9)	1 (0.4)*
Gingival pain	Common	9 (3.2)	
Cheilitis	Common	8 (2.8)	
Pancreatitis	Uncommon	1 (0.4)	1 (0.4)
**Skin and subcutaneous tissue disorders**			
Rash[7]	Very common	147 (51.8)	55 (19.4)*
Alopecia	Very common	58 (20.4)	
Pruritus	Very common	53 (18.7)	2 (0.7)*
Dry skin[8]	Very common	53 (18.7)	1 (0.4)*
Erythema[9]	Common	18 (6.3)	2 (0.7)*
Dermatitis[10]	Common	10 (3.5)	2 (0.7)*
Palmar-plantar erythrodysaesthesia syndrome	Common	5 (1.8)	
Erythema multiforme	Common	3 (1.1)	2 (0.7)*
Stevens-Johnson syndrome	Uncommon	1 (0.4)	1 (0.4)*
Drug reaction with eosinophilia and systemic symptoms (DRESS)[#]	Not known	Not known	Not known
**Musculoskeletal and connective tissue disorders**			
Muscle spasms	Common	22 (7.7)	
Myalgia	Common	19 (6.7)	1 (0.4)*
Osteonecrosis of jaw	Common	16 (5.6)	5 (1.8)*
**Renal and urinary disorders**			
Acute kidney injury	Common	16 (5.6)	5 (1.8)

**General disorders and administration site conditions**			
Fatigue[11]	Very common	123 (43.3)	16 (5.6)*
Mucosal inflammation	Very common	56 (19.7)	6 (2.1)*
Oedema peripheral	Very common	47 (16.5)	
Pyrexia	Very common	45 (15.8)	2 (0.7)
Mucosal dryness[12]	Very common	36 (12.7)	1 (0.4)
Oedema[13]	Common	18 (6.3)	
**Investigations**			
Weight decreased	Very common	79 (27.8)	15 (5.3)*
Blood creatinine increased	Very common	192 (67.6)	8 (2.8)*
Gamma-glutamyltransferase increased	Very common	151 (53.2)	34 (12.0)
Alanine aminotransferase increased	Very common	125 (44.0)	12 (4.2)*
Lipase increased	Very common	121 (42.6)	20 (7.0)
Activated partial thromboplastin time (aPTT) prolonged	Very common	63 (22.2)	2 (0.7)
Albumin decreased	Very common	41 (14.4)	1 (0.4)
Glycosylated haemoglobin increased	Common	8 (2.8)	0

* No grade 4 ADRs were observed

# Adverse reactions reported during post-marketing experience. These are derived from spontaneousreports for which it is not always possible to reliably establish frequency or a causal relationship toexposure to the medicinal product.

[1] Urinary tract infection: also includes a single case of urosepsis
[2] Hypersensitivity: also includes allergic dermatitis
[3] Ketoacidosis: also includes diabetic ketoacidosis
[4] Dysgeusia: also includes ageusia, hypogeusia
[5] Pneumonitis: also includes interstitial lung disease
[6] Stomatitis: also includes aphthous ulcer and mouth ulceration
[7] Rash: also includes rash maculopapular, rash macular, rash generalised, rash papular, rash pruritic
[8] Dry skin: also includes skin fissures, xerosis, xeroderma
[9] Erythema: also includes erythema generalised
[10] Dermatitis: also includes dermatitis acneiform
[11] Fatigue: also includes asthenia
[12] Mucosal dryness: also includes dry mouth, vulvovaginal dryness
[13] Oedema: also includes face swelling, face oedema, eyelid oedema

22 TEPOTINIB

Tepotinib could be assigned to patients

- with no history of ILD or interstitial Pneumonitis including radiation pneumonitis that required steroid treatment
- with no prior treatment with EGFR and HER2 inhibitors (antibodies or small molecule inhibitors)

With the wording prior therapy with e.g. anti-EGFR Abs in CRC or anti-HER2 Abs based on HER2 extraction in BC, the treatment wit Tepotinib would be still possible.

Recommended Dosage

The recommended dosage of TEPOTINIB is 500 mg orally (i.e. 2 tablets of 250 mg dose strength) once daily with food until disease progression or unacceptable toxicity. "500 mg tepotinib" corresponds to 500 mg tepotinib HCl hydrate and is equivalent to 450 mg tepotinib (free base The active ingredient is tepotinib HCl hydrate and the active moiety is tepotinib (free base).

Instruct patients to take their dose of TEPOTINIB at approximately the same time every day and toswallow tablets whole. Do not chew, crush or split tablets.

Advise patients not to make up a missed dose within 8 hours of the next scheduled dose.

If vomiting occurs after taking a dose of TEPOTINIB, advise patients to take the next dose at thescheduled time.

Dose Modifications for Adverse Reactions

The recommended dose reduction of TEPOTINIB for the management of adverse reactions is 250 mg orally once daily. "250 mg tepotinib" corresponds to 250 mg tepotinib HCl hydrate and is equivalent to 225 mg tepotinib (free base). The active ingredient is tepotinib HCl hydrate and the active moiety is tepotinib (free base).

Permanently discontinue TEPOTINIB in patients who are unable to tolerate 250 mg orally once daily. The recommended dosage modifications of TEPOTINIB for adverse reactions are provided in Table 1.

Table 1: Recommended TEPOTINIB Dosage Modifications for Adverse Reactions

Adverse Reaction	Severity	Dose Modification
Interstitial Lung Disease (ILD) /Pneumonitis	Any grade	Withhold TEPOTINIB if ILD is suspected.<br>Permanently discontinue TEPOTINIB if ILD is confirmed.
**Adverse Reaction**	**Severity**	**Dose Modification**

Increased ALT and/or AST without increased total bilirubin	Grade 3	Withhold TEPOTINIB until recovery to baseline ALT/AST.<br>If recovered to baseline within 7 days, then resume TEPOTINIB at the same dose; otherwise resume TEPOTINIB at a reduced dose.
	Grade 4	Permanently discontinue TEPOTINIB.
Increased ALT and/or AST with increased total bilirubin in the absence of cholestasis or hemolysis	ALT and/or AST greater than 3 times ULN with total bilirubin greater than 2 times ULN	Permanently discontinue TEPOTINIB.
Increased total bilirubin without concurrent increased ALT and/or AST	Grade 3	Withhold TEPOTINIB until recovery to baseline bilirubin.<br>If recovered to baseline within 7 days, then resume TEPOTINIB at a reduced dose; otherwise permanently discontinue.
	Grade 4	Permanently discontinue TEPOTINIB.
Other adverse reactions	Grade 2	Maintain dose level. If intolerable, consider withholding TEPOTINIB until resolved, then resume TEPOTINIB at a reduced dose.
	Grade 3	Withhold TEPOTINIB until resolved, then resume TEPOTINIB at a reduced dose.
	Grade 4	Permanently discontinue TEPOTINIB.

Warnings and precautions

Interstitial Lung Disease (ILD)/Pneumonitis

ILD/pneumonitis, which can be fatal, occurred in patients treated with TEPOTINIB. ILD/pneumonitis occurred in 2.2% patients treated with TEPOTINIB, with one patient experiencing a Grade 3 or higher event; this event resulted in death. Four patients (0.9%) discontinued TEPOTINIB due to ILD/pneumonitis.

Monitor patients for new or worsening pulmonary symptoms indicative of ILD/pneumonitis (e.g., dyspnea, cough, fever). Immediately withhold TEPOTINIB in patients with suspected ILD/pneumonitisand permanently discontinue if no other potential causes of ILD/pneumonitis are identified.

Hepatotoxicity

Hepatotoxicity occurred in patients treated with TEPOTINIB. Increased alanine aminotransferase (ALT)/increased aspartate aminotransferase (AST) occurred in 13% of patients treated with TEPOTINIB. Grade 3 or 4 increased ALT/AST occurred in 4.2% of patients. A fatal adversereaction of hepatic failure occurred in one patient (0.2%). Three patients (0.7%) discontinued TEPOTINIB due to increased ALT/AST. The median time-to-onset of Grade 3 or higher increased ALT/AST was 30 days (range 1 to 178).

Monitor liver function tests (including ALT, AST, and total bilirubin) prior to the start of TEPOTINIB, every 2 weeks during the first 3 months of treatment, then once a month or as clinically indicated, with more frequent testing in patients who develop increased transaminases or bilirubin. Based on the severity of the adverse reaction, withhold, dose reduce, or permanently discontinue TEPOTINIB.

Embryo-Fetal Toxicity

Based on findings in animal studies and its mechanism of action TEPOTINIB can cause fetal harm whenadministered to a pregnant woman. Oral administration of tepotinib to pregnant rabbits during the period of organogenesis resulted in malformations (teratogenicity) and anomalies at exposures less than the human exposure based on area under the curve (AUC) at the 450 mg daily clinical dose. Advise pregnantwomen of the potential risk to a fetus. Advise females of reproductive potential or males with female partners of reproductive potential to use effective contraception during treatment with TEPOTINIB and for one week after the final dose.

ADVERSE REACTIONS

The following adverse reactions are described in greater detail elsewhere in the labeling:

- Interstitial Lung Disease/Pneumonitis
- Hepatotoxicity

Clinical Trials Experience

Because clinical trials are conducted under widely varying conditions, adverse reaction rates observed inthe clinical trials of a drug cannot be directly compared to rates in the clinical trials of another drug and may not reflect the rates observed in practice.

The pooled safety population described in the WARNINGS AND PRECAUTIONS reflect exposure to TEPOTINIB in 448 patients with solid tumors enrolled in five open-label, single-arm studies receiving TEPOTINIB as single agent at a dose of 450 mg once daily. This included 255 patients with NSCLC positive for *MET*ex14 skipping alterations, who received TEPOTINIB in VISION. Among 448 patients who received TEPOTINIB, 32% were exposed for 6 months or longer, and 12% were exposed for greaterthan one year.

The data described below reflect exposure to TEPOTINIB 450 mg once daily in 255 patients with metastatic non-small cell lung cancer (NSCLC) with *MET*ex14 skipping alterations in VISION.

Serious adverse reactions occurred in 45% of patients who received TEPOTINIB. Serious adverse reactions in > 2% of patients included pleural effusion (7%), pneumonia (5%), edema (3.9%), dyspnea (3.9%), general health deterioration (3.5%), pulmonary embolism (2%), and musculoskeletal pain (2%).Fatal adverse reactions occurred in one patient (0.4%) due to pneumonitis, one patient (0.4%) due to hepatic failure, and one patient (0.4%) due to dyspnea from fluid overload.

Permanent discontinuation due to an adverse reaction occurred in 20% of patients who received TEPOTINIB. The most frequent adverse reactions (> 1%) leading to permanent discontinuations ofTEPOTINIB were edema (5%), pleural effusion (2%), dyspnea (1.6%), general health deterioration(1.6%), and pneumonitis (1.2%).

Dosage interruptions due to an adverse reaction occurred in 44% of patients who received TEPOTINIB.Adverse reactions which required dosage interruption in > 2% of patients who received TEPOTINIB included edema (23%), increased blood creatinine (6%), pleural effusion (4.3%), increased ALT (3.1%),
and pneumonia (2.4%).

Dose reductions due to an adverse reaction occurred in 30% of patients who received TEPOTINIB.Adverse reactions which required dose reductions in > 2% of patients who received TEPOTINIB included edema (19%), pleural effusion (2.7%), and increased blood creatinine (2.7%).

The most common adverse reactions (≥ 20%) in patients who received TEPOTINIB were edema, fatigue,nausea, diarrhea, musculoskeletal pain, and dyspnea. The most common Grade 3 to 4 laboratory abnormalities (≥ 2%) were decreased lymphocytes, decreased albumin, decreased sodium, increased gamma-glutamyltransferase, increased amylase, increased ALT, increased AST, and decreased hemoglobin.

Table 2 summarizes the adverse reactions in VISION.

Table 2: Adverse Reactions in ≥ 10% of Patients with NSCLC with *MET*ex14 SkippingAlterations Who Received TEPOTINIB in VISION

Adverse Reactions	TEPOTINIB (N = 255)	
	All Grades (%)	Grades 3 to 4 (%)
**General disorders and administration-site conditions**		
Edema [a]	70	9
Fatigue [b]	27	1.6
**Adverse Reactions**	**TEPOTINIB (N = 255)**	
	**All Grades (%)**	**Grades 3 to 4 (%)**
**Gastrointestinal disorders**		
Nausea	27	0.8
Diarrhea	26	0.4
Abdominal Pain [c]	16	0.8
Constipation	16	0
Vomiting [d]	13	1.2
**Musculoskeletal and Connective Tissue Disorders**		
Musculoskeletal Pain [e]	24	2.4
**Respiratory, thoracic, and mediastinal disorders**		
Dyspnea [f]	20	2
Cough [g]	15	0.4
Pleural effusion	13	5

**Metabolism and nutrition disorders**		
Decreased appetite	16	1.2
**Infections and Infestations**		
Pneumonia [h]	11	3.9

[a] Edema includes eye edema, face edema, generalized edema, localized edema, edema, genital edema, peripheral edema,peripheral swelling, periorbital edema, and scrotal edema.
[b] Fatigue includes asthenia and fatigue.
[c] Abdominal Pain includes abdominal discomfort, abdominal pain, abdominal pain lower, abdominal pain upper,gastrointestinal pain, and hepatic pain.
[d] Vomiting includes retching and vomiting.
[e] Musculoskeletal Pain includes arthralgia, arthritis, back pain, bone pain, musculoskeletal chest pain, musculoskeletal pain,myalgia, non-cardiac chest pain, pain in extremity, and spinal pain.
[f] Dyspnea includes dyspnea, dyspnea at rest, and dyspnea exertional.
[g] Cough includes cough, and productive cough.
[h] Pneumonia includes pneumonia, pneumonia aspiration, and pneumonia bacterial.

Clinically relevant adverse reactions in < 10% of patients who received TEPOTINIB includedILD/pneumonitis, rash, fever, dizziness, pruritus, and headache.

Table 3 summarizes the laboratory abnormalities observed in VISION.

Table 3: Select Laboratory Abnormalities (≥ 20%) That Worsened from Baseline in PatientsWho Received TEPOTINIB in VISION

**Laboratory Abnormalities**	**TEPOTINIB[1]**	
	**Grades 1 to 4 (%)**	**Grades 3 to 4 (%)**
**Chemistry**		
Decreased albumin	76	9
Increased creatinine	55	0.4
Increased alkaline phosphatase	50	1.6
Increased alanine aminotransferase	44	4.1
Increased aspartate aminotransferase	35	2.5
Decreased sodium	31	8
Increased potassium	25	1.6
Increased gamma-glutamyltransferase	24	5
Increased amylase	23	4.6
**Hematology**		
Decreased lymphocytes	48	11
Decreased hemoglobin	27	2
Decreased leukocytes	23	0.8

[1] The denominator used to calculate the rate varied from 207 to 246 based on the number of patients with a baseline value andat least one post-treatment value.

A clinically relevant laboratory abnormality in < 20% of patients who received TEPOTINIB wasincreased lipase in 18% of patients, including 3.7% Grades 3 to 4.

<u>Increased Creatinine</u>

A median increase in serum creatinine of 31% was observed 21 days after initiation of treatment with TEPOTINIB. The serum creatinine increases persisted throughout treatment and were reversible upontreatment completion.

DRUG INTERACTIONS

Effects of Other Drugs on TEPOTINIB

Dual Strong CYP3A Inhibitors and P-gp Inhibitors

The effect of strong CYP3A inhibitors or P-gp inhibitors on TEPOTINIB has not been studied clinically. However, metabolism and in vitro data suggest concomitant use of drugs that are strong CYP3A inhibitors and P-gp inhibitors may increase tepotinib exposure, whichmay increase the incidence and severity of adverse reactions of TEPOTINIB. Avoid concomitant use of TEPOTINIB with dual strong CYP3A inhibitors and P-gp inhibitors.

Strong CYP3A Inducers

The effect of strong CYP3A inducers on TEPOTINIB has not been studied clinically. However, metabolism and in vitro data suggest concomitant use may decrease tepotinib exposure which may reduce TEPOTINIB efficacy. Avoid concomitant use of TEPOTINIBwith strong CYP3A inducers.

Effects of TEPOTINIB on Other Drugs

Certain P-gp Substrates

Tepotinib is a P-gp inhibitor. Concomitant use of TEPOTINIB increases the concentration of P-gp substrates, which may increase the incidence and severity of adversereactions of these substrates. Avoid concomitant use of TEPOTINIB with certain P-gp substrates where minimal concentration changes may lead to serious or life-threatening toxicities. If concomitant use is unavoidable, reduce the P-gp substrate dosage if recommended in its approved product labeling

Geriatric Use

Of 255 patients with *MET*ex14 skipping alterations in VISION who received 450 mg TEPOTINIB once daily, 79% were 65 years or older, and 43% were 75 years or older. No clinically important differences insafety or efficacy were observed between patients aged 65 years or older and younger patients.

Renal Impairment

No dosage modification is recommended in patients with mild or moderate renal impairment (creatinineclearance [CLcr] 30 to 89 mL/min, estimated by Cockcroft-Gault). The recommended dosage has not been established for patients with severe renal impairment (CLcr < 30 mL/min).

Hepatic Impairment

No dosage modification is recommended in patients with mild (Child Pugh Class A) or moderate (ChildPugh Class B) hepatic impairment. The pharmacokinetics and safety of tepotinib in patients with severehepatic impairment (Child Pugh Class C) have not been studied.

23 GAVRETO (PRALSETINIB)

DOSAGE AND ADMINISTRATION

Recommended Dosage

The recommended dosage of pralsetinib is 400 mg orally once daily on an empty stomach (no food intake for at least 2 hours before and at least 1 hour after taking pralsetinib. Continue treatment until disease progression or until unacceptable toxicity.

If a dose of pralsetinib is missed, it can be taken as soon as possible on the same day. Resume the regular daily dose schedule for pralsetinib the next day.

Do not take an additional dose if vomiting occurs after pralsetinib but continue with the nextdose as scheduled.

Dosage Modifications for Adverse Reactions

The recommended dose reductions and dosage modifications for adverse reactions are provided in Table 1 and Table 2.

Table 1: Recommended Dose Reductions for pralsetinib for Adverse Reactions

Dose Reduction	Recommended Dosage
**First**	300 mg once daily
**Second**	200 mg once daily
**Third**	100 mg once daily

Permanently discontinue pralsetinib in patients who are unable to tolerate 100 mg taken orallyonce daily.

The recommended dosage modifications for adverse reactions are provided in Table 2

Table 2: Recommended Dosage Modifications for pralsetinib for Adverse Reactions

Adverse Reaction	Severity*	Dosage Modification
**ILD/Pneumonitis**	Grade 1 or 2	Withhold pralsetinib until resolution. Resume by reducingthe dose as shown in Table 1.<br>Permanently discontinue pralsetinib for recurrentILD/pneumonitis.
	Grade 3 or 4	Permanently discontinue forconfirmed ILD/pneumonitis.

**Hypertension**	Grade 3	Withhold pralsetinib for Grade3 hypertension that persists despite optimal antihypertensivetherapy. Resume at a reduced dose when hypertension is controlled.
	Grade 4	Discontinue pralsetinib.
**Hepatotoxicity**	Grade 3 or Grade 4	Withhold pralsetinib and monitor AST/ALT once weeklyuntil resolution to Grade 1 or baseline.<br><br>Resume at reduced dose (Table1).<br><br>If hepatotoxicity recurs at Grade3 or higher, discontinue pralsetinib.
**Hemorrhagic Events**	Grade 3 or Grade 4	Withhold pralsetinib until recovery to baseline or Grade 0 or 1.<br>Discontinue pralsetinib forsevere or life-threatening hemorrhagic events.
**QT prolongation**	Grade 3	Interrupt treatment with pralsetinib for QTc intervals >500 ms until QTc interval returns to <470 ms.<br>Resume at the same dose if risk factors that cause QT prolongation are identified and corrected.<br>Resume treatment at a reduced dose if other risk factors that cause QT prolongation are not identified.
	Grade 4	Permanently discontinue pralsetinib if the patient has life-threatening arrhythmia.
**Other Adverse Reactions**	Grade 3 or 4	Withhold pralsetinib until improvement to ≤ Grade 2. Resume at reduced dose (Table 1).<br><br>Permanently discontinue forrecurrent Grade 4 adverse reactions.

* Adverse reactions graded by the National Cancer Institute Common Terminology Criteria for Adverse Events(NCI-CTCAE) version 4.03

Dose Modification for Use with Combined P-glycoprotein (P-gp) andStrong CYP3A Inhibitors

Avoid coadministration of pralsetinib with known combined P-gp and strong CYP3A inhibitors. If coadministration with a combined P-gp and strong CYP3A inhibitor cannot be avoided, reduce the current dose of pralsetinib as recommended in Table 3. After the inhibitorhas been discontinued for 3 to 5 elimination half-lives, resume pralsetinib at the dose taken prior to initiating the combined P-gp and strong CYP3A inhibitor.

Table 3: Recommended Dosage Modifications for pralsetinib for Coadministrationwith Combined P-gp and Strong CYP3A Inhibitors

Current pralsetinib Dosage	Recommended pralsetinib Dosage
400 mg orally once daily	200 mg orally once daily
300 mg orally once daily	200 mg orally once daily
200 mg orally once daily	100 mg orally once daily

Dose Modification for Use with Strong CYP3A Inducers

Avoid coadministration of pralsetinib with strong CYP3A inducers. If coadministration with a strong CYP3A inducer cannot be avoided, increase the starting dose of pralsetinib to double the current pralsetinib dosage starting on Day 7 of coadministration of pralsetinib with the strong CYP3A inducer. After the inducer has been discontinued for at least 14 days, resume pralsetinib at the dose taken prior to initiating the strong CYP3A inducer.

WARNINGS AND PRECAUTIONS

Interstitial Lung Disease/Pneumonitis

Severe, life-threatening, and fatal interstitial lung disease (ILD)/pneumonitis can occur inpatients treated with pralsetinib. Pneumonitis occurred in 10% of patients who receivedpralsetinib, including 2.7% with Grade 3-4, and 0.5% with fatal reactions. Monitor for pulmonary symptoms indicative of ILD/pneumonitis. Withhold pralsetinib and promptly investigate for ILD in any patient who presents with acute or worsening of respiratorysymptoms which may be indicative of ILD (e.g., dyspnea, cough, and fever). Withhold, reduce dose or permanently discontinue pralsetinib based on severity of confirmed ILD.

Hypertension

Hypertension occurred in 29% of patients, including Grade 3 hypertension in 14% of patients. Overall, 7% had their dose interrupted and 3.2% had their dose reduced for hypertension. Treatment-emergent hypertension was most commonly managed withanti-hypertension medications.

Do not initiate pralsetinib in patients with uncontrolled hypertension. Optimize blood pressure prior to initiating pralsetinib. Monitor blood pressure after 1 week, at least monthly thereafter and as clinically indicated. Initiate or adjust anti-hypertensive therapy as appropriate. Withhold, reduce dose, or permanently discontinue pralsetinib based on the severity.

Hepatotoxicity

Serious hepatic adverse reactions occurred in 2.1% of patients treated for pralsetinib. Increased AST occurred in 69% of patients, including Grade 3 or 4 in 5.4% and increased ALT occurred in 46% of patients, including Grade 3 or 4 in 6%. The median time tofirst onset for increased AST was 15 days (range: 5 days to 1.5 years) and increased ALT was 22 days (range: 7 days to 1.7 years).
Monitor AST and ALT prior to initiating pralsetinib, every 2 weeks during the first 3 months,then monthly thereafter and as clinically indicated. Withhold, reduce dose or permanently discontinue pralsetinib based on severity

Hemorrhagic Events

Serious, including fatal, hemorrhagic events can occur with pralsetinib. Grade ≥ 3 hemorrhagic events occurred in 2.5% of patients treated with pralsetinib including one patient with a fatal hemorrhagic event. Permanently discontinue pralsetinib in patients with severe or life-threatening hemorrhage.

Tumor Lysis Syndrome

Tumor lysis syndrome (TLS) has been observed in < 1% of patients following pralsetinib treatment; all events included confounding factors. Investigators should monitor electrolyte status and renal function via laboratory testing. Patients with TLS may require supportive care with IV fluids and correction of electrolyte imbalances.

Patients that are at risk for TLS at study entry, such as those with high tumor burden, should be well hydrated before initiation of pralsetinib and avoid dehydration during the first cycle. If TLS is suspected, Investigators should manage the AE(s) according to standard institutional practices or accepted oncology management guidelines (Coiffier et al, 2008; Klastersky et al, 2016).

Decrease in Blood Counts

Decrease in blood counts (white blood cells, red blood cells, and platelets) have been observed following pralsetinib treatment. Preliminary analysis indicates that pralsetinib appears to cause a decrease in blood counts particularly within the first 3 weeks of treatment. However, blood counts tend to stabilize over time and rarely become worse than Grade 2. Investigators should monitor blood counts via routine laboratory testing. If the causality of decreased blood counts is at least possibly related to pralsetinib, management with supportive medications, dose interruption, and potential dose reduction and/or discontinuation should follow clinical standards of care and/or the guidelines in the specific protocol being used.

Physeal Dysplasia

Effects on bone including physeal dysplasia in both rodents and non-human primates and incisor tooth degeneration in rats were observed at exposures (AUC0-24) similar to clinical exposures at the 400 mg QD dose. These findings are attributed to off-target VEGFR inhibition which may impact actively growing bones and teeth.

Monitor growth plates in adolescent patients with open growth plates by performing serial X-rays at baseline, at 6 weeks, at Cycle 6, and every 6 months until the EOT. If an effect on growth plates is observed and causality is at least possibly related to pralsetinib, consider interrupting, dose reduction, and/or discontinuation of therapy based on the severity of any growth plate abnormalities.

Risk of Impaired Wound Healing

Impaired wound healing can occur in patients who receive drugs that inhibit the vascular endothelial growth factor (VEGF) signaling pathway. Therefore, pralsetinib has the potential to adversely affect wound healing.

Withhold pralsetinib for at least 5 days prior to elective surgery. Do not administer for at least 2 weeks following major surgery and until adequate wound healing. The safety of resumption of pralsetinib after resolution of wound healing complications has not been established.

Pregnancy

A reproductive toxicology study with pralsetinib in humans has not been performed. However, based on the preclinical embryofetal development study in rats that demonstrated severe embryofetal toxicity at all dosage levels, patients should be advised that pralsetinib may be toxic to a developing fetus. No information is available on the safety or efficacy of pralsetinib in pregnant females. Pralsetinib should not be administered to pregnant women.

Females of childbearing potential must either abstain from heterosexual intercourse or use at least one highly effective method of non-hormonal contraception (CTFG, 2014) prior to initiating treatment with pralsetinib. Males with female partners of childbearing potential must either abstain from sexual intercourse or they and their partners must use at least one highly effective method of contraception after initiating treatment with pralsetinib. Additional guidance is provided in the clinical study protocols.

Clinical Trials Experience

As of the cutoff date of 18 December 2020, 7 Blueprint-sponsored clinical studies have been initiated with pralsetinib (Table 13). Five Phase 1 studies in healthy volunteers have been completed, while 2 studies in patients with RET-altered solid tumors are ongoing: BLU-667-1101 (“ARROW”), a Phase 1/2 study of pralsetinib in patients with RET-altered cancers, and BLU-667-2303 (“AcceleRET-Lung”), a Phase 3 study of pralsetinib in comparison to Investigator’s choice platinum-containing chemotherapy regimen with or without pembrolizumab (i.e., standard of care) in patients with RET fusion-positive NSCLC. The current clinical experience with pralsetinib has demonstrated the following:
• In the dose-escalation portion of Study BLU-667-1101, pralsetinib doses ranging from 30 to 600 mg QD, 200/100 mg BID, and 100/100 mg BID were evaluated, and 400 mg QD was determined as the pralsetinib MTD. With consideration of available pharmacokinetic, pharmacodynamic, efficacy, and safety data, 400 mg QD was also selected as the recommended starting dose for the Phase 2 portion of Study BLU-667-1101.
Across the entire clinical experience, a total of 199 healthy volunteers and 717 oncology patients have received pralsetinib, including 578 patients in the Study BLU-667-1101, 4 patients in Study BLU-667-2303, and 135 patients who received pralsetinib as part of a pre-approval access program (PAAP). Among the 578 patients who received pralsetinib in Study BLU-667-1101, the most common treatment-related adverse events (AEs) (≥ 15%) have included aspartate aminotransferase increased, anaemia, alanine aminotransferase increased, white blood cell count decreased, constipation, hypertension, neutrophil count decreased, neutropenia, and hyperphosphataemia. Overall, 117 patients (20.2%) experienced serious adverse events (SAEs) considered to be related to study drug. Treatment-related SAEs occurring in ≥1% of patients included pneumonitis, pneumonia, anaemia, and neutropenia. A low rate of treatment discontinuation due to treatment-related AEs has been observed (5.9%).
• Pralsetinib administered at a dose of 400 mg QD had no clinically relevant or statistically significant effects on the ECG findings in a subset of patient (N = 34), and specifically was not associated with evidence of QTc prolongation.
• Pralsetinib administered at 400 mg QD has demonstrated broad and durable antitumor activity in patients with RET fusion-positive NSCLC, RET-mutant MTC, and RET fusion-positive thyroid cancer.
o As presented at the American Society of Clinical Oncology (ASCO) with a data cutoff date of 18 November 2019, in patients with RET fusion-positive NSCLC previously treated with platinum-containing chemotherapy, the overall response rate (ORR) was 61% (95% CI: 50-72%; 2 responses pending confirmation). In treatment-naïve patients, the ORR was 73% (95% CI: 52-88%; all confirmed). Among all patients with RET fusion-positive NSCLC regardless of

prior treatment, median DOR was not reached (95% CI: 11.3 months - not estimable), 96% of patients had measurable tumor shrinkage (including 100% of treatment-naïve patients), and the complete response rate was 6% (12% in treatment-naïve patients).

o As presented at the European Society for Medical Oncology (ESMO) conference with a data cutoff date of 13 February 2020, among patients with RET-mutant MTC who previously received cabozantinib and/or vandetanib, the ORR was 60% (95% CI: 46-74%; 1 response pending confirmation as of the data cutoff date). Disease control rate (DCR) was 96% (95% CI: 87-100%). Median DOR was not reached, with 94% of responding patients remaining on treatment. In the treatment-naïve patients, the ORR was 74% (95% CI: 49-91%; all responses confirmed). DCR was 100% (95% CI: 82-100%). Median DOR was not reached, with 93% of responding patients remaining on treatment.

As presented at the ASCO conference with a data cutoff of 13 February 2020, among patients with RET fusion-positive thyroid cancer, the ORR was 91% (95% CI: 59-100%) and all patients had measurable tumor shrinkage.

o As presented at the ASCO conference, antitumor activity has been demonstrated in patients with various other RET fusion-positive solid tumor types. As of a data cutoff of 13 February 2020, ORR among 12 patients with other RET fusion-positive solid tumor types was 50% (95% CI: 21-79%), with partial response observed in all patients with RET fusion-positive pancreatic adenocarcinoma (3/3), cholangiocarcinoma (2/2), and neuroendocrine tumor (1/1). DCR was 92%, and tumor shrinkage was observed in 92% of patients.

• The oral bioavailability of pralsetinib administered at a dose of 200 mg was increased when given under fed (standardized high-fat meal) conditions. In addition, food significantly delayed the absorption of pralsetinib compared to fasted conditions. Considering these results, pralsetinib will be administered with a glass of water (at least 8 ounces or 250 mL) in a fasted state, with no food intake from 2 hours before until 1 hour after study drug administration.

• Pralsetinib underwent limited metabolism following single oral dose of ~ 310 mg [14C]pralsetinib; excretion in feces (72%) was the predominant route of elimination and urine (6%) was a minor route of elimination. Unchanged pralsetinib was the predominant radioactive component in plasma, urine and feces, while its metabolites from oxidation and glucuronidation were detected in trace amounts (~5%). Overall, the metabolism of pralsetinib in humans was qualitatively similar to nonclinical toxicology species (rat and monkey), with no unique human metabolites observed.

• Coadministration of itraconazole, a strong CYP3A and P-gp inhibitor, increased pralsetinib Cmax by 84% and AUCInf by 251%, relative to a 200 mg dose of pralsetinib administered alone. Coadministration of rifampin, a strong CYP3A and P-gp inducer, decreased pralsetinib Cmax by 30% and AUC0-∞ by 68%, relative to a 400 mg dose of pralsetinib administered alone. The concomitant use of strong CYP3A inhibitors and inducers or combined P-gp and strong CYP3A inhibitors with pralsetinib should be avoided.

• Coadministration of esomeprazole, a proton-pump inhibitor, decreased pralsetinib Cmax by 25% and AUC0–∞ by 15%, relative to a 400 mg dose of pralsetinib administered alone. These changes are modest and unlikely to be clinically relevant.

DRUG INTERACTION

Effect of Other Drugs on Pralsetinib

<u>Strong CYP3A Inhibitors</u>

Avoid coadministration with strong CYP3A inhibitors. Coadministration of pralsetinib with a strong CYP3A inhibitor increases pralsetinib exposure, which may increase the incidence and severity of adverse reactions of pralsetinib.

Avoid coadministration of pralsetinib with combined P-gp and strong CYP3A inhibitors. If coadministration with a combined P-gp and strong CYP3A inhibitor cannot be avoided, reducethe pralsetinib dose.

Strong CYP3A Inducers

Coadministration of pralsetinib with a strong CYP3A inducer decreases pralsetinib exposure, which may decrease efficacy of pralsetinib. Avoid coadministration of pralsetinib with strong CYP3A inducers. If coadministration cannot be avoided, increase the pralsetinib dose.

Management of Potential Toxicity and Overdose

There have been no instances of pralsetinib overdose in humans, and the effects of a pralsetinib overdose are not known. General supportive measures should be undertaken in the event of an overdose.

24 TALZENNA (TALAZOPARIB)

<u>Posology</u>

The recommended dose is 1 mg talazoparib once daily. Patients should be treated until diseaseprogression or unacceptable toxicity occurs.

Missing dose
If the patient vomits or misses a dose, an additional dose should not be taken. The next prescribed dose should be taken at the usual time.

Dose adjustments
To manage adverse drug reactions, interruption of treatment or dose reduction based on severity andclinical presentation should be considered (Table 2). Recommended dose reductions are indicated inTable 1.

Table 1. Dose adjustments for toxicities

	**Dose level**
Recommended starting dose	1 mg (one 1 mg capsule) once daily
First dose reduction	0.75 mg (three 0.25 mg capsules) once daily
Second dose reduction	0.5 mg (two 0.25 mg capsules) once daily
Third dose reduction	0.25 mg (one 0.25 mg capsule) once daily

Complete blood count should be obtained prior to starting talazoparib therapy and monitored monthlyand as clinically indicated

Table 2. Dose modification and management

	**Withhold talazoparib untillevels resolve to**	**Resume talazoparib**
Haemoglobin < 8 g/dL	≥ 9 g/dL	Resume talazoparib at nextlower dose
Platelet count < 50,000/μL	≥ 75,000/μL	
Neutrophil count < 1,000/μL	≥ 1,500/μL	
Non-haematologic adverse reaction Grade 3 or Grade 4	[illegible] Grade 1	Consider resuming talazoparibat next lower dose or discontinue

Concomitant treatment with inhibitors of P-glycoprotein (P-gp)
Strong inhibitors of P-gp may lead to increased talazoparib exposure. Concomitant use of strong P-gpinhibitors during treatment with talazoparib should be avoided. Co-administration should only be considered after careful evaluation of the potential benefits and risks. If co-administration with a strong P-gp inhibitor is unavoidable, the talazoparib dose should be reduced to the next lower dose.
When the strong P-gp inhibitor is discontinued, the talazoparib dose should be increased (after
3-5 half-lives of the P-gp inhibitor) to the dose used prior to the initiation of the strong P-gp inhibitor.

Method of administration

talazoparib is for oral use. To avoid contact with the capsule content, the capsules should be swallowed whole, and must not be opened or dissolved. They can be taken with or without food

Special populations

Hepatic impairment
No dose adjustment is required for patients with mild hepatic impairment (total bilirubin ≤ 1 × upperlimit of normal [ULN] and aspartate aminotransferase (AST) > ULN, or total bilirubin > 1.0 to

1.5 × ULN and any AST). talazoparib has not been studied in patients with moderate (total bilirubin > 1.5 to 3.0 × ULN and any AST) or severe hepatic impairment (total bilirubin > 3.0 × ULN and any AST). talazoparib may only be used in patients with moderate or severe hepatic impairment if the benefit outweighs the potential risk, and the patient should be carefully monitored for hepatic function and adverse events.

Renal impairment
No dose adjustment is required for patients with mild renal impairment (60 mL/min ≤ creatinineclearance [CrCL] < 90 mL/min). For patients with moderate renal impairment (30 mL/min ≤ CrCL < 60 mL/min), the recommended starting dose of talazoparib is 0.75 mg once daily.For patients with severe renal impairment (15 mL/min ≤ CrCL < 30 mL/min), the recommended starting dose of talazoparib is 0.5 mg once daily. talazoparib has not been studied in patients with CrCL < 15 mL/min or patients requiring haemodialysis.

Elderly
No dose adjustment is necessary in elderly (≥ 65 years of age) patients.

Special warnings and precautions for use

Myelosuppression

Myelosuppression consisting of anaemia, leucopenia/neutropenia, and/or thrombocytopenia, have been reported in patients treated with talazoparib (see section 4.8). Talazoparib should not be starteduntil patients have recovered from haematological toxicity caused by previous therapy (≤ Grade 1). Precautions should be taken to routinely monitor haematology parameters and signs and symptoms associated with anaemia, leucopenia/neutropenia, and/or thrombocytopenia in patients receiving talazoparib. If such events occur, dose modifications (reduction or interruption) are recommended. Supportive care with or without blood and/or platelet transfusions and/or administrationof colony stimulating factors may be used as appropriate.

Myelodysplastic syndrome/Acute myeloid leukaemia

Myelodysplastic syndrome/Acute Myeloid Leukaemia (MDS/AML) have been reported in patients who received poly (adenosine diphosphate-ribose) polymerase (PARP) inhibitors, including talazoparib. Overall, MDS/AML has been reported in 2 out of 584 (0.3%) solid tumour patients treated with talazoparib in clinical studies. Potential contributing factors for the development of MDS/AML include previous platinum-containing chemotherapy, other DNA damaging agents or radiotherapy.

Complete blood counts should be obtained at baseline and monitored monthly for signs of haematologic toxicity during treatment. If MDS/AML is confirmed, talazoparib should be discontinued.

Interaction with other medicinal products and other forms of interaction

Talazoparib is a substrate for drug transporters P-gp and Breast Cancer Resistance Protein (BCRP) and it is mainly eliminated by renal clearance as unchanged compound.

<u>Agents that may affect talazoparib plasma concentrations</u>

P-gp inhibitors
Data from a drug-drug interaction study in patients with advanced solid tumours indicated that
co-administration of multiple daily doses of a P-gp inhibitor, itraconazole 100 mg twice daily with a single 0.5 mg talazoparib dose increased talazoparib total exposure (AUC_{inf}) and peak concentration (C_{max}) by approximately 56% and 40%, respectively, relative to a single 0.5 mg talazoparib dose administered alone. Population pharmacokinetic (PK) analysis has also shown that concomitant use of strong P-gp inhibitors increased talazoparib exposure by 45%, relative to talazoparib given alone.

Concomitant use of strong P-gp inhibitors (including but not limited to amiodarone, carvedilol, clarithromycin, cobicistat, darunavir, dronedarone, erythromycin, indinavir, itraconazole, ketoconazole, lapatinib, lopinavir, propafenone, quinidine, ranolazine, ritonavir, saquinavir, telaprevir, tipranavir, and verapamil) should be avoided. If co-administration with a strong P-gp inhibitor is unavoidable, the talazoparib dose should be reduced.

P-gp inducers
Data from a drug-drug interaction study in patients with advanced solid tumours indicated that
co-administration of single 1 mg talazoparib dose with multiple daily doses of a P-gp inducer, rifampin 600 mg, with rifampin co-administered 30 minutes before talazoparib on the day of talazoparib dosing, increased talazoparib C_{max} by approximately 37% whereas AUC_{inf} was not affected relative to a single 1 mg talazoparib dose administered alone. This is probably the net effect of both P-gp induction and inhibition by rifampin under the tested conditions in the drug-drug interaction study. No talazoparib dose adjustments are required when co-administered with rifampin. However, the effect of other P-gp

inducers on talazoparib exposure has not been studied. Other P-gp inducers (including but not limited tocarbamazepine, phenytoin, and St. John's wort) may decrease talazoparib exposure.

BCRP inhibitors
The effect of BCRP inhibitors on PK of talazoparib has not been studied *in vivo*. Co-administration oftalazoparib with BCRP inhibitors may increase talazoparib exposure. Concomitant use of strong BCRP inhibitors (including but not limited to curcumin and cyclosporine) should be avoided. If

co-administration of strong BCRP inhibitors cannot be avoided, patient should be monitored forpotential increased adverse reactions.

Effect of acid-reducing agents
Population PK analysis indicates that co-administration of acid-reducing agents including proton pump inhibitors and histamine receptor 2 antagonists (H2RA), or other acid-reducing agents had nosignificant impact on the absorption of talazoparib.

Systemic hormonal contraception
Drug-drug interaction studies between talazoparib and oral contraceptives have not been conducted.

talazoparib may have a minor influence on the ability to drive and use machines. Fatigue/asthenia ordizziness may occur following administration of talazoparib.

Undesirable effects
<u>Summary of the safety profile</u>

The overall safety profile of talazoparib is based on pooled data from 494 patients who receivedtalazoparib at 1 mg daily in clinical studies for solid tumours, including 286 patients from a randomised Phase 3 study with germline BRCA-mutated (gBRCAm), HER2-negative locally

advanced or metastatic breast cancer and 83 patients from a nonrandomised Phase 2 study in patientswith germline BRCA-mutated locally advanced or metastatic breast cancer.

The most common (≥ 25%) adverse reactions in patients receiving talazoparib in these clinical studieswere fatigue (57.1%), anaemia (49.6%), nausea (44.3%), neutropenia (30.2%), thrombocytopenia (29.6%), and headache (26.5%). The most common (≥ 10%) Grade ≥ 3 adverse reactions of talazoparib were anaemia (35.2%), neutropenia (17.4%), and thrombocytopenia (16.8%).

Dose modifications (dose reductions or dose interruptions) due to any adverse reaction occurred in62.3% of patients receiving talazoparib. The most common adverse reactions leading to dose modifications were anaemia (33.0%), neutropenia (15.8%), and thrombocytopenia (13.4%).

Permanent discontinuation due to an adverse reaction occurred in 3.6% of patients receiving talazoparib.The median duration of exposure was 5.4 months (range 0.03-61.1).

<u>Tabulated list of adverse reactions</u>

Table 3 summarises adverse reactions based on pooled dataset listed by system organ class, and frequency category. Frequency categories are defined as: very common (≥ 1/10) and common (≥ 1/100to < 1/10). Within each frequency grouping, adverse reactions are presented in order of decreasing seriousness.

Table 3. Adverse reactions based on pooled dataset from 5 studies (N=494)

System organ class<br>Frequency<br>Preferred term	All grades* n (%)	Grade 3n (%)	Grade 4n (%)
**Blood and lymphatic system disorders**			
Very common			
Thrombocytopenia[a]	146 (29.6)	63 (12.8)	20 (4.0)
Anaemia[b]	245 (49.6)	172 (34.8)	2 (0.4)
Neutropenia[c]	149 (30.2)	77 (15.6)	9 (1.8)
Leucopenia[d]	77 (15.6)	24 (4.9)	1 (0.2)
Common			
Lymphopenia[e]	30 (6.1)	13 (2.6)	0 (0.0)
**Metabolism and nutrition disorders**			
Very common			
Decreased appetite	100 (20.2)	2 (0.4)	0 (0.0)
**Nervous system disorders**			
Very common			
Dizziness	69 (14.0)	1 (0.2)	N/A
Headache	131 (26.5)	5 (1.0)	N/A
Common			
Dysgeusia	42 (8.5)	0 (0.0)	0 (0.0)
**Gastrointestinal disorders**			
Very common			
Vomiting	110 (22.3)	7 (1.4)	0 (0.0)
Diarrhoea	112 (22.7)	3 (0.6)	0 (0.0)
Nausea	219 (44.3)	4 (0.8)	N/A
Abdominal pain[f]	105 (21.3)	8 (1.6)	N/A
Common			
Stomatitis	32 (6.5)	0 (0.0)	0 (0.0)
Dyspepsia	41 (8.3)	0 (0.0)	N/A

**Skin and subcutaneous tissue disorders**<br>*Very common*<br>Alopecia[g]	110 (22.3)	N/A	N/A
**System organ class Frequency Preferred term**	**All grades[*] n (%)**	**Grade 3n (%)**	**Grade 4n (%)**
**General disorders and administration siteconditions**<br>*Very common*<br>Fatigue[h]	282 (57.1)	17 (3.4)	1 (0.2)

Abbreviations: n=number of patients; N/A=not applicable.
[*] There were no Grade 5 adverse drug reactions.
[a.] Includes preferred terms of thrombocytopenia and platelet count decreased.
[b.] Includes preferred terms of anaemia, haematocrit decreased and haemoglobin decreased.
[c.] Includes preferred terms of neutropenia and neutrophil count decreased.
[d.] Includes preferred terms of leucopenia and white blood cell count decreased.
[e.] Includes preferred terms of lymphocyte count decreased and lymphopenia.
[f.] Includes preferred terms of abdominal pain, abdominal pain upper, abdominal discomfort and abdominal painlower.
[g.] For talazoparib Grade 1 is 21% and Grade 2 is 2%.
[h.] Includes preferred terms of fatigue and asthenia.

Description of selected adverse reactions

Myelosuppression

Myelosuppression-related adverse reactions of anaemia, neutropenia, and thrombocytopenia were verycommonly reported in patients treated with talazoparib 1 mg/day. Grade 3 and Grade 4 myelosuppression-related events were reported for anaemia 34.8% and 0.4%, neutropenia 15.6% and1.8%, and thrombocytopenia 12.8% and 4.0%. No deaths were reported due to myelosuppression-related adverse reactions. Myelosuppression-related adverse events associated withdose modifications were reported for up to approximately 30% of patients in the talazoparib 1 mg/day population and those associated with permanent study drug discontinuation were reported for less than1% of patients.

OVERDOSE

There is limited experience of overdose with talazoparib. No adverse reactions were reported in one patient who accidentally self-administered thirty 1-mg capsules of talazoparib on Day 1 and was immediately treated with gastric decontamination. Symptoms of overdose are not established. In theevent of overdose, treatment with talazoparib should be stopped, and physicians should consider gastric decontamination, follow general supportive measures and treat symptomatically.

25 RETEVMO (SELPERCATINIB)

Inclusion and exclusion sections specific to Selpercatinib:
Standard safety language inclusion criteria
- ANC ⩾1500/µL.
- total bilirubin ⩽1.5X ULN (Except patients with a documented history of Gilbert syndrome who must have a total bilirubin level of <3.0X ULN) and have ALT/AST <2.5X ULN OR <5x ULN if the liver has tumor involvementĐu8226
- normal serum potassium, calcium, and magnesium levels (may be receiving supplements)

Standard safety language exclusion criteria:
- prolongation of the QT interval corrected for heart rate using Fridericia's formula (QTcF) >470 msec
- clinically significant active malabsorption syndrome or other condition likely to affect gastrointestinal absorption of the drug.
- administration of concomitant medication that is known to cause QTc prolongation
- active hemorrhage or at significant risk for hemorrhage

Important Administration Instructions
Selpercatinib may be taken with or without food unless coadministered with a proton pump inhibitor (PPI).

Recommended Dosage
The recommended dosage of selpercatinib based on body weight is:

- Less than 50 kg: 120 mg
- 50 kg or greater: 160 mg

Take selpercatinib orally twice daily (approximately every 12 hours) until disease progression orunacceptable toxicity.

Swallow the capsules whole. Do not crush or chew the capsules.
Do not take a missed dose unless it is more than 6 hours until next scheduled dose.

If vomiting occurs after selpercatinib administration, do not take an additional dose and continue to the next scheduled time for the next dose.

Dosage Modifications for Concomitant Use of Acid-Reducing Agents

Avoid concomitant use of a PPI, a histamine-2 (H2) receptor antagonist, or a locally-acting antacid with selpercatinib. If concomitant use cannot be avoided:

- Take selpercatinib with food when coadministered with a PPI.
-
- Take selpercatinib 2 hours before or 2 hours after administration of a locally-acting antacid.
- Take H2-blockin agent only between 2 and 3 hours after study intervention. If not taken during this time, the dose of H2-blocking agents should not be taken again until 2 to 3 hours after the next dose of study intervention.

Dosage Modifications for Adverse Reactions

The recommended dose reductions for adverse reactions are provided in Table 1.

Table 1 Recommended selpercatinib Dose Reductions for Adverse Reactions

Dose Reduction	Patients Weighing Less Than 50 kg	Patients Weighing 50 kg or Greater
First	80 mg orally twice daily	120 mg orally twice daily
Second	40 mg orally twice daily	80 mg orally twice daily
Third	Not applicable	40 mg orally twice daily

Permanently discontinue selpercatinib in patients unable to tolerate three dose reductions.The recommended dosage modifications for adverse reactions are provided in Table 2.

Table 2 Recommended selpercatinib Dosage Modifications for Adverse Reactions

Adverse Reaction	Severity	Dosage Modification
Hepatotoxicity	Grade 3 or Grade 4	• Withhold selpercatinib and monitor AST/ALT once weekly until resolution to Grade 1 or baseline.<br>• Resume at reduced dose by 2 dose levels and monitor AST and ALT once weekly until 4 weeks after reaching dose taken prior to the onset of Grade 3 or 4 increased AST or ALT.<br>• Increase dose by 1 dose level after a minimum of 2 weeks without recurrence and then increase to dose taken prior to the onset of Grade 3 or 4 increased AST or ALT after a minimum of 4 weeks without recurrence.<br>• Permanently discontinue selpercatinib if grade 3 or 4 increased ALT or AST recur despite dose modifications.
Hypertension	Grade 3	• Withhold selpercatinib for Grade 3 hypertension that persists despite optimal antihypertensive therapy. Resume at a reduced dose when hypertension is controlled.
	Grade 4	• Discontinue selpercatinib.
QT Interval Prolongation	Grade 3	• Withhold selpercatinib until recovery to baseline or Grade 0 or 1.<br>• Resume at a reduced dose.
	Grade 4	• Discontinue selpercatinib
**Adverse Reaction**	**Severity**	**Dosage Modification**
Hemorrhagic Events	Grade 3 or Grade 4	Withhold selpercatinib until recovery to baseline or Grade 0 or 1.<br>Discontinue selpercatinib for severe or life-threatening hemorrhagic events.
Hypersensitivity Reactions	All Grades	Withhold selpercatinib until resolution of the event. Initiatecorticosteroids.<br>Resume at a reduced dose by 3 dose levels while continuing corticosteroids.<br>Increase dose by 1 dose level each week until the dose taken prior to the onset of hypersensitivity is reached, then taper corticosteroids.<br>In case of a clinically significant recurrence of drug hypersensitivity at the initial re-exposure dose of 40 mg BID,

		selpercatinib should be discontinued.
Other Adverse Reactions	Grade 3 or Grade 4	Withhold selpercatinib until recovery to baseline or Grade 0 or 1.<br>Resume at a reduced dose.<br>Discontinue selpercatinib for severe or life-threatening events

Dosage Modifications for Concomitant Use of Strong and Moderate CYP3AInhibitors

Avoid concomitant use of strong and moderate CYP3A inhibitors with selpercatinib. If concomitant use of a strong or moderate CYP3A inhibitor cannot be avoided, reduce the selpercatinib dose as recommended in Table 3. After the inhibitor has been discontinued for 3 to5 elimination half-lives, resume selpercatinib at the dose taken prior to initiating the CYP3A inhibitor.

Table 3 Recommended selpercatinib Dosage for Concomitant Use of Strong and Moderate CYP3A Inhibitors

Current selpercatinib Dosage	Recommended selpercatinib Dosage	
	Moderate CYP3A Inhibitor	Strong CYP3A Inhibitor
120 mg orally twice daily	80 mg orally twice daily	40 mg orally twice daily
160 mg orally twice daily	120 mg orally twice daily	80 mg orally twice daily

Dosage Modification for Severe Hepatic Impairment

Reduce the recommended dosage of selpercatinib for patients with severe hepatic impairment as recommended in Table 4.

Table 4 Recommended selpercatinib Dosage for Severe Hepatic Impairment

Current selpercatinib Dosage	Recommended selpercatinib Dosage
120 mg orally twice daily	80 mg orally twice daily
160 mg orally twice daily	80 mg orally twice daily

WARNINGS AND PRECAUTIONS

Hepatotoxicity

Serious hepatic adverse reactions occurred in 2.6% of patients treated with selpercatinib. Increased AST occurred in 51% of patients, including Grade 3 or 4 events in 8% and increased ALT occurred in 45% of patients, including Grade 3 or 4 events in 9% [see Adverse Reactions (6.1)]. The median time to first onset for increased AST was 4.1 weeks (range: 5 days to 2 years) and increased ALT was 4.1 weeks (range: 6 days to 1.5 years). Monitor ALT and AST prior to initiating selpercatinib, every 2 weeks during the first 3 months, then monthly thereafter and as clinically indicated. Withhold, reduce dose or permanently discontinue selpercatinib based on the severity.

If a patient experiences Grade ≥3 elevated hepatic lab increases, study drug should be held and evaluation for potential alternative causes should be conducted (e.g., history of other hepatotoxic medications/substances, viral serologies, liver imaging). A repeat value 3 to 5 days after the initial finding of elevation of labs should be obtained to confirm the abnormality and to confirm if it is increasing or decreasing. Thereafter, hepatic labs should be monitored at least weekly until resolution to normal/baseline. If the abnormalities do not begin to resolve (or worsen) within 5 days of the AE, a hepatology consultation should be considered to evaluate the need for a liver biopsy. Some patients with persistent lab abnormalities have initiated treatment with steroids, with subsequent improvement.

Upon resolution, selpercatinib may be resumed at a reduced dose of 2 dose levels lower than at which the abnormalities occurred with weekly lab monitoring. In the absence of recurrent abnormalities, the dose of selpercatinib may be escalated sequentially to the next higher dose level after a minimum of 2 weeks at the lowest dose level, and again to the dose level at which the abnormalities occurredafter a minimum of 4 week at the dose level below which the abnormalities occurred. Once the patient has been treated at a stable dose of selpercatinib for a minimum of 4 weeks without recurrent lab abnormalities, the frequency of monitoring may be decreased (e.g., every 2 weeks for 2 cycles and then every cycle thereafter). Permanently discontinue selpercatinib if grade 3 or 4 increased ALT or AST recur despite dose modifications.

Hypertension

Hypertension occurred in 35% of patients, including Grade 3 hypertension in 17% and Grade 4 in one (0.1%) patient. Overall, 4.6% had their dose interrupted and 1.3% had their dose reduced for hypertension. Treatment-emergent hypertension was most commonly managed with anti-hypertension medications. Do not initiate selpercatinib in patients with uncontrolled hypertension. Optimize blood pressure prior to initiating selpercatinib. Monitor blood pressure after 1 week, at least monthly thereafter and as clinically indicated. Initiate or adjust anti-hypertensive therapy as appropriate. Withhold, reduce dose, or permanently discontinue selpercatinib based on the severity.

Hypertension is defined as:

- a sustained increase in blood pressure from baseline, as evidenced by ≥2 readings on ≥2 separate occasions, or
- a clinically significant elevation requiring acute treatment.

If hypertension occurs, study drug may be interrupted at the discretion of the investigator while

- considering initiation of a new anti-hypertensive medication regimen, or
- alteration of a preexisting regimen is optimized to improve BP control; ideally to a reproducible reading of ≤140/90 mmHg.

If study drug is interrupted, it may be resumed at the same or a lower dose at the discretion of the investigator. In all cases, the patient should continue to undergo regular blood pressure monitoring to ensure adequate blood pressure control. If the patient experiences uncontrolled BP ≥ 160/90mmHg despite anti-hypertensive regimen optimization and study drug dose reduction to 40 mg BID, selpercatinib should be discontinued. Study treatment may continue for hypertension that is considered Grade 3 on the basis of requiring ≥ 2 anti-hypertensives or more intensive anti-hypertensive therapy than baseline once blood pressure is stabilized.

QT Interval Prolongation

selpercatinib can cause concentration-dependent QT interval prolongation. An increase in QTcF interval to >500 ms was measured in 6% of patients and an increase in the QTcF interval of at least 60 ms over baseline was measured in 15% of patients. selpercatinib has not been studied in patients with clinically significant active cardiovascular disease or recent myocardial infarction. Monitor patients who are at significant risk of developing QTc prolongation, including patients with known long QT syndromes, clinically significant bradyarrhythmias, and severe or uncontrolled heart failure. Assess QT interval, electrolytes and TSH at baseline and periodically during treatment, adjusting frequency based upon risk factors including diarrhea. Correct hypokalemia, hypomagnesemia and hypocalcemia prior to initiating selpercatinib and during treatment. Monitor the QT interval more frequently when selpercatinib is concomitantly administered with strong and moderate CYP3A inhibitors or drugs known to prolong QTc interval. Withhold and dose reduce or permanently discontinue selpercatinib based on the severity.

Routine ECG monitoring is established into the protocol. Patients must have a QTcF interval of ≤470 ms and serum electrolytes within normal range before starting selpercatinib treatment. Monitor electrocardiograms and serum electrolytes in all patients after 1 week of selpercatinib treatment, at least monthly for the first 6 months of selpercatinib treatment, and otherwise as clinically indicated. The following actions should be taken if the QTcF is greater than 500 msec on at least 2 of 3 ECGs and the triplicate average QTcF is greater than 500 msec.

- Assess for alternative causes (concomitant medications, electrolyte abnormalities, presence of pacemaker). Potassium should be ≥4 meq/L and <ULN and magnesium and calcium should be within normal limits.
- Institutional guidelines or SOC measures for management of QTcF interval >500 msec and/or associated arrhythmias should be initiated.
- Clinical chemistry should be assessed and if electrolytes are abnormal, they should be replete as indicated.

If an alternate cause is not identified, Selpercatinib should be reduced 1 dose level and routine monitoring continued. If the patient experiences grade 3 QTcF interval prolongation, withhold selpercatinib until recovery to baseline or Grade 0 or 1 and resume at a reduced dose. Discontinue selpercatinib if the patient experiences a grade 4 event.

Hemorrhagic Events

Serious including fatal hemorrhagic events can occur with selpercatinib. Grade ≥ 3 hemorrhagic events occurred in 2.3% of patients treated with selpercatinib, including 3 (0.4%) patients with fatal hemorrhagic events, including one case each of cerebral hemorrhage, tracheostomy site hemorrhage, and hemoptysis. Permanently discontinue selpercatinib in patients with severe or life-threatening hemorrhage.

Hypersensitivity

Hypersensitivity occurred in 4.3% of patients receiving selpercatinib, including Grade 3 hypersensitivity in 1.6%. The median time to onset was 1.7 weeks (range: 6 days to 1.5 years). Signs and symptoms of hypersensitivity included fever, rash and arthralgias or myalgias with concurrent decreased platelets or transaminitis. If hypersensitivity occurs, withhold selpercatinib and begin corticosteroids at a dose of 1 mg/kg. Upon resolution of the event, resume selpercatinib at a reduced dose and increase the dose of selpercatinib by 1 dose level each week as tolerated until reaching the dose taken prior to onset of hypersensitivity. Continue steroids until patient reaches target dose and then taper. Permanently discontinue selpercatinib for recurrent

hypersensitivity.
If selpercatinib drug hypersensitivity is suspected, study drug should be held and treatment with steroids at 1 mg/kg prednisone (or equivalent) should be initiated. Upon resolution, selpercatinib may be resumed at a reduced dose of 40 mg BID while continuing steroids at the same dose. Hypersensitivity has recurred in some patients, typically at 3 to 6 hours following drug administration. The following guidelines should be applied if hypersensitivity recurs:

- If recurrence is severe, selpercatinib should again be held; patients with mild recurrence (e.g., isolated instances of rash or myalgias or low-grade fever) have been able to cautiously continue treatment with supportive therapy (e.g., topical treatments, ibuprofen).
- After a minimum of 7 days, and in the absence of clinically significant recurrent drug hypersensitivity, the dose of selpercatinib may be escalated sequentially to 80 mg BID, 120 mg BID, and 160 mg BID for a minimum of 7 days at each dose. Taper steroid dose after selpercatinib has been tolerated for at least 7 days at the final dose. If the patient experiences a clinically significant recurrence of drug hypersensitivity at the initial re-exposure dose of 40 mg BID, selpercatinib should be discontinued.

Risk of Impaired Wound Healing

Impaired wound healing can occur in patients who receive drugs that inhibit the vascular endothelial growth factor (VEGF) signaling pathway. Therefore, selpercatinib has the potential to adversely affect wound healing. Withhold selpercatinib for at least 7 days prior to elective surgery. Do not administer for at least 2 weeks following major surgery and until adequate wound healing. The safety of resumption of selpercatinib after resolution of wound healing complications has not been established.

Embryo-Fetal Toxicity Based on data from animal reproduction studies and its mechanism of action, selpercatinib can cause fetal harm when administered to a pregnant woman. Administration of selpercatinib to pregnant rats during organogenesis at maternal exposures that were approximately equal to those observed at the recommended human dose of 160 mg twice daily resulted in embryolethality and malformations. Advise pregnant women of the potential risk to a fetus..

Reproductive Precautions

Men with partners of childbearing potential or women of childbearing potential must agree to use a highly effective contraceptive method during treatment with study drug and for 6 months following the last dose of study drug. Selpercatinib could impair fertility in males and females. Advise women not to breastfeed during treatment with selpercatinib and for 1 week following the final dose

NOTE: Unless not allowed by local regulations, women of childbearing potential who are abstinent (if this is complete abstinence, as their preferred and usual lifestyle) or in a same-sex relationship (as part of their preferred and usual lifestyle) must agree to either remain abstinent or stay in a same-sex relationship without sexual relations with males. Periodic abstinence (e.g., calendar, ovulation, symptothermal, postovulation methods), declaration of abstinence just for the duration of the trial, and withdrawal are not acceptable methods of contraception.

Women of childbearing potential must have a negative pregnancy test (serum or urine, consistent with local regulations) documented within 24 hours prior to treatment with study drug and at least monthly while on study treatment.

Thrombocytopenia
If a patient is discovered to have thrombocytopenia Grade ≥3, study drug should be held and the patient should be evaluated for alternative causes (medications/substances, viral studies). A hematology consultation may be considered, as necessary, to understand the etiology and to consider a role for concomitant steroid therapy. The patient should undergo weekly CBC testing until the event has recovered to normal/baseline. Upon recovery, the patient should resume selpercatinib at a reduced dose of one level dose reduction with weekly CBC surveillance for 1 full cycle.

ADVERSE REACTIONS

Clinical Trials Experience

Because clinical trials are conducted under widely varying conditions, adverse reaction rates observed in the clinical trials of a drug cannot be directly compared to rates in the clinical trials of another drug and may not reflect the rates observed in practice.

RET Gene Fusion or Gene Mutation Positive Solid Tumors

The pooled safety population described in the WARNINGS and PRECAUTIONS and below reflects exposure to selpercatinib as a single agent at 160 mg orally twice daily evaluated in 702 patients in LIBRETTO-001. Among the 702 patients who received selpercatinib, 65% were exposed for 6 months or longer and 34% were exposed for greater than one year. Among these patients, 95% received at least one dose of selpercatinib at the recommended dosage of 160 mg orally twice daily. The median age was 59 years (range: 15 to 92 years); 0.3% were pediatric patients 12 to 16 years of age; 52% were male; and 69% were White, 22% were Asian, 5% were Hispanic/Latino, and 3% were Black. The most common tumors were NSCLC (47%), MTC (44%), and non- medullary thyroid carcinoma (5%). Serious adverse reactions occurred in 33% of patients who received selpercatinib. The most frequent serious adverse reaction (in ≥ 2% of patients) was pneumonia. Fatal adverse reactions occurred in 3% of patients; fatal adverse reactions which occurred in > 1 patient included sepsis (n = 3), cardiac arrest (n = 3) and respiratory failure (n = 3). Permanent discontinuation due to an adverse reaction occurred in 5% of patients who received selpercatinib. Adverse reactions resulting in permanent discontinuation included increased ALT (0.4%), sepsis (0.4%), increased AST (0.3%), drug hypersensitivity (0.3%), fatigue (0.3%), and thrombocytopenia (0.3%). Dosage interruptions due to an adverse reaction occurred in 42% of patients who received selpercatinib. Adverse reactions requiring dosage interruption in > 2% of patients included ALT increased, AST increased, hypertension, diarrhea, pyrexia, and QT prolongation. Dose reductions due to an adverse reaction occurred in 31% of patients who received selpercatinib. Adverse reactions requiring dosage reductions in > 2% of patients included ALT increased, AST increased, QT prolongation and fatigue. The most common adverse reactions, including laboratory abnormalities, (≥ 25%) were increased aspartate aminotransferase (AST), increased alanine aminotransferase (ALT), increased glucose, decreased leukocytes, decreased albumin, decreased calcium, dry mouth, diarrhea, increased creatinine, increased alkaline phosphatase, hypertension, fatigue, edema, decreased platelets, increased total cholesterol, rash, decreased sodium, and constipation.

Table 5 summarizes the adverse reactions in LIBRETTO-001.

	**selpercatinb<br>(n = 702)**

Adverse Reaction	Grades 1-4 (%)	Grades 3-4 (%)
**Gastrointestinal**		
Dry Mouth	39	0
Diarrhea[1]	37	3.4*
Constipation	25	0.6*
Nausea	23	0.6*
Abdominal pain[2]	23	1.9*
Vomiting	15	0.3*
**Vascular**		
Hypertension	35	18
**General**		
Fatigue[3]	35	2*
Edema[4]	33	0.3*
**Skin**		
Rash[5]	27	0.7*
**Nervous System**		
Headache[6]	23	1.4*
**Respiratory**		
Cough[7]	18	0
Dyspnea[8]	16	2.3
**Investigations**		
Prolonged QT interval	17	4*
**Blood and Lymphatic System**		
Hemorrhage[9]	15	1.9

[1]Diarrhea includes diarrhea, defecation urgency, frequent bowel movements, and anal incontinence
[2]Abdominal pain includes abdominal pain, abdominal pain upper, abdominal pain lower, abdominal discomfort,gastrointestinal pain
[3]Fatigue includes fatigue, asthenia, malaise.
[4]Edema includes edema, edema peripheral, face edema, eye edema, eyelid edema, generalized edema, localized edema, lymph edema, scrotal edema, peripheral swelling, scrotal swelling, swelling, swelling face, eye swelling, peripheral swelling
[5]Includes rash, rash erythematous, rash macular, rash maculopapular, rash morbilliform, rash pruritic
[6]Headache includes headache, sinus headache, tension headache
[7]Includes cough, productive cough
[8]Includes dyspnea, dyspnea exertional, dyspnea at rest
[9]Hemorrhage includes epistaxis, hematuria, hemoptysis, contusion, rectal hemorrhage, vaginal hemorrhage, ecchymosis, hematochezia, petechiae, traumatic hematoma, anal hemorrhage, blood blister, blood urine present, cerebral hemorrhage, gastric hemorrhage, hemorrhage intracranial,

spontaneous hematoma, abdominal wall hematoma, angina bullosa hemorrhagica, diverticulum intestinal hemorrhagic, eye hemorrhage, gastrointestinalhemorrhage, gingival bleeding, hematemesis, hemorrhagic anemia, intraabdominal hemorrhage, lower gastrointestinal hemorrhage, melena, mouth hemorrhage, occult blood positive, pelvic hematoma, periorbital hematoma, pharyngeal hemorrhage, pulmonary contusion, purpura, retroperitoneal hematoma, subarachnoid hemorrhage, subdural hemorrhage, upper gastrointestinal hemorrhage, vessel puncture site hematoma

*Only includes a grade 3 adverse reaction.

Clinically relevant adverse reactions in ≤ 15% of patients who received selpercatinib includehypothyroidism (9%).

Table 6 summarizes the laboratory abnormalities in LIBRETTO-001.

Table 6 Select Laboratory Abnormalities (≥ 20%) Worsening from Baseline in Patients Who Received selpercatinib in LIBRETTO-001

Laboratory Abnormality	selpercatinib[1]	
	Grades 1-4 (%)	Grades 3-4 (%)
**Chemistry**		
Increased AST	51	8
Increased ALT	45	9
Increased glucose	44	2.2
Decreased albumin	42	0.7
Decreased calcium	41	3.8
Increased creatinine	37	1.0
Increased alkaline phosphatase	36	2.3
Increased total cholesterol	31	0.1
Decreased sodium	27	7
Decreased magnesium	24	0.6
Increased potassium	24	1.2
Increased bilirubin	23	2.0
Decreased glucose	22	0.7
**Hematology**		
Decreased leukocytes	43	1.6
Decreased platelets	33	2.7

1Denominator for each laboratory parameter is based on the number of patients with a baseline and post-treatment laboratoryvalue available, which ranged from 675 to 692 patients.

<u>Increased Creatinine</u>

In healthy subjects administered selpercatinib 160 mg orally twice daily, serum creatinine

increased 18% after 10 days. Consider alternative markers of renal function if persistent elevations in serum creatinine are observed.

DRUG INTERACTIONS

Effects of Other Drugs on selpercatinib

Acid-Reducing Agents

Concomitant use of selpercatinib with acid-reducing agents decreases selpercatinib plasma concentrations, which may reduce selpercatinib anti-tumor activity. Avoid concomitant use of PPIs, H2 receptor antagonists, and locally-acting antacids with selpercatinib. If coadministration cannot be avoided, take selpercatinib with food (with a PPI) or modify its administration time (with a H2 receptor antagonist or a locally-acting antacid).

Strong and Moderate CYP3A Inhibitors

Concomitant use of selpercatinib with a strong or moderate CYP3A inhibitor increases selpercatinib plasma concentrations, which may increase the risk of selpercatinib adverse reactions, including QTc interval prolongation. Avoid concomitant use of strong and moderate CYP3A inhibitors with selpercatinib. If concomitant use of strong and moderate CYP3A inhibitors cannot be avoided, reduce the selpercatinib dosage and monitor the QT interval with ECGs more frequently.

Strong and Moderate CYP3A Inducers

Concomitant use of selpercatinib with a strong or moderate CYP3A inducer decreases selpercatinib plasma concentrations, which may reduce selpercatinib anti-tumor activity. Avoid coadministration of strong or moderate CYP3A inducers with selpercatinib.

Effects of selpercatinib on Other Drugs

CYP2C8 and CYP3A Substrates

Selpercatinib is a moderate CYP2C8 inhibitor and a weak CYP3A inhibitor. Concomitant use of selpercatinib with CYP2C8 and CYP3A substrates increases their plasma concentrations, which may increase the risk of adverse reactions related to these substrates. Avoid coadministration of selpercatinib with CYP2C8 and CYP3A substrates where minimal concentration changes may lead to increased adverse reactions. If coadministration cannot be avoided, follow recommendations for CYP2C8 and CYP3A substrates provided in their approved product labeling.

Drugs that Prolong QT Interval
The concurrent use of drugs known to prolong QTc is prohibited. Drugs with a possible or conditional risk should be avoided if possible.
Selpercatinib is associated with QTc interval prolongation. Monitor the QT interval with ECGs more frequently in patients who require treatment with concomitant medications known to prolong the QT interval.

Geriatric Use
Of 702 patients who received selpercatinib, 34% (239 patients) were ≥ 65 years of age and 10% (67 patients) were ≥ 75 years of age. No overall differences were observed in the safety or effectiveness of selpercatinib between patients who were ≥ 65 years of age and younger patients.

Renal Impairment

No dosage modification is recommended for patients with mild to moderate renal impairment (creatinine clearance [CLcr] > 30 mL/min, estimated by Cockcroft-Gault). The recommended dosage has not been established for patients with severe renal impairment (CLcr < 30 mL/min) or end-stage renal disease.

Hepatic Impairment

Reduce the dose when administering selpercatinib to patients with severe [total bilirubin greater than 3 to 10 times upper limit of normal (ULN) and any AST] hepatic impairment. No dosage modification is recommended for patients with mild (total bilirubin less than or equal to ULN with AST greater than ULN or total bilirubin greater than 1 to 1.5 times ULN with any AST) or moderate (total bilirubin greater than 1.5 to 3 times ULN and any AST) hepatic impairment. Monitor for selpercatinib-related adverse reactions in patients with hepatic impairment.

CONSORT 2010 checklist of information to include when reporting a randomised trial*

Section/Topic	Item No	Checklist item	Reported on page No
**Title and abstract**			
	1a	Identification as a randomised trial in the title	1
	1b	Structured summary of trial design, methods, results, and conclusions (for specific guidance see CONSORT for abstracts)	2
**Introduction**			
Background and objectives	2a	Scientific background and explanation of rationale	3
	2b	Specific objectives or hypotheses	3-4
**Methods**			
Trial design	3a	Description of trial design (such as parallel, factorial) including allocation ratio	10 to 13
	3b	Important changes to methods after trial commencement (such as eligibility criteria), with reasons	
Participants	4a	Eligibility criteria for participants	10 to 11
	4b	Settings and locations where the data were collected	11-12
Interventions	5	The interventions for each group with sufficient details to allow replication, including how and when they were actually administered	4-5 and 10-11
Outcomes	6a	Completely defined pre-specified primary and secondary outcome measures, including how and when they were assessed	12
	6b	Any changes to trial outcomes after the trial commenced, with reasons	n/a
Sample size	7a	How sample size was determined	13
	7b	When applicable, explanation of any interim analyses and stopping guidelines	13
Randomisation:			
Sequence generation	8a	Method used to generate the random allocation sequence	12 and 13
	8b	Type of randomisation; details of any restriction (such as blocking and block size)	11 and 12
Allocation concealment mechanism	9	Mechanism used to implement the random allocation sequence (such as sequentially numbered containers), describing any steps taken to conceal the sequence until interventions were assigned	10-13
Implementation	10	Who generated the random allocation sequence, who enrolled participants, and who assigned participants to interventions	10 to 13
Blinding	11a	If done, who was blinded after assignment to interventions (for example, participants, care providers, those	n/a

		assessing outcomes) and how	
	11b	If relevant, description of the similarity of interventions	
Statistical methods	12a	Statistical methods used to compare groups for primary and secondary outcomes	13
	12b	Methods for additional analyses, such as subgroup analyses and adjusted analyses	13
**Results**			
Participant flow (a diagram is strongly recommended)	13a	For each group, the numbers of participants who were randomly assigned, received intended treatment, and were analysed for the primary outcome	4
	13b	For each group, losses and exclusions after randomisation, together with reasons	4 and consort diagram
Recruitment	14a	Dates defining the periods of recruitment and follow-up	4
	14b	Why the trial ended or was stopped	4
Baseline data	15	A table showing baseline demographic and clinical characteristics for each group	4 and table 1
Numbers analysed	16	For each group, number of participants (denominator) included in each analysis and whether the analysis was by original assigned groups	4 to 6
Outcomes and estimation	17a	For each primary and secondary outcome, results for each group, and the estimated effect size and its precision (such as 95% confidence interval)	4 to 6
	17b	For binary outcomes, presentation of both absolute and relative effect sizes is recommended	4 and supplementar y
Ancillary analyses	18	Results of any other analyses performed, including subgroup analyses and adjusted analyses, distinguishing pre-specified from exploratory	4 to 6
Harms	19	All important harms or unintended effects in each group (for specific guidance see CONSORT for harms)	n/a
**Discussion**			
Limitations	20	Trial limitations, addressing sources of potential bias, imprecision, and, if relevant, multiplicity of analyses	6 and 7
Generalisability	21	Generalisability (external validity, applicability) of the trial findings	6-8
Interpretation	22	Interpretation consistent with results, balancing benefits and harms, and considering other relevant evidence	8
**Other information**			
Registration	23	Registration number and name of trial registry	16
Protocol	24	Where the full trial protocol can be accessed, if available	Included in submission
Funding	25	Sources of funding and other support (such as supply of drugs), role of funders	15

*We strongly recommend reading this statement in conjunction with the CONSORT 2010 Explanation and Elaboration for important clarifications on all the items. If relevant, we also recommend reading CONSORT extensions for cluster randomised trials, non-inferiority and equivalence trials, non-pharmacological treatments, herbal interventions, and pragmatic trials. Additional extensions are forthcoming: for those and for up to date references relevant to this checklist, see www.consort-statement.org.
