# Supplementary material for: Genomically matched therapy in advanced solid tumors: the randomized phase 2 ROME trial
Source: Nat Med. 2025 Sep 29;31(10):3514–23. doi: 10.1038/s41591-025-03918-x (PMC12532583; doi:10.1038/s41591-025-03918-x)
Supplement: Supplementary file 2 — Reporting Summary [file 41591_2025_3918_MOESM2_ESM.pdf]

nature portfolio

Corresponding author(s): Simone Scagnoli
Last updated by author(s): 26.05.2025

Reporting Summary

Nature Portfolio wishes to improve the reproducibility of the work that we publish. This form provides structure for consistency and transparency in reporting. For further information on Nature Portfolio policies, see our Editorial Policies and the Editorial Policy Checklist.

Statistics

For all statistical analyses, confirm that the following items are present in the figure legend, table legend, main text, or Methods section.

n/a	Confirmed	
☐	☒	The exact sample size (*n*) for each experimental group/condition, given as a discrete number and unit of measurement
☐	☒	A statement on whether measurements were taken from distinct samples or whether the same sample was measured repeatedly
☐	☒	The statistical test(s) used AND whether they are one- or two-sided<br>*Only common tests should be described solely by name; describe more complex techniques in the Methods section.*
☒	☐	A description of all covariates tested
☒	☐	A description of any assumptions or corrections, such as tests of normality and adjustment for multiple comparisons
☐	☒	A full description of the statistical parameters including central tendency (e.g. means) or other basic estimates (e.g. regression coefficient) AND variation (e.g. standard deviation) or associated estimates of uncertainty (e.g. confidence intervals)
☐	☒	For null hypothesis testing, the test statistic (e.g. *F*, *t*, *r*) with confidence intervals, effect sizes, degrees of freedom and *P* value noted<br>*Give P values as exact values whenever suitable.*
☒	☐	For Bayesian analysis, information on the choice of priors and Markov chain Monte Carlo settings
☒	☐	For hierarchical and complex designs, identification of the appropriate level for tests and full reporting of outcomes
☒	☐	Estimates of effect sizes (e.g. Cohen's *d*, Pearson's *r*), indicating how they were calculated

Our web collection on statistics for biologists contains articles on many of the points above.

Software and code

Policy information about availability of computer code

Data collection	Study customized EDC system NPC data V. 1.0
Data analysis	software SAS version 9.4

For manuscripts utilizing custom algorithms or software that are central to the research but not yet described in published literature, software must be made available to editors and reviewers. We strongly encourage code deposition in a community repository (e.g. GitHub). See the Nature Portfolio guidelines for submitting code & software for further information.

Data

Policy information about availability of data

All manuscripts must include a data availability statement. This statement should provide the following information, where applicable:

- Accession codes, unique identifiers, or web links for publicly available datasets
- A description of any restrictions on data availability
- For clinical datasets or third party data, please ensure that the statement adheres to our policy

Individual de-identified participant data generated during the current study are available upon reasonable request from academic or qualified clinical researchers affiliated
with recognized institutions, strictly for the purpose of conducting non-commercial, ethically approvable research aligned with the original scope of the trial.
Applicants are required to submit a detailed research proposal, curriculum vitae, and a declaration of non-conflict of interest.

Requests must clearly describe the research objectives and methodology, and must be reviewed and approved by the Steering Committee of the Rome Trial during dedicated review sessions. Approval is granted based on scientific merit, data availability, intended data use, and absence of overlapping research initiatives by the trial investigators.
All approved requestors will be required to sign a Data Access Agreement that restricts data use solely to the approved research project and prohibits any further distribution or use.
Data will be shared via a secure data-sharing platform within 4 to 8 weeks of approval, contingent upon data volume and complexity.
Data requests will be considered within 12 months of manuscript publication.
The trial registration, study protocol, and methodological details are publicly accessible through ClinicalTrials.gov (accession code: NCT04591431; https://clinicaltrials.gov/ct2/show/NCT04591431).
Additional publicly available datasets used in the analysis include:
ClinVar database: freely accessible at https://www.ncbi.nlm.nih.gov/clinvar/
OncoKB database: accessible at https://www.oncokb.org/
COSMIC database: accessible at https://cancer.sanger.ac.uk/cosmic
ESMO ESCAT scale: accessible at https://www.esmo.org/guidelines/esmo-scale-for-clinical-actionability-of-molecular-targets-escat
No other public repositories or datasets requiring accession codes were utilized in this study

Research involving human participants, their data, or biological material

Policy information about studies with human participants or human data. See also policy information about sex, gender (identity/presentation), and sexual orientation and race, ethnicity and racism.

Reporting on sex and gender	The manuscript reports anonimyzed and non-individual informations about patient's sex
Reporting on race, ethnicity, or other socially relevant groupings	the manuscript reports anomyzed and non-individual infortmations about ethnicity
Population characteristics	able 1 reports the following anonimyzed and non-indivdual patiens characteristics: Age (years); Gender; Ethnicity, primary tumor, PS-ECOG, previous lines of treatment
Recruitment	Participants in the ROME trial were recruited prospectively from specialized oncology centers. Eligible consecutive patients were identified and invited to participate based on clear inclusion and exclusion criteria detailed in the published protocol (NCT04591431). Recruitment involved direct clinician referral and systematic screening of eligible patients from participating institutions. Considering the broad inclusion criteria, the agnostic purpose of the trial, and that all the procedures and the NGS testing were included in the trial procedures, no selection bias or self-selection bias is expected.
Ethics oversight	The study was approved by the Institutional Ethical Committee of the coordinating center (Sapienza no. rif. C.E. 5575 - 02/2020) and by the Ethical Committee of each participating center. The investigational sites that approved the study protocol were: AOU Policlinico Umberto I, Rome; European Institute of Oncology (IEO), IRCCS; Milan; Veneto Institute of Oncology IOV-IRCCS, Padua; Division of Medical Oncology, Pisa University Hospital; IRCCS Istituto Romagnolo per lo Studio dei Tumori (IRST) “Dino Amadori”, Meldola; Istituto Nazionale Tumori IRCCS Fondazione "G. Pascale", Naples; IRCCS Istituto Nazionale Tumori Regina Elena (IRE), Rome; IRCCS - Istituto di Candiolo, Candiolo; Medical Oncology Unit -ARNAS Garibaldi Catania; Azienda Ospedaliera Santa Maria di Terni, Terni; Pederzoli Hospital, Peschiera del Garda; IRCSS Ospedale Policlinico San Martino, Genoa; Ospedali Galliera, Genoa; Central Hospital of Belcolle, Viterbo; IRCCS Sacro Cuore Don Calabria Hospital, Negrar di Valpolicella; Santa Maria della Misericordia Hospital, Perugia; Nuovo Ospedale di Prato-Santo Stefano, Azienda USL Toscana Centro, Prato; CRO Aviano, National Cancer Institute, IRCCS, Aviano; Santa Maria delle Croci Hospital, AUSL Romagna, Ravenna; Oncology Unit ASST Papa Giovanni XXIII, Bergamo; Ordine Mauriziano Hospital, Turin; AOU Policlinico S. Andrea, Rome; Azienda Ospedaliera Universitaria Federico II, Napoli; Fondazione IRCCS Istituto Tumori di Milano; Fondazione Policlinico Universitario Campus Bio-Medico, Rome; A.O. Papardo-Messina; IRCCS Istituto Tumori "Giovanni Paolo II", Bari; Policlinico universitario “Mater Domini”, Catanzaro; Centro Oncologico San Leopoldo Mandic, Isola Tiberina Gemelli isola; Rome; University Hospital, Ferrara; Misericordia Hospital, Grosseto; AUSL Piacenza Guglielmo da Saliceto Hospital, Piacenza; Comprehensive Cancer Centre, AUSL-IRCCS di Reggio Emilia, Reggio Emilia; Humanitas Gradenigo, Torino; Foundation IRCCS Casa Sollievo della Sofferenza, San Giovanni Rotondo; Ramazzini Hospital, Azienda Unità Sanitaria Locale Modena (AUSL), Carpi; AOUP "Paolo Giaccone", Palermo; AOU delle Marche, Ancona.<br>The competent authority Agenzia Italiana del Farmaco (AIFA) authorized the trial on 08/July/2020 (AIFA/SC/P/76132). The trial was registered in ClinicalTrials.gov with ClinicalTrials.gov ID NCT04591431. EudraCT number: 2018-002190-21. The trial adhered to the principles of the Declaration of Helsinki regarding research involving human subjects. 41 centers received ethical approval and participated in the study enrollment. All patients signed the specifically conceived informed consent form.

Note that full information on the approval of the study protocol must also be provided in the manuscript.

Field-specific reporting

Please select the one below that is the best fit for your research. If you are not sure, read the appropriate sections before making your selection.

☒ Life sciences ☐ Behavioural & social sciences ☐ Ecological, evolutionary & environmental sciences

For a reference copy of the document with all sections, see nature.com/documents/nr-reporting-summary-flat.pdf

Life sciences study design

All studies must disclose on these points even when the disclosure is negative.

Sample size	We hypothesized that tailored therapy (TT) would yield higher ORR compared to SoC, specifically a 20% ORR for TT versus 5% for SoC. According to the site of primary tumor, four cohorts were defined as breast cancer (stratum A), non-colorectal gastrointestinal cancers (stratum B), NSCLC (stratum C), and other malignancies (stratum D), with competitive enrollment across these strata. To detect a 15% difference between the two arms, assuming an alpha of 0.10, a beta of 0.20 (80% power), and employing a one-sided Chi-square test, a total of 86 patients (43 in each arm of the four cohorts) were required.
Data exclusions	no data were excluded. CONSORT diagram is included in the manuscript
Replication	Next-generation sequencing (NGS) tests were performed centrally by Foundation One Inc., utilizing standardized protocols and comprehensive quality control measures provided by the company. Each test was conducted only once per tissue (paraffin-embedded slides) and blood samples. Residual tissue samples, if any, were returned to the recruiting centers, which managed their return to local pathology archives to remain available for the patient. Blood samples could only be used once, making the test inherently non-repeatable.
Randomization	patients were randomized 1:1 to receive Tailored Treatment (TT) or Standard of Care (SoC). SAP 2.0 is provided
Blinding	open randomization

Reporting for specific materials, systems and methods

We require information from authors about some types of materials, experimental systems and methods used in many studies. Here, indicate whether each material, system or method listed is relevant to your study. If you are not sure if a list item applies to your research, read the appropriate section before selecting a response.

Materials & experimental systems

n/a	Involved in the study	
☒	☐	Antibodies
☒	☐	Eukaryotic cell lines
☒	☐	Palaeontology and archaeology
☒	☐	Animals and other organisms
☐	☒	Clinical data
☒	☐	Dual use research of concern
☒	☐	Plants

Methods

n/a	Involved in the study	
☒	☐	ChIP-seq
☒	☐	Flow cytometry
☒	☐	MRI-based neuroimaging

Clinical data

Policy information about clinical studies

All manuscripts should comply with the ICMJE guidelines for publication of clinical research and a completed CONSORT checklist must be included with all submissions.

Clinical trial registration	The competent authority Agenzia Italiana del Farmaco (AIFA) authorized the trial on 08/July/2020 (AIFA/SC/P/76132). The trial was registered in ClinicalTrials.gov with ClinicalTrials.gov ID NCT04591431. EudraCT number: 2018-002190-21.
Study protocol	included in the supplementary materials
Data collection	Data were collected from every site investigators in a specific eCRF. Recruitment was active from 13th October 2020 to 19th July 2023. Data collection ended on 30/Jan/2025 due to database lock. 1794 patients were screened. 400 were randomized.
Outcomes	The primary endpoint of the study was the overall response rate (ORR), defined as the proportion of patients achieving a complete response (CR) or partial response (PR), respect to the total number of randomized patients. Secondary endpoints included progression-free survival (PFS), defined as the time from randomization to disease progression or death, whichever occurred first; time to treatment failure (TTF), defined as the time from randomization until patient withdrawal for any reason including disease progression or death; time to next treatment (TTNT), defined as the time from randomization to the start of the next line of therapy; overall survival (OS), defined as the time from randomization to death from any cause and safety. Tumor response was locally assessed according to the RECIST version 1.1 and irRC criteria. Adverse events (AEs) were graded using the Common Terminology Criteria for Adverse Events (CTCAE) version 5.0. Additional planned secondary endpoints not reported in this manuscript are: concordance between molecular profile on tumor tissue and ctDNA, Quality of Life (QoLs), the immune fitness in the two treatment arms, and the association between the molecular evaluation and gene expression profiling.

Plants

Seed stocks	*Report on the source of all seed stocks or other plant material used. If applicable, state the seed stock centre and catalogue number. If plant specimens were collected from the field, describe the collection location, date and sampling procedures.*
Novel plant genotypes	*Describe the methods by which all novel plant genotypes were produced. This includes those generated by transgenic approaches, gene editing, chemical/radiation-based mutagenesis and hybridization. For transgenic lines, describe the transformation method, the number of independent lines analyzed and the generation upon which experiments were performed. For gene-edited lines, describe the editor used, the endogenous sequence targeted for editing, the targeting guide RNA sequence (if applicable) and how the editor was applied.*
Authentication	*Describe any authentication procedures for each seed stock used or novel genotype generated. Describe any experiments used to assess the effect of a mutation and, where applicable, how potential secondary effects (e.g. second site T-DNA insertions, mosiacism, off-target gene editing) were examined.*
